# Modular assembly of amines and diborons with photocatalysis enabled halogen atom transfer of organohalides for C(sp^3^)–C(sp^3^) bond formation†

**DOI:** 10.1039/d5sc00190k

**Published:** 2025-01-24

**Authors:** Rong-Bin Liang, Ting-Ting Miao, Xiang-Rui Li, Jia-Bo Huang, Shao-Fei Ni, Sanliang Li, Qing-Xiao Tong, Jian-Ji Zhong

**Affiliations:** a College of Chemistry and Chemical Engineering, Key (Guangdong-Hong Kong Joint) Laboratory for Preparation and Application of Ordered Structural Materials of Guangdong Province, Shantou University Shantou 515063 P. R. China jjzhong@stu.edu.cn; b Chemistry and Chemical Engineering Guangdong Laboratory Shantou 515063 P. R. China

## Abstract

In the past few years, the direct activation of organohalides by ligated boryl radicals has emerged as a potential synthetic tool for cross-coupling reactions. In most existing methods, ligated boryl radicals are accessed from NHC-boranes or amine-boranes. In this work, we report a new photocatalytic platform by modular assembly of readily available amines and diboron esters to access a library of ligated boryl radicals for reaction screening, thus enabling the cross-coupling of organohalides and alkenes including both activated and unactivated ones for C(sp^3^)–C(sp^3^) bond formation by using the assembly of DABCO A1 and B_2_Nep_2_B1. The strategy features operational simplicity, mild conditions and good functional group tolerance. A range of organohalides including activated alkyl chlorides, alkyl bromides (1°, 2° and 3° C–Br) as well as aromatic bromides are applicable in the strategy. Experimental and computational studies rationalize the proposed mechanism.

## Abstract 2

A new photocatalytic approach by using the assembly of DABCO A1 and B_2_Nep_2_B1 has been established to enable the cross-coupling of organohalides and alkenes for C(sp^3^)–C(sp^3^) bond formation.

## Graphical abstract

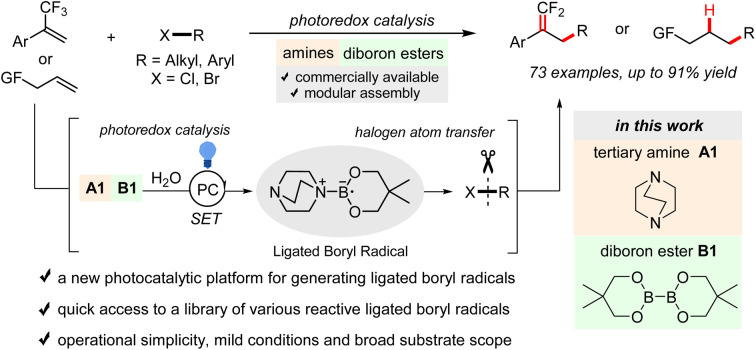

## Introduction

**Scheme 1:**
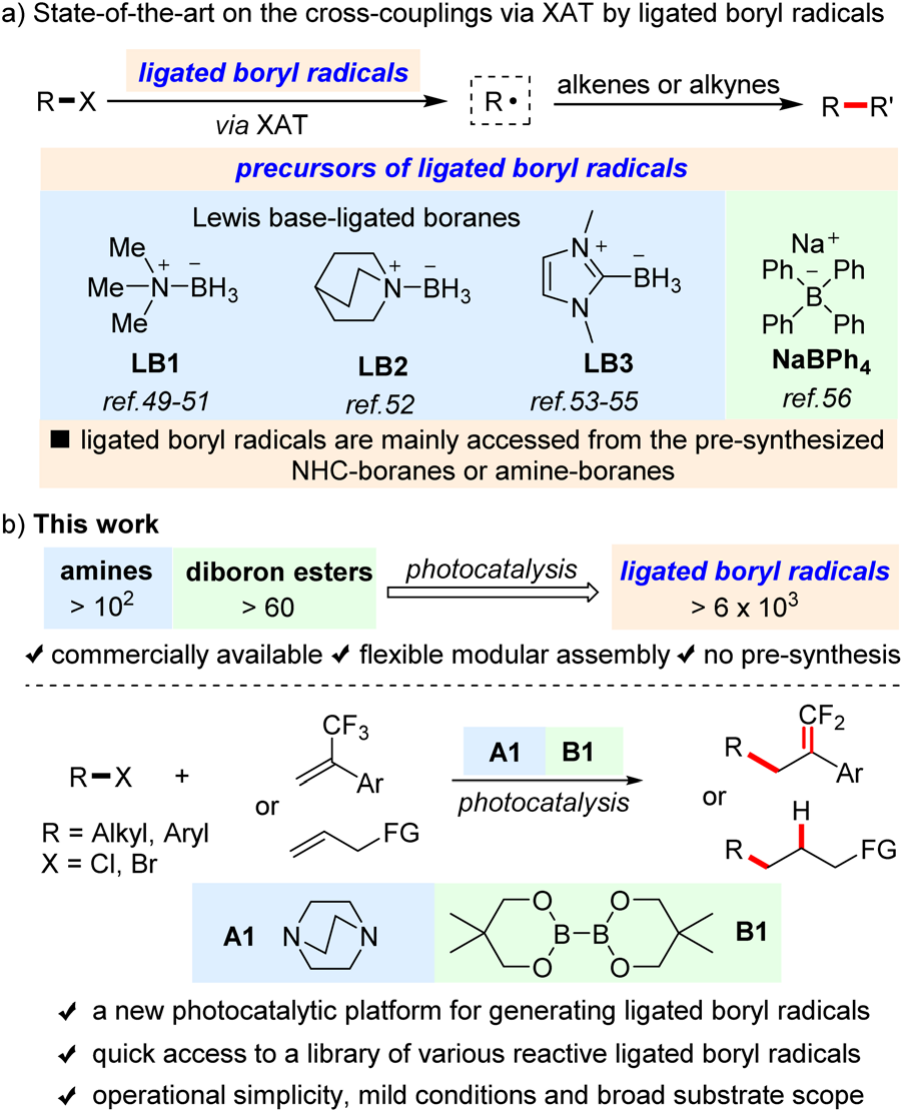
(a) State-of-the-art of the cross-couplings *via* XAT by ligated boryl radicals. (b) This work.

Halogen atom transfer (XAT) of organohalides to the corresponding carbon-centered radicals for subsequent transformations is a powerful synthetic strategy in organic synthesis.^1,2^ Historically, tin-containing reagents as efficient halogen abstractors have made great contributions to this field.^3,4^ However, the safety and selectivity concerns about tin-containing reagents drive an ever-increasing interest in seeking alternatives.^5^ In the last few decades, photoredox catalysis^6–17^ has emerged as a green and efficient platform for the activation of organohalides *via* XAT by silyl radicals^18–23^ and α-aminoalkyl radicals.^24–27^ In recent years, photocatalytically generated ligated boryl radicals (LBRs), which exhibit a comparable performance in halogen atom abstraction, are found to be a promising alternative. Nevertheless, compared with silyl radicals and α-aminoalkyl radicals, ligated boryl radicals^28–48^ mediated XAT for cross-couplings is still in its infancy and has been less explored. To the best of our knowledge, only several successful examples have so far been reported. For example, as illustrated in Scheme 1a, Wu^49^ and Leonori,^50,51^ respectively, reported that tertiary amine-borane LB1 was able to activate the C–Cl bond of Freon-22 (ClCF_2_H) or the C–I/Br bond of alkyl halides for alkylation of alkenes or alkynes under photocatalysis. Subsequently, Wu disclosed that tertiary amine-borane LB2 with photocatalysis could activate the C–Cl bond of ClCF_2_R for difluoromethylation of alkenes.^52^ In addition, the groups of Noël,^53^ Capaldo,^54^ and Fan,^55^ respectively, reported that N-heterocyclic carbene–borane LB3 with photocatalysis could activate the C–I bond of alkyl iodides to initiate Giese-type reactions. Very recently, Sharma^56^ reported that, after photocatalytic single-electron oxidation, sodium tetraphenylborate (NaBPh_4_) could generate a reactive boryl radical to enable hydroalkylation of styrenes with alkyl iodides or bromides. Although such progress has been made, the ligated boryl radicals in the existing methods are mainly accessed from pre-synthesized NHC-boranes or amine-boranes, and the boryl-radical precursors are limited to a few Lewis base-ligated boranes, which restricts the application of ligated boryl radical-mediated XAT for cross-coupling reactions. Therefore, the development of new methodologies to generate various reactive ligated boryl radicals will be interesting and highly desirable. In this work, we report a new photocatalytic platform by modular assembly of commercially available amines and diboron esters, which provides a simple and convenient approach to access a library of various reactive ligated boryl radicals for reaction screening, thus enabling the cross-coupling of organohalides and alkenes including both the activated and unactivated ones for C(sp^3^)–C(sp^3^) bond formation by using the assembly of DABCO A1 and B_2_Nep_2_B1 under photocatalysis.

## Results and discussion

Dichloromethane (DCM), one of the most ubiquitous solvents, is widely used in chemical research and industry. It would be a desirable C1 synthon if the ligated boryl radical could selectively activate the C–Cl bond. However, due to its inertness and low reactivity, the activation of C–Cl bond in dichloromethane for organic transformations is still rather limited.^57^ To this end, we commenced our investigations by evaluating the activation of dichloromethane using various reactive ligated boryl radicals, which were *in situ* generated from the platform of modular assembly of commercially available amines and diboron esters under photocatalysis. After extensive screenings, we were delighted to find that when α-trifluoromethyl arylalkene S-1 was selected as the substrate, the assembly of tertiary amine A1 and diboron ester B1 with Ir(dFMeppy)_2_(dtbbpy)PF_6_ as a photocatalyst could successfully furnish the desired chloromethylation product, the *gem*-difluoroalkene 1, in 91% yield with excellent chemo-selectivity at room temperature under 450 nm LED irradiation for 6 h (Table 1, entry 1). Herein, dichloromethane served not only as a raw material, but also as a solvent. Replacing the photocatalyst with 4CzIPN resulted in a decreased yield of 72% (entry 2). Trace amount of the desired product was observed when other photocatalysts such as Mes-Acr-Me^+^ClO_4_^−^, Ru(bpy)_3_(PF_6_)_2_, or Ir(ppy)_3_ was used (entries 3–5). Moreover, other assemblies of amines and diboron esters were examined (entries 6–16), and the assembly of A1 and B1 proved to be the best. Additionally, several NHC-boranes or amine-boranes, which are commonly used precursors of ligated boryl radicals under photocatalysis, were tested, however, they could not accelerate this transformation in a satisfactory yield (entries 17–20), demonstrating the advantages of this flexible platform. Finally, no desired product was detected when the reaction took place in the absence of the photocatalyst, light, A1 or B1, indicating the essential role of all these parameters (entry 21).

**Table 1:** Optimization of the reaction parameters^a^

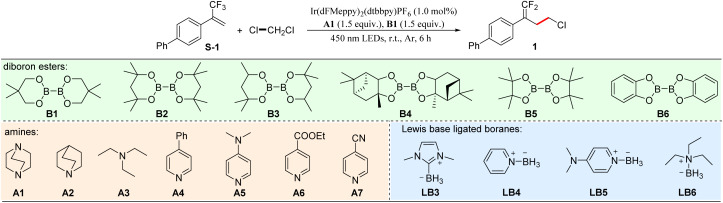		
Entry	Variations from the standard conditions	1^b^ (%)
1	None	91
2	4CzIPN instead of Ir(dFMeppy)_2_(dtbbpy)PF_6_	72
3	Mes-Acr-Me^+^ClO_4_^−^ instead of Ir(dFMeppy)_2_(dtbbpy)PF_6_	Trace
4	Ru(bpy)_3_(PF_6_)_2_ instead of Ir(dFMeppy)_2_(dtbbpy)PF_6_	Trace
5	Ir(ppy)_3_ instead of Ir(dFMeppy)_2_(dtbbpy)PF_6_	Trace
6	B2 instead of B1	72
7	B3 instead of B1	83
8	B4 instead of B1	67
9	B5 instead of B1	76
10	B6 instead of B1	Trace
11	A2 instead of A1	24
12	A3 instead of A1	12
13	A4 instead of A1	56
14	A5 instead of A1	41
15	A6 instead of A1	14
16	A7 instead of A1	NR
17	LB3 instead of A1&B1	29
18	LB4 instead of A1&B1	36
19	LB5 instead of A1&B1	27
20	LB6 instead of A1&B1	Trace
21	Without Ir(dFMeppy)_2_(dtbbpy)PF_6_, light, A1, or B1	NR

a Standard conditions: S-1 (0.2 mmol), photocatalyst (1.0 mol%), A1 (0.3 mmol), B1 (0.3 mmol), CH_2_Cl_2_ (2.0 mL), r.t., Ar atmosphere, and 450 nm LED irradiation for 6 h.

b Isolated yields.

To verify the generality of this photocatalytic protocol for the C–Cl activation reaction, we first evaluated the scope of alkenes (Table 2). To our delight, various α-trifluoromethyl arylalkenes bearing different functional groups, regardless of electron-donating or electron-withdrawing groups, on the aromatic ring were all well tolerated, giving the corresponding chloromethylation *gem*-difluoroalkenes 1–14 in good to excellent yields. Notably, when the *para*-position of the aromatic ring was substituted by a strong electron-withdrawing group such as the cyano group, besides the desired product 15, the hydrochloromethylation of alkene was competitive to afford a byproduct 15′, probably due to the fact that delocalization of the electron density into the electron-poor ring slows down the fluoride elimination. Trifluoromethyl alkenes possessing different aromatic scaffolds, including naphthalene (16), pyrene (17), dibenzofuran (18), dibenzothiophene (19), carbazole (20), thianthrene (21), quinoline (22), and thiophene (23) moieties, were all compatible with the reaction to deliver the corresponding products in good yields. With respect to the C–Cl precursor, the protocol could also activate the C–Cl bond of another commonly used industrial feedstock dichloroethane (DCE) to give the corresponding products 24–30 in moderate to good yields. Additionally, the C–Cl bonds of chloroform and trichloroethane could also be activated to furnish 31 and 32 in 75% and 51% yields, respectively. To illustrate the practicability of this protocol, a gram-scale reaction was conducted, and the desired product 1 was obtained in 76% yield. Moreover, the mild conditions and excellent functional group tolerance inspired us to explore its application to late-stage modification of complex molecules. To our delight, the trifluoromethyl alkenes derived from indomethacin, d-phenylalanine, ciprofibrate, and estrone successfully afforded the corresponding products 33–36 in satisfactory yields. Furthermore, inspired by the case of 15, we envisioned that the photocatalytic protocol was probably able to enable a hydroalkylation reaction. Indeed, when we employed this protocol to activate the C–Cl bonds of DCE, ClCH_2_CN, chloroform, and various ClCF_2_R, the corresponding carbon-centered radicals could smoothly react with a range of alkenes including various activated and unactivated ones to give the corresponding hydroalkylation products 37–48 in moderate yields.

**Table 2:** Substrate scope of the C–Cl activation reaction^a^^,^^b^

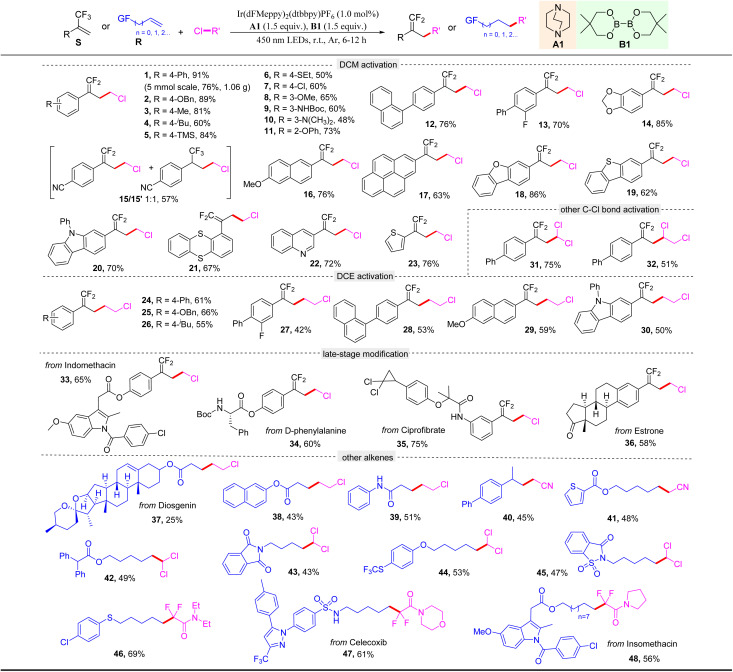

a Standard conditions: S or R (0.2 mmol), photocatalyst (1.0 mol%), A1 (0.3 mmol), B1 (0.3 mmol), RCl (2.0 mL), r.t., Ar, and 450 nm LED irradiation for 6–12 h.

b Isolated yields.

Encouraged by the above results, we wondered whether this protocol would be able to activate the C–Br bond of organic bromides. Therefore, a broad set of organic bromides reacting with α-trifluoromethyl arylalkene S-2 were examined. It should be noted that herein 5.0 equivalent of organic bromides was used, and acetonitrile was selected as a solvent. As shown in Table 3, primary alkyl bromides bearing various functional groups, including different chain length alkyl (49–52), ether (53), *tert*-butyl carbamate (54), free hydroxyl (55–57), furan (58), ester (59), and vinyl (60, 61) groups, were all well tolerated to give the corresponding alkylation products in moderate to good yields. Notably, when 1-bromo-3-chloropropane was used in this protocol, the C–Br bond was selectively activated to access 62 in 60% yield, and the C–Cl bond remained intact. Secondary alkyl bromides such as cyclobutyl (63), cyclopentyl (64), cyclohexyl (65), piperidyl (66), and pyranyl (67) bromides were all suitable substrates. Furthermore, tertiary alkyl bromides were also found to be amenable to deliver the products 68–70 in good yields. Moreover, the C(sp^2^)–Br bond of aromatic bromides could also be activated to realize this transformation with high efficiency (71–73), demonstrating the excellent ability of ligated boryl radicals from this protocol for C–Br bond activation.

**Table 3:** Substrate scope of the C–Br activation reaction^a^^,^^b^

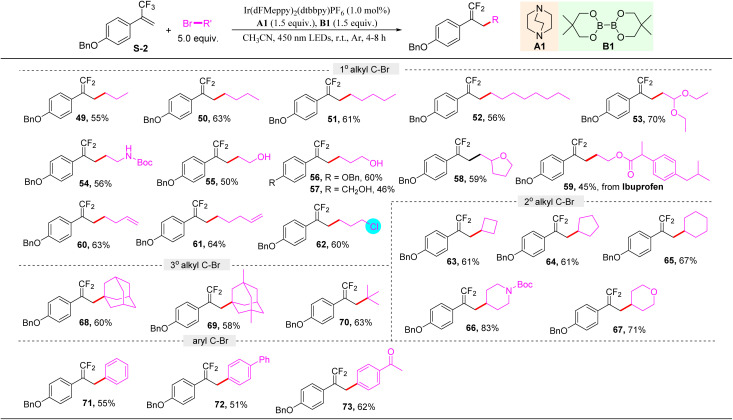

a Standard conditions: S-2 (0.2 mmol), photocatalyst (1.0 mol%), A1 (0.3 mmol), B1 (0.3 mmol), RBr (5.0 equiv.), CH_3_CN (3.0 mL), r.t., Ar, and 450 nm LED irradiation for 4–8 h.

b Isolated yields.

**Scheme 2:**
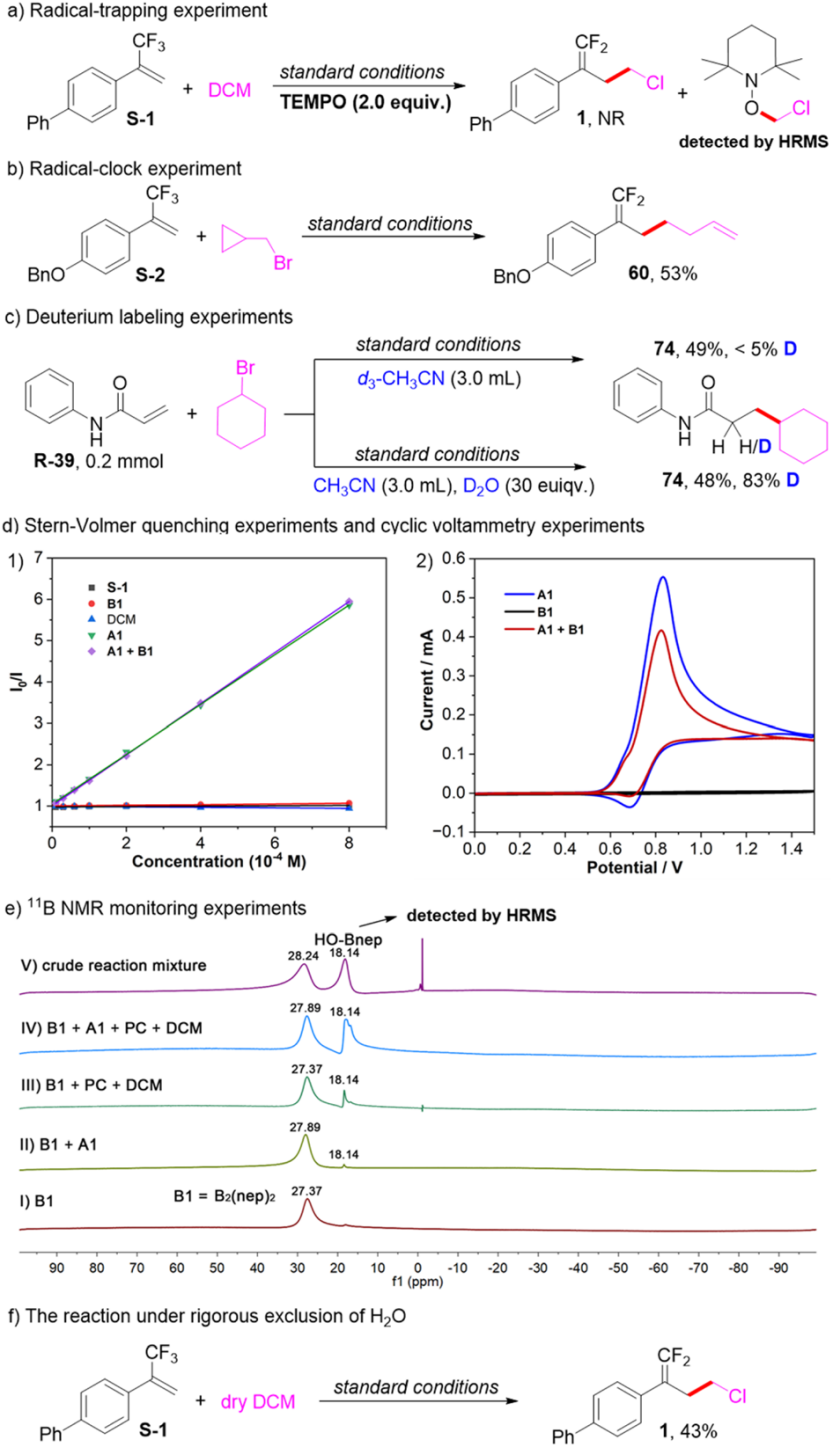
(a) Radical-trapping experiment. (b) Radical-clock experiment. (c) Deuterium labeling experiments. (d) Luminescence quenching experiments and cyclic voltammetry experiments. (e) ^11^B NMR monitoring experiments. (f) The reaction under rigorous exclusion of H_2_O.

A series of experimental studies were carried out to gain insights into the reaction mechanism (Scheme 2). When a radical scavenger 2,2,6,6-tetramethyl-1-piperidinyloxy (TEMPO) was added into the model reaction under standard conditions, formation of the desired product 1 was completely inhibited, and the adduct TEMPO-CH_2_Cl was detected by high resolution mass spectrometry (HRMS) (Scheme 2a). In addition, the radical-clock experiment with cyclopropylmethyl bromide under standard conditions afforded the ring-opening product 60 in 53% yield (Scheme 2b). These results suggested that the reaction proceeded *via* a radical pathway. For the hydroalkylation reaction, to confirm the hydrogen source of the products, the reaction of R-39 with cyclohexyl bromide under standard conditions using *d*_3_-CH_3_CN was conducted, just a small amount of deuterium atom was incorporated into the product 74. Moreover, addition of 30 equivalent of deuterium oxide to the reaction resulted in 83% deuterium incorporation, suggesting that the hydrogen source of the product is the H_2_O in the solvent (Scheme 2c). Furthermore, Stern–Volmer quenching experiments were conducted as shown in Scheme 2d(1). The results revealed that the excited-state photocatalyst could not be quenched by trifluoromethyl alkene S-1, B1, or dichloromethane, while it could be effectively quenched by the assembly of A1 and B1 or A1 alone, and their quenching efficiencies are comparable. Additionally, cyclic voltammetry experiments showed that the oxidation potential of the assembly of A1 and B1 is almost the same as that of A1 at about +0.83 V *vs.* SCE (Scheme 2d(2)). These results indicated that the single electron transfer between the photocatalyst and the assembly of A1 and B1 is feasible, and it is actually initiated by the photocatalytic oxidation of A1. Moreover, a series of ^11^B NMR monitoring experiments were carried out. As shown in Scheme 2e, in spectrum V of the crude mixture after the reaction was complete, a new species with a chemical shift of 18.14 ppm was observed, which was assigned to the signal of HO-Bnep.^58^ And the generation of HO-Bnep could be further detected by HRMS. In spectrum I and II, a chemical shift difference of 0.52 ppm was observed, revealing a weak coordination between A1 and B1. Additionally, in spectrum III and IV, the signal of HO-Bnep was still observed even without A1. The above results indicated that besides the photocatalyst and A1, H_2_O in the solvent probably participated in accelerating the B–B bond cleavage of B1 to generate the reactive ligated boryl radical. Therefore, a control reaction under rigorous exclusion of H_2_O was conducted, the yield was sharply decreased to 43%, further confirming that H_2_O could promote this transformation (Scheme 2f). Note that an amino radical transfer (ART) mechanism was reported to enable the generation of ligated boryl radicals from alkyl boronic esters,^59^ while primary or secondary alkylamines are typically required in those cases. In this work, tertiary amines (A1–A3) or pyridines (A4–A6) were used. Thus, a mechanism through the single electron transfer between the photocatalyst and the assembly of A1 and B1 rather than the ART mechanism is more reasonable in this protocol.

**Scheme 3:**
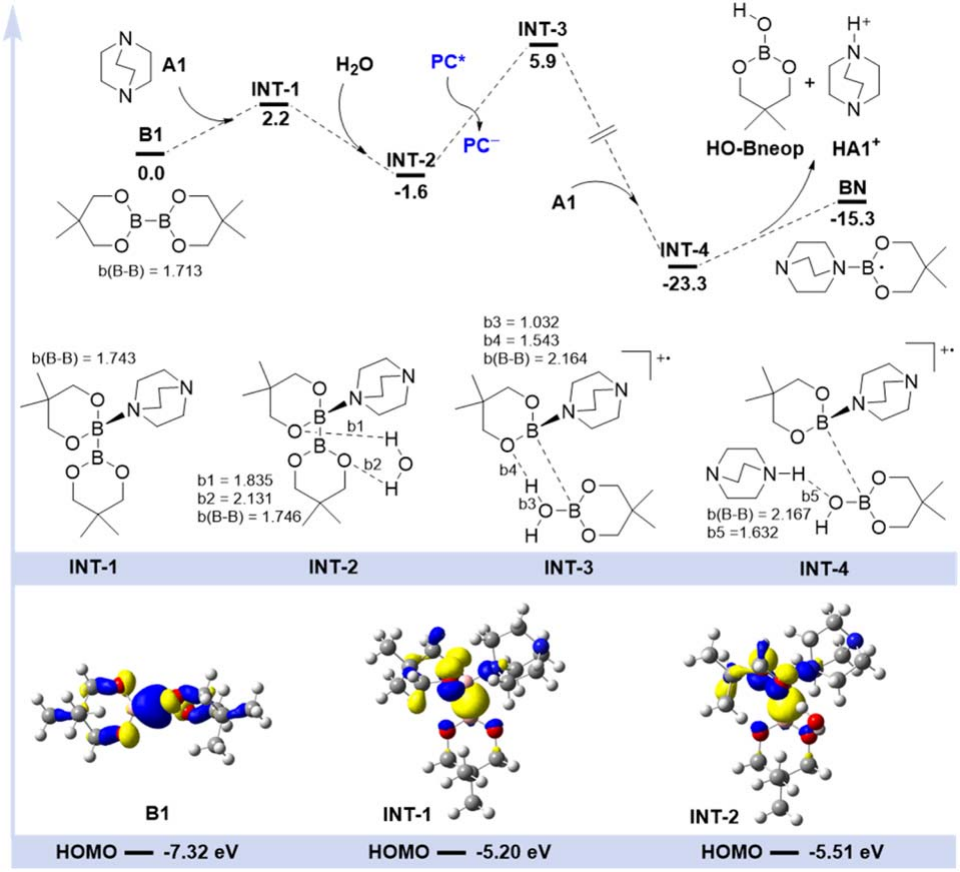
DFT calculated potential energy surface and frontier molecular orbital analysis for the ligated boryl radical formation process in kcal mol^−1^.

Based on the above experimental results, computational studies were carried out for further understanding of the generation of ligated boryl radicals (Scheme 3). First, the weak coordination of tertiary amine A1 to one boron atom of B1 produced the intermediate INT-1, with a slightly activated B–B bond elongated from 1.713 Å to 1.743 Å, which is in accordance with the observation in the ^11^B NMR spectrum I and II (Scheme 2e). Then, INT-1 could be stabilized by one molecule of water to form a more stable intermediate INT-2, with two O–H⋯O hydrogen bonding interactions (*b*1 = 1.835 Å, *b*2 = 2.131 Å) observed. Frontier molecular orbital analysis indicated that the HOMO orbital of B1 could be activated with the coordination of amine and water, which was elevated from −7.32 eV (B1) to −5.20 eV (INT-1) and −5.51 eV (INT-2), respectively. Additionally, orbital diagrams showed that the HOMO orbital of B1 was mainly assigned to the two boron atoms. In comparison, the nitrogen atom in the tertiary amine made some contribution to the HOMO orbital of INT-1 and INT-2, indicating that the tertiary amine played a role in the activation of the B–B bond. The cleavage of the B–B bond happened with the aid of the single electron transfer (SET) process by the photocatalyst, producing a radical cation species INT-3 with an energy difference of 7.5 kcal mol^−1^ in terms of Gibbs free energy. The B–B bond in INT-2 was calculated to be 1.746 Å, and was totally cleaved to 2.164 Å in INT-3. Relaxed potential energy scan showed that the B–B bond cleavage process was barrierless (see more details in the ESI, Fig. S3a†). Then, the deprotonation process released the energy of 29.2 kcal mol^−1^ with the aid of tertiary amine A1 to form INT-4. Structural analysis showed that the bond length of the O–H bond increased from 1.032 Å in INT-3 to 1.632 Å in INT-4, indicating that the proton was transferred from HO-Bneop to the tertiary amine, which was also confirmed to be barrierless (see more details in the ESI, Fig. S3b†). Finally, INT-4 may have readily dissociated into three parts, HO-Bneop, protonated tertiary amine HA1^+^ and tertiary amine-ligated boryl radical BN. Though experimental and computational studies provide us a rational pathway, the exact nature of the generation of ligated boryl radicals is still unclear due to the complexity of this photocatalytic system, more detailed investigations need to be conducted in the future.

**Scheme 4:**
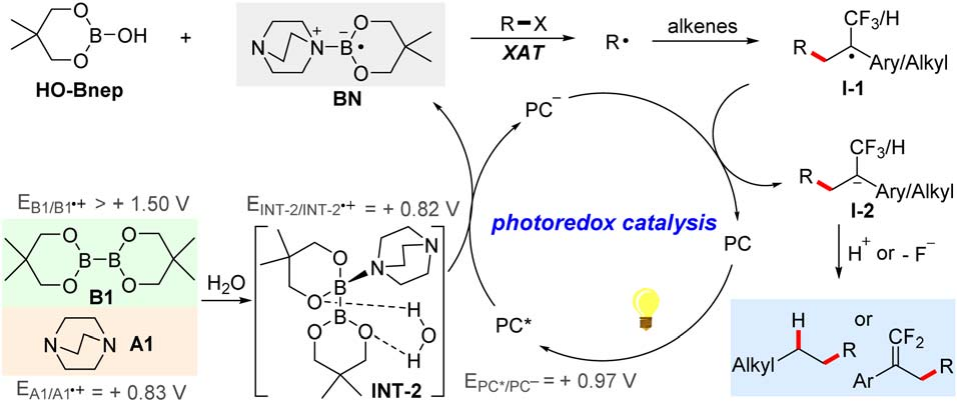
Plausible mechanism.

Based on the above experimental and computational results, a plausible mechanism is proposed as shown in Scheme 4. With the aid of tertiary amine A1 and H_2_O, the activation of the B–B bond of diboron ester B1 through single-electron transfer by the excited-state photocatalyst PC*, which is a feasible process supported by luminescence quenching experiments and cyclic voltammetry experiments, generates the reduced photocatalyst PC^−^ and tertiary amine-ligated boryl radical BN. Then, BN undergoes a facile halogen atom transfer with organohalides to result in the generation of the corresponding alkyl radical R˙, which can be trapped by alkenes *via* intermolecular radical addition to furnish a new alkyl radical I-1. Subsequently, single-electron transfer from PC^−^ to I-1 regenerates the photocatalytic cycle and produces a carbanion intermediate I-2. In the case of the trifluoromethyl alkene substrate, the following defluorination of I-2 leads to the alkylation product *gem*-difluoroalkenes. In the case of the unactivated alkenes, subsequent protonation of I-2 results in the final hydroalkylation products. It should be noted that in previous reports,^60–63^ ligated boryl radicals were able to act as a highly reducing agent to activate aryl or alkyl halides *via* single electron transfer. In this work, considering the fact that the reduction potential of dichloromethane is too high, halogen atom transfer is believed to be the actual process to activate the C–X bond of organohalides. However, the activation *via* the SET mechanism could not be completely excluded.

## Conclusions

In summary, a new photocatalytic platform has been established to generate ligated boryl radicals. By flexible modular assembly of readily available amines and diboron esters, we can quickly access a library of various reactive ligated boryl radicals for reaction screening. Therefore, by employing the assembly of DABCO A1 and B_2_Nep_2_B1, the C–Cl/Br bond of organohalides could be effectively activated *via* a halogen atom transfer process, thus enabling the cross-coupling of organohalides with alkenes including activated and unactivated ones for C(sp^3^)–C(sp^3^) bond formation under photocatalysis. Experimental and computational studies rationalize that the ligated boryl radical is generated through the activation of the B–B bond of diboron ester *via* photoinduced single electron transfer with the aid of amine and H_2_O. Such a protocol features mild conditions, good functional group tolerance and broad substrate scope. Scale-up synthesis and late-stage modification of structurally complex molecules demonstrate the practicability. Further application of this photocatalytic platform for ligated boryl radical-mediated organic transformations is ongoing in our laboratory.

## Data availability

The data supporting this article have been included as part of the ESI.†

## Author contributions

The manuscript was written through contributions of all authors. All authors have given approval to the final version of the manuscript. R. B. L., T. T. M., and X. R. L. designed and performed the experiments. J. B. H. and S. F. N. performed the density functional theory calculations. S. L. and Q. X. T. participated in the data discussion. J. J. Z. directed the project and wrote the manuscript.

## Conflicts of interest

There are no conflicts to declare.

## Supplementary Material

SC-016-D5SC00190K-s001

## References

[cit1] Juliá F., Constantin T., Leonori D. (2022). Chem. Rev..

[cit2] Ji C.-L., Zhai X., Fang Q.-Y., Zhu C., Han J., Xie J. (2023). Chem. Soc. Rev..

[cit3] Kuivila H. G., Menapace L. W. (1963). J. Org. Chem..

[cit4] Crespi S., Fagnoni M. (2020). Chem. Rev..

[cit5] Le Grognec E., Chrétien J.-M., Zammattio F., Quintard J.-P. (2015). Chem. Rev..

[cit6] Zeitler K. (2009). Angew. Chem., Int. Ed..

[cit7] Yoon T. P., Ischay M. A., Du J. (2010). Nat. Chem..

[cit8] Narayanam J. M., Stephenson C. R. (2011). Chem. Soc. Rev..

[cit9] Shi L., Xia W. (2012). Chem. Soc. Rev..

[cit10] Prier C. K., Rankic D. A., MacMillan D. W. C. (2013). Chem. Rev..

[cit11] Nicewicz D. A., Nguyen T. M. (2014). ACS Catal..

[cit12] Fabry D. C., Rueping M. (2016). Acc. Chem. Res..

[cit13] Ghosh I., Marzo L., Das A., Shaikh R., König B. (2016). Acc. Chem. Res..

[cit14] Liu Q., Wu L.-Z. (2017). Natl. Sci. Rev..

[cit15] Chen Y., Lu L.-Q., Yu D.-G., Zhu C.-J., Xiao W.-J. (2018). Sci. China:Chem..

[cit16] Yu X. Y., Zhao Q. Q., Chen J., Xiao W. J., Chen J. R. (2020). Acc. Chem. Res..

[cit17] Yu X. Y., Chen J. R., Xiao W. J. (2021). Chem. Rev..

[cit18] Zhang P., Le C. C., MacMillan D. W. C. (2016). J. Am. Chem. Soc..

[cit19] ElMarrouni A., Ritts C. B., Balsells J. (2018). Chem. Sci..

[cit20] Yu X., Lübbesmeyer M., Studer A. (2021). Angew. Chem., Int. Ed..

[cit21] Mistry S., Kumar R., Lister A., Gaunt M. J. (2022). Chem. Sci..

[cit22] Luridiana A., Mazzarella D., Capaldo L., Rincón J. A., García-Losada P., Mateos C., Frederick M. O., Nuño M., Jan Buma W., Noël T. (2022). ACS Catal..

[cit23] Lovett G. H., Chen S., Xue X.-S., Houk K. N., MacMillan D. W. C. (2019). J. Am. Chem. Soc..

[cit24] Constantin T., Zanini M., Regni A., Sheikh N. S., Juliá F., Leonori D. (2020). Science.

[cit25] Zhou X.-S., Yan D.-M., Chen J.-R. (2020). Chem.

[cit26] Kostromitin V. S., Sorokin A. O., Levin V. V., Dilman A. D. (2023). Chem. Sci..

[cit27] Yue F., Dong J., Liu Y., Wang Q. (2021). Org. Lett..

[cit28] Taniguchi T. (2021). Chem. Soc. Rev..

[cit29] Capaldo L., Noël T., Ravelli D. (2022). Chem Catal..

[cit30] Peng T.-Y., Zhang F.-L., Wang Y.-F. (2023). Acc. Chem. Res..

[cit31] Baban J. A., Roberts B. P. (1983). J. Chem. Soc., Chem. Commun..

[cit32] Ueng S.-H., Solovyev A., Yuan X., Geib S. J., Fensterbank L., Lacôte E., Malacria M., Newcomb M., Walton J. C., Curran D. P. (2009). J. Am. Chem. Soc..

[cit33] Curran D. P., Solovyev A., Makhlouf Brahmi M., Fensterbank L., Malacria M., Lacôte E. (2011). Angew. Chem., Int. Ed..

[cit34] Pan X., Lacôte E., Lalevée J., Curran D. P. (2012). J. Am. Chem. Soc..

[cit35] Kawamoto T., Geib S. J., Curran D. P. (2015). J. Am. Chem. Soc..

[cit36] Duret G., Quinlan R., Bisseret P., Blanchard N. (2015). Chem. Sci..

[cit37] Wang G., Zhang H., Zhao J., Li W., Cao J., Zhu C., Li S. (2016). Angew. Chem., Int. Ed..

[cit38] Fawcett A., Pradeilles J., Wang Y., Mutsuga T., Myers E. L., Aggarwal V. K. (2017). Science.

[cit39] Supranovich V. I., Levin V. V., Struchkova M. I., Korlyukov A. A., Dilman A. D. (2017). Org. Lett..

[cit40] Zhang L., Jiao L. (2017). J. Am. Chem. Soc..

[cit41] Yu Y.-J., Zhang F.-L., Peng T.-Y., Wang C.-L., Cheng J., Chen C., Houk K. N., Wang Y.-F. (2021). Science.

[cit42] Ding Z., Liu Z., Wang Z., Yu T., Xu M., Wen J., Yang K., Zhang H., Xu L., Li P. (2022). J. Am. Chem. Soc..

[cit43] Kuehn L., Zapf L., Werner L., Stang M., Würtemberger-Pietsch S., Krummenacher I., Braunschweig H., Lacôte E., Marder T. B., Radius U. (2022). Chem. Sci..

[cit44] Li W.-D., Wu Y., Li S.-J., Jiang Y.-Q., Li Y.-L., Lan Y., Xia J.-B. (2022). J. Am. Chem. Soc..

[cit45] Liu Y., Lin S., Li Y., Xue J.-H., Li Q., Wang H. (2023). ACS Catal..

[cit46] Wang C.-L., Wang J., Jin J.-K., Li B., Phang Y. L., Zhang F.-L., Ye T., Xia H.-M., Hui L.-W., Su J.-H., Fu Y., Wang Y.-F. (2023). Science.

[cit47] Fang C.-Z., Zhang B.-B., Tu Y.-L., Liu Q., Wang Z.-X., Chen X.-Y. (2024). J. Am. Chem. Soc..

[cit48] Koo J., Kim W., Jhun B. H., Park S., Song D., You Y., Lee H. G. (2024). J. Am. Chem. Soc..

[cit49] Zhang Z.-Q., Sang Y.-Q., Wang C.-Q., Dai P., Xue X.-S., Piper J. L., Peng Z.-H., Ma J.-A., Zhang F.-G., Wu J. (2022). J. Am. Chem. Soc..

[cit50] Zhang Z. H., Tilby M. J., Leonori D. (2024). Nat. Synth..

[cit51] Corpas J., Alonso M., Leonori D. (2024). Chem. Sci..

[cit52] Zhang Z.-Q., Wang C.-Q., Li L.-J., Piper J. L., Peng Z.-H., Ma J.-A., Zhang F.-G., Wu J. (2023). Chem. Sci..

[cit53] Wan T., Capaldo L., Ravelli D., Vitullo W., de Zwart F. J., de Bruin B., Noël T. (2023). J. Am. Chem. Soc..

[cit54] Wan T., Ciszewski L. W., Ravelli D., Capaldo L. (2024). Org. Lett..

[cit55] Li X., Zhong Y., Tan F., Fei Y., Zhao X., Xu J., Fan B. (2025). Org. Chem. Front..

[cit56] Pillitteri S., Walia R., Van der Eycken E. V., Sharma U. K. (2024). Chem. Sci..

[cit57] Ji C.-L., Han J., Li T., Zhao C.-G., Zhu C., Xie J. (2022). Nat. Catal..

[cit58] Candish L., Teders M., Glorius F. (2017). J. Am. Chem. Soc..

[cit59] Speckmeier E., Maier T. C. (2022). J. Am. Chem. Soc..

[cit60] Zhang L., Jiao L. (2018). Chem. Sci..

[cit61] Zhang L., Jiao L. (2019). J. Am. Chem. Soc..

[cit62] Bai L., Jiao L. (2023). Chem.

[cit63] Wang B., Peng P., Ma W., Liu Z., Huang C., Cao Y., Hu P., Qi X., Lu Q. (2021). J. Am. Chem. Soc..

