# Supplementary material for: Modular assembly of amines and diborons with photocatalysis enabled halogen atom transfer of organohalides for C(sp3)–C(sp3) bond formation
Source: Chem Sci. 2025 Jan 24;16(8):3580–7. doi: 10.1039/d5sc00190k (PMC11758988; doi:10.1039/d5sc00190k)
Supplement: SC-016-D5SC00190K-s001 [file SC-016-D5SC00190K-s001.pdf]

Supplementary Information (SI) for Chemical Science.
This journal is © The Royal Society of Chemistry 2025

Electronic Supplementary Information

Modular Assembly of Amines and Diborons with Photocatalysis Enabled Halogen Atom Transfer of Organohalides for $C(sp^3)$-$C(sp^3)$ Bond Formation

Rong-Bin Liang,[1,#] Ting-Ting Miao,[1,#] Xiang-Rui Li,[1] Jia-Bo Huang,[1] Shao-Fei Ni,*[,1]
Sanliang Li,[1] Qing-Xiao Tong,[1] and Jian-Ji Zhong*[,1,2]

[1]College of Chemistry and Chemical Engineering, and Key (Guangdong-Hong Kong Joint) Laboratory for Preparation and Application of Ordered Structural Materials of Guangdong Province, Shantou University, Shantou 515063, P. R. China

[2] Chemistry and Chemical Engineering Guangdong Laboratory, Shantou 515063, P. R. China

[#]These two authors contributed equally to this work

*E-mail: sfni@stu.edu.cn; jjzhong@stu.edu.cn

Table of Contents

Electronic Supplementary Information 1

Table of Contents 2

1. Experimental section 3

    1) General information 3

    2) Preparation of α-trifluoromethyl arylalkenes 4

    3) General procedure for the photochemical reactions 5

    4) Stern-Volmer Quenching Experiments 6

    5) Cyclic Voltammetry experiments 7

    6) Radical-trapping experiment 8

    7) Radical-clock experiment 9

    8) Deuterium labeling experiments 10

    9) ^{11}B NMR monitoring experiments 11

    10) Computational studies 12

    11) References 30

2. Characterization data of the products 32

3. NMR spectra for the products 59

1. Experimental section

1) General information

All chemicals, unless otherwise noted, were purchased from commercial sources and were used without further purification. Unless stated otherwise, all reactions were carried out under argon atmosphere. The substrates α-trifluoromethyl arylalkenes (**S**) were synthesized according to the literature methods with slight modification.[1-4] Irradiation with visible light was performed using blue LEDs (λ = 450 ± 10 nm) illumination instruments (The instruments were designed by ourselves and the actual output power density of the LEDs at 0.5 cm distance is 33.70 mW/cm^2 detected by CEL-NP2000-10 (Beijing Ceau Light Co. Ltd., China) light power meter). For irradiation, the material of the reaction vessel is common glass; the distance from the light source is about 0.5 cm.

The nuclear magnetic resonance spectra were recorded on the Bruker Ascend™ 400 MHz NMR spectrometer with tetramethylsilane (TMS) as an internal standard. High resolution mass spectra were recorded using a Q Exactive mass spectrometer (Thermo Fisher Scientific, USA).

2) Preparation of *α*-trifluoromethyl arylalkenes

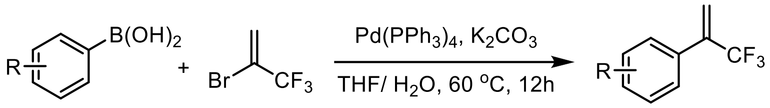

Arylboronic acid (10.0 mmol), $Pd(PPh_3)_4$ (0.3 mmol, 3 mol%), K_2CO_3 (20.0 mmol, 2.0 equiv.) were dissolved in co-solvents (THF/ H_2O = 2:3, (v/v), 50.0 mL) in a two-neck flask under argon atmosphere. Then, 2-bromo-3,3,3-trifluoroprop-1-ene (20.0 mmol, 2.1 mL) was added dropwise into the mixture. The mixture was heated to 60 °C in an oil bath for at least 12 h. Then the mixed solution was extracted with ethyl acetate (3 × 15.0 mL). The organic layer was washed with brine (20.0 mL), dried over Na_2SO_4, and then concentrated under reduced pressure. The resulting residue was purified by silica gel column chromatography (petroleum ether/ethyl acetate = 200/1) to afford the desired products.

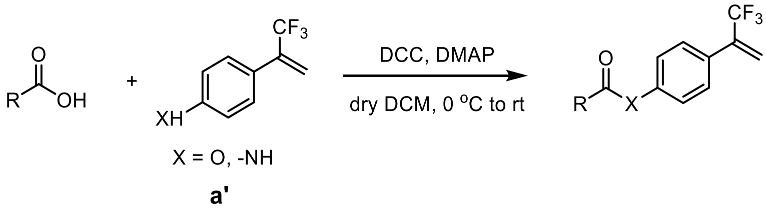

To a solution of carboxylic acid (5.0 mmol), 4-dimethylaminopyridine (DMAP) (20 mol%, 1.0 mmol, 122 mg) and **a’** (5.5 mmol, 1.1 equiv.) in dry DCM (15 mL) at 0 °C, followed by adding *N*,*N*’-dicyclohexylcarbodiimide (DCC) (5.5 mmol, 1.14 g, 1.1 equiv.). After stirring for 5 min at 0 °C, the reaction mixture was stirred at room temperature for 12h. Upon completion, the resulting mixture was filtered through a pad of Celite. The filtrate was concentrated under reduced pressure. The resulting residue was purified by silica gel column chromatography to afford the desired products.

3) General procedure for the photochemical reactions

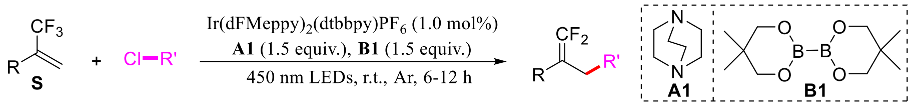

S (1.0 equiv., 0.2 mmol), Ir(dFMeppy)$_2$(dtbbpy)PF$_6$ (2.0 mg, 1.0 mol%), **A1** (1.5 equiv., 0.3 mmol), **B1** (1.5 equiv., 0.3 mmol) were dissolved in **Cl-R'** (2.0 mL) in a 10.0 mL tube equipped with magnetic stirring bar, then the reaction tube was irradiated by blue LEDs (λ = 450 ± 10 nm) at room temperature for 6-12 h. After reaction, the solvent was removed by rotary evaporation and purified by column-chromatography on silica gel using hexane/ ethyl acetate as the eluent to afford the desired products.

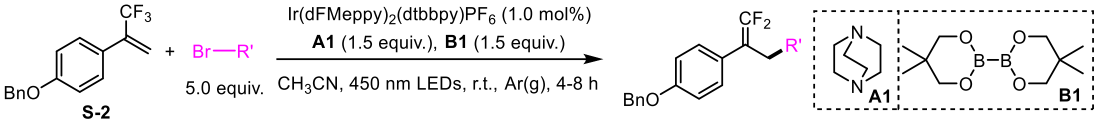

S-2 (1.0 equiv., 0.2 mmol), **Br-R'** (5.0 equiv., 1.0 mmol), Ir(dFMeppy)$_2$(dtbbpy)PF$_6$ (2.0 mg, 1.0 mol%), **A1** (1.5 equiv., 0.3 mmol), **B1** (1.5 equiv., 0.3 mmol) were dissolved in 3.0 mL CH_3CN in a 10.0 mL flask equipped with magnetic stirring bar, then the reaction tube was irradiated by blue LEDs (λ = 450 ± 10 nm) at room temperature for 4-8 h. After reaction, the solvent was removed by rotary evaporation and purified by column-chromatography on silica gel using hexane/ ethyl acetate as the eluent to afford the desired products.

4) Stern-Volmer Quenching Experiments

All Stern-Volmer quenching experiments were carried out on Fluorescence spectrometer (PTI QM-TM) under an argon atmosphere. Stern-Volmer quenching experiments were measured with excitation at 400 nm. The acetonitrile solution of 1.0×10^{-5} M Ir(dFMeppy)$_2$(dtbbpy)PF$_6$ and 1.0×10^{-3} M **DCM**, **S-1**, **A1**, **B1** or (**A1+B1**), respectively, were prepared. The experiments were conducted in 1.25 cm x 1.25 cm x 4.5 cm quartz cuvette at room temperature. Appropriate volume (the whole solution volume change < 5%) of quenchers were respectively injected to the acetonitrile solution (3.0 mL) of 1.0×10^{-5} M Ir(dFMeppy)$_2$(dtbbpy)PF$_6$ in the quartz cuvette by microsyringe.

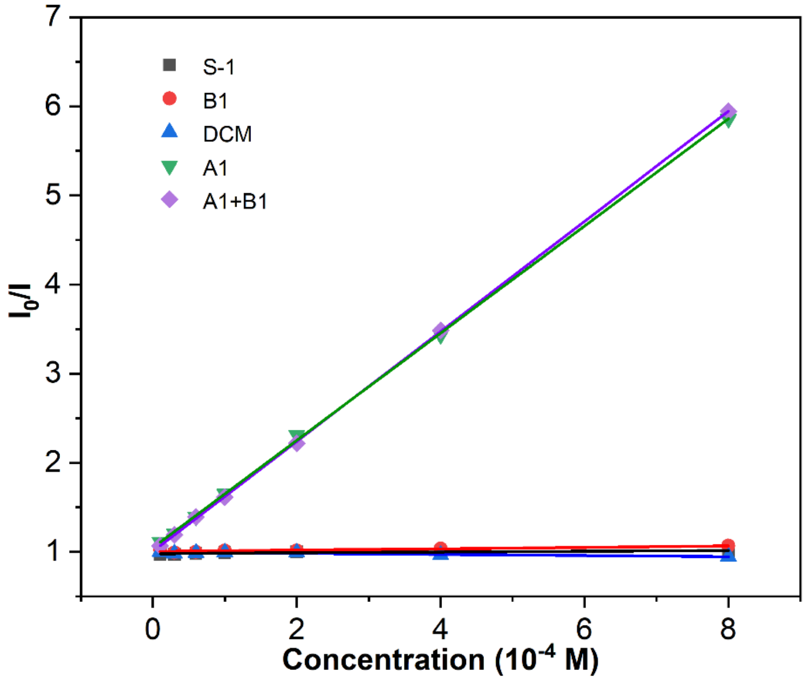

Figure S1. Ir(dFMeppy)$_2$(dtbbpy)PF$_6$ emission quenching by **S-1**, **A1**, **B1**, **(A1+B1)** and **DCM**

5) Cyclic Voltammetry experiments

All Cyclic Voltammetry studies were performed using a Shanghai Chenhua CHI-760E workstation under an argon atmosphere. Polish the glass carbon electrode with aluminum oxide. A glassy-carbon (GC) electrode (5 mm-diameter, disk-electrode) was used as the working electrode, Pt wire was used as the counter electrode and an Ag/AgCl electrode was used as the reference electrode.

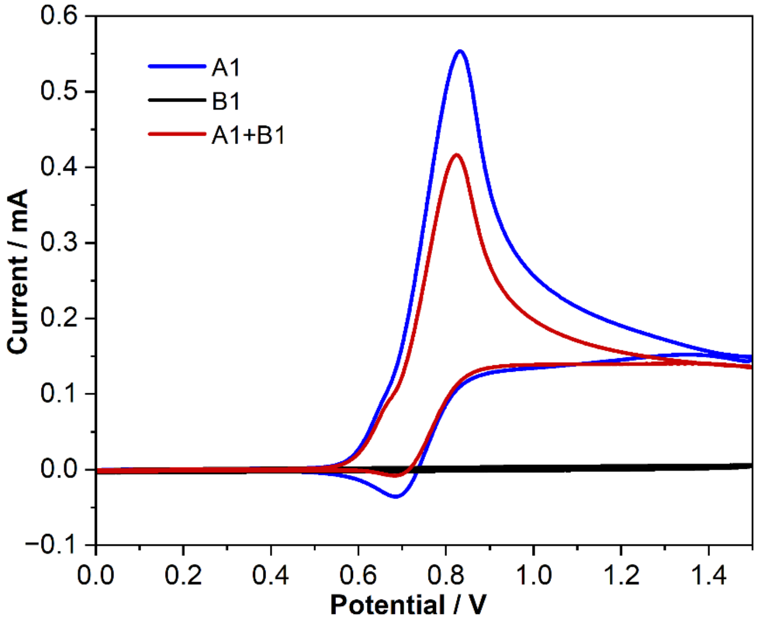

Figure S2. Cyclic voltammograms study for **A1**, **B1** and (**A1**+**B1)**

6) Radical-trapping experiment

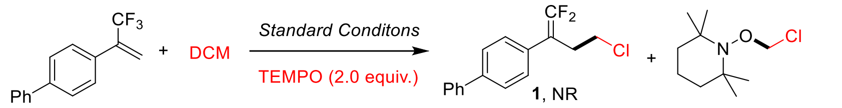

detected by HRMS

HRMS (ES^+) Calcd [M+H]$^+$: 206.1306

Found : 206.1305

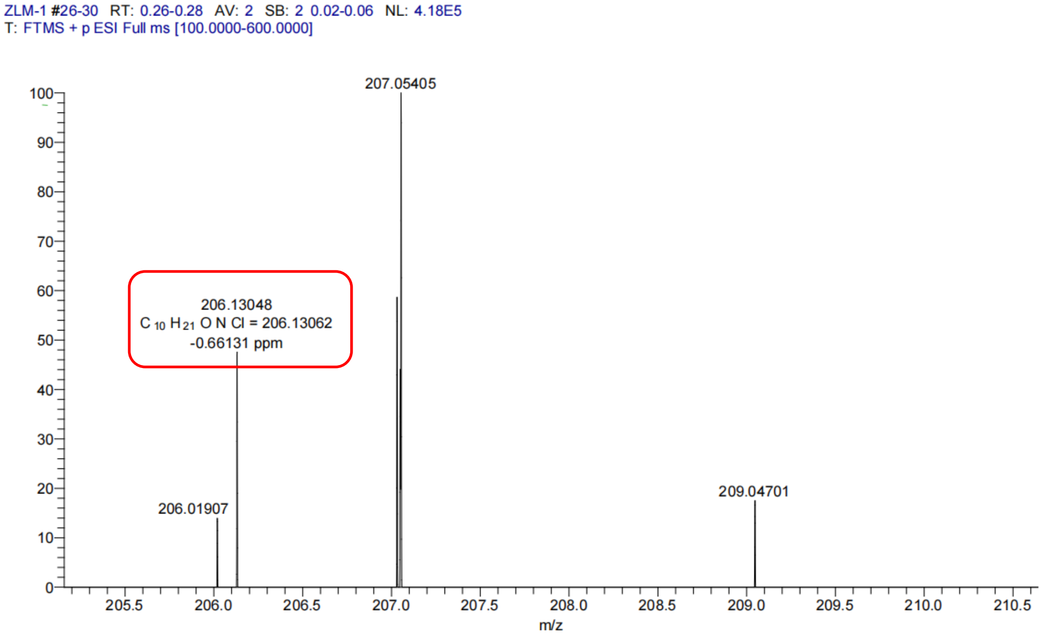

7) Radical-clock experiment

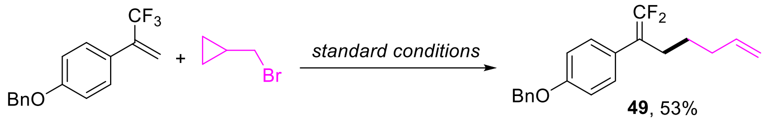

49, 53%

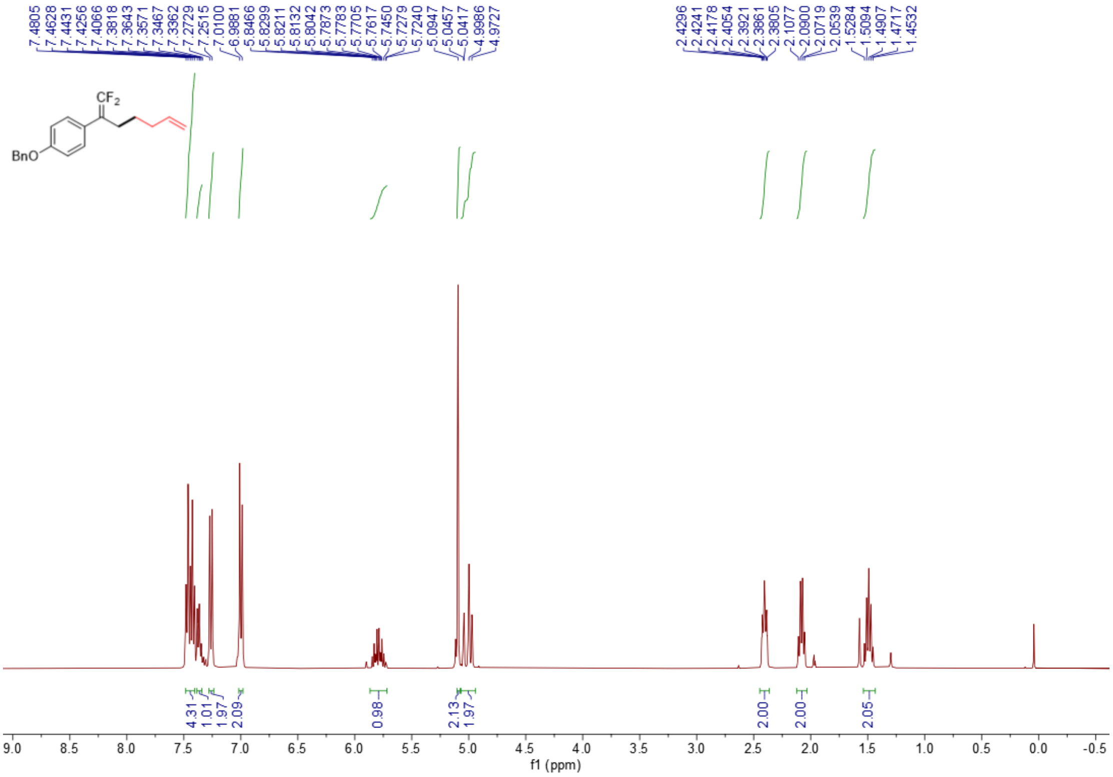

8) Deuterium labeling experiments

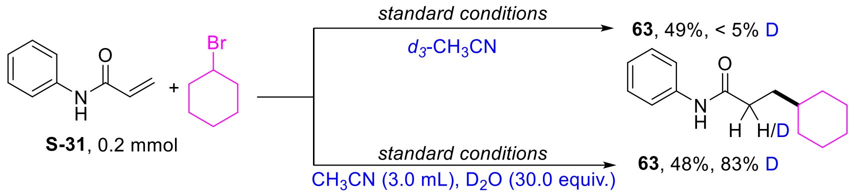

With d_3-CH_3CN

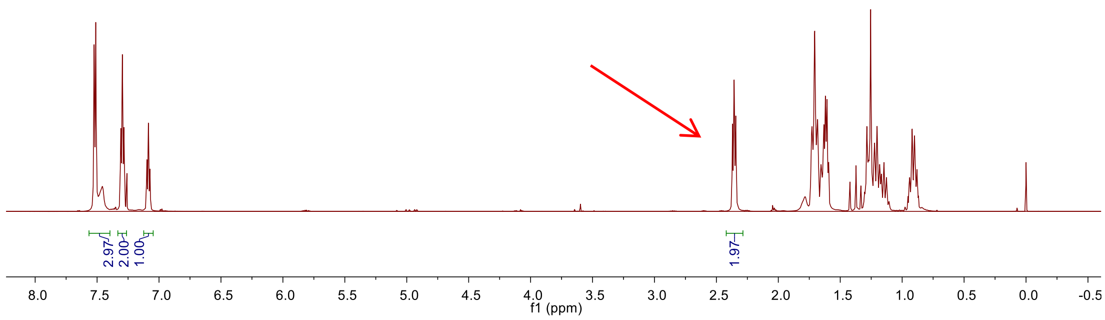

With D_2O (30.0 equiv.)

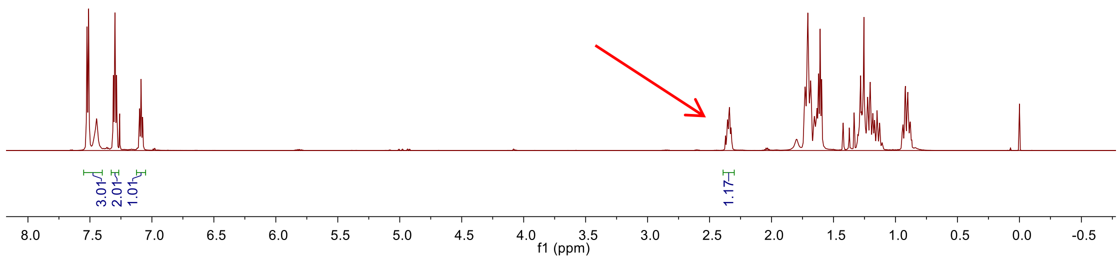

9) ^{11}B NMR monitoring experiments

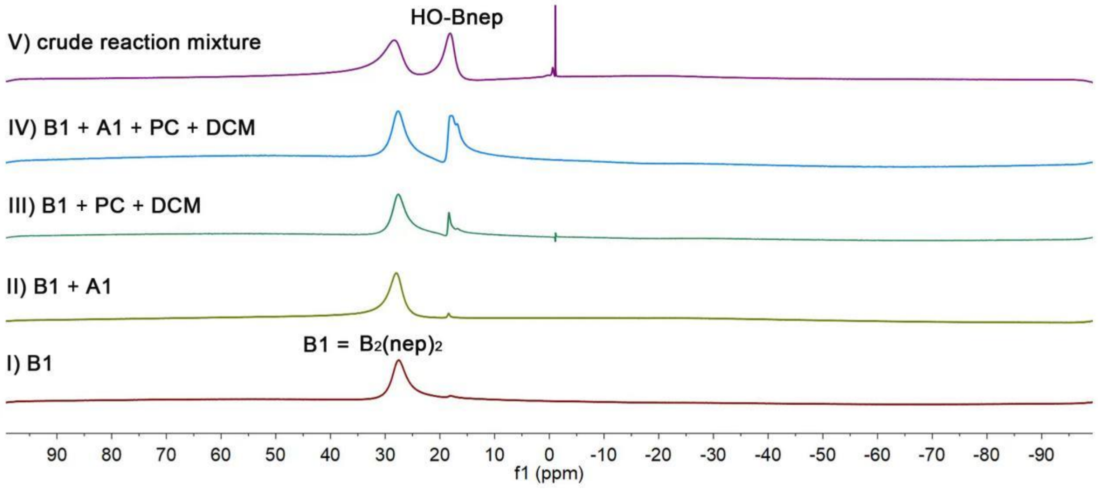

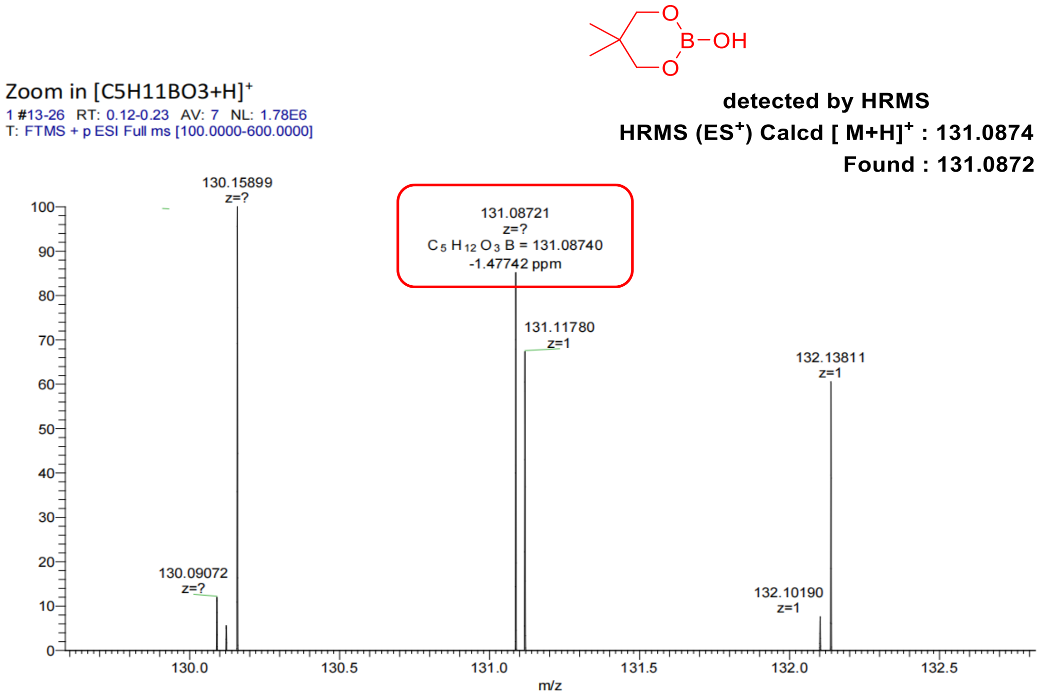

10) Computational studies

All calculations were performed using Gaussian 16, Revision A.03 package.[5] All of the reactants, intermediates, transition states, products were optimized by the DFT with the B3LYP-D3 functional.[6-11] For geometry optimizations and frequency calculations, BS-I basis set system was employed. In BS-I, we employed 6-31G(d) basis sets for H, B, C, N and O. All the stationary structures were characterized with no imaginary frequency and the transition state structures (TSs) were characterized with a single imaginary frequency. Intrinsic reaction coordinate (IRC) calculations were performed on the TSs. The solvent effect of dichloromethane (DCM) was evaluated through the SMD method,[12] in which a better basis system BS-II was used. In BS-II, we employed 6-311++G(d,p) basis sets for H, B, C, N and O. All reported energies are free energies at a concentration of 1 M and a temperature of 298.15 K. A method developed by Whitesides *et al*.[13] was used to estimate the entropic contributions. This method was designed to better describe the suppression of the translational entropy upon moving from gas phase to a solvent for each species.[14,15]Three-dimensional structures and the orbitals' diagrams of molecules were visualized using GaussView 6.0.16.[16]

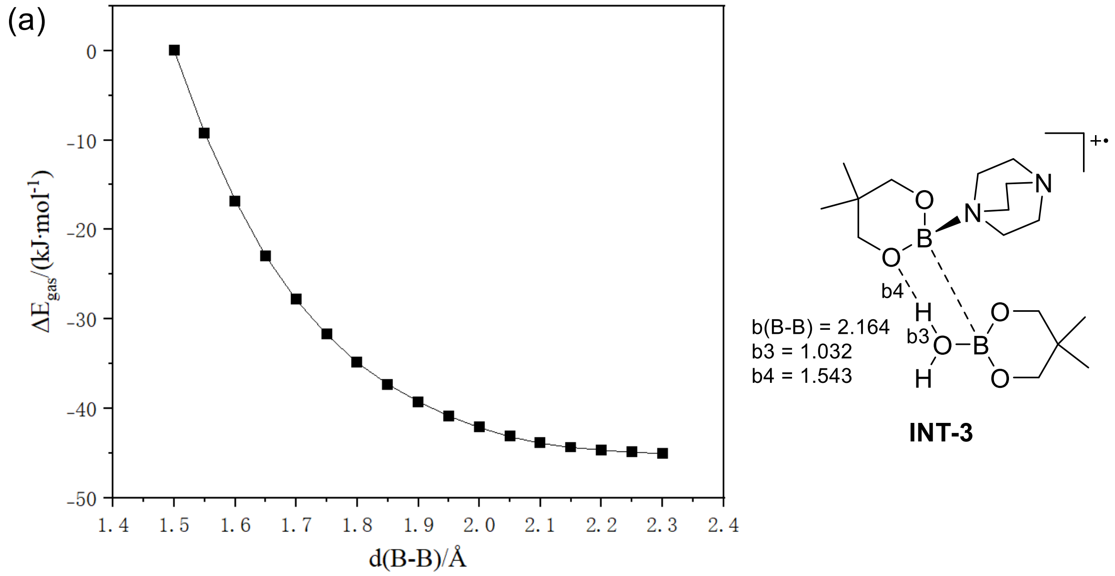

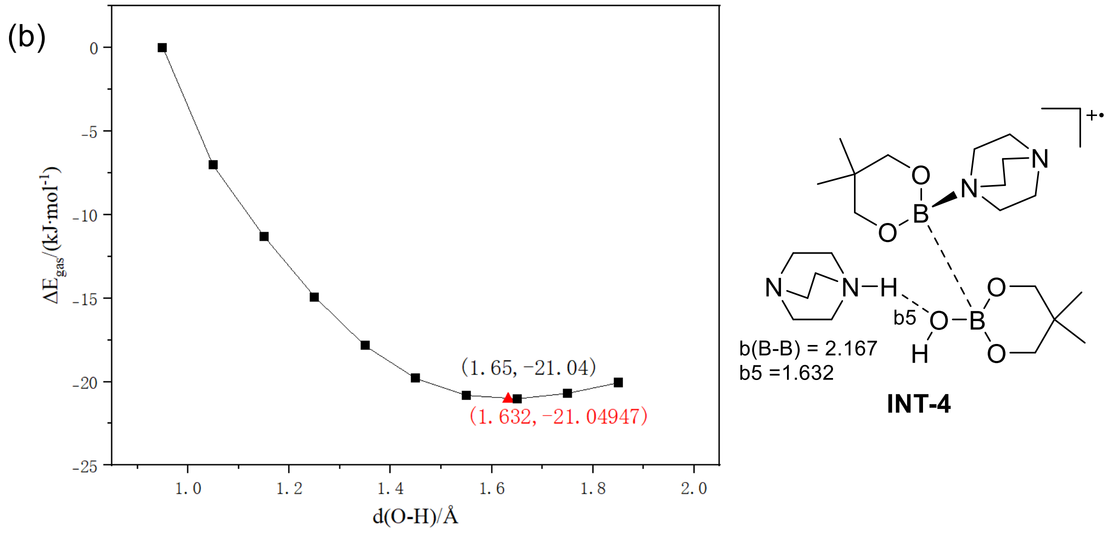

Figure S3. Graph sheets of potential energy scan.

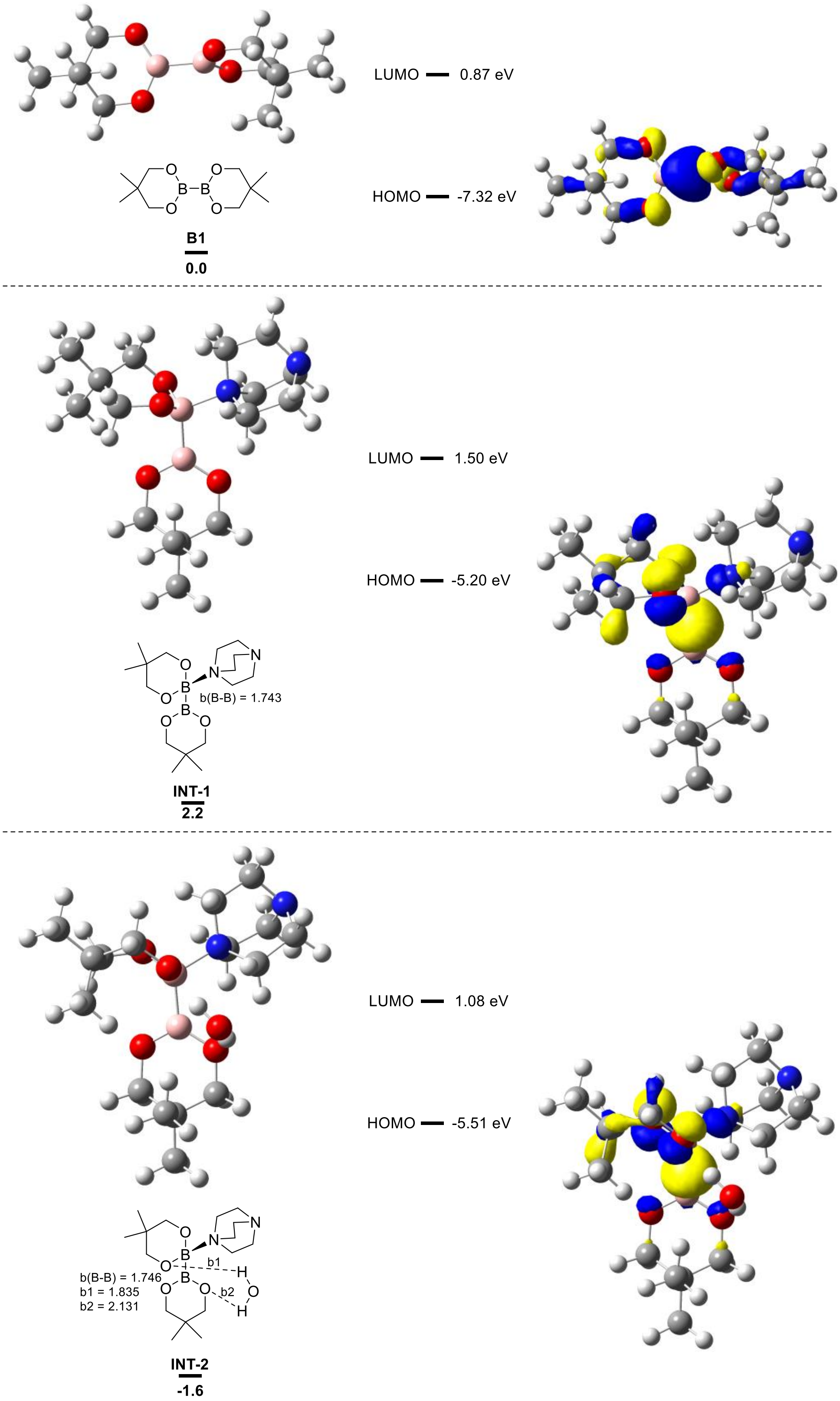

Figure S4. Three-dimensional structures and HOMO orbitals' energy values and diagrams of **B1**, **INT-1** and **INT-2**.

Cartesian coordinates of the optimized structures:

B1

E = -743.923192661 a.u.

0 1

C	-2.81404300	1.54211200	0.27547800
C	-2.71847400	3.89573300	-0.45675700
C	-3.30055800	2.51665500	-0.81032100
H	-3.28575800	1.78621800	1.23829800
H	-2.92149200	4.61598000	-1.25844100
H	-3.18714800	4.27725000	0.46181300
H	-3.09070600	0.51216300	0.02010400
C	3.01053800	3.48131900	1.51979200
C	3.20103500	1.99603000	-0.44052800
C	3.75416700	3.28639000	0.18774700
H	3.30363200	2.69661600	2.23216100
H	3.26741600	4.44960100	1.96587800
H	3.59652400	1.86178900	-1.45455200
H	3.50907900	1.12538700	0.15619900
B	-0.68658100	2.71664900	0.17689300
B	1.01709300	2.71385600	0.35713700
O	-1.30263900	3.86233900	-0.26471000
O	-1.39490600	1.57052600	0.44171400
O	1.77516400	1.99890300	-0.53802000
O	1.59022900	3.45017200	1.36445600
C	-4.83359100	2.58464600	-0.79342100
H	-5.20043200	3.29284400	-1.54615200
H	-5.26944200	1.60415900	-1.02003500
H	-5.21320600	2.90488300	0.18468000
C	-2.80628000	2.06439500	-2.19605200
H	-3.20984300	1.07572000	-2.44466400
H	-3.13305600	2.76870500	-2.97025200
H	-1.71504900	2.00088000	-2.23755300
C	3.50674000	4.48622500	-0.74409800
H	3.88848300	5.40941100	-0.29227300
H	4.01827100	4.34104300	-1.70299700
H	2.44173400	4.62896900	-0.94886900
C	5.25703700	3.12661400	0.45418100
H	5.80341100	2.96264700	-0.48247100
H	5.66732100	4.02854300	0.92414500
H	5.46185000	2.27728700	1.11753400

A1

E = -345.339880758 a.u.

0 1

C	-0.72408100	-0.50704600	0.00012900
C	-1.25631200	0.96507400	0.00065600
H	-1.06580100	-1.05899200	-0.88341500
H	-1.06569100	-1.05960900	0.88333000
H	-1.87279500	1.17122100	-0.88224900
H	-1.87150100	1.17103900	0.88450500
C	1.22780300	0.19859500	-1.19852600
H	2.32371600	0.16632200	-1.19767100
H	0.88494500	-0.35430700	-2.08102500
C	0.69521000	1.67061600	-1.19871400
H	1.51679700	2.39660200	-1.19849000
H	0.07760100	1.87583200	-2.08105100
C	0.69629600	1.67099400	1.19802600
H	1.51830200	2.39650200	1.19635100
H	0.07978200	1.87722400	2.08089300
C	1.22806200	0.19867400	1.19832300
H	2.32395700	0.16576500	1.19771800
H	0.88468900	-0.35379300	2.08089300
N	0.75015400	-0.51481000	-0.00002800
N	-0.12784700	1.91372500	-0.00001000

INT-1

E = -1089.28124878 a.u.

0 1

C	3.40097700	0.08882900	0.50905600
C	2.73807800	1.08369200	-1.63818900
C	3.36068600	1.41136200	-0.27258600
H	4.11898900	-0.60106700	0.04203300
H	3.72860300	0.26096400	1.54164000
H	2.57940500	2.00003900	-2.22040700
H	3.41498700	0.43516800	-2.21308700
B	1.17157600	-0.36439300	-0.41425700
O	1.47042200	0.43358700	-1.51456700
O	2.12465600	-0.54401400	0.56819200
C	2.50268300	2.44880300	0.47378000
H	2.94096000	2.67648900	1.45259900
H	2.44436100	3.38359500	-0.09681300
H	1.48286200	2.08921900	0.63978600
C	4.78599800	1.94451700	-0.46796000
H	4.77854900	2.87805100	-1.04368000
H	5.25858700	2.15449800	0.49908200
H	5.41713200	1.22343200	-1.00192300
C	-0.65391500	-0.83232900	2.30372400

C	-1.04020900	-3.02634500	1.14247300
C	-0.54858800	-2.38969200	2.45995000
H	-1.38355100	-0.43226100	3.01987800
H	0.32023700	-0.37474700	2.53554100
H	-0.85094000	-4.10834100	1.16483800
H	-2.13238200	-2.89070900	1.06896900
B	-0.39853300	-1.05308800	-0.09920800
O	-0.37178200	-2.49975900	0.01183100
O	-1.08532700	-0.42019400	1.01804100
C	0.91045300	-2.80451200	2.71524000
H	1.29554900	-2.33097900	3.62783100
H	0.98990900	-3.89212800	2.84318100
H	1.54605100	-2.50083000	1.88032100
C	-1.43816400	-2.87424300	3.61522800
H	-1.39661100	-3.96645700	3.71816300
H	-1.11252400	-2.43977700	4.56874800
H	-2.48637700	-2.58884800	3.45839700
C	-2.82066800	-1.32025900	-1.12949200
C	-3.81686200	-0.90047600	-2.24744100
H	-2.65610900	-2.39849600	-1.10312500
H	-3.12644500	-0.99467200	-0.13444900
H	-4.32295700	-1.77805000	-2.66490500
H	-4.59095500	-0.23230200	-1.85420700
C	-0.95284400	-1.25780900	-2.67940600
H	-0.01668700	-0.73970100	-2.88936300
H	-0.72877600	-2.30996300	-2.48928200
C	-2.01089600	-1.04734600	-3.80203800
H	-1.55429600	-0.57236100	-4.67736200
H	-2.43350200	-2.00297800	-4.13147200
C	-2.56349000	1.05370000	-2.79804100
H	-1.98236600	1.53338200	-3.59339100
H	-3.39352600	1.72406100	-2.54869100
C	-1.67586700	0.78410900	-1.54810400
H	-0.68630700	1.23202000	-1.63732700
H	-2.12704200	1.13196500	-0.61722000
N	-1.48113200	-0.69589100	-1.39374800
N	-3.11994600	-0.19842000	-3.33588100

H_2O

E = -76.4070324119 a.u.

0 1

O	-1.83875200	0.17257000	0.02385400
H	-0.87132900	0.22001500	0.02385400
H	-2.11696100	1.10034100	0.02385400

INT-2

E = -1165.71707929 a.u.

0 1

C	3.52152300	-0.49593100	-0.20090100
C	2.64997900	1.39902000	-1.50729500
C	3.60179300	1.03029600	-0.35970700
H	3.98221700	-0.98947200	-1.06885700
H	4.06696700	-0.81792800	0.69411000
H	2.56722500	2.48733600	-1.60782600
H	3.02886600	0.99926800	-2.45842700
B	1.10592500	-0.28177700	-0.58264000
O	1.32136400	0.89339200	-1.29968100
O	2.18127600	-0.97044900	-0.07210700
C	3.18103100	1.74560100	0.93786700
H	3.78787200	1.39723100	1.78190000
H	3.33066400	2.82745900	0.83905900
H	2.12671700	1.59486600	1.18526400
C	5.03726200	1.42604900	-0.73513600
H	5.11488900	2.50890300	-0.88908600
H	5.73512600	1.15476400	0.06600200
H	5.36910500	0.92841300	-1.65493100
C	-1.45266700	-1.08412000	2.06120000
C	-0.02239600	-2.92408900	1.14715300
C	-0.37408700	-2.11566500	2.44129000
H	-2.37703800	-1.61174500	1.77679800
H	-1.68739900	-0.44892800	2.92456300
H	1.06809100	-3.03613500	1.06621100
H	-0.45656800	-3.93045700	1.21265300
B	-0.47065800	-0.91072100	-0.17164900
O	-0.53869300	-2.34882800	-0.04193300
O	-1.01603100	-0.23121700	1.01326200
C	0.86555800	-1.38521400	2.98794400
H	0.61375800	-0.80875900	3.88723600
H	1.64923900	-2.10465300	3.25627200
H	1.28046500	-0.70170800	2.24495100
C	-0.93061600	-3.05877200	3.51911100
H	-0.19561000	-3.83094700	3.77810400
H	-1.17386700	-2.51176400	4.43889300
H	-1.84088300	-3.56628600	3.17529700
C	-2.95316700	-1.14760800	-1.00460300
C	-3.93490200	-1.04989000	-2.20518000
H	-2.80005600	-2.16905400	-0.65561200
H	-3.26746000	-0.53632000	-0.15684800

H	-4.17496100	-2.04400900	-2.59809800
H	-4.87665600	-0.58264800	-1.89731100
C	-1.18216900	-1.32801300	-2.67144500
H	-0.16216100	-1.00426800	-2.89451100
H	-1.15348800	-2.39311000	-2.43556600
C	-2.17631200	-0.97556200	-3.81668700
H	-1.69696500	-0.34024300	-4.56933200
H	-2.51800000	-1.88455600	-4.32358400
C	-2.90828900	1.03421000	-2.74365600
H	-2.58890600	1.67900700	-3.56958500
H	-3.77345000	1.51210000	-2.27182000
C	-1.74957800	0.84182200	-1.72436700
H	-0.79141300	1.17349100	-2.12388000
H	-1.90960700	1.36669700	-0.78133800
N	-1.60519000	-0.62311800	-1.41747100
N	-3.35007500	-0.25709400	-3.29698000
O	-0.24191900	2.42567500	0.79762200
H	0.25169600	2.20970100	-0.00918400
H	-0.54324300	1.53990900	1.09003000

PC*

E = -3089.93305277 a.u.

0 1

N	7.62669500	3.94149400	1.79936000
N	7.81241000	2.28695900	-0.29166600
N	9.76309800	4.69000400	-0.06846700
N	5.73738800	4.33395700	-0.72340300
C	7.66305700	2.61477200	2.08873600
C	7.76178400	1.69760000	0.93103400
C	10.17744800	6.04517100	0.14671800
C	5.39223900	4.47548100	-2.03296500
C	10.69038900	3.72256900	-0.09563700
C	4.79241900	4.11131600	0.20839100
C	7.52407400	4.82597600	2.80110200
C	7.84973700	1.50889200	-1.38048400
C	7.58471600	2.18079100	3.41302200
C	7.77389100	0.31329000	1.05506000
C	11.56893100	6.31325000	0.30305900
C	4.04120900	4.43035400	-2.39160200
C	12.05512700	3.94665300	0.06279300
C	3.43776700	4.04656000	-0.08320000
C	7.44580300	4.44657700	4.13549500
C	7.86601700	0.12215800	-1.31099900
C	7.47111300	3.08711300	4.47466800

C	7.80188500	-0.51776000	-0.07180000
C	12.49473600	5.30728100	0.26509000
C	3.07287600	4.21729500	-1.42564800
H	10.31587100	2.71873500	-0.26717100
H	5.15001400	3.98323300	1.22254500
H	7.49255200	5.86830600	2.50097400
H	7.85471600	2.02691100	-2.33153600
H	7.60267100	1.11884900	3.61979000
H	7.70706300	-0.13382100	2.03818400
H	11.88606400	7.34112600	0.44710700
H	3.75886400	4.53708300	-3.43216300
H	7.36158400	5.22009400	4.88816200
H	7.85572800	-0.43528700	-2.23602200
H	13.55386900	5.51749100	0.37807900
H	2.02678700	4.15341600	-1.71006900
P	4.14491300	0.32403900	-1.31126000
F	5.03599200	0.69112700	0.02998800
F	4.89631400	1.56449500	-2.09007000
F	2.96669900	1.33843000	-0.79357400
F	3.28345400	-0.01259200	-2.64261800
F	3.45741500	-0.91107000	-0.49733900
F	5.33479000	-0.69700600	-1.82184400
C	6.52697800	4.66044400	-2.93809200
C	6.40950700	4.77480200	-4.33041600
C	7.80413300	4.70436000	-2.31307800
C	7.54177900	4.93940600	-5.12308400
H	5.43640700	4.72613100	-4.80978700
C	8.93652200	4.87630100	-3.11914500
C	8.78509700	4.98887200	-4.49590600
H	7.48056500	5.02404300	-6.20243600
H	9.93410600	4.92362300	-2.69571600
F	9.88741600	5.14859700	-5.26094200
C	7.37341600	2.57310000	5.91607800
C	7.26082500	3.72202900	6.93477200
H	6.36314600	4.32791000	6.76725500
H	7.19479400	3.30898300	7.94672300
H	8.13577800	4.38143900	6.90375600
C	6.11927700	1.67588600	6.04597500
H	5.20843800	2.23784200	5.81183000
H	6.16125800	0.81089500	5.37617100
H	6.03456100	1.29957800	7.07178500
C	8.63953000	1.74725600	6.24581900
H	8.74928100	0.88244100	5.58327100
H	9.54326800	2.36021900	6.15447400

H	8.58315000	1.37265200	7.27414300
C	7.69873300	-2.03542400	0.09608500
C	6.36765200	-2.35636700	0.82190600
H	6.25556500	-3.44344600	0.91210200
H	6.34761200	-1.93791500	1.83489000
H	5.51513000	-1.95388900	0.27255200
C	8.89415400	-2.53687800	0.93913000
H	8.81833100	-3.62068900	1.08350800
H	9.84770600	-2.32648500	0.43984900
H	8.92281000	-2.07307500	1.93193600
C	7.70394100	-2.76740100	-1.25808500
H	6.84898100	-2.46414200	-1.86704400
H	8.62897700	-2.58182600	-1.81815300
H	7.63463300	-3.84720600	-1.08726700
C	2.42919000	3.72790000	0.98568600
H	2.82550700	3.92419900	1.98772100
H	1.51212300	4.31384300	0.85919600
H	2.16962200	2.66648100	0.91847600
C	13.04303300	2.81868400	0.01483600
H	13.62023500	2.75695000	0.94810800
H	12.55279800	1.85275200	-0.14385700
H	13.77112500	2.96483300	-0.79520500
C	9.12064300	6.97475600	0.17102500
C	9.27648300	8.38351700	0.39954500
C	7.77272400	6.42796500	-0.06445600
C	8.17648000	9.20946300	0.40019200
H	10.26081600	8.80598100	0.57474700
C	6.68925900	7.30421500	-0.06437500
C	6.89219700	8.66260000	0.16902500
H	8.26516700	10.27741400	0.57257400
H	5.68075300	6.95002000	-0.25086400
F	5.83623800	9.50390300	0.18246600
Ir	7.76058100	4.45754500	-0.32691900

PC⁻

E = -3090.06834218 a.u.

-1 2

N	7.69544800	4.08795800	1.82147800
N	7.86108400	2.41018900	-0.26499700
N	9.84529300	4.83384900	0.03366000
N	5.84509400	4.30642800	-0.69788900
C	7.73865400	2.71920300	2.10999000
C	7.76467200	1.83103600	0.99308200
C	10.25034900	6.12056200	0.25669800

C	5.51725100	4.34712300	-2.02231700
C	10.73790800	3.82640400	0.04145200
C	4.94118400	3.89095900	0.21042500
C	7.64408700	4.96185800	2.84045600
C	7.75051100	1.62190200	-1.35133200
C	7.71653000	2.31087100	3.47248800
C	7.64768000	0.41979200	1.08749800
C	11.61152500	6.37535300	0.48252200
C	4.22722100	3.98617100	-2.41876100
C	12.09777900	4.01590500	0.26193300
C	3.65909600	3.47852200	-0.12961400
C	7.61818700	4.59982700	4.17446800
C	7.60468700	0.25012600	-1.29570000
C	7.65555500	3.21055800	4.51642200
C	7.55847000	-0.38970400	-0.02947500
C	12.52709300	5.33402100	0.48399200
C	3.30684900	3.54363500	-1.48331800
H	10.31959600	2.84222300	-0.13799500
H	5.29496500	3.85137700	1.23353800
H	7.61726700	6.00757100	2.54779900
H	7.76913200	2.14219000	-2.30336400
H	7.75545100	1.24771600	3.68403700
H	7.56715400	-0.02259100	2.07376100
H	11.94222700	7.39373500	0.65497300
H	3.97535200	3.96795900	-3.47220100
H	7.57057100	5.37809700	4.92482800
H	7.45201000	-0.29492700	-2.21564000
H	13.58132600	5.53784400	0.65896900
H	2.34362300	3.16759900	-1.80584100
P	3.57133000	-0.08746600	-1.92680600
F	4.46426200	0.65644700	-0.76565300
F	3.98305900	1.08402200	-2.99314600
F	2.25540100	0.79395900	-1.46140000
F	2.67734900	-0.82691300	-3.07122100
F	3.16503600	-1.25081200	-0.84601600
F	4.88958300	-0.96021500	-2.37582300
C	6.62691700	4.70648800	-2.90963400
C	6.49728700	4.87467900	-4.29533700
C	7.88864500	4.84164000	-2.26624000
C	7.60871800	5.18436300	-5.07606500
H	5.52987800	4.76511200	-4.77798100
C	8.99726800	5.15443000	-3.06697600
C	8.83872400	5.31674300	-4.43700700
H	7.54035000	5.32034700	-6.15017400

H	9.98735800	5.27768400	-2.63809500
F	9.92776300	5.61976600	-5.19239300
C	7.63475800	2.70987100	5.97025400
C	7.55068300	3.86771900	6.98295600
H	6.64061100	4.46147600	6.84081300
H	7.53392800	3.46897400	8.00461000
H	8.41275500	4.53957400	6.90052500
C	6.40772700	1.79362500	6.18608200
H	5.47810000	2.33882700	5.98695600
H	6.43104200	0.92488000	5.52049300
H	6.37618000	1.42577800	7.22057300
C	8.92644400	1.91062100	6.26094200
H	9.02025300	1.04455900	5.59780900
H	9.81191800	2.54016300	6.11449100
H	8.93157200	1.54499000	7.29673000
C	7.36343000	-1.90504900	0.13192100
C	5.98108500	-2.15946500	0.77748800
H	5.81154300	-3.23883900	0.89255600
H	5.91783600	-1.70093000	1.77141900
H	5.18279100	-1.74425600	0.16004600
C	8.47813400	-2.48064300	1.03562300
H	8.34321400	-3.56321600	1.15779900
H	9.46876000	-2.30788500	0.59648400
H	8.47224600	-2.03193700	2.03500900
C	7.41026000	-2.64409100	-1.21901300
H	6.58851700	-2.32924600	-1.86513500
H	8.36110200	-2.47178300	-1.73938900
H	7.31297100	-3.72356200	-1.04879900
C	2.74120900	2.86649700	0.89264300
H	3.12646000	2.99561700	1.90972700
H	1.73557800	3.30155800	0.84470700
H	2.65343100	1.79704000	0.67772000
C	13.05482400	2.85072600	0.26228300
H	13.57372300	2.76219100	1.22519800
H	12.53177300	1.90739000	0.07711500
H	13.82358600	2.96572800	-0.51241700
C	9.17024500	7.10245900	0.22315900
C	9.35455300	8.47728900	0.44115800
C	7.87880100	6.55846500	-0.04675300
C	8.27046700	9.34665200	0.39432600
H	10.34223300	8.88142200	0.65107600
C	6.79866600	7.45482100	-0.09604100
C	7.01132400	8.80802200	0.12281400
H	8.37551000	10.41391800	0.55753400

H	5.79253900	7.10384100	-0.30372200
F	5.95492700	9.65750500	0.07604600
Ir	7.84107800	4.56349100	-0.26927300

INT-3

E = -1165.49372286 a.u.

1 2

C	3.04040900	-0.29783700	0.04589000
C	2.30183800	1.42532800	-1.57673700
C	3.49495600	0.95035600	-0.73021300
H	2.77365900	-1.11185700	-0.64400800
H	3.83289800	-0.65708900	0.70832600
H	2.49425900	2.41066500	-2.01124800
H	2.12497500	0.72168400	-2.40201000
B	0.90025900	0.72932800	0.29541900
O	1.08069500	1.54526400	-0.81407500
O	1.91134200	0.00136700	0.87884100
C	3.93387400	2.05360700	0.24930300
H	4.77219800	1.70961500	0.86474600
H	4.26030000	2.94551600	-0.29674600
H	3.12357200	2.34404000	0.92470600
C	4.65204900	0.57574300	-1.66919500
H	4.96229100	1.43909400	-2.26843600
H	5.52258700	0.24402300	-1.09304800
H	4.37343200	-0.23199200	-2.35674000
C	-1.79996500	-1.38881000	2.15944700
C	0.21391200	-2.64535700	1.31518100
C	-0.54983100	-2.16793900	2.59057200
H	-2.51713800	-2.04885900	1.65689400
H	-2.29400200	-0.93402300	3.02152200
H	1.29062000	-2.50063800	1.44798100
H	0.02999600	-3.70834300	1.13327800
B	-0.69938400	-0.71478400	0.10206600
O	-0.17041200	-1.98725800	0.09635200
O	-1.48647400	-0.30535700	1.24223900
C	0.34910600	-1.28615900	3.47887700
H	-0.20397500	-0.93189100	4.35662000
H	1.20559400	-1.86579500	3.84008400
H	0.75300900	-0.42484500	2.94478600
C	-1.00860100	-3.39609200	3.39953300
H	-0.14550500	-4.00186000	3.69654400
H	-1.52815900	-3.09281100	4.31550600
H	-1.68387600	-4.03508000	2.81813000
C	-2.71544500	-1.39958100	-1.27049600

C	-3.45254400	-1.27219300	-2.63624700
H	-2.33581000	-2.40478000	-1.08191000
H	-3.33968600	-1.09553500	-0.42774600
H	-3.37726200	-2.20460500	-3.20332300
H	-4.51528900	-1.06679700	-2.47738800
C	-0.65759600	-0.79647700	-2.47393300
H	0.23069800	-0.17103300	-2.40595900
H	-0.35558700	-1.83901800	-2.36707700
C	-1.48334800	-0.52366800	-3.76579200
H	-1.05374100	0.30968400	-4.32980100
H	-1.47334400	-1.40246900	-4.41712900
C	-2.91059100	1.05456500	-2.67018100
H	-2.56533000	1.87373200	-3.30782300
H	-3.94982400	1.26302300	-2.39925900
C	-2.01649400	0.95044400	-1.39737600
H	-1.13505900	1.58956800	-1.45785100
H	-2.56360700	1.17484000	-0.47905100
N	-1.51022800	-0.46950700	-1.26729500
N	-2.87481900	-0.19193900	-3.44086300
O	0.12822600	1.55231900	1.40066800
H	-0.14712900	2.41853300	1.04548500
H	-0.69256900	0.96409200	1.61551700

INT-4

E = -1510.90631550 a.u.

1 2

C	2.35969800	0.39360100	1.19357400
C	2.35183500	0.78059700	-1.24153500
C	3.18659700	0.97245800	0.03485100
H	2.24583300	-0.68883900	1.05663100
H	2.86147400	0.56736500	2.15255900
H	2.84530400	1.24440500	-2.10269000
H	2.22926700	-0.29196000	-1.44721300
B	0.36077700	1.22275300	0.08499900
O	1.05926000	1.38368100	-1.12635900
O	1.06458200	0.99632000	1.28749800
C	3.47442100	2.46431300	0.27049500
H	4.06242700	2.60452500	1.18507000
H	4.04496600	2.88656500	-0.56461100
H	2.54786900	3.03720700	0.37196700
C	4.49878800	0.18608600	-0.09354100
H	5.09835500	0.56273400	-0.93019300
H	5.10249500	0.28405700	0.81616400
H	4.31476100	-0.88171100	-0.26369700

C	-1.85636700	-1.86183500	1.72141500
C	0.16683700	-2.81727400	0.63621600
C	-0.54826700	-2.62644300	1.98702700
H	-2.55863700	-2.51360900	1.18077700
H	-2.33369900	-1.59146500	2.67192700
H	1.19138000	-3.17027900	0.79751000
H	-0.35655800	-3.59238700	0.05505700
B	-0.73899400	-0.63610500	-0.08659400
O	0.24611700	-1.63106000	-0.15798300
O	-1.67518900	-0.64538600	0.98089400
C	0.33771400	-1.85062700	2.97379900
H	-0.16737200	-1.75001400	3.94324500
H	1.28031300	-2.38161200	3.14835400
H	0.56962400	-0.85462100	2.59691500
C	-0.88586300	-4.00819600	2.57093900
H	0.02781200	-4.58735000	2.74668700
H	-1.40275200	-3.90880000	3.53258100
H	-1.52938000	-4.58989400	1.90019500
C	-2.45084900	-1.82862800	-1.59216600
C	-3.11994700	-1.93489500	-2.99355700
H	-1.82306300	-2.69236200	-1.36818000
H	-3.18542900	-1.71285000	-0.79386700
H	-2.70635000	-2.77497900	-3.55984700
H	-4.19599700	-2.10614600	-2.88792400
C	-0.63103800	-0.65659200	-2.73098300
H	0.06097300	0.17592700	-2.62070200
H	-0.07021000	-1.58754600	-2.63770600
C	-1.46632100	-0.57452800	-4.04114700
H	-1.30415800	0.38372500	-4.54476200
H	-1.16727700	-1.36456300	-4.73703200
C	-3.34283600	0.43760200	-2.95778800
H	-3.29957500	1.34090000	-3.57446700
H	-4.39044800	0.27869700	-2.68197700
C	-2.45080200	0.60693100	-1.69304800
H	-1.79044600	1.46840200	-1.76374700
H	-3.02470100	0.69961200	-0.76919500
N	-1.56458800	-0.60127000	-1.54327900
N	-2.90341800	-0.70763100	-3.76423100
O	-0.69267000	2.21674400	0.23128900
H	-0.48645900	2.95201600	-0.36831100
H	-1.17041900	2.44400600	1.77523000
C	-3.07398200	1.98504100	2.46768800
C	-3.76872500	1.87238400	3.85831800
H	-3.53799800	2.73101000	1.81681800

H	-2.99961100	1.04047500	1.92508900
H	-4.68891200	2.46331200	3.87925300
H	-4.03796700	0.83374900	4.07168700
C	-1.64558900	3.81056100	3.32533300
H	-0.59896200	4.08006500	3.48780900
H	-2.07297400	4.50233600	2.59461100
C	-2.47034900	3.73052300	4.64960400
H	-1.87694800	4.09865700	5.49105700
H	-3.36915200	4.35000700	4.58198100
C	-1.68479500	1.48906100	4.98809600
H	-1.02438900	1.87389200	5.77055300
H	-1.99190200	0.48120200	5.28317300
C	-0.95857100	1.45484600	3.61309700
H	0.09293300	1.74312400	3.64972600
H	-1.01897800	0.49672400	3.10307000
N	-1.65763500	2.43929600	2.71208400
N	-2.87707800	2.34737800	4.92505600

HO-Bneop

E = -447.837867322 a.u.

0 1

C	-2.57896600	1.29669200	-0.33367100
C	-2.96525500	3.65363000	0.30608300
C	-3.28223200	2.59517500	-0.76563500
H	-3.05403500	0.89756300	0.57452900
H	-3.34312200	4.63515700	-0.00336200
H	-3.46236000	3.38808000	1.25098900
H	-2.66417900	0.53447800	-1.11715200
B	-0.74687000	2.71330200	0.37577900
O	-1.56768200	3.79442100	0.54453600
O	-1.18791100	1.48898500	-0.07723000
C	-4.79808300	2.36087500	-0.82283600
H	-5.32287900	3.28037100	-1.10788700
H	-5.04589300	1.59380700	-1.56619100
H	-5.19390600	2.03283900	0.14611100
C	-2.76480800	3.05357600	-2.14069900
H	-2.97100400	2.29360000	-2.90357800
H	-3.25745300	3.98393800	-2.44642600
H	-1.68619500	3.23507200	-2.12946000
O	0.58335500	2.87904800	0.65063800
H	1.05876300	2.05284300	0.48370600

$HA1^+$

E = -345.726631674 a.u.

1 1

C	2.16905700	1.24956900	-3.63558700
C	1.41380900	1.39018600	-4.99571400
H	2.65949100	0.27491800	-3.57295500
H	1.46604400	1.32268500	-2.80202000
H	1.54659700	0.53129900	-5.65785600
H	0.34501600	1.58612700	-4.88156200
C	4.17276100	2.19404600	-4.56993600
H	4.93851000	2.95948600	-4.42134600
H	4.66058400	1.21818200	-4.50618600
C	3.49338500	2.37042400	-5.96545900
H	3.85510500	3.24060800	-6.51833000
H	3.57537400	1.48744400	-6.60369400
C	1.80649200	3.85137700	-4.87564300
H	2.20938200	4.68538400	-5.45516800
H	0.72810100	3.98705800	-4.76436000
C	2.54733200	3.62098500	-3.51990200
H	3.31517300	4.38459700	-3.37273400
H	1.84383100	3.69116800	-2.68653800
N	3.18101600	2.30002100	-3.49649700
N	2.01116100	2.59443600	-5.70748200
H	1.53583600	2.71413800	-6.60592400

BN

E = -717.242764373 a.u.

0 2

C	2.92786500	1.50389200	0.01816300
C	4.59390600	0.04280800	-1.05597600
C	4.14843400	0.61117600	0.30283100
H	2.09410200	0.86157700	-0.32315600
H	2.59614900	2.00010100	0.94063000
H	5.50865200	-0.55413400	-0.93720500
H	3.80623000	-0.63999000	-1.42689600
B	4.12968000	2.27439100	-1.98892500
O	4.85857400	1.05375200	-2.01587300
O	3.19541100	2.51249100	-0.94352000
C	5.28059000	1.43691000	0.93619100
H	4.95877200	1.86054500	1.89562500
H	6.16009400	0.80776400	1.12104600
H	5.58045100	2.26025500	0.28229900
C	3.74296400	-0.53733600	1.23600500
H	4.59408200	-1.20174100	1.43016000
H	3.39565300	-0.15124500	2.20236200
H	2.93507800	-1.14286300	0.80590000

C	2.24238000	1.26103400	-3.52859700
C	1.46854700	1.37008400	-4.87715300
H	2.79055800	0.32129500	-3.43045500
H	1.59315700	1.37746600	-2.65790800
H	1.58976300	0.45867900	-5.47279800
H	0.39609300	1.51000600	-4.70278400
C	4.20202400	2.22454700	-4.60667100
H	4.94543100	3.01882100	-4.49421700
H	4.71794600	1.27123100	-4.48000300
C	3.39436800	2.31945500	-5.93429400
H	3.74178400	3.16065800	-6.54396200
H	3.51705000	1.40962500	-6.53182700
C	1.77882800	3.74308500	-4.89119400
H	2.12580600	4.58420500	-5.50122600
H	0.70684400	3.88565400	-4.71681200
C	2.55609500	3.67599600	-3.54382700
H	3.32658400	4.44643500	-3.44946200
H	1.91066600	3.74735500	-2.66667600
N	3.26421800	2.36033200	-3.44750800
N	1.95773600	2.50731200	-5.67158300

11) References

[1] C.-M. Zhu, R.-B. Liang, Y. Xiao, W. Zhou, Q.-X. Tong and J.-J. Zhong, *Green Chem.* 2023, **25**, 960.

[2] R.-B. Liang, C.-M. Zhu, P.-Q. Song, L.-M. Zhao, Q.-X. Tong and J.-J. Zhong, *Org. Chem. Front.* 2022, **9**, 4536.

[3] B. Du, C.-M. Chan and W.-Y. Yu, *Nat. Commun.*, 2021, **12**, 412.

[4] X. Lu, X.-X. Wang, S.-J. He and Y. Fu, *Chem. Sci*. 2019, **10**, 809.

[5] Gaussian 16, Revision A.03, M. J. Frisch, G. W. Trucks, H. B. Schlegel, G. E. Scuseria, M. A. Robb, J. R. Cheeseman, G. Scalmani, V. Barone, G. A. Petersson, H. Nakatsuji, X. Li, M. Caricato, A. V. Marenich, J. Bloino, B. G. Janesko, R. Gomperts, B. Mennucci, H. P. Hratchian, J. V. Ortiz, A. F. Izmaylov, J. L. Sonnenberg, D. Williams-Young, F. Ding, F. Lipparini, F. Egidi, J. Goings, B. Peng, A. Petrone, T. Henderson, D. Ranasinghe, V. G. Zakrzewski, J. Gao, N. Rega, G. Zheng, W. Liang, M. Hada, M. Ehara, K. Toyota, R. Fukuda, J. Hasegawa, M. Ishida, T. Nakajima,; Y. Honda, O. Kitao, H. Nakai, T. Vreven, K. Throssell, J. A. Montgomery Jr., J. E. Peralta, F. Ogliaro, M. J. Bearpark, J. J. Heyd, E. N. Brothers, K. N. Kudin, V. N. Staroverov, T. A. Keith, R. Kobayashi, J. Normand, K. Raghavachari, A. P. Rendell, J. C. Burant, S. S. Iyengar, J. Tomasi, M. Cossi, J. M. Millam, M. Klene, C. Adamo, R. Cammi, J. W. Ochterski, R. L. Martin, K. Morokuma, O. Farkas, J. B. Foresman, D. J. Fox, Gaussian16, Gaussian, Inc., Wallingford, CT, **2016**.

[6] S. H. Vosko, L. Wilk and M. Nusair, *Can. J. Phys.*, 1980, **58**, 1200.

[8] C. Lee, W. Yang and R. G. Parr, *Phys. Rev. B*: *Condens. Matter Mater. Phys.* 1988, **37**, 785.

[9] Becke, A. D. *J. Chem. Phys.* 1993, **98**, 5648.

[10] P. J. Stephens, F. J. Devlin, C. F. Chabalowski and M. J. Frisch, *J. Phys. Chem.* 1994, **98**, 11623.

[11] G. Stefan, A. Jens, E. Stephan and K. Helge, *J. Chem. Phys*., 2010, **132**, 154104.

[12] A. V. Marenich, C. J. Cramer and D. G. Truhlar, *J. Phys. Chem. B.* 2009, **113**, 6378.

[13] M. Mammen, E. I. Shakhnovich, J. M. Deutch and G. M. Whitesides, *J. Org. Chem.* 1998, **63**, 3821.

[14] G. Jindal and R. B. Sunoj, *J. Am. Chem. Soc.* 2014, **136**, 15998.

[15] G. Zeng and S. Li, *Inorg. Chem.* 2011, **50**, 10572.

[16] Gauss View, Version 6.0.16, R. Dennington, T. A. Keith, J. M. Millam, Semichem Inc., Shawnee Mission, KS, 2016.

2. Characterization data of the products

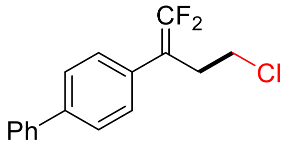

1

Purification by column chromatography on silica gel (hexane) afforded **1** as a white solid (50.6 mg, 91% yield).

^{1}H NMR (400 MHz, Chloroform-*d*) δ 7.66 – 7.59 (m, 4H), 7.48 (t, *J* = 7.6 Hz, 2H), 7.41 (t, *J* = 7.7 Hz, 3H), 3.54 (t, *J* = 7.1 Hz, 2H), 2.93 (tt, *J* = 6.9, 2.1 Hz, 2H).

^{13}C NMR (101 MHz, $CDCl_3$) δ 154.55 (dd, *J* = 291.5, 289.5 Hz), 140.61, 140.40, 131.23, 128.87, 128.68 (t, *J* = 3.1 Hz), 127.56, 127.38, 127.05, 89.31 (dd, *J* = 21.3, 15.8 Hz), 41.76, 31.21.

^{19}F NMR (376 MHz, Chloroform-*d*) δ -88.81 (d, *J* = 37.7 Hz, 1F), -89.13 (d, *J* = 37.7 Hz, 1F).

HRMS (ESI) m/z calcd. for $C_{16}H_{14}ClF_2$ $[M+H]^+$: 279.0747, found: 279.0746.

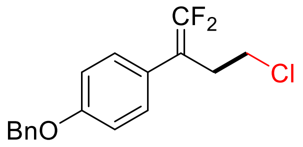

2

Purification by column chromatography on silica gel (hexane/ethyl acetate = 50/1, v/v) afforded **2** as a white solid (54.8 mg, 89% yield).

^{1}H NMR (400 MHz, Chloroform-*d*) δ 7.45 (d, *J* = 13.7 Hz, 4H), 7.40 – 7.35 (m, 1H), 7.27 (d, *J* = 8.7 Hz, 2H), 7.02 (d, *J* = 8.8 Hz, 2H), 5.10 (s, 2H), 3.50 (t, *J* = 7.1 Hz, 2H), 2.86 (tt, *J* = 7.0, 2.0 Hz, 2H).

^{13}C NMR (101 MHz, $CDCl_3$) δ 158.31, 154.35 (t, *J* = 291.2 Hz), 136.81, 129.52 (t, *J* = 3.2 Hz), 128.64, 128.07, 127.48, 124.66, 115.07, 89.03 (dd, *J* = 21.5, 16.4 Hz), 70.08, 41.76, 31.41.

^{19}F NMR (376 MHz, Chloroform-*d*) δ = -90.11 (d, *J* = 40.6, 1F), -90.57 (d, *J* = 40.6, 1F).

HRMS (ESI) m/z calcd. for $C_{17}H_{16}ClF_2O$ $[M+H]^+$: 309.0852, found: 309.0855.

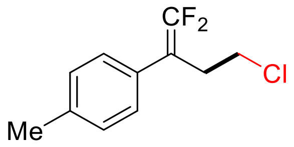

3

Purification by column chromatography on silica gel (hexane) afforded **3** as a colorless oil (35.0 mg, 81% yield).

^{1}H NMR (400 MHz, Chloroform-*d*) δ 7.21 (s, 4H), 3.48 (t, *J* = 7.1 Hz, 2H), 2.86 (ddq, *J* = 7.2, 4.5, 2.2 Hz, 2H), 2.38 (s, 3H).

^{13}C NMR (101 MHz, $CDCl_3$) δ 154.37 (dd, *J* = 290.4, 288.4 Hz), 137.60, 129.38, 128.17 (t, *J* = 3.0 Hz), 89.35 (dd, *J* = 21.2, 16.4 Hz), 41.74, 31.32, 21.13.

^{19}F NMR (376 MHz, Chloroform-*d*) δ -89.76 (d, *J* = 39.7 Hz, 1F), -90.26 (d, *J* = 39.7 Hz, 1F).

HRMS (ESI) m/z calcd. for $C_{11}H_{12}ClF_2$ $[M+H]^+$: 217.0590, found: 217.0599.

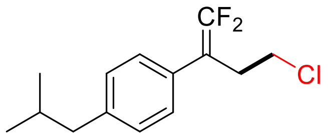

4

Purification by column chromatography on silica gel (hexane) afforded **4** as a colorless oil (31.0 mg, 60% yield).

^{1}H NMR (400 MHz, Chloroform-*d*) δ 7.25 (s, 2H), 7.17 (d, *J* = 8.2 Hz, 2H), 3.49 (t, *J* = 7.2 Hz, 2H), 2.87 (tt, *J* = 7.2, 2.1 Hz, 2H), 2.49 (d, *J* = 7.2 Hz, 2H), 1.88 (dq, *J* = 13.6, 6.8 Hz, 1H), 0.94 (s, 3H), 0.93 (s, 3H).

^{13}C NMR (101 MHz, $CDCl_3$) δ 154.39 (dd, *J* = 291.1, 288.5 Hz), 141.36, 129.50, 129.38, 127.94 (t, *J* = 3.2 Hz), 89.39 (dd, *J* = 21.2, 16.0 Hz), 45.08, 41.75, 31.35, 30.12, 22.35.

^{19}F NMR (376 MHz, Chloroform-*d*) δ = -89.62 (d, *J* = 39.5, 1F), -90.10 (d, *J* = 39.5, 1F).

HRMS (ESI) m/z calcd. for $C_{14}H_{18}ClF_2$ $[M+H]^+$: 259.1060, found: 259.1062.

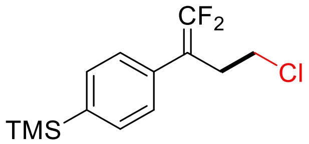

5

Purification by column chromatography on silica gel (hexane) afforded **5** as a colorless oil (46.0 mg, 84% yield).

^{1}H NMR (400 MHz, Chloroform-*d*) δ 7.56 (d, *J* = 8.0 Hz, 2H), 7.33 (d, *J* = 7.2 Hz, 2H), 3.50 (t, *J* = 7.1 Hz, 2H), 2.89 (tt, *J* = 7.1, 2.1 Hz, 2H), 0.30 (s, 9H).

^{13}C NMR (101 MHz, $CDCl_3$) δ 154.51 (dd, *J* = 291.7, 289.1 Hz), 140.20, 133.66, 132.66, 127.52 (t, *J* = 3.1 Hz), 89.56 (dd, *J* = 21.2, 16.0 Hz), 41.72, 31.21, -1.19.

^{19}F NMR (376 MHz, Chloroform-*d*) δ -89.02 (d, *J* = 37.7 Hz, 1F), -89.37 (d, *J* = 37.9 Hz, 1F).

HRMS (ESI) m/z calcd. for $C_{13}H_{18}ClF_2Si$ $[M+H]^+$: 275.0829, found: 275.0823.

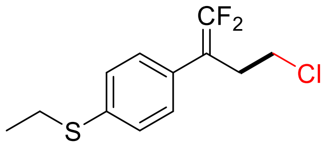

6

Purification by column chromatography on silica gel (hexane/ethyl acetate = 50/1, v/v) afforded **6** as a yellow oil (26.2 mg, 50% yield).

^{1}H NMR (400 MHz, Chloroform-*d*) δ 7.31 (d, *J* = 8.5 Hz, 2H), 7.24 (d, *J* = 8.4 Hz, 2H), 3.48 (t, *J* = 7.1 Hz, 2H), 2.97 (q, *J* = 7.4 Hz, 2H), 2.85 (tt, *J* = 7.1, 2.1 Hz, 2H), 1.34 (t, *J* = 7.4 Hz, 3H).

^{13}C NMR (101 MHz, $CDCl_3$) δ 154.44 (dd, *J* = 291.7, 289.3 Hz), 136.67, 129.49, 128.66 (t, *J* = 3.4 Hz), 128.64, 89.11 (dd, *J* = 21.5, 15.7 Hz), 41.67, 31.13, 27.29, 14.29.

^{19}F NMR (376 MHz, Chloroform-*d*) δ -88.96 (d, *J* = 38.0 Hz, 1F), -89.36 (d, *J* = 38.0 Hz, 1F).

HRMS (ESI) m/z calcd. for $C_{12}H_{14}ClF_2S$ $[M+H]^+$: 263.0467, found: 263.0469.

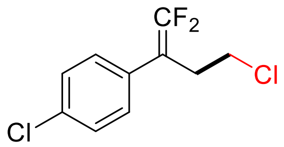

7

Purification by column chromatography on silica gel (hexane) afforded **7** as a colorless oil (28.3 mg, 60% yield).

^{1}H NMR (400 MHz, Chloroform-*d*) δ 7.36 (d, *J* = 8.5 Hz, 2H), 7.26 (d, *J* = 8.0 Hz, 2H), 3.46 (t, *J* = 6.9 Hz, 2H), 2.85 (ddt, *J* = 6.9, 4.7, 2.2 Hz, 2H).

^{13}C NMR (101 MHz, $CDCl_3$) δ 154.47 (dd, *J* = 291.5, 289.6 Hz), 133.68, 130.78, 129.67 (t, *J* = 3.2 Hz), 128.92, 88.81 (dd, *J* = 21.9, 15.9 Hz), 41.51, 31.10.

^{19}F NMR (376 MHz, Chloroform-*d*) δ -88.55 (d, *J* = 37.0 Hz, 1F), -88.92 (d, *J* = 36.9 Hz, 1F).

HRMS (ESI) m/z calcd. for $C_{10}H_9Cl_2F_2$ $[M+H]^+$: 237.0044, found: 237.0044.

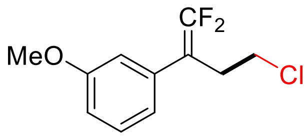

8

Purification by column chromatography on silica gel (hexane/ethyl acetate = 50/1, v/v) afforded **8** as a colorless oil (30.2 mg, 65% yield).

^{1}H NMR (400 MHz, Chloroform-*d*) δ 7.30 (t, *J* = 8.3 Hz, 1H), 6.91 (d, *J* = 7.7 Hz, 1H), 6.86 (d, *J* = 5.4 Hz, 2H), 3.83 (s, 3H), 3.48 (t, *J* = 7.0 Hz, 2H), 2.86 (ddt, *J* = 7.0, 5.1, 2.2 Hz, 2H).

^{13}C NMR (101 MHz, $CDCl_3$) δ 159.73, 154.45 (dd, *J* = 291.2, 288.7 Hz), 133.67, 129.67, 120.67 (t, *J* = 3.1 Hz), 114.39 (t, *J* = 3.4 Hz), 113.02, 89.51 (dd, *J* = 21.1, 15.9 Hz), 55.25, 41.69, 31.33.

^{19}F NMR (376 MHz, Chloroform-*d*) δ -88.73 (d, *J* = 38.0 Hz, 1F), -89.65 (d, *J* = 38.1 Hz, 1F).

HRMS (ESI) m/z calcd. for $C_{11}H_{12}ClF_2O$ $[M+H]^+$: 233.0539, found: 233.0530.

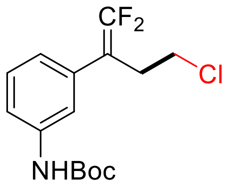

9

Purification by column chromatography on silica gel (hexane/ethyl acetate = 20/1, v/v) afforded **9** as a brown oil (38.0 mg, 60% yield).

^{1}H NMR (400 MHz, Chloroform-*d*) δ 7.35 (s, 1H), 7.26 (d, *J* = 5.2 Hz, 2H), 6.99 – 6.91 (m, 1H), 6.67 (s, 1H), 3.44 (t, *J* = 7.0 Hz, 2H), 2.81 (t, *J* = 6.9 Hz, 2H), 1.50 (s, 9H).

^{13}C NMR (101 MHz, $CDCl_3$) δ 154.43 (dd, *J* = 292.3, 289.9 Hz), 152.71, 138.80, 133.12, 129.23, 122.98 (t, *J* = 2.9 Hz), 118.37, 117.92, 89.46 (dd, *J* = 21.6, 16.0 Hz), 80.72, 41.68, 31.25, 28.30.

^{19}F NMR (376 MHz, Chloroform-*d*) δ -88.89 (d, *J* = 38.0 Hz, 1F), -89.79 (d, *J* = 38.1 Hz, 1F).

HRMS (ESI) m/z calcd. for $C_{15}H_{19}ClF_2NO_2$ $[M+H]^+$: 318.1067, found: 318.1066.

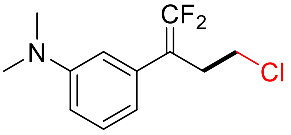

10

Purification by column chromatography on silica gel (hexane/ethyl acetate = 20/1, v/v) afforded **10** as a brown oil (23.5 mg, 48% yield).

^{1}H NMR (400 MHz, Chloroform-*d*) δ 7.27 – 7.21 (m, 1H), 6.71 – 6.62 (m, 3H), 3.49 (t, *J* = 7.1 Hz, 2H), 2.97 (s, 6H), 2.84 (tt, *J* = 7.1, 2.2 Hz, 2H).

^{13}C NMR (101 MHz, $CDCl_3$) δ 154.35 (dd, *J* = 290.3, 288.4 Hz), 150.68, 133.07, 129.28, 116.52, 112.58, 112.02, 90.15 (dd, *J* = 20.9, 16.2 Hz), 41.86, 40.52, 31.63.

^{19}F NMR (376 MHz, Chloroform-*d*) δ -89.33 (d, *J* = 40.0 Hz, 1F), -90.73 (d, *J* = 40.0 Hz, 1F).

HRMS (ESI) m/z calcd. for $C_{12}H_{15}ClF_2N$ $[M+H]^+$: 246.0856, found: 246.0865.

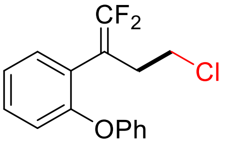

11

Purification by column chromatography on silica gel (hexane/ethyl acetate = 50/1, v/v) afforded **11** as a colorless oil (42.9 mg, 73% yield).

^{1}H NMR (400 MHz, Chloroform-*d*) δ 7.38 – 7.28 (m, 4H), 7.18 – 7.09 (m, 2H), 6.99 – 6.90 (m, 3H), 3.48 (t, *J* = 7.0 Hz, 2H), 2.84 (tt, *J* = 7.0, 2.0 Hz, 2H).

^{13}C NMR (101 MHz, $CDCl_3$) δ 154.99, 154.97 , 154.19 (t, *J* = 290.2 Hz), 131.95, 129.81, 129.74, 123.68, 123.33, 119.28, 118.29, 86.50 (dd, *J* = 23.6, 18.5 Hz), 41.88, 31.24

^{19}F NMR (376 MHz, Chloroform-*d*) δ -87.49 (d, *J* = 37.4 Hz, 1F), -91.04 (d, *J* = 37.4 Hz, 1F).

HRMS (ESI) m/z calcd. for $C_{16}H_{14}ClF_2O$ $[M+H]^+$: 295.0696, found: 295.0687.

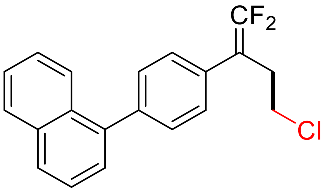

12

Purification by column chromatography on silica gel (hexane) afforded **12** as a white solid (49.9 mg, 76% yield).

^{1}H NMR (400 MHz, Chloroform-*d*) δ 7.99 – 7.94 (m, 2H), 7.92 (d, *J* = 8.2 Hz, 1H), 7.60 – 7.52 (m, 4H), 7.52 – 7.45 (m, 4H), 3.61 (t, *J* = 7.1 Hz, 2H), 2.98 (ddt, *J* = 7.1, 5.1, 2.1 Hz, 2H).

^{13}C NMR (101 MHz, $CDCl_3$) δ 154.65 (dd, *J* = 291.8, 289.1 Hz), 140.27, 139.52, 133.87, 131.52, 131.27, 130.40, 128.39, 128.18 (t, *J* = 3.2 Hz), 127.91, 126.99, 126.19, 125.90, 125.88, 125.42, 89.44 (dd, *J* = 21.2, 15.8 Hz), 41.84, 31.31.

^{19}F NMR (376 MHz, Chloroform-*d*) δ -88.74 (d, *J* = 37.7 Hz, 1F), -89.02 (d, *J* = 37.7 Hz, 1F).

HRMS (ESI) m/z calcd. for $C_{20}H_{16}ClF_2$ $[M+H]^+$: 329.0903, found: 329.0901.

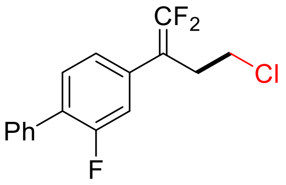

13

Purification by column chromatography on silica gel (hexane/ethyl acetate = 50/1, v/v) afforded **13** as a brown solid (41.4 mg, 70% yield).

^{1}H NMR (400 MHz, Chloroform-*d*) δ 7.60 – 7.55 (m, 2H), 7.50 – 7.44 (m, 3H), 7.41 (t, *J* = 7.3 Hz, 1H), 7.18 (dd, *J* = 15.1, 10.2 Hz, 2H), 3.55 (t, *J* = 7.0 Hz, 2H), 2.91 (tt, *J* = 7.0, 2.2 Hz, 2H).

^{13}C NMR (101 MHz, $CDCl_3$) δ 159.67 (d, *J* = 248.7 Hz), 154.74 (dd, *J* = 293.1, 290.2 Hz), 135.15, 133.29, 130.91 (d, *J*=4.2 Hz), 128.93 (d, *J* = 2.9 Hz), 128.54, 128.37, 127.92, 124.18 (d, *J* = 3.3 Hz), 116.02 (d, *J* = 24.3 Hz), 88.79 (dd, *J* = 22.5, 14.8 Hz), 41.60, 30.95.

^{19}F NMR (376 MHz, Chloroform-*d*) δ -87.44 (d, *J* = 34.9 Hz, 1F), -87.88 (d, *J* = 34.9 Hz, 1F), -117.28, 1F.

HRMS (ESI) m/z calcd. for $C_{16}H_{13}ClF_3$ $[M+H]^+$: 297.0652, found: 297.0642.

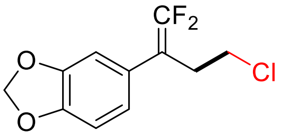

14

Purification by column chromatography on silica gel (hexane/ethyl acetate = 50/1, v/v) afforded **14** as a colorless oil (41.8 mg, 85% yield).

^{1}H NMR (400 MHz, Chloroform-*d*) δ 6.80 (dt, *J* = 14.7, 8.3 Hz, 3H), 5.98 (s, 2H), 3.47 (t, *J* = 7.0 Hz, 2H), 2.80 (tt, *J* = 7.3, 2.3 Hz, 2H).

^{13}C NMR (101 MHz, $CDCl_3$) δ 154.39 (t, *J* = 289.3 Hz), 147.90, 147.16, 125.82, 121.94 (t, *J* = 3.1 Hz), 108.89 (t, *J* = 3.3 Hz), 108.48, 101.25, 89.28 (dd, *J* = 21.9, 16.3 Hz), 41.64, 31.54.

^{19}F NMR (376 MHz, Chloroform-*d*) δ -89.59 (d, *J* = 39.9 Hz, 1F), -90.47 (d, *J* = 40.0 Hz, 1F).

HRMS (ESI) m/z calcd. for $C_{11}H_{10}ClF_2O_2$ $[M+H]^+$: 247.0332, found: 247.0328.

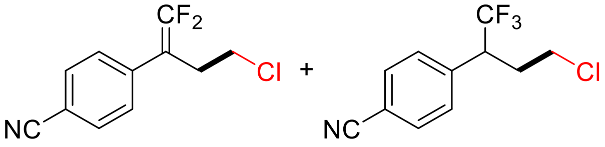

15

Purification by column chromatography on silica gel (hexane/ethyl acetate = 10/1, v/v) afforded **15** as a yellow oil (25.9 mg, 57% yield).

^{1}H NMR (400 MHz, Chloroform-*d*) δ = 7.75 – 7.61 (m, 4H), 7.45 (dt, *J* = 13.9, 7.0 Hz, 4H), 3.80 – 3.69 (m, 1H), 3.67 – 3.56 (m, 1H), 3.54 – 3.41 (m, 2H), 3.13 (tt, *J*=16.4, 8.2 Hz, 1H), 2.93 – 2.86 (m, 2H), 2.56 – 2.40 (m, 1H), 2.39 – 2.24 (m, 1H).

^{13}C NMR (101 MHz, $CDCl_3$) δ = 154.79 (dd, *J* = 293.7, 291.8 Hz), 138.47, 137.42 (t, *J* = 3.3 Hz), 132.69, 132.40, 129.92, 128.98 (t, *J* = 3.4 Hz), 127.55 (t, *J* = 281.0 Hz), 118.39, 118.12, 112.83, 111.51, 89.08 (dd, *J* = 21.6, 15.9 Hz), 47.01 (q, *J* = 27.6 Hz), 41.41, 40.99, 31.41, 30.63.

^{19}F NMR (376 MHz, Chloroform-*d*) δ = -69.02 (s), -86.15 (d, *J* = 31.2 Hz, 1F), -86.32 (d, *J* = 31.3 Hz, 1F).

HRMS (ESI) m/z calcd. for $C_{11}H_9ClF_2N$ and $C_{11}H_{10}ClF_3N$ $[M+H]^+$: 228.0386 and 248.0448, found: 228.0388 and 248.0445.

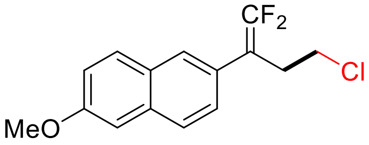

16

Purification by column chromatography on silica gel (hexane/ethyl acetate = 50/1, v/v) afforded **16** as a white solid (42.9 mg, 76% yield).

^{1}H NMR (400 MHz, Chloroform-*d*) δ 7.78 – 7.71 (m, 3H), 7.41 (dt, *J* = 8.6, 1.4 Hz, 1H), 7.20 (dd, *J* = 8.9, 2.5 Hz, 1H), 7.15 (d, *J* = 2.4 Hz, 1H), 3.95 (s, 3H), 3.52 (t, *J* = 7.1 Hz, 2H), 2.96 (tt, *J* = 7.1, 2.1 Hz, 2H).

^{13}C NMR (101 MHz, $CDCl_3$) δ 158.12, 154.60 (dd, *J* = 290.9, 288.9 Hz), 133.87, 129.44, 128.75, 127.31 (t, *J* = 3.3 Hz), 127.20, 126.46 (t, *J* = 3.0 Hz), 119.35, 105.61, 99.99, 89.62 (dd, *J* = 21.0, 16.1 Hz), 55.34, 41.81, 31.40.

^{19}F NMR (376 MHz, Chloroform-*d*) δ = -89.48 (d, *J*=38.9, 1F), -89.75 (d, *J*=39.0, 1F).

HRMS (ESI) m/z calcd. for $C_{15}H_{14}ClF_2O$ $[M+H]^+$: 283.0696, found: 283.0698.

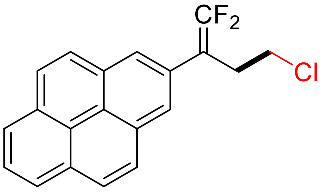

17

Purification by column chromatography on silica gel (hexane/ethyl acetate = 50/1, v/v) afforded **17** as a white solid (41.1 mg, 63% yield).

^{1}H NMR (400 MHz, Chloroform-*d*) δ 8.25 – 8.16 (m, 5H), 8.07 (dq, *J* = 15.3, 8.3, 7.6 Hz, 3H), 7.92 (d, *J* = 7.9 Hz, 1H), 3.48 (t, *J* = 6.6 Hz, 2H), 3.10 (d, *J* = 7.4 Hz, 2H).

^{13}C NMR (101 MHz, $CDCl_3$) δ 154.38 (dd, *J* = 292.0, 288.4 Hz), 131.34, 131.25, 130.85, 129.53, 128.27, 127.98, 127.39 (d, *J* = 1.4 Hz), 127.21, 126.95 (d, *J* = 3.7 Hz), 126.22, 125.55, 125.42, 124.99, 124.79, 124.66, 124.03, 87.82(t, *J* = 21.5 Hz), 41.77, 33.26.

^{19}F NMR (376 MHz, Chloroform-*d*) δ -85.89 (d, *J* = 37.2 Hz, 1F), -90.62 (d, *J* = 37.2 Hz, 1F).

HRMS (ESI) m/z calcd. for $C_{20}H_{14}ClF_2$ $[M+H]^+$: 327.0747, found: 327.0740.

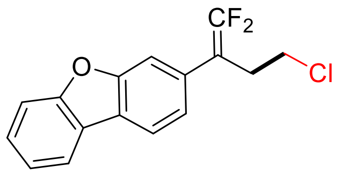

18

Purification by column chromatography on silica gel (hexane/ethyl acetate = 50/1, v/v) afforded **18** as a white solid (50.2 mg, 86% yield).

^{1}H NMR (400 MHz, Chloroform-*d*) δ 8.02 – 7.92 (m, 2H), 7.62 (d, *J* = 8.2 Hz, 1H), 7.54 – 7.48 (m, 1H), 7.43 – 7.34 (m, 3H), 3.52 (t, *J* = 7.0 Hz, 2H), 3.12 (tt, *J* = 6.9, 2.1 Hz, 2H).

^{13}C NMR (101 MHz, $CDCl_3$) δ 156.02, 154.60 (t, *J* = 293.2 Hz), 153.75, 128.15, 127.50, 124.78, 123.98, 123.02, 122.96, 120.77, 120.61, 116.69, 111.82, 85.73 (dd, *J* = 24.1, 17.4 Hz), 41.93, 30.96.

^{19}F NMR (376 MHz, Chloroform-*d*) δ -86.24 (d, *J* = 34.0 Hz, 1F), -89.38 (d, *J* = 34.0 Hz, 1F).

HRMS (ESI) m/z calcd. for $C_{16}H_{12}ClF_2O$ $[M+H]^+$: 293.0539, found: 293.0537.

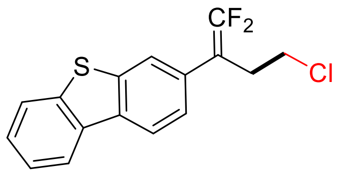

19

Purification by column chromatography on silica gel (hexane/ethyl acetate = 50/1, v/v) afforded **19** as a white solid (38.2 mg, 62% yield).

^{1}H NMR (400 MHz, Chloroform-*d*) δ 8.15 (d, *J* = 7.8 Hz, 2H), 7.91 – 7.82 (m, 1H), 7.52 – 7.44 (m, 3H), 7.38 (d, *J* = 7.2 Hz, 1H), 3.47 (t, *J* = 6.7 Hz, 2H), 3.00 (d, *J* = 7.1 Hz, 2H).

^{13}C NMR (101 MHz, $CDCl_3$) δ 154.34 (t, *J* = 293.1 Hz), 139.91, 139.87, 139.06, 136.21, 135.64, 127.84, 127.11, 124.86, 124.61, 122.79, 121.81, 121.48, 88.51 (dd, *J* = 22.9, 18.8 Hz), 41.70, 31.04.

^{19}F NMR (376 MHz, Chloroform-*d*) δ -84.74 (d, *J* = 33.4 Hz, 1F), -89.69 (d, *J* = 33.4 Hz, 1F).

HRMS (ESI) m/z calcd. for $C_{16}H_{12}ClF_2S$ $[M+H]^+$: 309.0311, found: 309.0305.

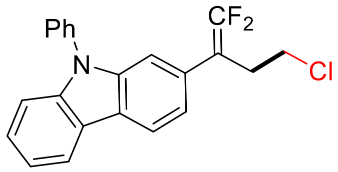

20

Purification by column chromatography on silica gel (hexane/ethyl acetate = 20/1, v/v) afforded **20** as a brown solid (51.4 mg, 70% yield).

^{1}H NMR (400 MHz, Chloroform-*d*) δ 8.17 (dd, *J* = 7.9, 2.9 Hz, 2H), 7.69 – 7.63 (m, 2H), 7.63 – 7.58 (m, 2H), 7.56 – 7.50 (m, 1H), 7.48 – 7.44 (m, 2H), 7.39 (s, 1H), 7.34 (ddd, *J* = 8.0, 5.3, 2.8 Hz, 1H), 7.26 (d, *J* = 8.1 Hz, 1H), 3.52 (t, *J* = 7.0 Hz, 2H), 2.94 (tt, *J* = 7.0, 2.0 Hz, 2H).

^{13}C NMR (101 MHz, $CDCl_3$) δ 154.46 (t, *J* = 291.0 Hz), 141.45, 140.99, 137.43, 130.05, 127.71, 127.13, 126.31, 123.01, 122.96, 120.51, 120.40, 120.24, 109.91, 109.86, 109.83, 109.79, 90.35 (dd, *J* = 21.3, 16.3 Hz)., 41.88, 31.85.

^{19}F NMR (376 MHz, Chloroform-*d*) δ -89.44 (d, *J* = 39.1 Hz, 1F), -90.13 (d, *J* = 39.1 Hz, 1F).

HRMS (ESI) m/z calcd. for $C_{22}H_{17}ClF_2N$ $[M+H]^+$: 368.1012, found: 368.1009.

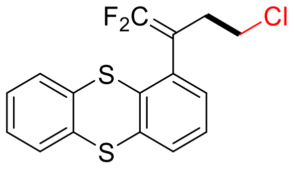

21

Purification by column chromatography on silica gel (hexane/ethyl acetate = 50/1, v/v) afforded **21** as a white solid (45.6 mg, 67% yield).

^{1}H NMR (400 MHz, Chloroform-*d*) δ 7.49 (dt, *J* = 7.7, 2.1 Hz, 3H), 7.26 – 7.21 (m, 3H), 7.19 (dd, *J* = 7.6, 1.5 Hz, 1H), 3.42 (t, *J* = 6.9 Hz, 2H), 2.92 – 2.81 (m, 2H).

^{13}C NMR (101 MHz, $CDCl_3$) δ 154.46 (dd, *J* = 292.5, 287.9 Hz), 136.72, 136.29, 136.08, 135.07, 132.33, 129.59, 128.93, 128.88, 128.67, 128.02, 127.87, 127.47, 88.19 (dd, *J* = 24.5, 19.4 Hz), 41.55, 31.82.

^{19}F NMR (376 MHz, Chloroform-*d*) δ -85.72 (d, *J* = 35.5 Hz, 1F), -90.33 (d, *J* = 35.8 Hz, 1F).

HRMS (ESI) m/z calcd. for $C_{16}H_{12}ClF_2S_2$ $[M+H]^+$: 341.0032, found: 341.0030.

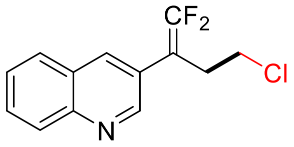

22

Purification by column chromatography on silica gel (hexane/ethyl acetate = 10/1, v/v) afforded **22** as a brown oil (36.4 mg, 72% yield).

^{1}H NMR (400 MHz, Chloroform-*d*) δ 8.88 (s, 1H), 8.11 (d, *J* = 9.4 Hz, 2H), 7.82 (d, *J* = 8.3 Hz, 1H), 7.77 – 7.70 (m, 1H), 7.58 (t, *J* = 7.5 Hz, 1H), 3.52 (t, *J* = 6.8 Hz, 2H), 2.98 (tt, *J* = 6.9, 2.1 Hz, 2H).

^{13}C NMR (101 MHz, $CDCl_3$) δ 155.06 (t, *J* = 293.4 Hz), 149.97 (t, *J* = 3.5 Hz), 147.21, 135.31, 130.00, 129.20, 127.78, 127.58, 127.27, 125.68 (t, *J* = 3.5 Hz), 87.23 (dd, *J* = 21.4, 14.7 Hz), 41.50, 30.93.

^{19}F NMR (376 MHz, Chloroform-*d*) δ -87.12 (d, *J* = 34.1 Hz, 1F), -87.54 (d, *J* = 34.1 Hz, 1F).

HRMS (ESI) m/z calcd. for $C_{13}H_{11}ClF_2N$ $[M+H]^+$: 254.0543, found: 254.0533.

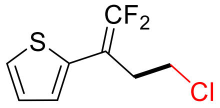

23

Purification by column chromatography on silica gel (hexane/ethyl acetate = 50/1, v/v) afforded **23** as a colorless oil (31.6 mg, 76% yield).

^{1}H NMR (400 MHz, Chloroform-*d*) δ 7.35 (d, *J* = 4.3 Hz, 1H), 7.25 – 7.20 (m, 1H), 7.20 – 7.11 (m, 1H), 3.57 (t, *J* = 7.1 Hz, 2H), 2.87 (t, *J* = 6.1 Hz, 2H).

^{13}C NMR (101 MHz, $CDCl_3$) δ 154.74 (dd, *J* = 294.2, 288.3 Hz), 132.46, 126.75 (d, *J* = 3.8 Hz), 126.07, 122.19 (t, *J* = 5.3 Hz), 85.83 (dd, *J* = 23.4, 15.0 Hz), 41.87, 30.93.

^{19}F NMR (376 MHz, Chloroform-*d*) δ -85.46 (d, *J* = 35.8 Hz, 1F), -89.67 (d, *J* = 35.7 Hz, 1F).

HRMS (ESI) m/z calcd. for $C_8H_8ClF_2S$ $[M+H]^+$: 208.9998, found: 208.9998.

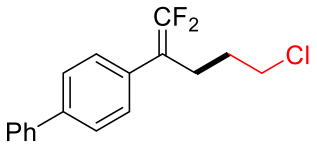

24

Purification by column chromatography on silica gel (hexane) afforded **24** as a white solid (35.6 mg, 61% yield).

^{1}H NMR (400 MHz, Chloroform-*d*) δ 7.67 (d, *J* = 7.2 Hz, 4H), 7.51 (t, *J* = 7.6 Hz, 2H), 7.45 (dd, *J* = 12.0, 7.8 Hz, 3H), 3.58 (t, *J* = 6.4 Hz, 2H), 2.73 – 2.64 (m, 2H), 1.94 (p, *J* = 6.6 Hz, 2H).

^{13}C NMR (101 MHz, $CDCl_3$) δ 153.92 (t, *J* = 289.8 Hz),, 140.48, 140.32, 132.03, 128.89, 128.56 (t, *J* = 3.3 Hz), 127.53, 127.30, 127.05, 91.01 (t, *J* = 17.5 Hz), 44.11, 30.66, 24.90.

^{19}F NMR (376 MHz, $CDCl_3$) δ -89.93, 2F.

HRMS (ESI) m/z calcd. for $C_{17}H_{16}ClF_2$ $[M+H]^+$: 293.0903, found: 293.0904.

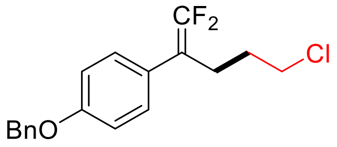

25

Purification by column chromatography on silica gel (hexane/ethyl acetate = 50/1, v/v) afforded **25** as a white solid (42.5 mg, 66% yield).

^{1}H NMR (400 MHz, Chloroform-*d*) δ 7.44 (dt, *J* = 14.6, 7.4 Hz, 4H), 7.36 (dd, *J* = 8.3, 5.8 Hz, 1H), 7.26 (d, *J* = 7.8 Hz, 2H), 7.02 – 6.97 (m, 2H), 5.09 (s, 2H), 3.52 (t, *J* = 6.5 Hz, 2H), 2.57 (ddd, *J* = 10.1, 4.6, 2.2 Hz, 2H), 1.86 (p, *J* = 6.6 Hz, 2H).

^{13}C NMR (101 MHz, $CDCl_3$) δ 158.10, 153.65 (dd, *J* = 289.9, 287.6 Hz), 136.87, 129.33 (t, *J* = 3.3 Hz), 128.63, 128.04, 127.48, 125.47, 114.97, 90.64 (dd, *J* = 20.9, 14.8 Hz), 70.08, 44.07, 30.59, 25.06.

^{19}F NMR (376 MHz, Chloroform-*d*) δ -91.37 (d, *J* = 44.1 Hz, 1F), -91.53 (d, *J* = 44.2 Hz, 1F).

HRMS (ESI) m/z calcd. for $C_{18}H_{18}ClF_2O$ $[M+H]^+$: 323.1009, found: 323.1001.

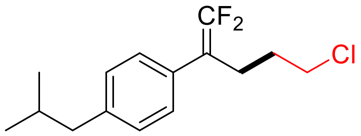

26

Purification by column chromatography on silica gel (hexane) afforded **26** as a colorless oil (29.9 mg, 55% yield).

^{1}H NMR (400 MHz, Chloroform-*d*) δ 7.23 (d, *J* = 7.6 Hz, 2H), 7.15 (d, *J* = 8.2 Hz, 2H), 3.52 (t, *J* = 6.5 Hz, 2H), 2.58 (ddt, *J* = 8.6, 4.7, 2.3 Hz, 2H), 2.48 (d, *J* = 7.2 Hz, 2H), 1.92 – 1.82 (m, 3H), 0.93 (d, *J* = 6.6 Hz, 6H).

^{13}C NMR (101 MHz, $CDCl_3$) δ 153.72 (dd, *J* = 290.7, 287.4 Hz), 141.04, 130.28, 129.29, 127.80 (t, *J* = 3.3 Hz), 91.02 (dd, *J* = 20.7, 14.2 Hz), 45.07, 44.07, 30.64, 30.12, 24.98, 22.36.

^{19}F NMR (376 MHz, Chloroform-*d*) δ -90.83 (d, *J* = 42.9 Hz, 1F), -91.02 (d, *J* = 42.9 H, 1Fz).

HRMS (ESI) m/z calcd. for $C_{15}H_{20}ClF_2$ $[M+H]^+$: 273.1216, found: 273.1213.

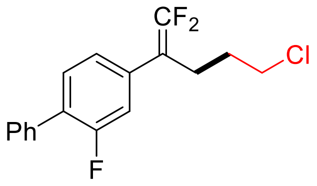

27

Purification by column chromatography on silica gel (hexane/ethyl acetate = 50/1, v/v) afforded **27** as a brown solid (26.0 mg, 42% yield).

^{1}H NMR (400 MHz, Chloroform-*d*) δ 7.61 – 7.54 (m, 2H), 7.46 (td, *J* = 8.1, 7.6, 5.3 Hz, 3H), 7.43 – 7.36 (m, 1H), 7.18 (dd, *J* = 16.4, 10.0 Hz, 2H), 3.56 (t, *J* = 6.4 Hz, 2H), 2.66 – 2.59 (m, 2H), 1.91 (dt, *J* = 13.4, 6.5 Hz, 2H).

^{13}C NMR (101 MHz, $CDCl_3$) δ 159.66 (d, *J* = 248.2 Hz), 154.06 (dd, *J* = 292.8, 289.0 Hz), 135.22, 134.08, 130.77 (d, *J* = 4.2 Hz), 128.92 (d, *J* = 3.0 Hz), 128.50, 128.11 (d, *J* = 13.7 Hz), 127.84, 124.00 (dd, *J* = 7.0, 3.5 Hz), 115.80 (dt, *J*=24.2, 3.8 Hz), 90.45 (dd, *J* = 22.6, 13.8 Hz), 43.94, 30.60, 24.71.

^{19}F NMR (376 MHz, Chloroform-*d*) δ -88.53 (d, *J* = 38.1 Hz, 1F), -88.68 (d, *J* = 38.1 Hz, 1F), -117.50, 1F.

HRMS (ESI) m/z calcd. for $C_{17}H_{15}ClF_3$ $[M+H]^+$: 311.0809, found: 311.0803.

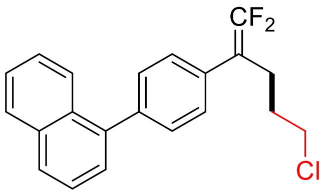

28

Purification by column chromatography on silica gel (hexane/ethyl acetate = 50/1, v/v) afforded **28** as a white solid (36.3 mg, 53% yield).

^{1}H NMR (400 MHz, Chloroform-*d*) δ 7.98 – 7.92 (m, 2H), 7.90 (d, *J* = 8.2 Hz, 1H), 7.58 – 7.51 (m, 4H), 7.51 – 7.44 (m, 4H), 3.60 (t, *J* = 6.5 Hz, 2H), 2.69 (ddd, *J* = 8.6, 5.9, 2.3 Hz, 2H), 1.97 (dt, *J* = 13.6, 6.6 Hz, 2H).

^{13}C NMR (101 MHz, $CDCl_3$) δ 153.96 (t, *J* = 289.6 Hz), 139.95, 139.59, 133.86, 132.03, 131.54, 130.27, 128.35, 128.00 (t, *J* = 3.4 Hz), 127.83, 126.96, 126.14, 125.89, 125.86, 125.39, 91.06 (dd, *J* = 19.0, 16.3 Hz), 44.12, 30.74, 25.01.

^{19}F NMR (376 MHz, Chloroform-*d*) δ (-89.93, 1F), (-89.94, 1F).

HRMS (ESI) m/z calcd. for $C_{21}H_{18}ClF_2$ $[M+H]^+$: 343.1060, found: 343.1058.

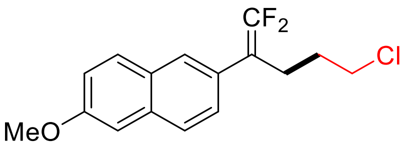

29

Purification by column chromatography on silica gel (hexane/ethyl acetate = 50/1, v/v) afforded **29** as a white solid (34.9 mg, 59% yield).

^{1}H NMR (400 MHz, Chloroform-*d*) δ 7.77 – 7.70 (m, 3H), 7.41 (d, *J* = 8.6 Hz, 1H), 7.21 – 7.11 (m, 2H), 3.94 (s, 3H), 3.53 (t, *J* = 6.5 Hz, 2H), 2.68 (tt, *J* = 8.4, 2.2 Hz, 2H), 1.89 (p, *J* = 6.6 Hz, 2H).

^{13}C NMR (101 MHz, $CDCl_3$) δ 158.04, 153.91 (dd, *J* = 290.4, 288.5 Hz), 133.72, 129.42, 128.77, 128.13, 127.09, 127.06, 126.42 (t, *J* = 3.3 Hz), 119.25, 105.62, 91.25 (dd, *J* = 20.0, 15.3 Hz), 55.34, 44.07, 30.63, 25.05.

^{19}F NMR (376 MHz, Chloroform-*d*) δ -90.57 (d, *J* = 42.3 Hz, 1F), -90.74 (d, *J* = 42.3 Hz, 1F).

HRMS (ESI) m/z calcd. for $C_{16}H_{16}ClF_2O$ $[M+H]^+$: 297.0852, found: 297.0847.

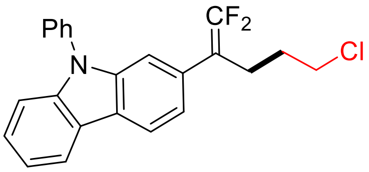

30

Purification by column chromatography on silica gel (hexane/ethyl acetate = 20/1, v/v) afforded **30** as a brown solid (38.1 mg, 50% yield).

^{1}H NMR (400 MHz, Chloroform-*d*) δ 8.15 (dd, *J* = 7.9, 4.7 Hz, 2H), 7.65 (t, *J* = 7.7 Hz, 2H), 7.61 – 7.57 (m, 2H), 7.54 – 7.49 (m, 1H), 7.45 – 7.42 (m, 2H), 7.37 (s, 1H), 7.32 (ddd, *J* = 8.0, 5.2, 2.9 Hz, 1H), 7.26 (d, *J* = 8.0 Hz, 1H), 3.51 (t, *J* = 6.4 Hz, 2H), 2.65 (dd, *J* = 8.6, 4.0 Hz, 2H), 1.87 (p, *J* = 6.6 Hz, 2H).

^{13}C NMR (101 MHz, $CDCl_3$) δ 153.77 (dd, *J* = 290.0, 287.9 Hz), 141.42, 141.01, 137.47, 130.87, 130.00, 127.64, 127.14, 126.19, 123.01, 122.75, 120.36, 120.33, 120.21, 120.17, 109.86, 109.53 (t, *J* = 3.3 Hz), 91.96 (dd, *J* = 21.4, 14.0 Hz), 44.13, 30.65, 25.43.

^{19}F NMR (376 MHz, Chloroform-*d*) δ -90.65 (d, *J* = 42.3 Hz, 1F), -90.92 (d, *J* = 42.3 Hz, 1F).

HRMS (ESI) m/z calcd. for $C_{23}H_{19}ClF_2N$ $[M+H]^+$: 382.1169, found: 382.1159.

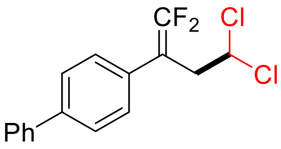

31

Purification by column chromatography on silica gel (hexane) afforded **31** as a white solid (46.8 mg, 75% yield).

^{1}H NMR (400 MHz, Chloroform-*d*) δ 7.63 (t, *J* = 7.8 Hz, 4H), 7.48 (t, *J* = 7.5 Hz, 2H), 7.40 (d, *J* = 8.1 Hz, 3H), 5.60 (t, *J* = 6.7 Hz, 1H), 3.35 (d, *J* = 6.8 Hz, 2H).

^{13}C NMR (101 MHz, $CDCl_3$) δ 155.20 (t, *J* = 291.9 Hz), 141.04, 140.27, 130.38, 128.89, 128.73 (t, *J* = 3.0 Hz), 127.66, 127.57, 127.06, 88.66 (dd, *J* = 20.5, 18.3 Hz), 70.43 (t, *J* = 4.5 Hz), 42.24.

^{19}F NMR (376 MHz, Chloroform-*d*) δ -87.21 (d, *J* = 32.7 Hz, 1F), -88.04 (d, *J* = 32.6 Hz, 1F).

HRMS (ESI) m/z calcd. for $C_{16}H_{13}Cl_2F_2$ $[M+H]^+$: 313.0357, found: 313.0355.

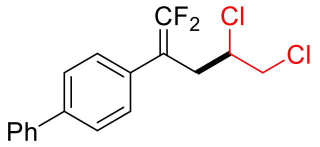

32

Purification by column chromatography on silica gel (hexane) afforded **32** as a white solid (33.2 mg, 51% yield).

^{1}H NMR (400 MHz, Chloroform-*d*) δ 7.64 (t, *J* = 7.0 Hz, 4H), 7.45 (ddd, *J* = 21.9, 14.5, 7.4 Hz, 5H), 4.08 – 3.95 (m, 1H), 3.79 (dd, *J* = 11.5, 4.8 Hz, 1H), 3.70 (dd, *J* = 11.4, 7.3 Hz, 1H), 3.20 (dq, *J* = 15.0, 3.3 Hz, 1H), 2.89 (dd, *J* = 15.0, 9.3 Hz, 1H).

^{13}C NMR (101 MHz, $CDCl_3$) δ 154.78 (t, *J* = 290.6 Hz), 140.78, 140.35, 130.91, 128.87, 128.73 (t, *J*=3.1 Hz), 127.59, 127.47, 127.05, 88.84 (t, *J* = 18.9 Hz), 58.00, 47.83, 33.89.

^{19}F NMR (376 MHz, Chloroform-*d*) δ -88.48 (d, *J* = 2.8 Hz， 2F).

HRMS (ESI) m/z calcd. for $C_{17}H_{15}Cl_2F_2$ $[M+H]^+$: 327.0513, found: 327.0510.

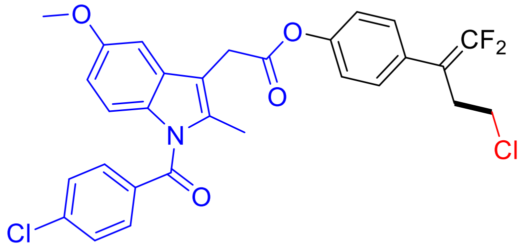

33

Purification by column chromatography on silica gel (hexane/ethyl acetate = 2/1, v/v) afforded **33** as a white solid (72.4 mg, 65% yield).

^{1}H NMR (400 MHz, Chloroform-*d*) δ 7.69 (d, *J* = 8.6 Hz, 2H), 7.48 (d, *J* = 8.6 Hz, 2H), 7.31 (d, *J* = 8.1 Hz, 2H), 7.13 – 7.04 (m, 3H), 6.91 (d, *J* = 9.0 Hz, 1H), 6.71 (dd, *J* = 9.0, 2.5 Hz, 1H), 3.92 (s, 2H), 3.85 (s, 3H), 3.46 (t, *J* = 7.0 Hz, 2H), 2.84 (ddt, *J* = 7.0, 4.9, 2.1 Hz, 2H), 2.47 (s, 3H).

^{13}C NMR (101 MHz, $CDCl_3$) δ 169.12, 168.28, 156.18, 154.51 (dd, *J* = 292.3, 290.5 Hz), 150.11, 139.38, 136.25, 133.84, 131.19, 130.90, 130.47, 130.04, 129.44 (t, *J* = 3.1 Hz), 129.15, 121.93, 121.69, 115.02, 111.85, 111.80, 101.30, 88.89 (dd, *J* = 21.7, 15.9 Hz), 55.74, 41.52, 31.26, 30.56, 13.37.

^{19}F NMR (376 MHz, Chloroform-*d*) δ -88.86 (d, *J* = 37.4 Hz, 1F), -89.26 (d, *J* = 37.4 Hz, 1F).

HRMS (ESI) m/z calcd. for $C_{29}H_{24}Cl_2F_2NO_4$ $[M+H]^+$: 558.1050, found: 558.1053.

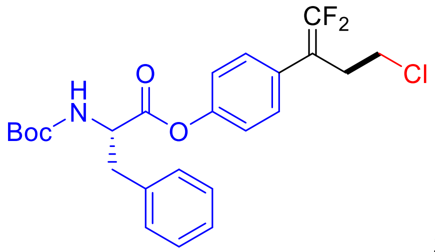

34

Purification by column chromatography on silica gel (hexane/ethyl acetate = 5/1, v/v) afforded **34** as a white solid (55.8 mg, 60% yield)

^{1}H NMR (400 MHz, Chloroform-*d*) δ 7.34 (dd, *J* = 15.0, 7.6 Hz, 5H), 7.26 (d, *J* = 8.6 Hz, 2H), 7.02 (d, *J* = 8.5 Hz, 2H), 5.08 (d, *J* = 7.7 Hz, 1H), 4.83 (q, *J* = 7.4, 6.9 Hz, 1H), 3.48 (t, *J* = 7.0 Hz, 2H), 3.25 (d, *J* = 6.0 Hz, 2H), 2.85 (tt, *J* = 7.1, 2.2 Hz, 2H), 1.46 (s, 9H).

^{13}C NMR (101 MHz, $CDCl_3$) δ 170.50, 154.54 (dd, *J* = 291.5, 289.5 Hz), 149.75, 135.68, 130.19, 129.47, 129.44, 128.75, 127.31, 121.61, 88.88 (dd, *J* = 22.2, 15.6 Hz), 80.21, 54.69, 41.55, 38.34, 31.26, 28.30.

^{19}F NMR (376 MHz, Chloroform-*d*) δ -88.77 (d, *J* = 37.4 Hz, 1F), -89.19 (d, *J* = 37.4 Hz, 1F).

HRMS (ESI) m/z calcd. for $C_{24}H_{27}ClF_2NO_4$ $[M+H]^+$: 466.1591, found: 466.1591.

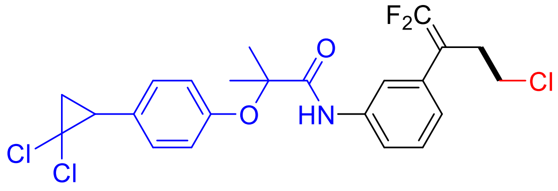

35

Purification by column chromatography on silica gel (hexane/ethyl acetate = 10/1, v/v) afforded **35** as a white solid (70.1 mg, 75% yield)

^{1}H NMR (400 MHz, Chloroform-*d*) δ 8.63 (s, 1H), 7.63 (s, 1H), 7.52 – 7.48 (m, 1H), 7.36 (t, *J* = 7.9 Hz, 1H), 7.21 (d, *J* = 8.5 Hz, 2H), 7.10 (d, *J* = 7.1 Hz, 1H), 7.00 (d, *J* = 8.6 Hz, 2H), 3.49 (t, *J* = 7.0 Hz, 2H), 2.91 – 2.84 (m, 3H), 1.99 (dd, *J* = 10.7, 7.5 Hz, 1H), 1.82 (t, *J* = 7.9 Hz, 1H), 1.60 (s, 6H).

^{13}C NMR (101 MHz, $CDCl_3$) δ 172.97, 154.51 (dd, *J* = 293.4, 290.0 Hz), 153.28, 137.87, 133.30, 130.32, 129.91, 129.33, 124.52 (t, *J* = 3.0 Hz), 121.73, 119.69 (t, *J* = 3.3 Hz), 119.22, 89.39 (dd, *J* = 21.7, 15.8 Hz), 82.17, 60.69, 41.66, 34.83, 31.24, 25.91, 24.98, 24.96.

^{19}F NMR (376 MHz, Chloroform-*d*) δ -88.60 (d, *J* = 37.5 Hz, 1F), -89.39 (d, *J* = 37.5 Hz, 1F).

HRMS (ESI) m/z calcd. for $C_{23}H_{23}Cl_3F_2NO_2$ $[M+H]^+$: 488.0757, found: 488.0761.

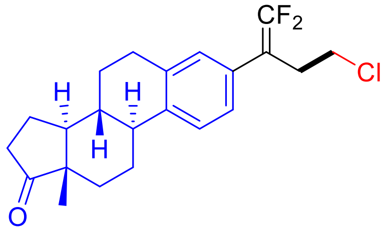

36

Purification by column chromatography on silica gel (hexane/ethyl acetate = 10/1, v/v) afforded **36** as a white solid (43.8 mg, 58% yield).

^{1}H NMR (400 MHz, Chloroform-*d*) δ 7.29 (d, *J* = 7.9 Hz, 1H), 7.11 – 7.01 (m, 2H), 3.47 (t, *J* = 7.0 Hz, 2H), 2.96 – 2.89 (m, 2H), 2.84 (d, *J* = 6.7 Hz, 2H), 2.51 (dd, *J* = 18.7, 8.6 Hz, 1H), 2.41 (t, *J* = 12.6 Hz, 1H), 2.31 (d, *J* = 9.7 Hz, 1H), 2.20 – 2.12 (m, 1H), 2.06 (t, *J* = 9.8 Hz, 2H), 1.97 (d, *J* = 10.8 Hz, 1H), 1.62 (q, *J* = 9.6 Hz, 2H), 1.57 – 1.44 (m, 4H), 0.92 (s, 3H).

^{13}C NMR (101 MHz, $CDCl_3$) δ 154.39 (t, J = 289.7 Hz), 139.44, 136.86, 129.67, 128.79, 125.66, 125.62, 89.25 (dd, J = 20.9, 16.4 Hz), 50.50, 47.96, 44.34, 41.79, 38.03, 35.84, 31.57, 31.26, 29.38, 26.42, 25.60, 21.57, 13.83.

^{19}F NMR (376 MHz, Chloroform-*d*) δ -89.46 (d, J = 39.3 Hz, 1F), -89.97 (d, J = 39.3 Hz, 1F).

HRMS (ESI) m/z calcd. for $C_{22}H_{26}ClF_2O$ $[M+H]^+$: 379.1635, found: 379.1635.

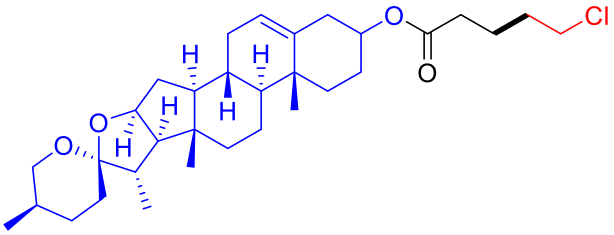

37

Purification by column chromatography on silica gel (hexane/ethyl acetate = 20/1, v/v) afforded **37** as a white solid (26.6 mg, 25% yield).

^{1}H NMR (400 MHz, Chloroform-*d*) δ 5.37 (d, J = 5.0 Hz, 1H), 4.67 – 4.55 (m, 1H), 4.41 (td, J = 8.0, 6.5 Hz, 1H), 3.55 (t, J = 6.1 Hz, 2H), 3.47 (ddd, J = 11.0, 4.6, 2.0 Hz, 1H), 3.37 (t, J = 10.9 Hz, 1H), 2.36 – 2.25 (m, 4H), 2.00 (dtd, J = 17.1, 8.2, 7.4, 5.1 Hz, 2H), 1.89 – 1.74 (m, 8H), 1.69 – 1.40 (m, 10H), 1.33 – 1.24 (m, 1H), 1.22 – 1.07 (m, 3H), 1.04 (s, 3H), 0.97 (d, J = 7.0 Hz, 4H), 0.79 (t, J = 3.1 Hz, 6H).

^{13}C NMR (101 MHz, $CDCl_3$) δ 172.51, 139.64, 122.38, 109.23, 80.78, 73.88, 66.82, 62.11, 56.43, 49.95, 44.43, 41.61, 40.25, 39.72, 38.11, 36.94, 36.72, 33.75, 32.03, 31.85, 31.40, 30.29, 28.80, 27.77, 22.33, 20.81, 19.32, 17.11, 16.26, 14.50.

HRMS (ESI) m/z calcd. for $C_{32}H_{50}ClO_4$ $[M+H]^+$: 533.3392, found: 533.3390.

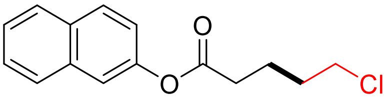

38

Purification by column chromatography on silica gel (hexane/ethyl acetate = 20/1, v/v) afforded **38** as a white solid (22.5 mg, 43% yield).

^{1}H NMR (400 MHz, Chloroform-*d*) δ = 7.85 (ddd, J = 16.4, 9.0, 6.0, 3H), 7.58 (d, J = 2.2, 1H), 7.54 – 7.45 (m, 2H), 7.24 (dd, J = 8.9, 2.3, 1H), 3.64 (t, J = 6.2, 2H), 2.69 (t, J = 7.1, 2H), 2.00 – 1.93 (m, 4H).

^{13}C NMR (101 MHz, $CDCl_3$) δ = 171.77, 148.30, 133.77, 131.47, 129.41, 127.77, 127.63, 126.58, 125.71, 121.07, 118.46, 44.39, 33.58, 31.83, 22.28.

HRMS (ESI) m/z calcd. for $C_{15}H_{16}ClO_2$ $[M+H]^+$: 263.0833, found: 263.0833.

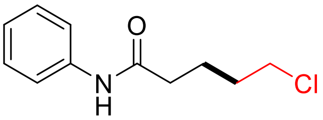

39

Purification by column chromatography on silica gel (hexane/ethyl acetate = 10/1, v/v) afforded **39** as a white solid (21.5 mg, 51% yield).

^{1}H NMR (400 MHz, Chloroform-*d*) δ = 7.52 (d, *J* = 7.9, 2H), 7.47 (s, 1H), 7.32 (t, *J* = 7.8, 2H), 7.11 (t, *J*=7.4, 1H), 3.57 (t, *J*=5.9, 2H), 2.40 (dd, *J*=8.6, 4.8, 2H), 1.92 – 1.81 (m, 4H).

^{13}C NMR (101 MHz, $CDCl_3$) δ = 170.69, 137.81, 128.98, 124.33, 119.92, 44.55, 36.62, 31.89, 22.81.

HRMS (ESI) m/z calcd. for $C_{11}H_{15}ClNO$ $[M+H]^+$: 212.0837, found: 212.0847.

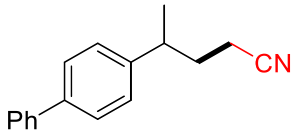

40

Purification by column chromatography on silica gel (hexane/ethyl acetate = 100/1, v/v) afforded **40** as a white solid (21.2 mg, 45% yield).

1H NMR (400 MHz, Chloroform-d) δ 7.58 (ddd, J = 10.4, 7.6, 1.6 Hz, 4H), 7.48 – 7.41 (m, 2H), 7.39 – 7.33 (m, 1H), 7.28 (d, J = 7.9 Hz, 2H), 2.92 (ddd, J = 13.7, 11.1, 6.5 Hz, 1H), 2.28 (ddd, J = 16.8, 7.6, 5.8 Hz, 1H), 2.22 – 2.10 (m, 1H), 2.07 – 1.87 (m, 2H), 1.36 (d, J = 6.9 Hz, 3H).

^{13}C NMR (101 MHz, $CDCl_3$) δ 143.63, 140.75, 139.75, 128.76, 127.49, 127.33, 127.22, 126.98, 119.58, 38.60, 33.56, 21.91, 15.50.

HRMS (ESI) m/z calcd. for $C_{17}H_{18}N$ $[M+H]^+$: 236.1434, found: 236.1435.

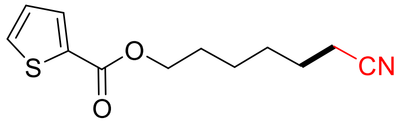

41

Purification by column chromatography on silica gel (hexane/ethyl acetate = 20/1, v/v) afforded **41** as a colorless oil (22.8 mg, 48% yield).

^{1}H NMR (600 MHz, Chloroform-*d*) δ 7.80 (dd, *J* = 3.8, 1.4 Hz, 1H), 7.56 (dd, *J* = 5.0, 1.3 Hz, 1H), 7.11 (dd, *J* = 5.0, 3.7 Hz, 1H), 4.30 (t, *J* = 6.5 Hz, 2H), 2.36 (t, *J* = 7.1 Hz, 2H), 1.80 – 1.75 (m, 2H), 1.69 (p, *J* = 7.2 Hz, 2H), 1.56 – 1.44 (m, 4H).

^{13}C NMR (151 MHz, $CDCl_3$) δ 162.27, 133.88, 133.36, 132.33, 127.78, 119.68, 64.85, 28.42, 28.31, 25.28, 17.08.

HRMS (ESI) m/z calcd. for $C_{12}H_{16}NO_2S$ $[M+H]^+$: 238.0896, found: 238.0897.

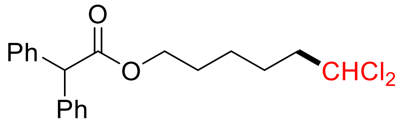

42

Purification by column chromatography on silica gel (hexane/ethyl acetate = 20/1, v/v) afforded **42** as a colorless oil (35.7 mg, 49% yield).

^{1}H NMR (600 MHz, Chloroform-*d*) δ 7.31 (d, *J* = 3.8 Hz, 8H), 7.26 (td, *J* = 5.1, 3.1 Hz, 2H), 5.66 (t, *J* = 6.0 Hz, 1H), 5.02 (s, 1H), 4.15 (t, *J* = 6.5 Hz, 2H), 2.10 (dt, *J* = 8.1, 6.0 Hz, 2H), 1.66 – 1.60 (m, 2H), 1.52 – 1.45 (m, 2H), 1.31 – 1.25 (m, 2H).

^{13}C NMR (151 MHz, Chloroform-*d*) δ 172.53, 138.70, 128.64, 128.62, 127.31, 73.40, 64.82, 57.23, 43.38, 28.29, 25.46, 24.86.

HRMS (ESI) m/z calcd. for $C_{20}H_{23}Cl_2O_2$ $[M+H]^+$: 365.1070, found: 365.1073.

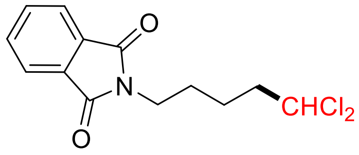

43

Purification by column chromatography on silica gel (hexane/ethyl acetate = 10/1, v/v) afforded **43** as a colorless oil (24.5 mg, 43% yield).

^{1}H NMR (600 MHz, Chloroform-*d*) δ 7.77 (dd, *J* = 5.5, 3.1 Hz, 2H), 7.65 (dd, *J* = 5.5, 3.1 Hz, 2H), 5.67 (t, *J* = 6.0 Hz, 1H), 3.64 (t, *J* = 7.2 Hz, 2H), 2.21 – 2.15 (m, 2H), 1.70 – 1.64 (m, 2H), 1.58 – 1.50 (m, 2H).

^{13}C NMR (151 MHz, $CDCl_3$) δ 168.36, 133.99, 132.07, 123.27, 73.15, 42.92, 37.47, 27.54, 23.18.

HRMS (ESI) m/z calcd. for $C_{13}H_{14}Cl_2NO_2$ $[M+H]^+$: 286.0396, found: 286.0395.

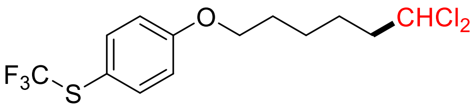

44

Purification by column chromatography on silica gel (hexane/ethyl acetate = 20/1, v/v) afforded **44** as a colorless oil (36.7 mg, 53% yield).

^{1}H NMR (600 MHz, Chloroform-*d*) δ 7.56 (d, *J* = 8.7 Hz, 2H), 6.91 (d, *J* = 8.8 Hz, 2H), 5.77 (t, *J* = 6.0 Hz, 1H), 3.98 (t, *J* = 6.3 Hz, 2H), 2.23 (dd, *J* = 15.5, 6.0 Hz, 2H), 1.87 – 1.79 (m, 2H), 1.64 (p, *J* = 7.4 Hz, 2H), 1.54 (q, *J* = 8.4 Hz, 2H).

^{13}C NMR (151 MHz, Chloroform-*d*) δ 161.29, 138.30, 129.64 (q, *J* = 308.1 Hz), 115.45, 73.43, 67.80, 43.43, 28.87, 25.63, 25.12.

^{19}F NMR (565 MHz, Chloroform-*d*) δ -43.92, 3F.

HRMS (ESI) m/z calcd. for $C_{13}H_{16}Cl_2F_3OS$ $[M+H]^+$: 347.0246, found: 347.0245.

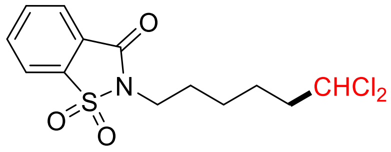

45

Purification by column chromatography on silica gel (hexane/ethyl acetate = 10/1, v/v) afforded **45** as a colorless oil (31.5 mg, 47% yield).

^{1}H NMR (600 MHz, Chloroform-*d*) δ 8.06 (d, *J* = 7.1 Hz, 1H), 7.92 (d, *J* = 7.4 Hz, 1H), 7.89 – 7.85 (m, 1H), 7.85 – 7.82 (m, 1H), 5.75 (t, *J* = 6.0 Hz, 1H), 3.79 (t, *J* = 7.3 Hz, 2H), 2.24 – 2.19 (m, 2H), 1.89 (p, *J* = 7.5 Hz, 2H), 1.63 (ddt, *J* = 11.1, 7.8, 3.8 Hz, 2H), 1.48 (p, *J* = 7.8 Hz, 2H).

^{13}C NMR (151 MHz, $CDCl_3$) δ 159.01, 137.68, 134.77, 134.36, 127.38, 125.17, 120.94, 73.36, 43.30, 39.12, 28.14, 25.73, 25.35.

HRMS (ESI) m/z calcd. for $C_{13}H_{16}Cl_2NO_3S$ $[M+H]^+$: 336.0222, found: 336.0222.

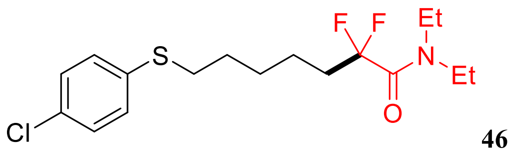

46

Purification by column chromatography on silica gel (hexane/ethyl acetate = 7/1, v/v) afforded **46** as a colorless oil (50.1 mg, 69% yield).

^{1}H NMR (600 MHz, Chloroform-*d*) δ 7.16 (s, 4H), 3.43 (q, J = 7.1 Hz, 2H), 3.29 (q, J = 7.1 Hz, 2H), 2.82 (t, J = 7.3 Hz, 2H), 2.12 – 1.97 (m, 2H), 1.58 (p, J = 7.4 Hz, 2H), 1.51 – 1.37 (m, 4H), 1.12 (t, J = 7.0 Hz, 3H), 1.07 (t, J = 7.1 Hz, 3H).

^{13}C NMR (151 MHz, Chloroform-*d*) δ 162.82 (t, J = 29.4 Hz), 135.34, 131.71, 130.34, 128.96, 119.59 (t, J = 254.2 Hz), 41.83 (t, J = 6.4 Hz), 41.45, 34.67 (t, J = 23.4 Hz), 33.65, 28.76, 28.41, 21.17 (t, J = 4.6 Hz), 14.30, 12.33.

^{19}F NMR (376 MHz, Chloroform-*d*) δ -100.28, 2F.

HRMS (ESI) m/z calcd. for $C_{17}H_{25}ClF_2NOS$ $[M+H]^+$: 364.1308, found: 364.1310.

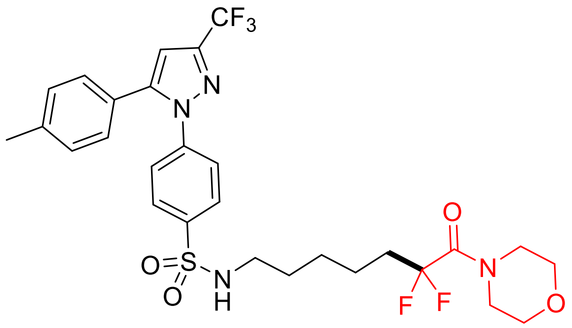

47

Purification by column chromatography on silica gel (hexane/ethyl acetate = 2/1, v/v) afforded **47** as a yellow oil (74.9 mg, 61% yield).

^{1}H NMR (600 MHz, Chloroform-*d*) δ 7.84 (d, J = 8.6 Hz, 2H), 7.47 (d, J = 8.6 Hz, 2H), 7.17 (d, J = 7.9 Hz, 2H), 7.11 (d, J = 8.1 Hz, 2H), 6.75 (s, 1H), 4.93 – 4.81 (m, 1H), 3.72 (q, J = 5.0 Hz, 4H), 3.70 – 3.67 (m, 2H), 3.63 (t, J = 4.8 Hz, 2H), 2.95 (q, J = 6.7 Hz, 2H), 2.38 (s, 3H), 2.15 – 2.05 (m, 2H), 1.50 (qq, J = 7.9, 5.5, 4.4 Hz, 4H), 1.36 (ddd, J = 15.2, 8.7, 6.1 Hz, 2H).

^{13}C NMR (151 MHz, Chloroform-*d*) δ 162.01 (t, J = 29.9 Hz), 145.27, 144.06 (q, J = 38.4 Hz), 142.46, 139.81, 139.54, 129.75, 128.72, 128.06, 125.69, 125.57, 121.07 (q, J = 269.2 Hz), 119.43 (t, J = 254.1 Hz), 106.27, 66.79, 66.71, 46.53 (t, J = 6.3 Hz), 43.34, 42.99, 34.33 (t, J = 22.9 Hz), 29.25, 26.14, 21.31, 21.01 (t, J = 4.6 Hz).

^{19}F NMR (565 MHz, $CDCl_3$) δ -62.42, 3F, -99.26, 2F.

HRMS (ESI) m/z calcd. for $C_{28}H_{32}F_5N_4O_4S$ $[M+H]^+$: 615.2059, found: 615.2059.

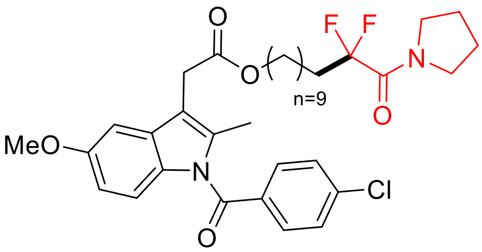

48

Purification by column chromatography on silica gel (hexane/ethyl acetate = 4/1, v/v) afforded **48** as a yellow oil (72.2 mg, 56% yield).

^{1}H NMR (600 MHz, Chloroform-*d*) δ 7.66 (d, *J* = 8.5 Hz, 2H), 7.47 (d, *J* = 8.5 Hz, 2H), 6.97 (d, *J* = 2.5 Hz, 1H), 6.87 (d, *J* = 9.0 Hz, 1H), 6.67 (dd, *J* = 9.0, 2.6 Hz, 1H), 4.09 (t, *J* = 6.7 Hz, 2H), 3.83 (s, 3H), 3.68 (t, *J* = 6.9 Hz, 2H), 3.65 (s, 2H), 3.52 (t, *J* = 7.1 Hz, 2H), 2.38 (s, 3H), 2.15 – 2.05 (m, 2H), 1.95 (p, *J* = 6.8 Hz, 2H), 1.85 (p, *J* = 6.9 Hz, 2H), 1.61 (p, *J* = 6.8 Hz, 2H), 1.50 (qd, *J* = 8.8, 8.0, 6.2 Hz, 2H), 1.37 – 1.31 (m, 2H), 1.30 – 1.21 (m, 10H).

^{13}C NMR (151 MHz, Chloroform-*d*) δ 170.95, 168.29, 162.48 (t, *J* = 30.3 Hz), 156.06, 139.24, 135.86, 133.98, 131.17, 130.83, 130.70, 129.11, 119.24 (t, *J* = 252.4 Hz), 114.93, 112.78, 111.68, 101.35, 65.18, 55.69, 47.33, 46.59 (t, *J* = 6.5 Hz), 34.28 (t, *J* = 23.2 Hz), 30.44, 29.43, 29.33, 29.29, 29.18, 28.62, 26.53, 25.87, 23.30, 21.46 (t, *J* = 4.3 Hz), 13.35.

^{19}F NMR (565 MHz, Chloroform-*d*) δ -103.26, 2F.

HRMS (ESI) m/z calcd. for $C_{35}H_{44}ClF_2N_2O_5$ $[M+H]^+$: 645.2901, found: 645.2904.

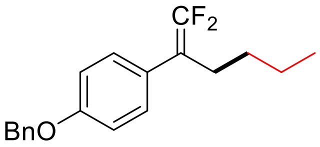

49

Purification by column chromatography on silica gel (hexane/ethyl acetate = 50/1, v/v) afforded **49** as a white solid (33.2 mg, 55% yield).

^{1}H NMR (400 MHz, Chloroform-*d*) δ 7.47 (d, *J* = 7.2 Hz, 2H), 7.43 (t, *J* = 7.3 Hz, 2H), 7.37 (dd, *J* = 8.3, 5.8 Hz, 1H), 7.27 (d, *J* = 8.8 Hz, 2H), 7.02 – 6.98 (m, 2H), 5.10 (s, 2H), 2.44 – 2.34 (m, 2H), 1.36 (h, *J* = 6.4 Hz, 4H), 0.91 (t, *J* = 7.0 Hz, 3H).

^{13}C NMR (101 MHz, $CDCl_3$) δ 157.88, 153.48 (dd, *J* = 288.2, 286.4 Hz), 136.97, 129.37 (t, *J* = 3.3 Hz), 128.60, 127.99, 127.48, 126.33, 114.77, 91.86 (dd, *J* = 19.7, 15.1 Hz), 70.06, 29.89 (t, *J* = 2.5 Hz), 27.43, 22.11, 13.75.

^{19}F NMR (376 MHz, Chloroform-*d*) δ -92.86, 2F.

HRMS (ESI) m/z calcd. for $C_{19}H_{21}F_2O$ $[M+H]^+$: 303.1555, found: 303.1552.

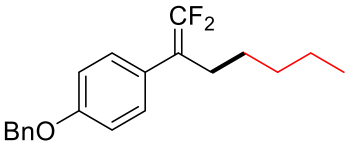

50

Purification by column chromatography on silica gel (hexane/ethyl acetate = 50/1, v/v) afforded **50** as a white solid (39.8 mg, 63% yield).

^{1}H NMR (400 MHz, Chloroform-*d*) δ 7.49 – 7.39 (m, 4H), 7.36 (t, *J* = 7.1 Hz, 1H), 7.26 (d, *J* = 8.7 Hz, 2H), 6.99 (d, *J* = 8.8 Hz, 2H), 5.09 (s, 2H), 2.37 (dd, *J* = 10.0, 4.7 Hz, 2H), 1.38 (p, *J* = 7.0, 6.4 Hz, 2H), 1.32 – 1.26 (m, 4H), 0.89 (t, *J* = 6.7 Hz, 3H).

^{13}C NMR (101 MHz, $CDCl_3$) δ 157.87, 153.48 (dd, *J* = 288.0, 286.8 Hz), 136.97, 129.37 (t, *J* = 3.3 Hz), 128.60, 127.99, 127.48, 126.34, 114.77, 91.89 (dd, *J* = 19.7, 15.3 Hz), 70.06, 31.21, 27.68, 27.39, 22.34, 13.97.

^{19}F NMR (376 MHz, Chloroform-*d*) δ -92.87 (d, *J* = 1.5 Hz, 2F).

HRMS (ESI) m/z calcd. for $C_{20}H_{23}F_2O$ $[M+H]^+$:317.1711, found: 317.1709.

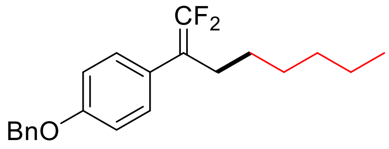

51

Purification by column chromatography on silica gel (hexane/ethyl acetate = 50/1, v/v) afforded **51** as a white solid (40.3 mg, 61% yield).

^{1}H NMR (400 MHz, Chloroform-*d*) δ 7.49 – 7.39 (m, 4H), 7.36 (dd, *J* = 8.3, 5.8 Hz, 1H), 7.26 (d, *J* = 8.8 Hz, 2H), 6.99 (d, *J* = 8.8 Hz, 2H), 5.09 (s, 2H), 2.37 (dd, *J* = 9.8, 4.7 Hz, 2H), 1.41 – 1.21 (m, 8H), 0.89 (t, *J* = 6.8 Hz, 3H).

^{13}C NMR (101 MHz, $CDCl_3$) δ 157.89, 153.49(t, *J* = 288.9 Hz), 136.99, 129.38 (t, *J* = 3.3 Hz), 128.61, 128.00, 127.49, 126.35, 114.78, 91.91 (dd, *J* = 19.4, 15.6 Hz), 70.06, 31.52, 28.69, 27.73, 27.70, 22.58, 14.01.

^{19}F NMR (376 MHz, Chloroform-*d*) δ -92.86, 2F.

HRMS (ESI) m/z calcd. for $C_{21}H_{25}F_2O$ $[M+H]^+$: 331.1868, found: 331.1868.

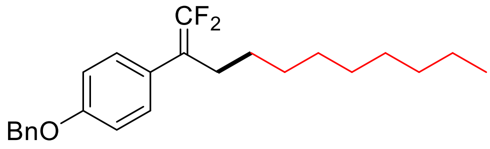

52

Purification by column chromatography on silica gel (hexane/ethyl acetate = 50/1, v/v) afforded **52** as a white solid (41.7 mg, 56% yield).

^{1}H NMR (400 MHz, Chloroform-*d*) δ 7.48 – 7.39 (m, 4H), 7.37 (d, *J* = 7.1 Hz, 1H), 7.28 – 7.23 (m, 2H), 6.99 (d, *J* = 8.8 Hz, 2H), 5.09 (s, 2H), 2.44 – 2.29 (m, 2H), 1.45 – 1.16 (m, 14H), 0.91 (t, *J* = 6.9 Hz, 3H).

^{13}C NMR (101 MHz, $CDCl_3$) δ 157.88, 153.49 (t, *J* = 288.0 Hz), 136.98, 129.38 (t, *J* = 3.3 Hz), 128.60, 127.99, 127.48, 126.36, 114.78, 91.91 (dd, *J* = 19.3, 15.6 Hz), 70.07, 31.87, 29.51, 29.30, 29.26, 29.02, 27.72, 22.66, 14.08.

^{19}F NMR (376 MHz, Chloroform-*d*) δ -92.85, 2F.

HRMS (ESI) m/z calcd. for $C_{24}H_{31}F_2O$ $[M+H]^+$: 373.2337, found: 373.2343.

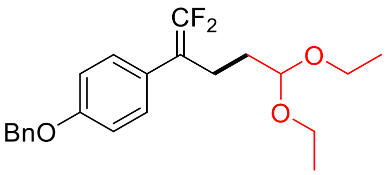

53

Purification by column chromatography on silica gel (hexane/ethyl acetate = 50/1, v/v) afforded **53** as a white solid (52.6 mg, 70% yield).

^{1}H NMR (400 MHz, Chloroform-*d*) δ 7.43 (dt, *J* = 14.8, 7.5 Hz, 4H), 7.35 (t, *J* = 7.0 Hz, 1H), 7.26 (d, *J* = 8.6 Hz, 2H), 6.99 (d, *J* = 8.7 Hz, 2H), 5.08 (s, 2H), 4.48 (t, *J* = 5.7 Hz, 1H), 3.67 – 3.58 (m, 2H), 3.50 – 3.41 (m, 2H), 2.47 (dd, *J* = 10.3, 5.0 Hz, 2H), 1.74 – 1.67 (m, 2H), 1.21 (t, *J* = 7.0 Hz, 6H).

^{13}C NMR (101 MHz, $CDCl_3$) δ 157.98, 153.37 (t, *J* = 287.9 Hz), 136.94, 129.39 (t, *J* = 3.3 Hz), 128.60, 127.99, 127.45, 125.92, 114.88, 102.11, 91.36 (dd, *J* = 19.3, 16.1 Hz), 70.05, 61.15, 31.75, 23.11, 15.28.

^{19}F NMR (376 MHz, Chloroform-*d*) δ -92.21, 2F.

HRMS (ESI) m/z calcd. for $C_{22}H_{27}F_2O_3$ $[M+H]^+$: 377.1923, found: 377.1920.

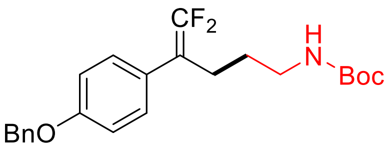

54

Purification by column chromatography on silica gel (hexane/ethyl acetate = 20/1, v/v) afforded **54** as a white solid (45.1 mg, 56% yield).

^{1}H NMR (400 MHz, Chloroform-*d*) δ 7.46 – 7.39 (m, 4H), 7.35 (dd, *J* = 8.3, 5.8 Hz, 1H), 7.24 (d, *J* = 8.5 Hz, 2H), 6.98 (d, *J* = 8.8 Hz, 2H), 5.08 (s, 2H), 4.53 (s, 1H), 3.12 (d, *J* = 6.2 Hz, 2H), 2.46 – 2.38 (m, 2H), 1.55 (h, *J* = 6.8, 6.4 Hz, 2H), 1.46 (s, 9H).

^{13}C NMR (101 MHz, $CDCl_3$) δ 158.03, 155.94, 153.48 (dd, *J* = 290.1, 288.1 Hz), 136.90, 129.36, 128.61, 128.01, 127.46, 125.72, 114.92, 91.13 (dd, *J* = 20.5, 14.4 Hz), 79.17, 70.06, 39.85, 28.40, 28.06, 25.00.

^{19}F NMR (376 MHz, $CDCl_3$) δ = -91.99 (d, *J*=45.9 Hz, 1F), -92.14 (d, *J*=45.9 Hz, 1F).

HRMS (ESI) m/z calcd. for $C_{23}H_{28}F_2NO_3$ $[M+H]^+$: 404.2032, found: 404.2040.

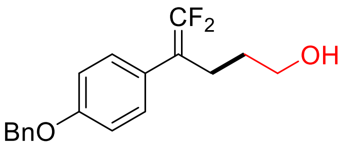

55

Purification by column chromatography on silica gel (hexane/ethyl acetate = 10/1, v/v) afforded **55** as a white solid (30.4 mg, 50% yield).

^{1}H NMR (400 MHz, Chloroform-*d*) δ 7.37 (dt, *J* = 14.7, 7.3 Hz, 4H), 7.31 (d, *J* = 6.9 Hz, 1H), 7.21 (d, *J* = 8.8 Hz, 2H), 6.93 (d, *J* = 8.6 Hz, 2H), 5.03 (s, 2H), 3.58 (t, *J* = 6.4 Hz, 2H), 2.43 (t, *J* = 7.4 Hz, 2H), 1.59 (p, *J* = 6.6 Hz, 2H), 1.52 (s, 1H).

^{13}C NMR (101 MHz, $CDCl_3$) δ 157.96, 153.47 (dd, *J* = 289.7, 286.2, Hz), 136.86, 129.34 (t, *J* = 3.3 Hz), 128.60, 128.01, 127.46, 125.85, 114.87, 91.26 (dd, *J* = 20.9, 14.7 Hz), 70.04, 61.95, 30.66, 24.02.

^{19}F NMR (376 MHz, Chloroform-*d*) δ -92.13 (d, *J* = 45.8 Hz, 1F), -92.29 (d, *J* = 45.8 Hz, 1F).

HRMS (ESI) m/z calcd. for $C_{18}H_{19}F_2O_2$ $[M+H]^+$: 305.1348, found: 305.1345.

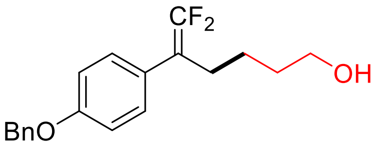

56

Purification by column chromatography on silica gel (hexane/ethyl acetate = 10/1, v/v) afforded **56** as a white solid (38.2 mg, 60% yield).

^{1}H NMR (400 MHz, Chloroform-*d*) δ 7.41 (dt, *J* = 14.5, 7.3 Hz, 4H), 7.36 – 7.30 (m, 1H), 7.23 (d, *J* = 8.6 Hz, 2H), 6.96 (d, *J* = 8.7 Hz, 2H), 5.06 (s, 2H), 3.59 (t, *J* = 6.5 Hz, 2H), 2.44 – 2.35 (m, 2H), 1.56 (p, *J* = 6.5 Hz, 2H), 1.42 (p, *J* = 7.4 Hz, 2H).

^{13}C NMR (101 MHz, $CDCl_3$) δ 157.94, 153.52 (dd, *J* = 288.5, 287.0 Hz), 136.90, 129.36 (t, *J* = 3.2 Hz), 128.60, 128.01, 127.48, 125.99, 114.83, 91.59 (dd, *J* = 19.7, 15.3 Hz), 70.05, 62.58, 31.99, 27.45, 23.91.

^{19}F NMR (376 MHz, Chloroform-*d*) δ -92.53, 2F.

HRMS (ESI) m/z calcd. for $C_{19}H_{21}F_2O_2$ $[M+H]^+$: 319.1504, found: 319.1500.

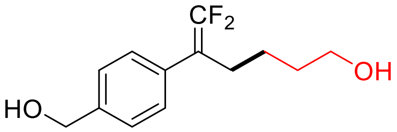

57

Purification by column chromatography on silica gel (hexane/ethyl acetate = 2/1, v/v) afforded **57** as a colorless oil (22.3 mg, 46% yield).

^{1}H NMR (600 MHz, Chloroform-*d*) δ 7.35 (d, *J* = 8.0 Hz, 2H), 7.29 (d, *J* = 8.0 Hz, 2H), 4.67 (s, 2H), 3.58 (t, *J* = 6.5 Hz, 2H), 2.43 (tt, *J* = 7.5, 2.4 Hz, 2H), 1.55 (dt, *J* = 15.3, 6.7 Hz, 2H), 1.42 (p, *J* = 7.6 Hz, 2H).

^{13}C NMR (151 MHz, Chloroform-*d*) δ 153.65 (dd, *J* = 289.6, 287.5 Hz), 139.93, 132.93 (d, *J* = 2.7 Hz), 128.43 (t, *J* = 3.3 Hz), 127.11, 91.93 (dd, *J* = 19.8, 15.1 Hz), 64.94, 62.53, 31.95, 27.34, 23.91.

^{19}F NMR (565 MHz, Chloroform-*d*) δ -91.49 (d, *J* = 44.1 Hz, 1F), -91.58 (d, *J* = 44.0 Hz, 1F)

HRMS (ESI) m/z calcd. for $C_{13}H_{17}F_2O_2$ $[M+H]^+$: 243.1191, found: 243.1194.

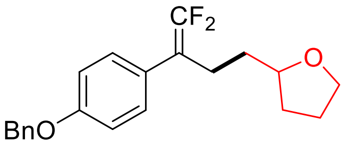

58

Purification by column chromatography on silica gel (hexane/ethyl acetate = 50/1, v/v) afforded **58** as a white solid (40.6 mg, 59% yield).

^{1}H NMR (400 MHz, Chloroform-*d*) δ 7.48 – 7.39 (m, 4H), 7.36 (d, *J* = 7.0 Hz, 1H), 7.27 (d, *J* = 8.5 Hz, 2H), 6.99 (d, *J* = 8.8 Hz, 2H), 5.09 (s, 2H), 3.84 (ddt, *J* = 21.4, 13.6, 7.2 Hz, 2H), 3.73 (q, *J* = 7.6 Hz, 1H), 2.59 – 2.39 (m, 2H), 2.03 – 1.93 (m, 1H), 1.92 – 1.81 (m, 2H), 1.69 – 1.60 (m, 1H), 1.59 – 1.49 (m, 1H), 1.47 – 1.38 (m, 1H).

^{13}C NMR (101 MHz, $CDCl_3$) δ 157.92, 153.38 (dd, *J* = 288.1, 287.0 Hz), 136.94, 129.37 (t, *J* = 3.4 Hz), 128.59, 127.99, 127.46, 126.02, 114.84, 91.63 (dd, *J* = 19.0, 16.0 Hz), 78.57, 70.05, 67.63, 33.81 (t, *J* = 2.4 Hz), 31.22, 25.68, 24.72.

^{19}F NMR (376 MHz, Chloroform-*d*) δ -92.22 (d, *J* = 45.8 Hz, 1F), -92.38 (d, *J* = 45.8 Hz, 1F).

HRMS (ESI) m/z calcd. for $C_{21}H_{23}F_2O_2$ $[M+H]^+$: 345.1661, found: 345.1660.

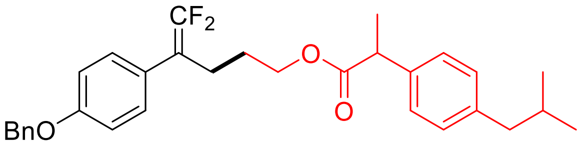

59

Purification by column chromatography on silica gel (hexane/ethyl acetate = 20/1, v/v) afforded **59** as a colorless oil (44.3 mg, 45% yield).

^{1}H NMR (400 MHz, $CDCl_3$) δ = 7.47 – 7.38 (m, 4H), 7.35 (d, *J* = 7.1, 1H), 7.20 (dd, *J* = 15.9, 8.2, 4H), 7.11 (d, *J*=8.0, 2H), 6.96 (d, *J* = 8.7, 2H), 5.07 (s, 2H), 4.04 (t, *J* = 6.2, 2H), 3.70 (q, *J* = 7.2, 1H), 2.46 (d, *J* = 7.2, 2H), 2.35 (t, *J* = 7.4, 2H), 1.85 (dp, *J* = 13.5, 6.7, 1H), 1.69 – 1.60 (m, 2H), 1.50 (d, *J* = 7.2, 3H), 0.90 (d, *J*=6.6, 6H).

^{13}C NMR (101 MHz, $CDCl_3$) δ = 174.68, 157.99, 153.48 (dd, *J* = 289.3, 287.3 Hz), 140.52, 137.77, 136.87, 129.32, 129.30 (t, *J* = 3.4 Hz), 128.62, 128.03, 127.47, 127.16, 125.56, 114.87, 90.87 (dd, *J* = 20.4, 15.0 Hz), 70.04, 63.60, 45.19, 45.05, 30.18, 26.72, 24.05, 22.37, 18.38.

^{19}F NMR (376 MHz, $CDCl_3$) δ = -91.77 (d, *J* = 44.7 Hz, 1F), -91.92 (d, *J* = 44.7 Hz, 1F).

HRMS (ESI) m/z calcd. for $C_{31}H_{35}F_2O_3$ $[M+H]^+$: 493.2549, found: 493.2548.

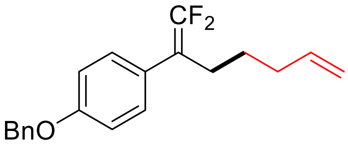

60

Purification by column chromatography on silica gel (hexane/ethyl acetate = 50/1, v/v) afforded **60** as a white solid (39.6 mg, 63% yield).

^{1}H NMR (400 MHz, Chloroform-*d*) δ 7.44 (dt, *J* = 14.6, 7.3 Hz, 4H), 7.36 (dd, *J* = 8.5, 5.6 Hz, 1H), 7.26 (d, *J* = 8.6 Hz, 2H), 7.00 (d, *J* = 8.7 Hz, 2H), 5.80 (ddt, *J* = 16.9, 10.2, 6.7 Hz, 1H), 5.09 (s, 2H), 5.07 – 4.94 (m, 2H), 2.45 – 2.36 (m, 2H), 2.08 (q, *J* = 7.1 Hz, 2H), 1.49 (p, *J* = 7.5 Hz, 2H).

^{13}C NMR (101 MHz, $CDCl_3$) δ 157.95, 153.51 (dd, *J* = 289.6, 285.3 Hz), 138.14, 136.96, 129.38 (t, *J* = 3.3 Hz), 128.60, 128.00, 127.47, 126.17, 114.86, 114.84, 91.66 (dd, *J* = 20.2, 15.0 Hz), 70.08, 33.05, 27.18, 26.96.

^{19}F NMR (376 MHz, Chloroform-*d*) δ -92.51, 2F.

HRMS (ESI) m/z calcd. for $C_{20}H_{21}F_2O$ $[M+H]^+$: 315.1555, found: 315.1550.

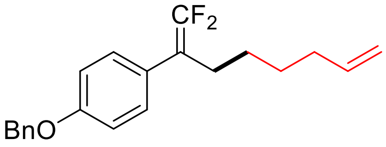

61

Purification by column chromatography on silica gel (hexane/ethyl acetate = 50/1, v/v) afforded **61** as a white solid (42.0 mg, 64% yield).

^{1}H NMR (400 MHz, Chloroform-*d*) δ 7.46 – 7.40 (m, 4H), 7.36 (t, *J* = 7.1 Hz, 1H), 7.25 (d, *J* = 6.6 Hz, 2H), 6.99 (d, *J* = 8.8 Hz, 2H), 5.79 (ddt, *J* = 16.9, 10.2, 6.7 Hz, 1H), 5.09 (s, 2H), 5.04 – 4.90 (m, 2H), 2.43 – 2.33 (m, 2H), 2.04 (d, *J* = 6.7 Hz, 2H), 1.48 – 1.32 (m, 4H).

^{13}C NMR (101 MHz, $CDCl_3$) δ 157.89, 153.49 (t, *J* = 287.8 Hz), 138.69, 136.94, 129.36 (t, *J* = 3.3 Hz), 128.61, 128.01, 127.49, 126.21, 114.78, 114.46, 91.74 (dd, *J* = 19.1, 15.8 Hz), 70.05, 33.39, 28.24, 27.53, 27.15.

^{19}F NMR (376 MHz, Chloroform-*d*) δ -92.69 (d, *J* = 1.2 Hz, 2F).

HRMS (ESI) m/z calcd. for $C_{21}H_{23}F_2O$ $[M+H]^+$: 329.1711, found: 329.1711.

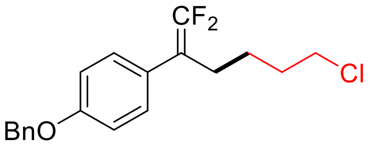

62

Purification by column chromatography on silica gel (hexane/ethyl acetate = 50/1, v/v) afforded **62** as a white solid (40.3 mg, 60% yield).

^{1}H NMR (400 MHz, Chloroform-*d*) δ 7.48 – 7.41 (m, 4H), 7.40 – 7.35 (m, 1H), 7.26 (d, *J* = 8.2 Hz, 2H), 7.03 – 6.99 (m, 2H), 5.10 (s, 2H), 3.52 (t, *J* = 6.7 Hz, 2H), 2.44 (tt, *J* = 7.5, 2.3 Hz, 2H), 1.85 – 1.75 (m, 2H), 1.56 (dt, *J* = 10.2, 6.5 Hz, 2H).

^{13}C NMR (101 MHz, $CDCl_3$) δ 158.04, 153.58 (dd, *J* = 289.3, 286.5 Hz), 136.92, 129.37 (t, *J* = 3.2 Hz), 128.63, 128.03, 127.50, 125.80, 114.92, 91.37 (dd, *J* = 21.0, 14.2 Hz), 70.08, 44.57, 31.81, 26.97, 24.96.

^{19}F NMR (376 MHz, Chloroform-*d*) δ -92.13 (d, *J* = 45.9 Hz, 1F), -92.31 (d, *J* = 46.0 Hz, 1F).

HRMS (ESI) m/z calcd. for $C_{19}H_{20}ClF_2O$ $[M+H]^+$: 337.1165, found: 337.1172.

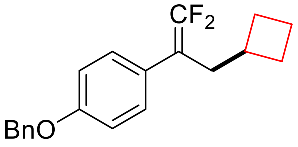

63

Purification by column chromatography on silica gel (hexane/ethyl acetate = 50/1, v/v) afforded **63** as a white solid (38.3 mg, 61% yield).

^{1}H NMR (400 MHz, Chloroform-*d*) δ 7.49 – 7.37 (m, 5H), 7.24 (d, *J* = 8.5 Hz, 2H), 6.99 (d, *J* = 8.7 Hz, 2H), 5.10 (s, 2H), 2.48 (dt, *J* = 7.6, 2.2 Hz, 2H), 2.33 (dt, *J* = 15.5, 7.8 Hz, 1H), 2.02 – 1.93 (m, 2H), 1.86 – 1.78 (m, 2H), 1.72 – 1.61 (m, 2H).

^{13}C NMR (101 MHz, $CDCl_3$) δ 157.92, 153.93 (dd, *J* = 288.5, 285.6 Hz), 136.98, 129.47 (t, *J* = 3.2 Hz), 128.60, 127.99, 127.48, 126.48, 114.73, 90.76 (dd, *J* = 22.1, 13.1 Hz), 70.07, 34.79, 34.32, 27.94, 18.17.

^{19}F NMR (376 MHz, Chloroform-*d*) δ -92.85 (d, *J* = 47.4 Hz, 1F), -93.15 (d, *J* = 47.4 Hz, 1F).

HRMS (ESI) m/z calcd. for $C_{20}H_{21}F_2O$ $[M+H]^+$: 315.1555, found: 315.1553.

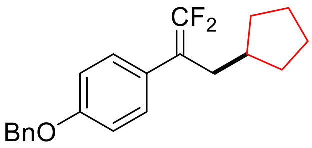

64

Purification by column chromatography on silica gel (hexane/ethyl acetate = 50/1, v/v) afforded **64** as a white solid (40.0 mg, 61% yield).

^{1}H NMR (400 MHz, Chloroform-*d*) δ 7.49 – 7.41 (m, 4H), 7.37 (dd, *J* = 8.2, 5.9 Hz, 1H), 7.28 (d, *J* = 8.1 Hz, 2H), 7.01 (d, *J* = 8.8 Hz, 2H), 5.10 (s, 2H), 2.40 (dt, *J* = 7.4, 2.3 Hz, 2H), 1.84 (dt, *J* = 15.1, 7.5 Hz, 1H), 1.67 (dtd, *J* = 17.1, 9.5, 8.4, 4.3 Hz, 4H), 1.51 (dt, *J* = 7.6, 3.7 Hz, 2H), 1.24 – 1.14 (m, 2H).

^{13}C NMR (101 MHz, $CDCl_3$) δ 157.91, 153.81 (t, *J* = 289.9 Hz), 136.98, 129.50 (t, *J* = 3.2 Hz), 128.61, 128.00, 127.50, 126.47, 114.75, 91.77 (dd, *J* = 21.6, 13.3 Hz), 70.06, 38.25, 33.70, 32.15, 25.00.

^{19}F NMR (376 MHz, Chloroform-*d*) δ -92.93 (d, *J* = 47.5 Hz, 1F), -93.24 (d, *J* = 47.5 Hz, 1F).

HRMS (ESI) m/z calcd. for $C_{21}H_{23}F_2O$ $[M+H]^+$: 329.1711, found: 329.1710.

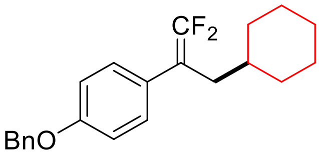

65

Purification by column chromatography on silica gel (hexane/ethyl acetate = 50/1, v/v) afforded **65** as a white solid (45.8 mg, 67% yield).

^{1}H NMR (400 MHz, Chloroform-*d*) δ 7.50 (d, *J* = 6.9 Hz, 2H), 7.47 – 7.42 (m, 2H), 7.39 (dd, *J* = 8.3, 5.7 Hz, 1H), 7.32 – 7.26 (m, 2H), 7.05 – 6.99 (m, 2H), 5.11 (s, 2H), 2.30 (dt, *J* = 7.1, 2.3 Hz, 2H), 1.77 – 1.64 (m, 5H), 1.32 (ddq, *J* = 14.8, 7.4, 3.7, 2.9 Hz, 1H), 1.18 (s, 3H), 1.04 – 0.91 (m, 2H).

^{13}C NMR (101 MHz, $CDCl_3$) δ 157.90, 153.96 (dd, *J* = 289.0, 285.5 Hz), 137.02, 129.44 (t, *J* = 3.2 Hz), 128.64, 128.03, 127.53, 126.62, 114.79, 90.54 (dd, *J* = 22.1, 13.0 Hz), 70.08, 35.71, 35.36, 32.91, 26.48, 26.12.

^{19}F NMR (376 MHz, Chloroform-*d*) δ -92.14 (d, *J*= 46.5 Hz, 1F), -92.56 (d, *J*= 46.4 Hz, 1F).

HRMS (ESI) m/z calcd. for $C_{22}H_{25}F_2O$ $[M+H]^+$: 343.1868, found: 343.1875.

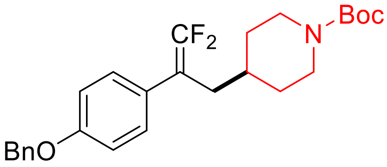

66

Purification by column chromatography on silica gel (hexane/ethyl acetate = 20/1, v/v) afforded **66** as a brown solid (73.5 mg, 83% yield).

^{1}H NMR (400 MHz, Chloroform-*d*) δ 7.49 – 7.37 (m, 4H), 7.35 (d, *J* = 7.1 Hz, 1H), 7.23 (d, *J* = 8.3 Hz, 2H), 6.98 (d, *J* = 8.6 Hz, 2H), 5.07 (s, 2H), 4.04 (s, 2H), 2.59 (d, *J* = 10.4 Hz, 2H), 2.30 (d, *J* = 7.4 Hz, 2H), 1.66 – 1.56 (m, 2H), 1.45 (s, 9H), 1.43 – 1.36 (m, 1H), 1.18 – 1.04 (m, 2H).

^{13}C NMR (101 MHz, $CDCl_3$) δ 157.99, 154.78, 153.95 (dd, *J* = 290.6, 287.1 Hz), 136.85, 129.32 (t, *J* = 3.1 Hz Hz), 128.61, 128.03, 127.48, 126.03, 114.88, 89.92 (dd, *J* = 21.8, 13.7 Hz Hz), 79.26, 70.06, 43.59, 34.45, 34.21, 31.70, 28.44.

^{19}F NMR (376 MHz, Chloroform-*d*) δ -91.59 (d, *J* = 45.1 Hz, 1F), -91.93 (d, *J* = 45.1 Hz, 1F).

HRMS (ESI) m/z calcd. for $C_{26}H_{32}F_2NO_3$ $[M+H]^+$: 444.2345, found: 444.2335.

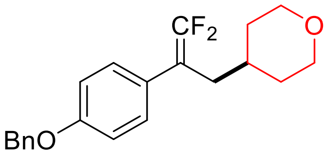

67

Purification by column chromatography on silica gel (hexane/ethyl acetate = 50/1, v/v) afforded **67** as a white solid (44.8 mg, 71% yield).

^{1}H NMR (400 MHz, Chloroform-*d*) δ 7.50 – 7.39 (m, 4H), 7.37 (d, *J* = 7.1 Hz, 1H), 7.26 (d, *J* = 8.4 Hz, 2H), 7.00 (d, *J* = 8.7 Hz, 2H), 5.09 (s, 2H), 3.94 (dd, *J* = 11.4, 3.8 Hz, 2H), 3.29 (t, *J* = 11.1 Hz, 2H), 2.34 (dd, *J* = 4.5, 2.2 Hz, 2H), 1.62 – 1.47 (m, 3H), 1.33 (ddt, *J* = 15.0, 11.0, 5.9 Hz, 2H).

^{13}C NMR (101 MHz, $CDCl_3$) δ 158.02, 154.02 (dd, *J* = 289.4, 286.4 Hz), 136.90, 129.35 (t, *J* = 3.2 Hz) 128.62, 128.04, 127.49, 126.11, 114.91, 89.82 (dd, *J* = 21.8, 13.6 Hz)., 70.08, 67.79, 34.82, 33.23, 32.65.

^{19}F NMR (376 MHz, Chloroform-*d*) δ -91.63 (d, *J* = 45.2 Hz, 1F), -91.93 (d, *J* = 45.3 Hz, 1F).

HRMS (ESI) m/z calcd. for $C_{21}H_{23}F_2O_2$ $[M+H]^+$: 345.1661, found: 345.1670.

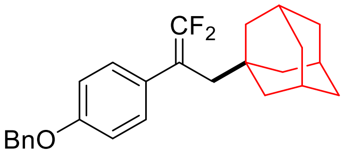

68

Purification by column chromatography on silica gel (hexane/ethyl acetate = 50/1, v/v) afforded **68** as a white solid (47.3 mg, 60% yield).

^{1}H NMR (400 MHz, Chloroform-*d*) δ 7.48 (d, *J* = 7.2 Hz, 2H), 7.43 (t, *J* = 7.3 Hz, 2H), 7.37 (dd, *J* = 8.3, 5.6 Hz, 1H), 7.31 – 7.26 (m, 2H), 7.01 – 6.97 (m, 2H), 5.09 (s, 2H), 2.25 – 2.18 (m, 2H), 1.91 (s, 3H), 1.67 (d, *J* = 12.2 Hz, 3H), 1.61 – 1.55 (m, 3H), 1.47 – 1.37 (m, 6H).

^{13}C NMR (101 MHz, $CDCl_3$) δ 157.70, 154.34 (dd, *J* = 289.4, 286.9 Hz), 137.00, 129.47 (t, *J* = 2.9 Hz), 128.60, 128.01, 127.56, 127.47, 114.64, 89.21 (dd, *J* = 21.8, 12.8 Hz), 70.08, 42.73, 41.99, 36.93, 34.63, 28.68.

^{19}F NMR (376 MHz, Chloroform-*d*) δ -89.88 (d, *J* = 43.2 Hz, 1F), -92.90 (d, *J* = 43.2 Hz, 1F).

HRMS (ESI) m/z calcd. for $C_{26}H_{29}F_2O$ $[M+H]^+$: 395.2181, found: 395.2183 .

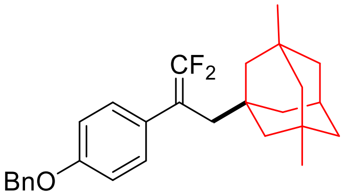

69

Purification by column chromatography on silica gel (hexane/ethyl acetate = 50/1, v/v) afforded **69** as a white solid (49.0 mg, 58% yield).

^{1}H NMR (400 MHz, Chloroform-*d*) δ 7.48 (d, *J* = 7.4 Hz, 2H), 7.43 (t, *J* = 7.3 Hz, 2H), 7.37 (dd, *J* = 8.3, 5.9 Hz, 1H), 7.27 (d, *J* = 7.9 Hz, 2H), 6.98 (d, *J* = 8.7 Hz, 2H), 5.10 (s, 2H), 2.24 (t, *J* = 2.6 Hz, 2H), 2.02 – 1.93 (m, 1H), 1.23 (d, *J* = 21.1 Hz, 6H), 1.11 (d, *J* = 12.0 Hz, 3H), 1.03 (d, *J* = 12.2 Hz, 3H), 0.78 (s, 6H).

^{13}C NMR (101 MHz, $CDCl_3$) δ 157.67, 154.30 (dd, *J* = 289.5, 286.6 Hz), 136.99, 129.44 (t, *J* = 3.0 Hz), 128.33 (dd, *J* = 4.6, 2.9 Hz), 128.29, 127.97, 127.51, 114.66, 89.28 (dd, *J* = 21.9, 13.1 Hz), 70.06, 51.12, 49.08, 43.15, 41.35, 41.17, 36.31, 36.29, 36.26, 31.22, 30.64, 29.73.

^{19}F NMR (376 MHz, Chloroform-*d*) δ -89.80 (d, *J* = 43.3 Hz, 1F), -92.78 (d, *J* = 43.3 Hz, 1F).

HRMS (ESI) m/z calcd. for $C_{28}H_{33}F_2O$ $[M+H]^+$: 423.2494, found: 423.2498.

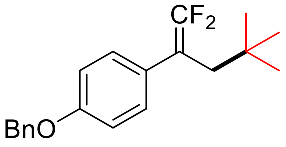

70

Purification by column chromatography on silica gel (hexane/ethyl acetate = 50/1, v/v) afforded **70** as a white solid (39.8 mg, 63% yield).

^{1}H NMR (400 MHz, Chloroform-*d*) δ 7.50 – 7.45 (m, 2H), 7.42 (t, *J* = 7.3 Hz, 2H), 7.36 (dd, *J* = 8.4, 5.8 Hz, 1H), 7.27 (dd, *J* = 8.7, 1.2 Hz, 2H), 6.99 (d, *J* = 8.8 Hz, 2H), 5.08 (s, 2H), 2.34 (t, *J* = 2.4 Hz, 2H), 0.85 (s, 9H).

^{13}C NMR (101 MHz, $CDCl_3$) δ 157.78, 154.35 (dd, *J* = 289.2, 286.9 Hz), 136.97, 129.55 (t, *J* = 2.9 Hz), 128.05 (dd, *J* = 4.7, 3.2 Hz), 128.01, 127.98, 127.52, 114.68, 90.56 (dd, *J*=21.5, 13.1 Hz), 70.08, 41.23, 32.66, 29.77.

^{19}F NMR (376 MHz, Chloroform-*d*) δ -90.64 (d, *J* = 43.3 Hz, 1F), -93.17 (d, *J* = 43.2 Hz, 1F).

HRMS (ESI) m/z calcd. for $C_{20}H_{23}F_2O$ $[M+H]^+$: 317.1711, found: 317.1716.

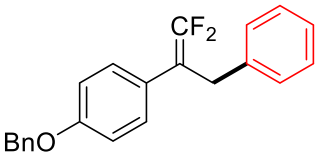

71

Purification by column chromatography on silica gel (hexane/ethyl acetate = 50/1, v/v) afforded **71** as a white solid (37.0 mg, 55% yield)

^{1}H NMR (400 MHz, Chloroform-*d*) δ = 7.39 (q, *J* = 7.9 Hz, 4H), 7.34 (d, *J* = 6.9 Hz, 1H), 7.25 (d, *J* = 7.3 Hz, 2H), 7.18 (t, *J* = 9.3 Hz, 5H), 6.90 (d, *J* = 8.5 Hz, 2H), 5.02 (s, 2H), 3.71 (s, 2H).

^{13}C NMR (101 MHz, $CDCl_3$) δ = 157.92, 154.30 (dd, *J* = 290.9, 288.1 Hz), 138.57 (t, *J* = 2.7 Hz), 136.87, 129.42 (t, *J* = 3.5 Hz), 128.58, 128.46, 128.28, 127.99, 127.46, 126.35, 125.98 (t, *J* = 3.6 Hz), 114.73, 91.13 (dd, *J* = 21.3, 13.9 Hz), 69.99, 33.99.

^{19}**F NMR** (376 MHz, Chloroform-*d*) δ = -91.32 (dd, *J* = 42.4 Hz, 3.8 Hz, 1F), -91.75 (dd, *J* = 42.4 Hz, 3.8 Hz, 1F).

HRMS (ESI) m/z calcd. for $C_{22}H_{19}F_2O$ $[M+H]^+$: 337.1398, found: 337.1398.

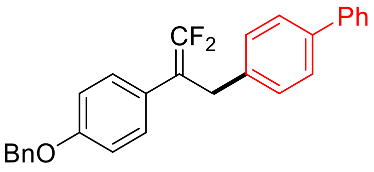

72

Purification by column chromatography on silica gel (hexane/ethyl acetate = 50/1, v/v) afforded **72** as a white solid (42.0 mg, 51% yield).

^{1}**H NMR** (400 MHz, Chloroform-*d*) δ = 7.58 (d, *J* = 7.6 Hz, 2H), 7.50 (t, *J* = 7.1 Hz, 2H), 7.43 (dd, *J* = 15.0 Hz, 8.4 Hz, 6H), 7.34 (t, *J* = 7.3 Hz, 2H), 7.25 (d, *J* = 8.4 Hz, 4H), 6.93 (d, *J* = 8.7 Hz, 2H), 5.05 (s, 2H), 3.77 (s, 2H).

^{13}**C NMR** (101 MHz, $CDCl_3$) δ = 157.94, 154.33 (dd, *J* = 290.9, 287.2 Hz), 140.84, 139.29, 137.66 (t, *J* = 2.7 Hz), 136.86, 129.43 (t, *J* = 3.5 Hz), 128.71, 128.68, 128.58, 127.99, 127.46, 127.19, 127.12, 126.96, 125.94, 114.78, 91.06 (dd, *J* = 21.2, 14.0 Hz), 70.00, 33.64.

^{19}**F NMR** (376 MHz, Chloroform-*d*) δ = -91.14 (d, *J* = 42.2 Hz, 1F), -91.55 (d, *J* = 42.2 Hz, 1F).

HRMS (ESI) m/z calcd. for $C_{28}H_{23}F_2O$ $[M+H]^+$: 413.1711, found: 413.1710.

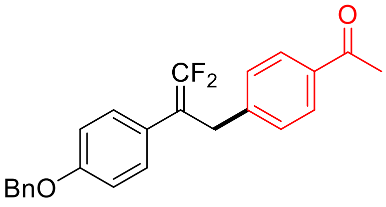

73

Purification by column chromatography on silica gel (hexane/ethyl acetate = 20/1, v/v) afforded **73** as a white solid (46.9 mg, 62% yield).

^{1}**H NMR** (400 MHz, Chloroform-*d*) δ = 7.84 (d, *J* = 8.1 Hz, 2H), 7.39 (t, *J* = 7.9 Hz, 4H), 7.34 – 7.29 (m, 1H), 7.24 (d, *J* = 8.0 Hz, 2H), 7.16 (d, *J*=8.4 Hz, 2H), 6.89 (d, *J* = 8.7 Hz, 2H), 5.01 (s, 2H), 3.75 (s, 2H), 2.55 (s, 3H).

^{13}**C NMR** (101 MHz, $CDCl_3$) δ = 197.70, 158.04, 154.31 (dd, *J* = 291.2, 287.5 Hz), 136.77, 135.57, 134.01, 129.35 (t, *J* = 3.5 Hz), 128.63, 128.58, 128.50, 128.02, 127.45, 125.41, 114.86, 90.63 (dd, *J* = 21.2, 14.6 Hz), 69.99, 33.99, 26.51.

^{19}**F NMR** (376 MHz, Chloroform-*d*) δ = -90.75 (d, *J* = 41.2 Hz, 1F), -91.09 (d, *J* = 41.1 Hz, 1F).

HRMS (ESI) m/z calcd. for $C_{24}H_{21}F_2O_2$ $[M+H]^+$: 379.1504, found: 379.1500.

3. NMR spectra for the products

NMR spectrum of 1

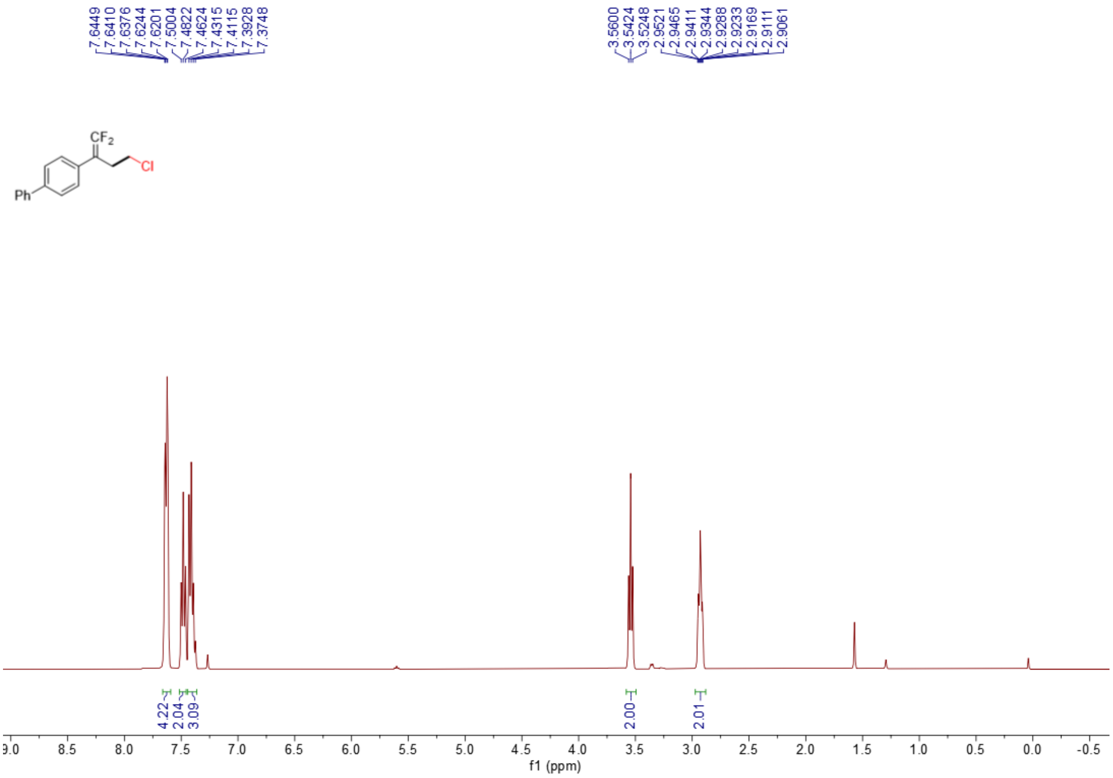

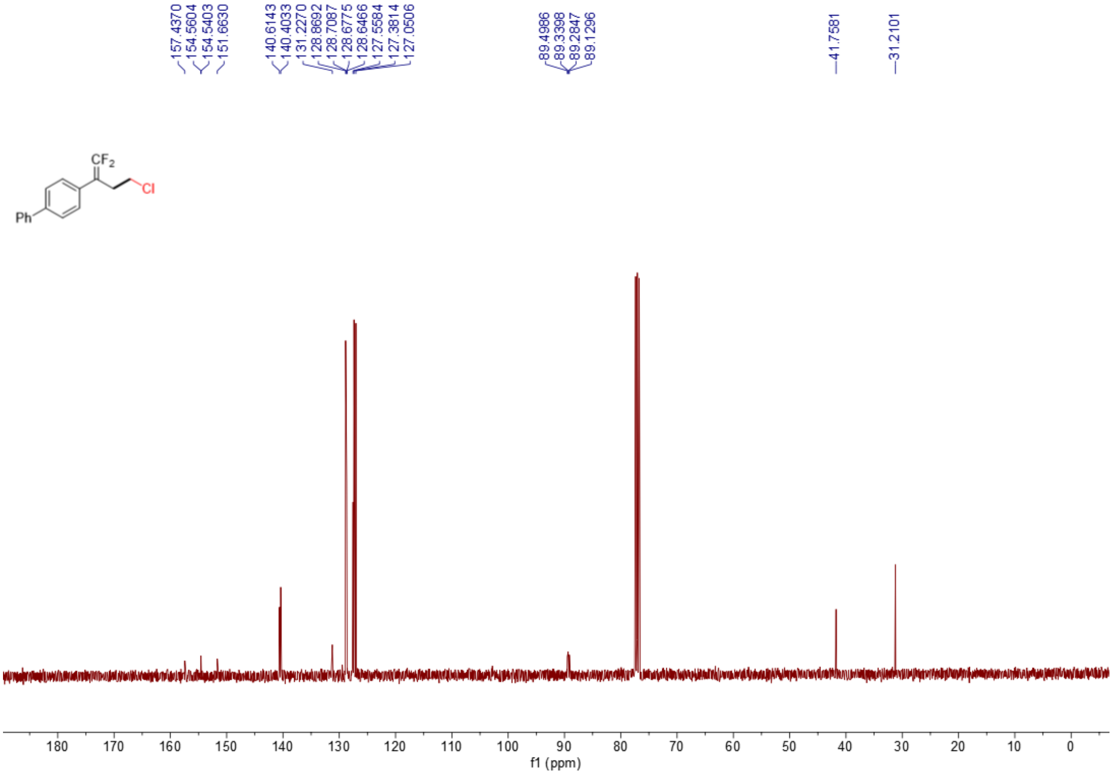

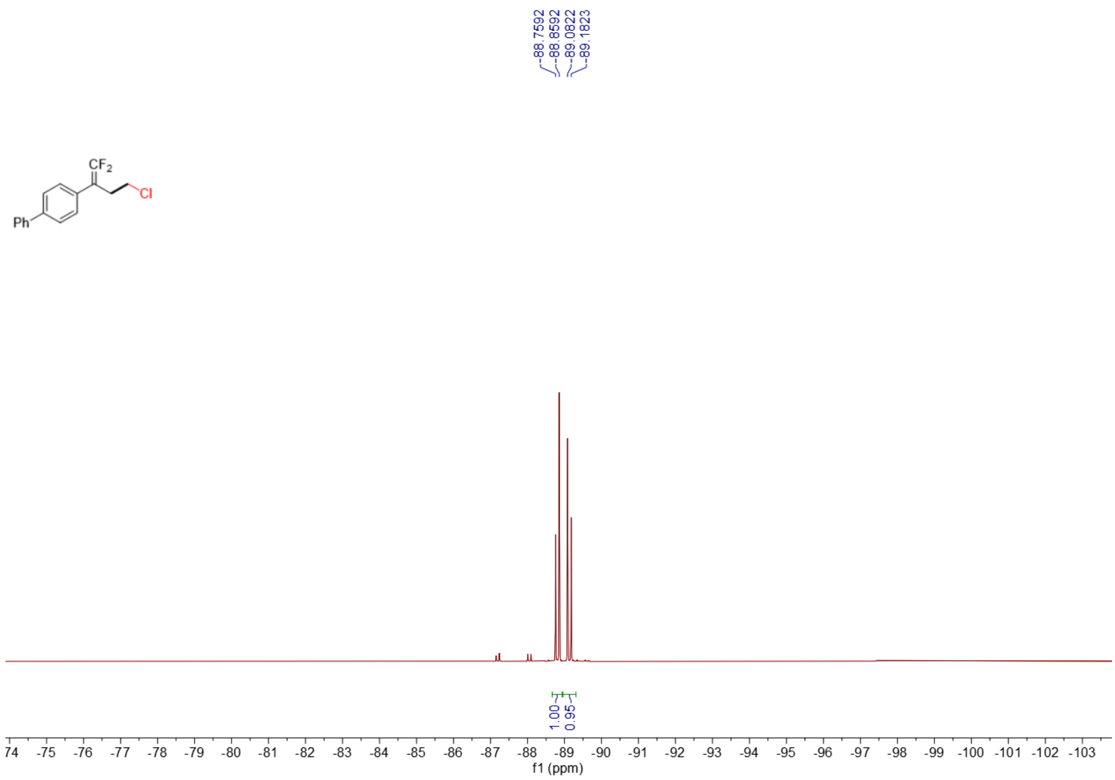

NMR spectrum of 2

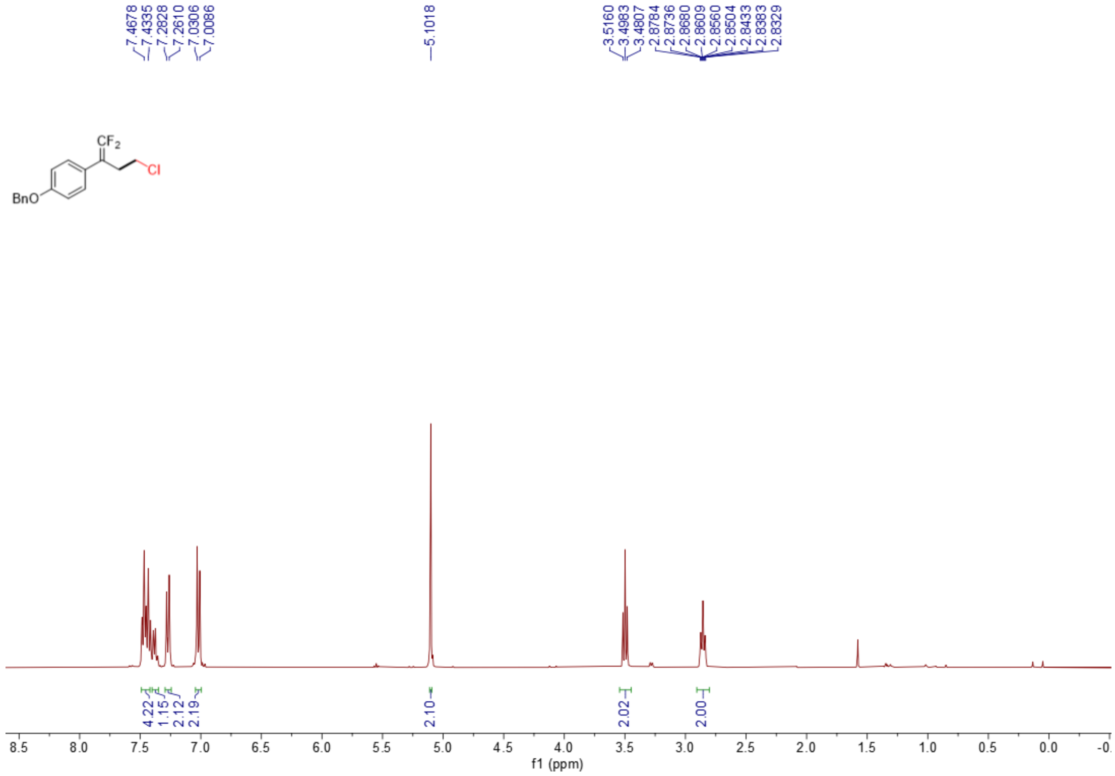

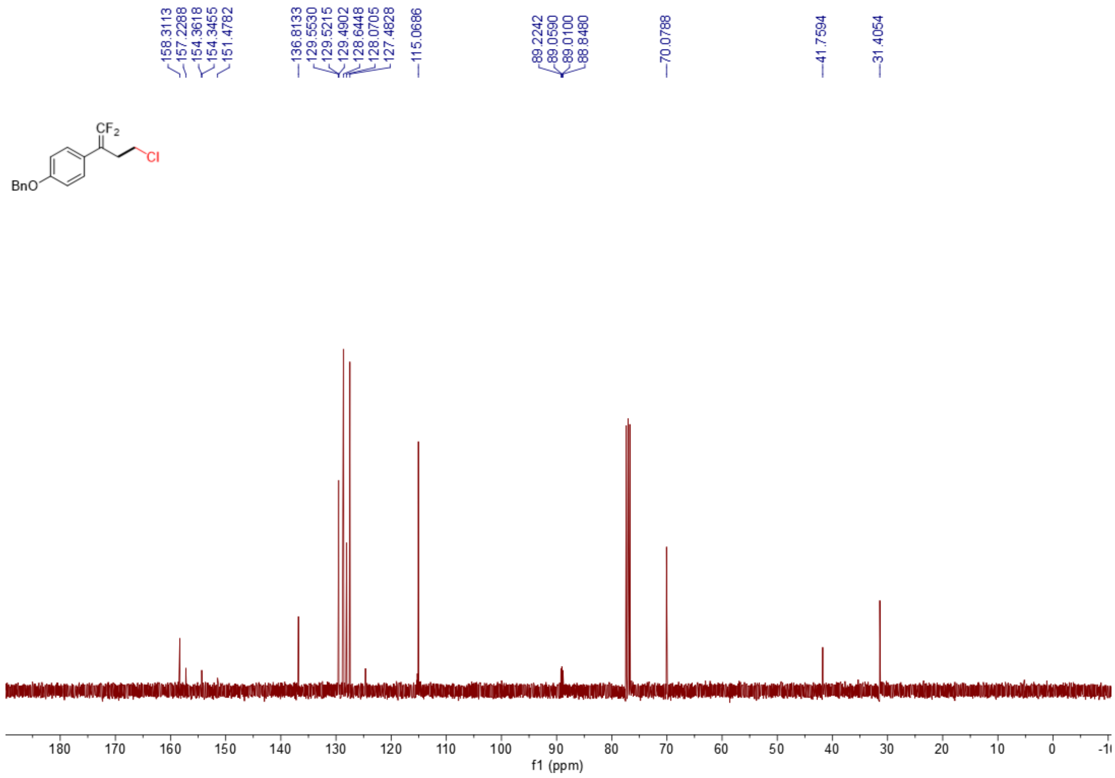
158.3113
157.2288
154.3618
154.3455
151.4782
136.8133
129.5530
129.5215
129.4902
128.6448
128.0705
127.4828
115.0686
89.2242
89.0590
89.0100
88.8480
70.0788
41.7594
31.4054
CF2
Cl
BnO
f1 (ppm)

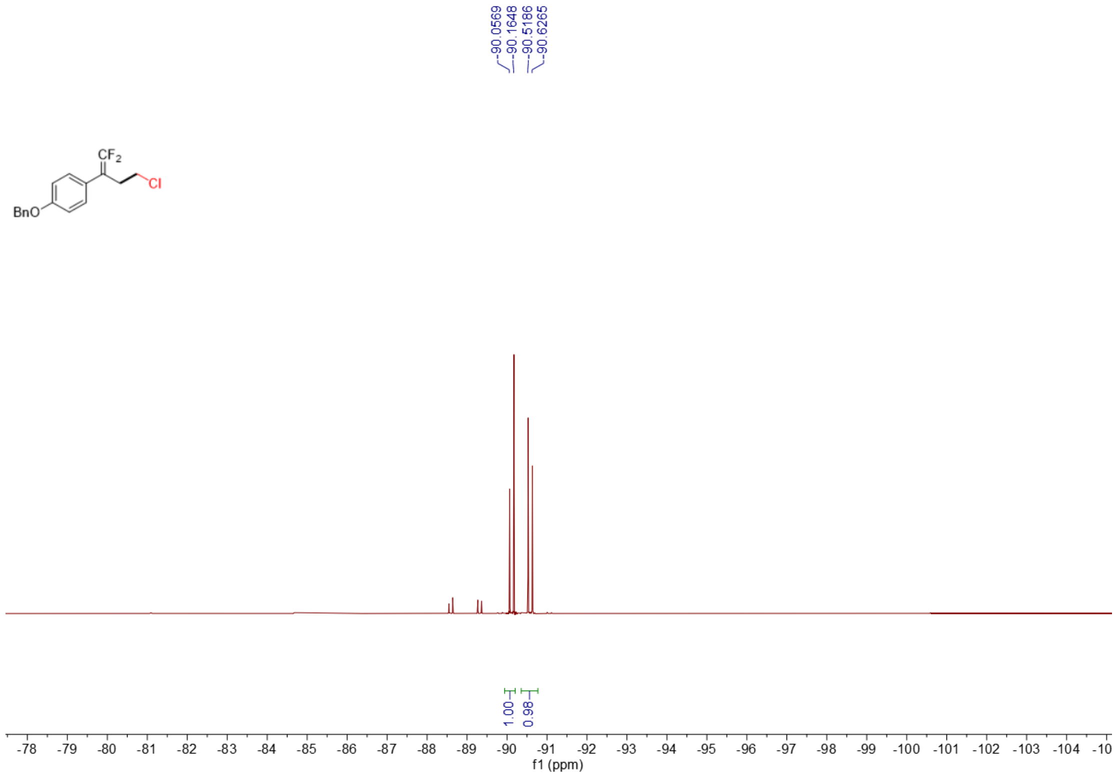
-90.0569
-90.1648
-90.5186
-90.6265
CF2
Cl
BnO
1.00
0.98
f1 (ppm)

NMR spectrum of 3

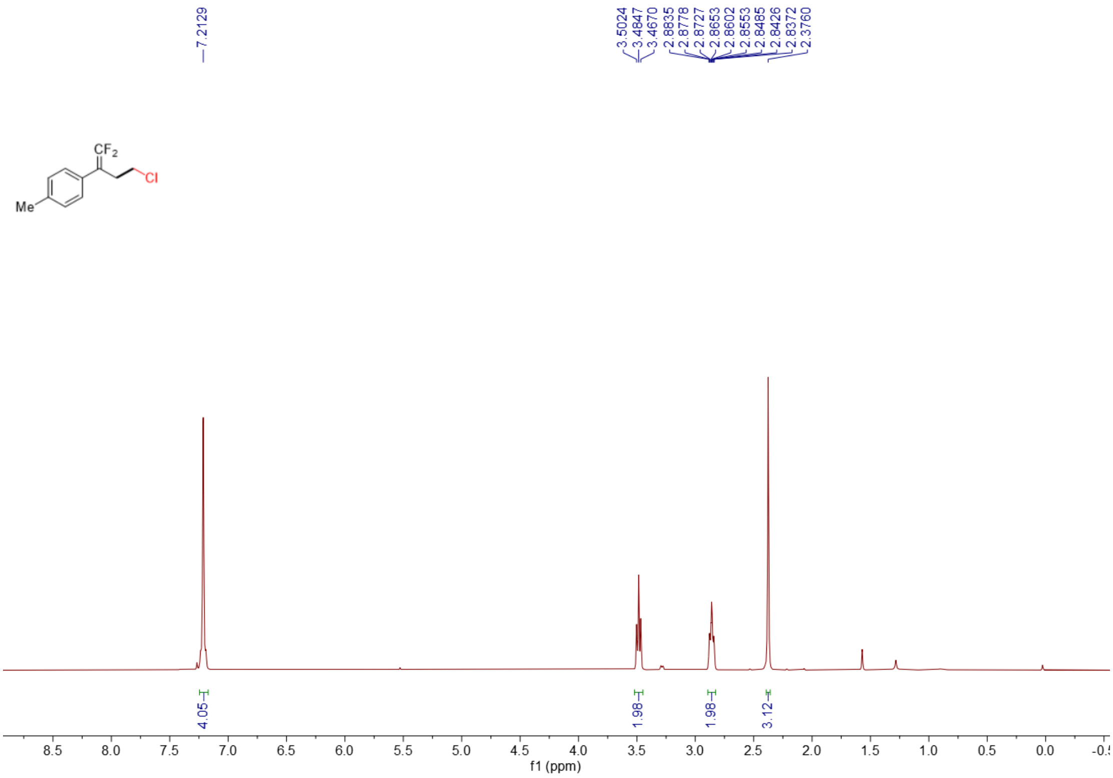

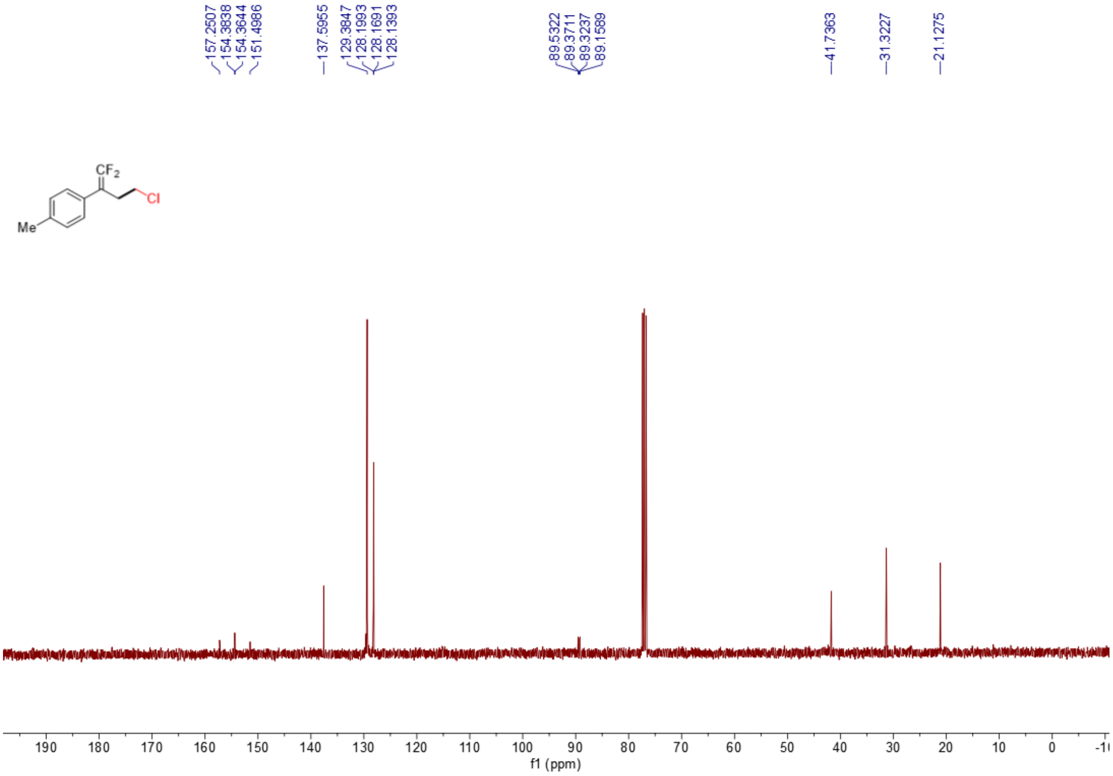

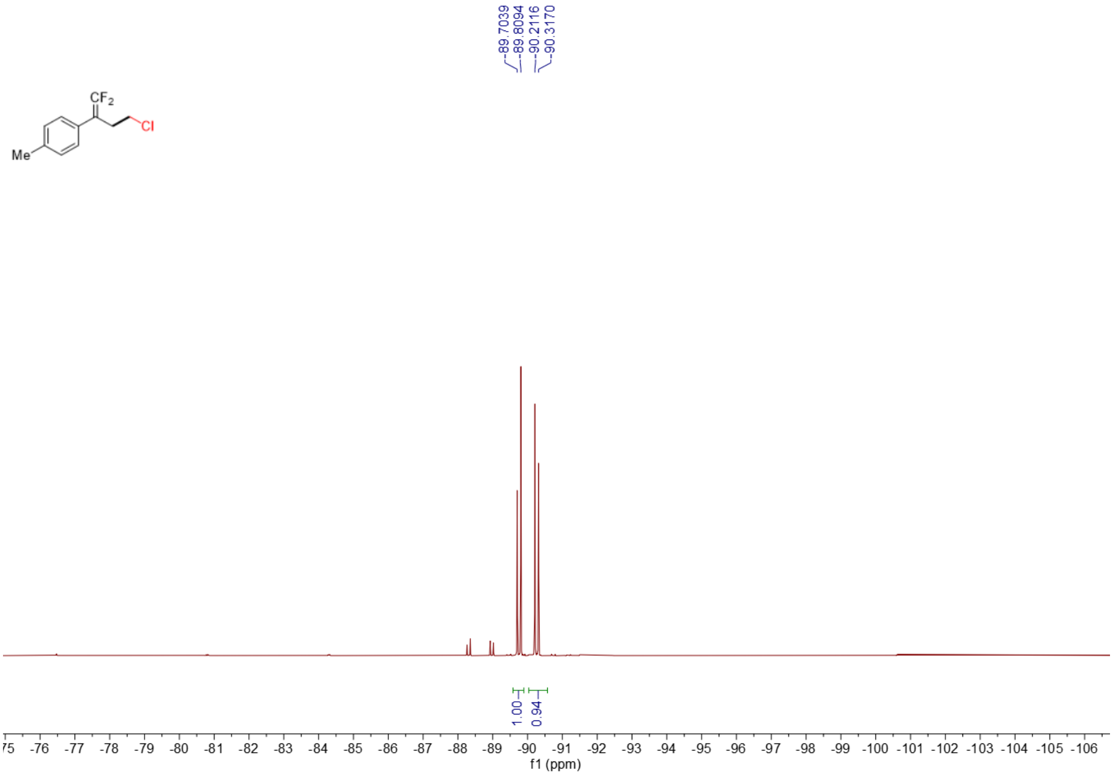

NMR spectrum of 4

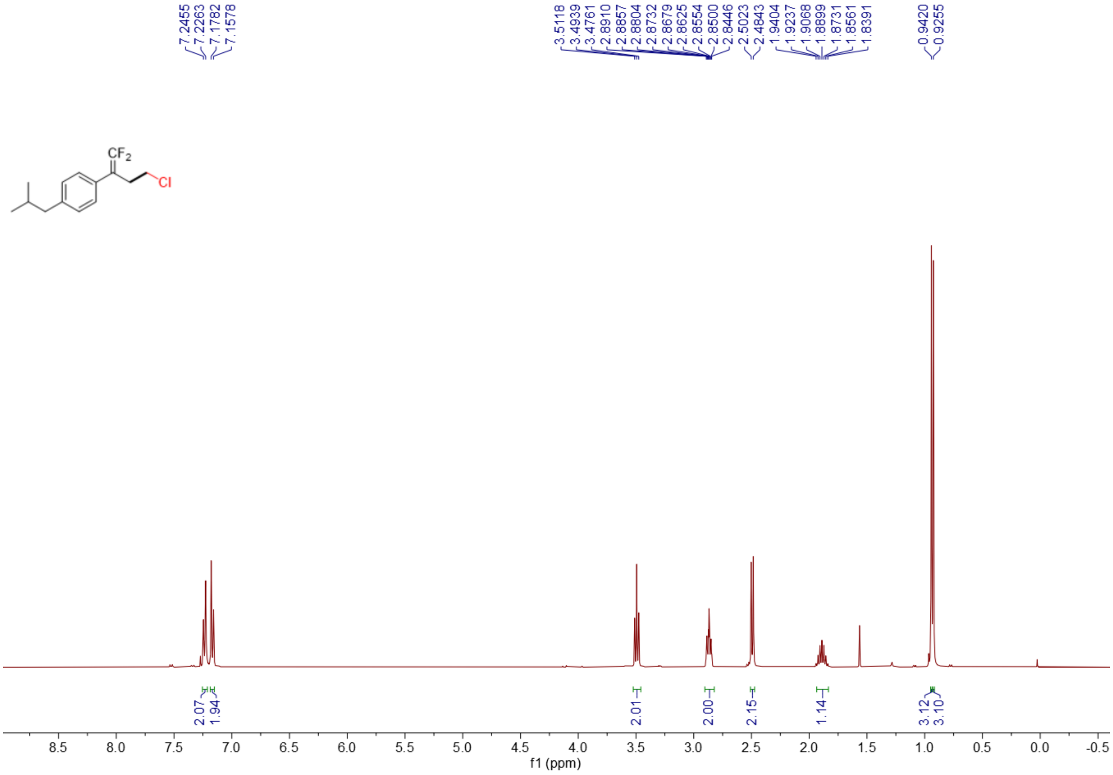

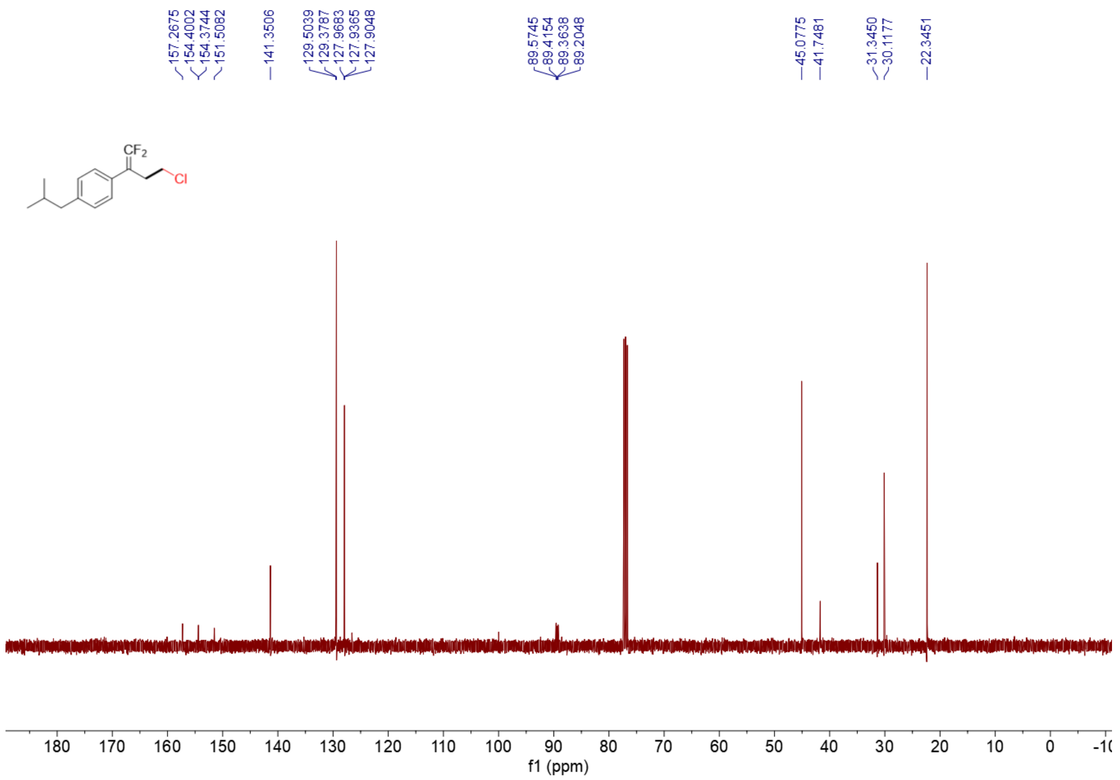
157.2675
154.4002
154.3744
151.5082
141.3506
129.5039
129.3787
127.9683
127.9365
127.9048
89.5745
89.4154
89.3638
89.2048
45.0775
41.7481
31.3450
30.1177
22.3451
CF2
Cl
f1 (ppm)

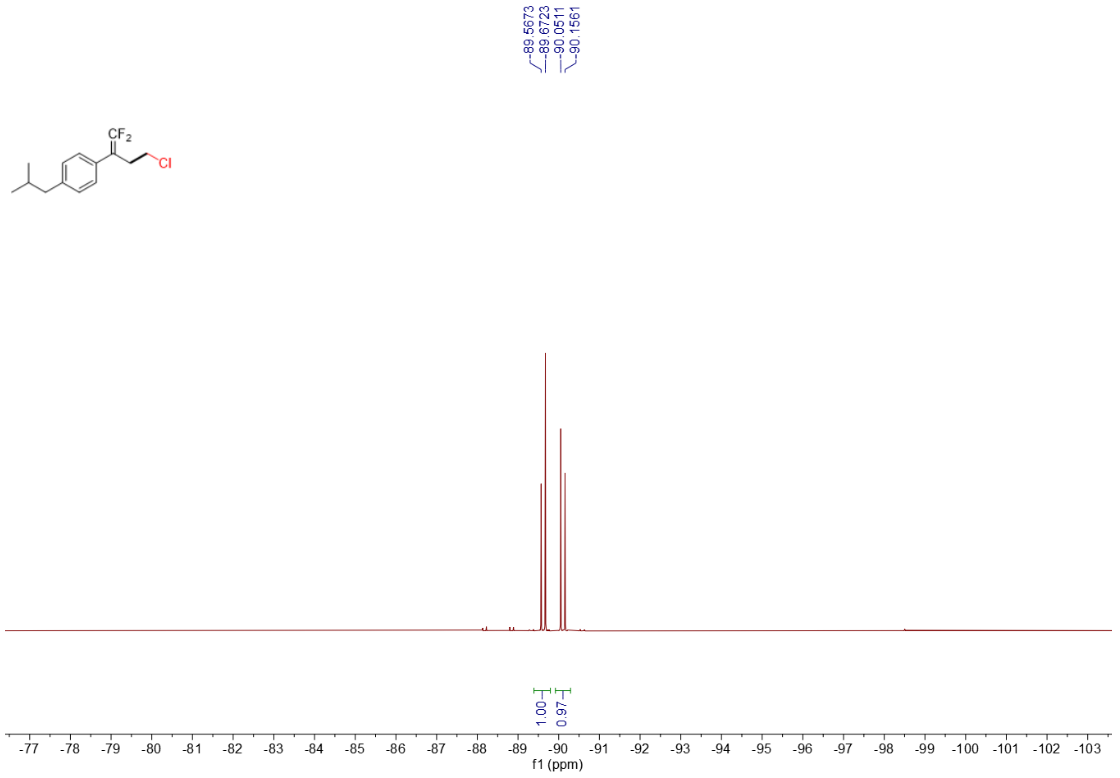
-89.5673
-89.6723
-90.0511
-90.1561
CF2
Cl
1.00
0.97
f1 (ppm)

NMR spectrum of 5

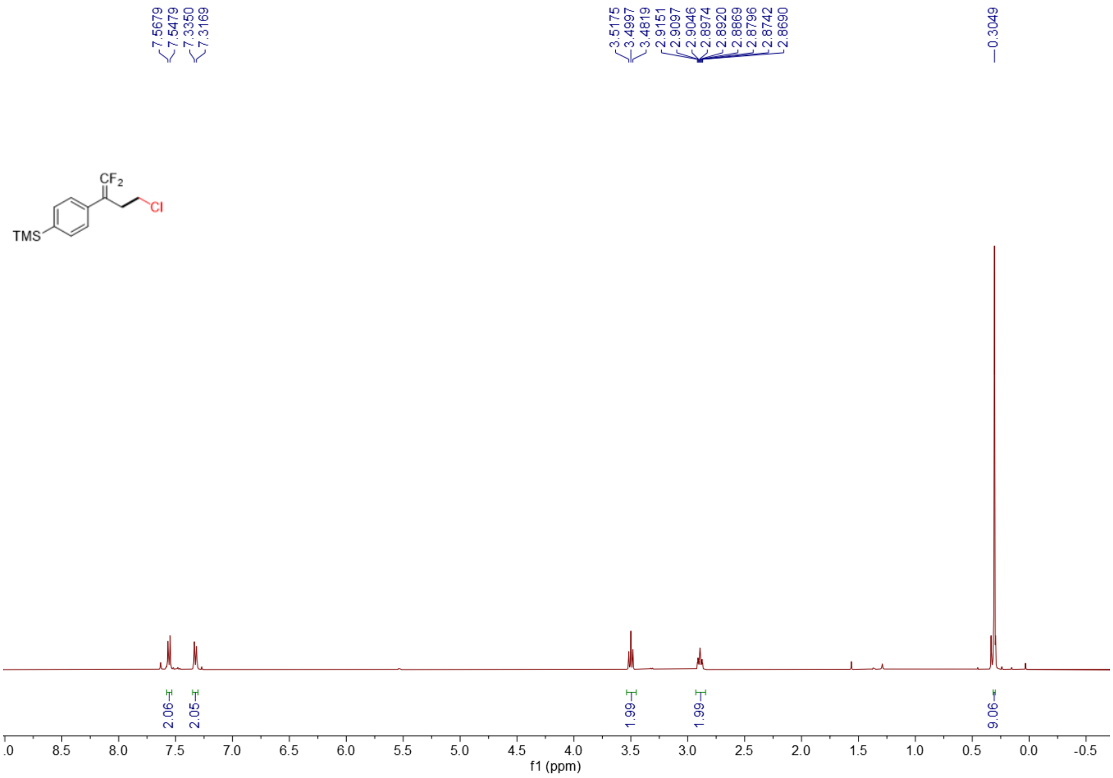

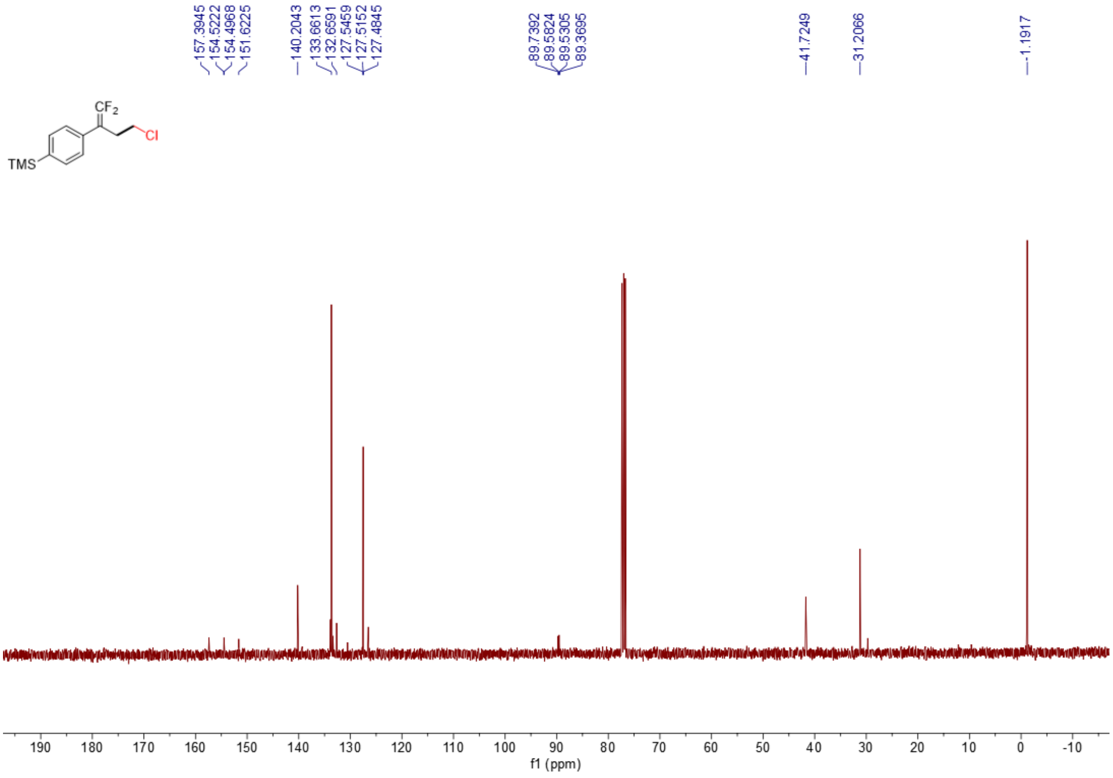

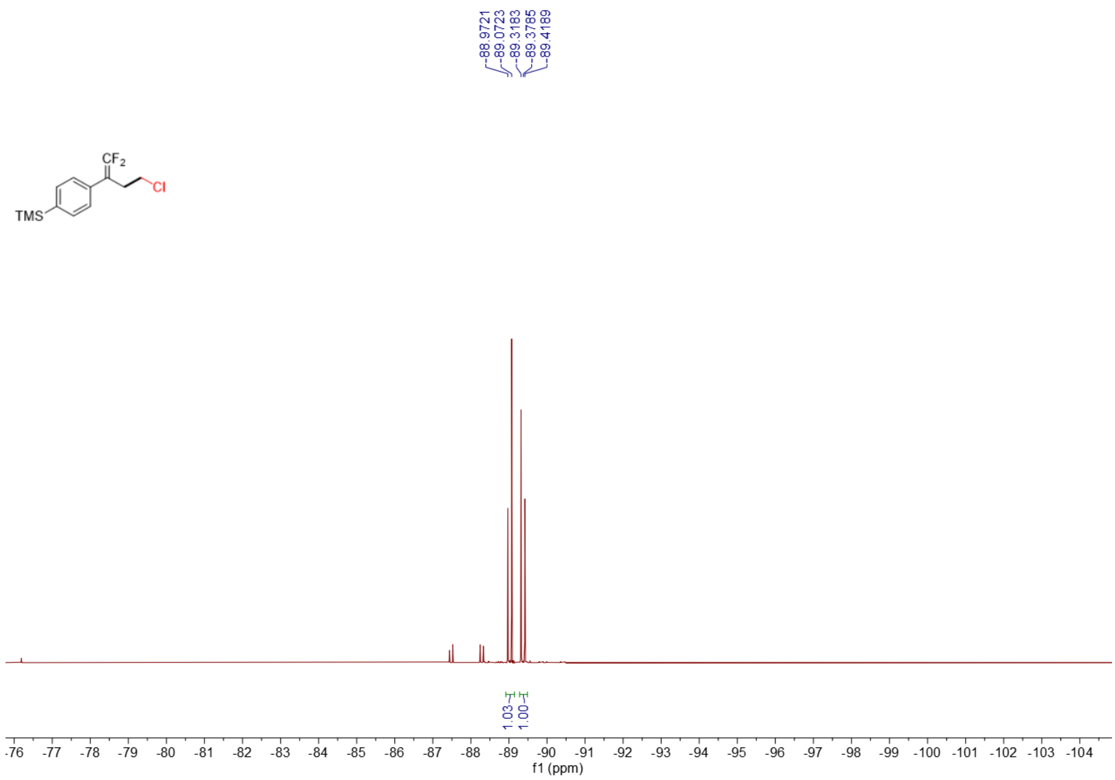

NMR spectrum of 6

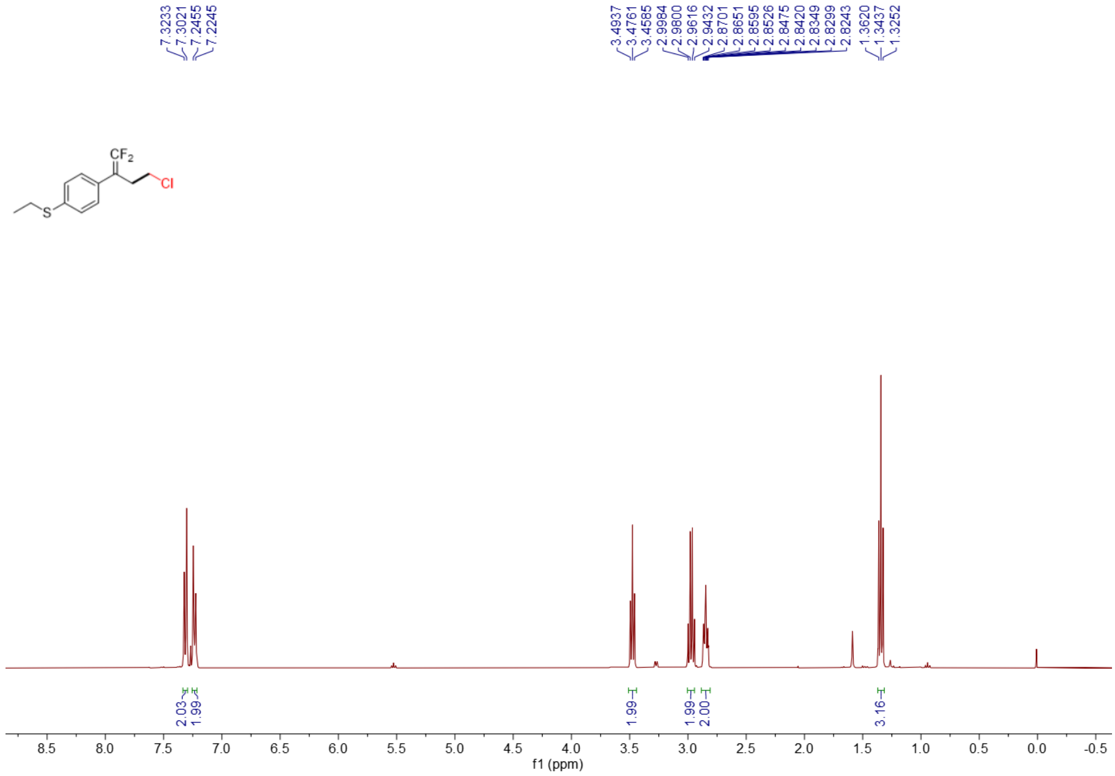

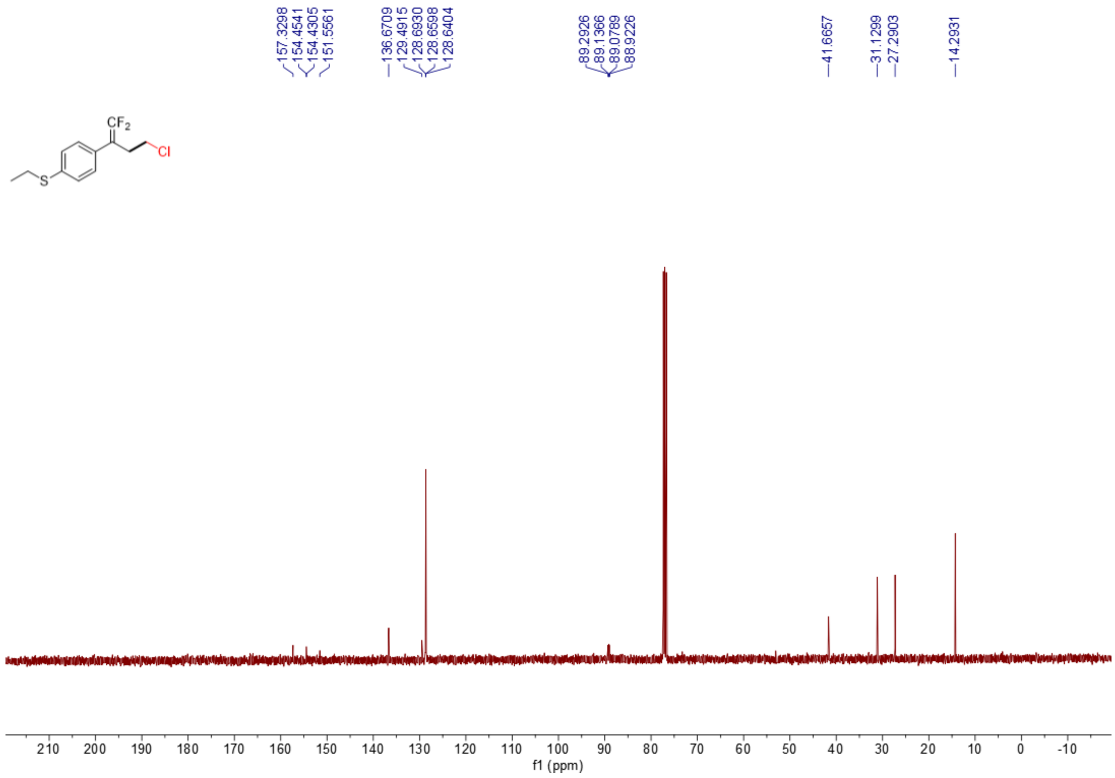
157.3298
154.4541
154.4305
151.5561
136.6709
129.4915
128.6930
128.6598
128.6404
89.2926
89.1366
89.0789
88.9226
41.6657
31.1299
27.2903
14.2931
CF2
S
Cl
210 200 190 180 170 160 150 140 130 120 110 100 90 80 70 60 50 40 30 20 10 0 -10
f1 (ppm)

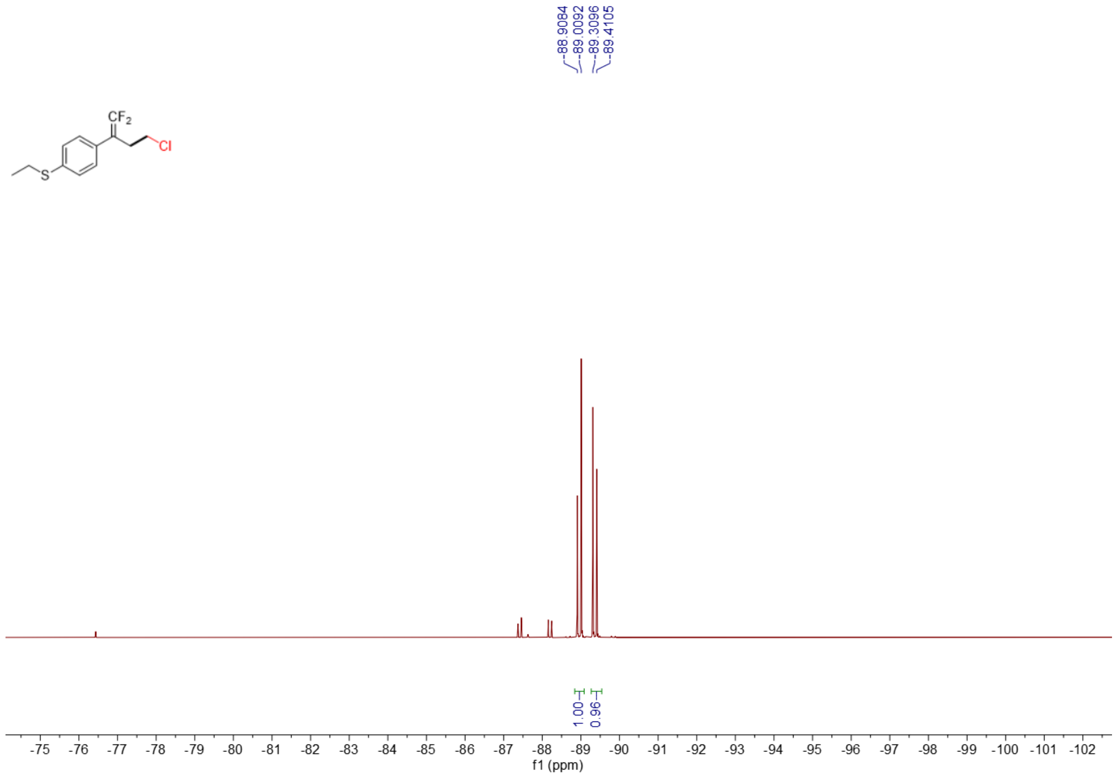
-88.9084
-89.0092
-89.3096
-89.4105
CF2
S
Cl
1.00
0.96
-75 -76 -77 -78 -79 -80 -81 -82 -83 -84 -85 -86 -87 -88 -89 -90 -91 -92 -93 -94 -95 -96 -97 -98 -99 -100 -101 -102
f1 (ppm)

NMR spectrum of 7

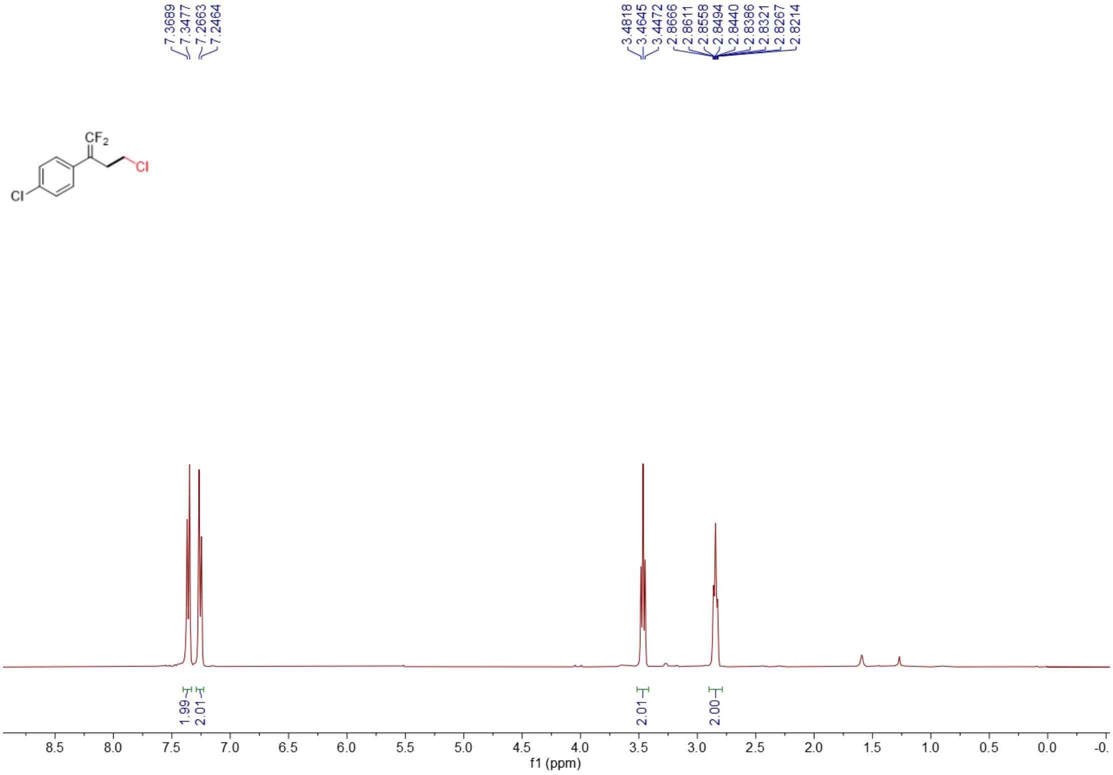

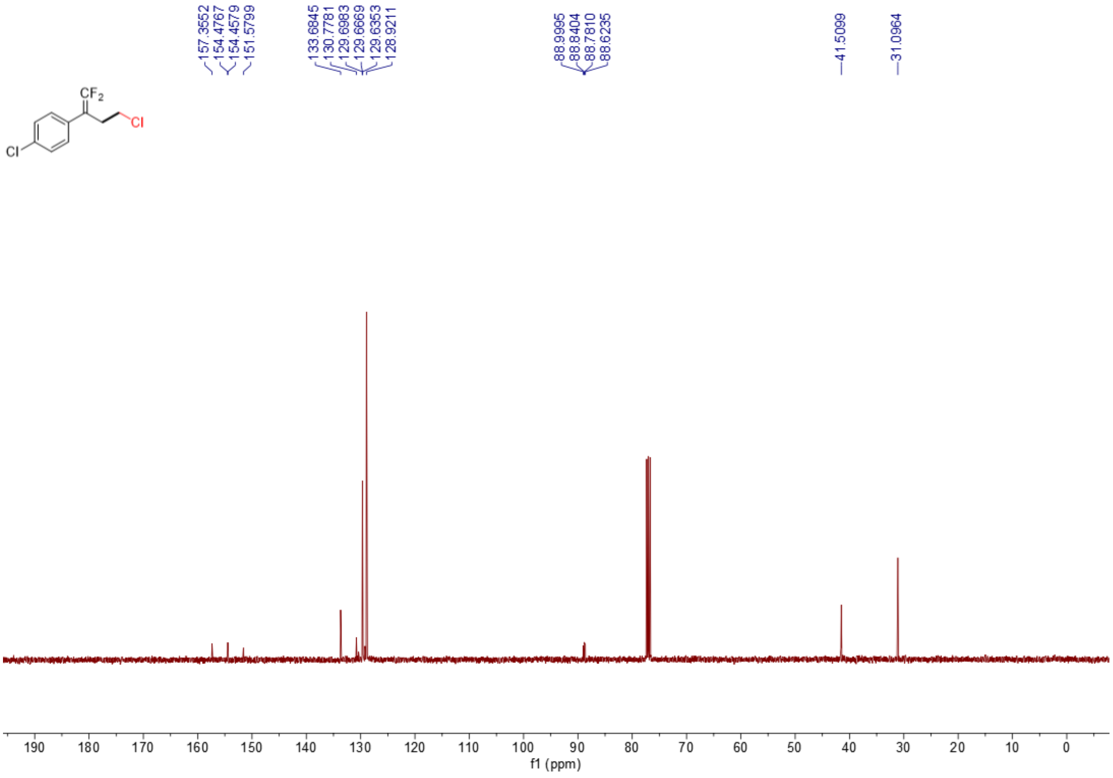

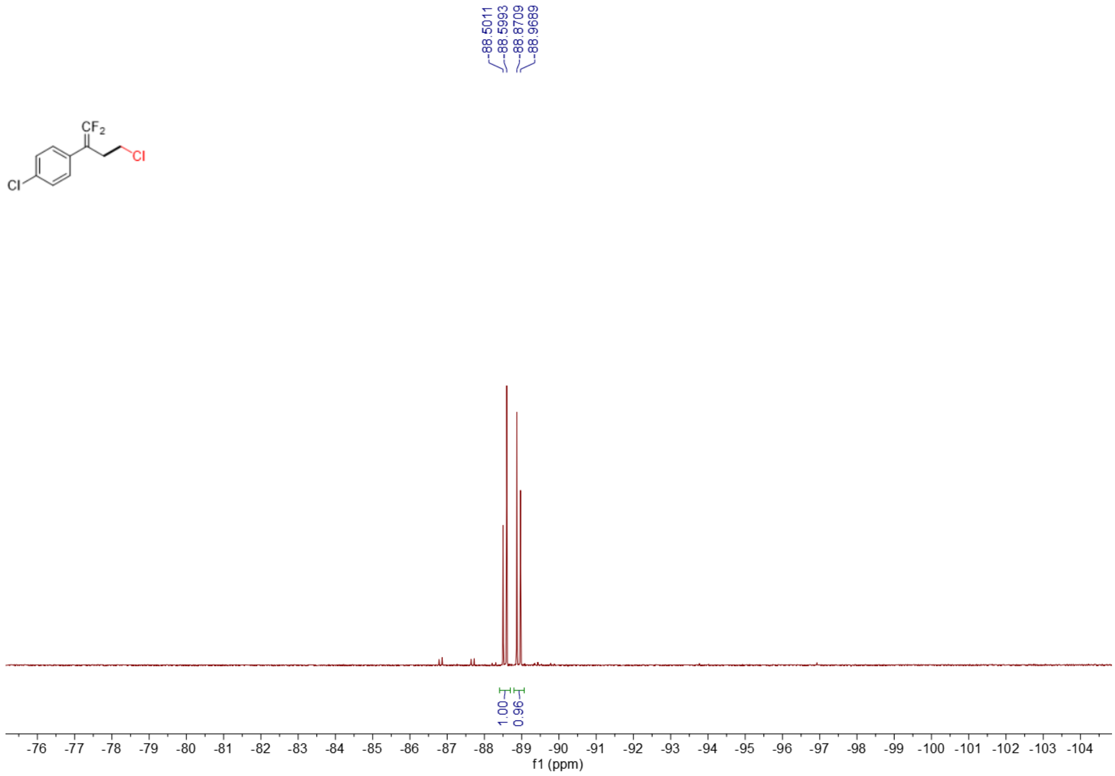

NMR spectrum of 8

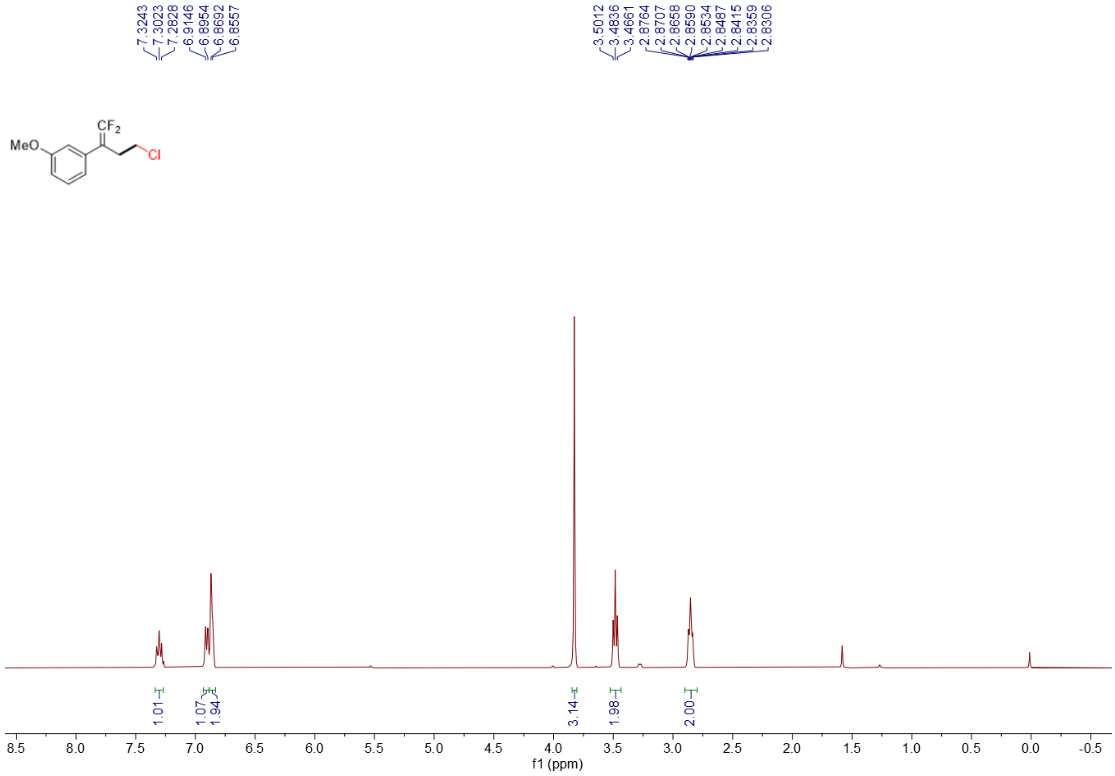

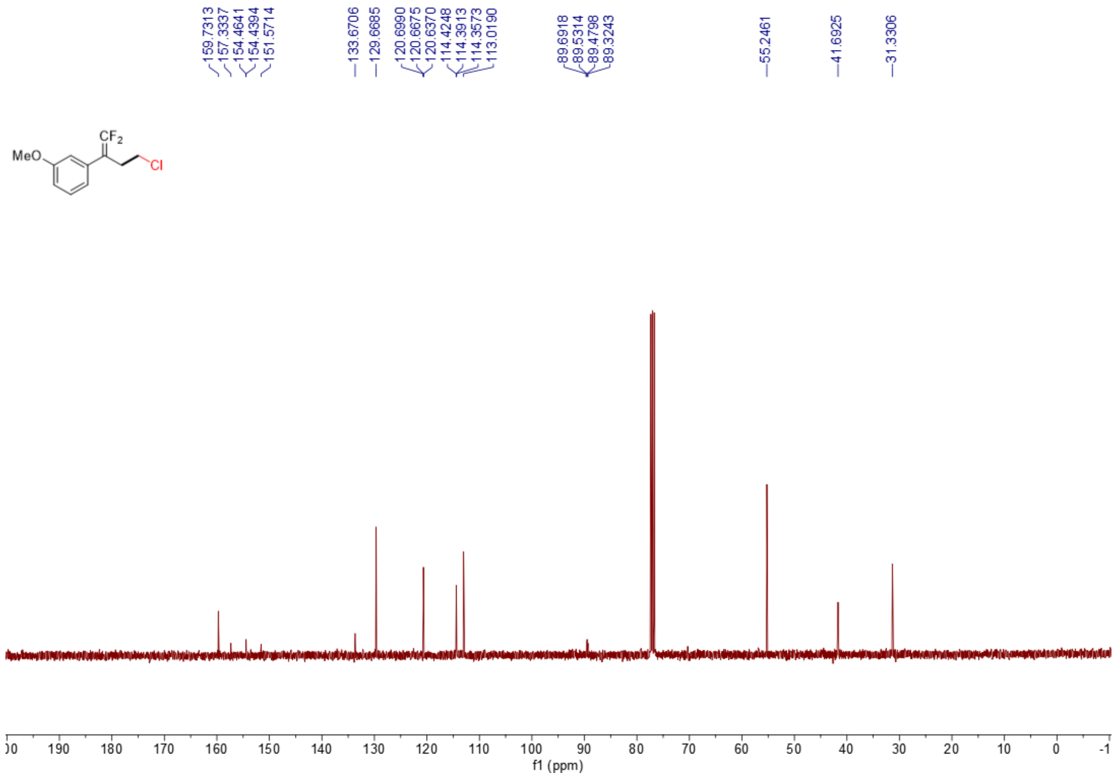
159.7313
157.3337
154.4641
154.4394
151.5714
133.6706
129.6685
120.6990
120.6675
120.6370
114.4248
114.3913
114.3573
113.0190
89.6918
89.5314
89.4798
89.3243
55.2461
41.6925
31.3306
MeO
CF2
Cl
f1 (ppm)

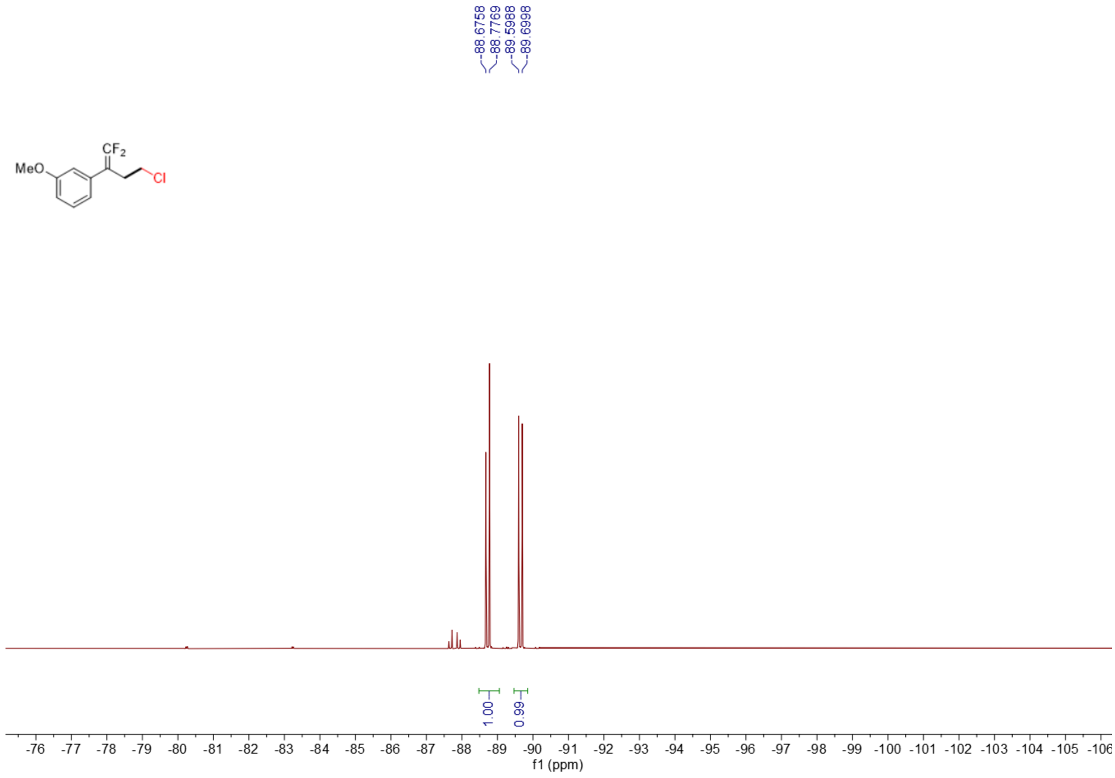
-88.6758
-88.7769
-89.5988
-89.6998
MeO
CF2
Cl
1.00
0.99
f1 (ppm)

NMR spectrum of 9

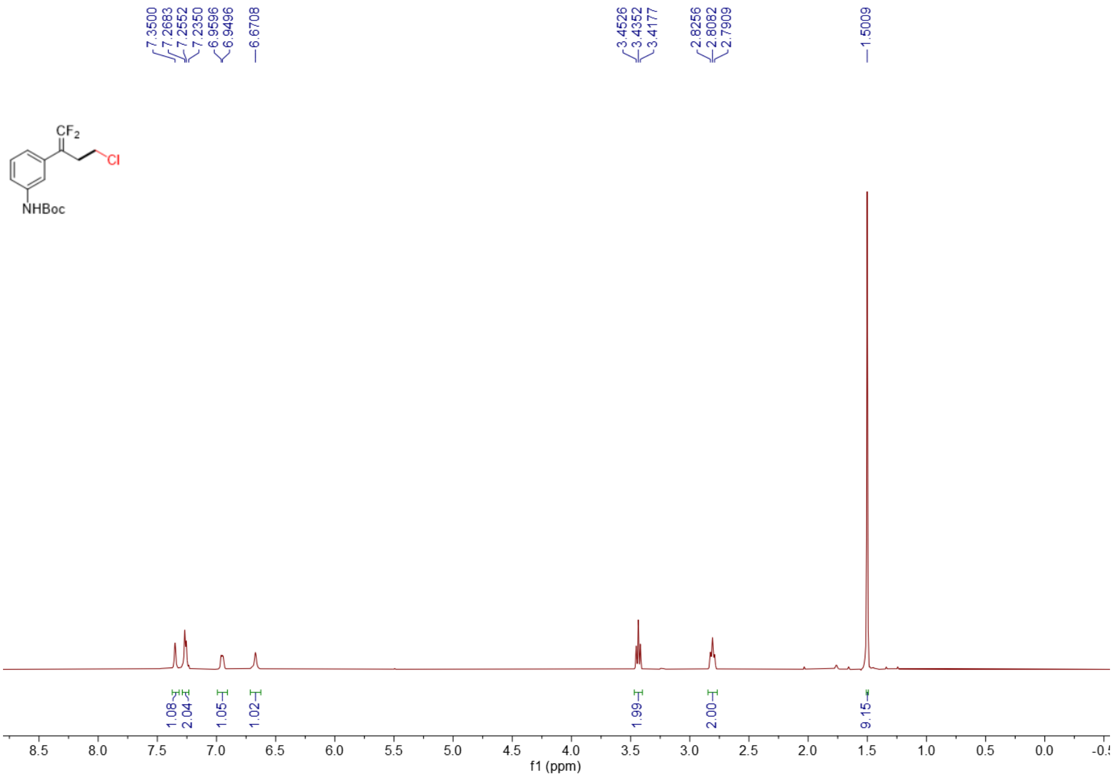

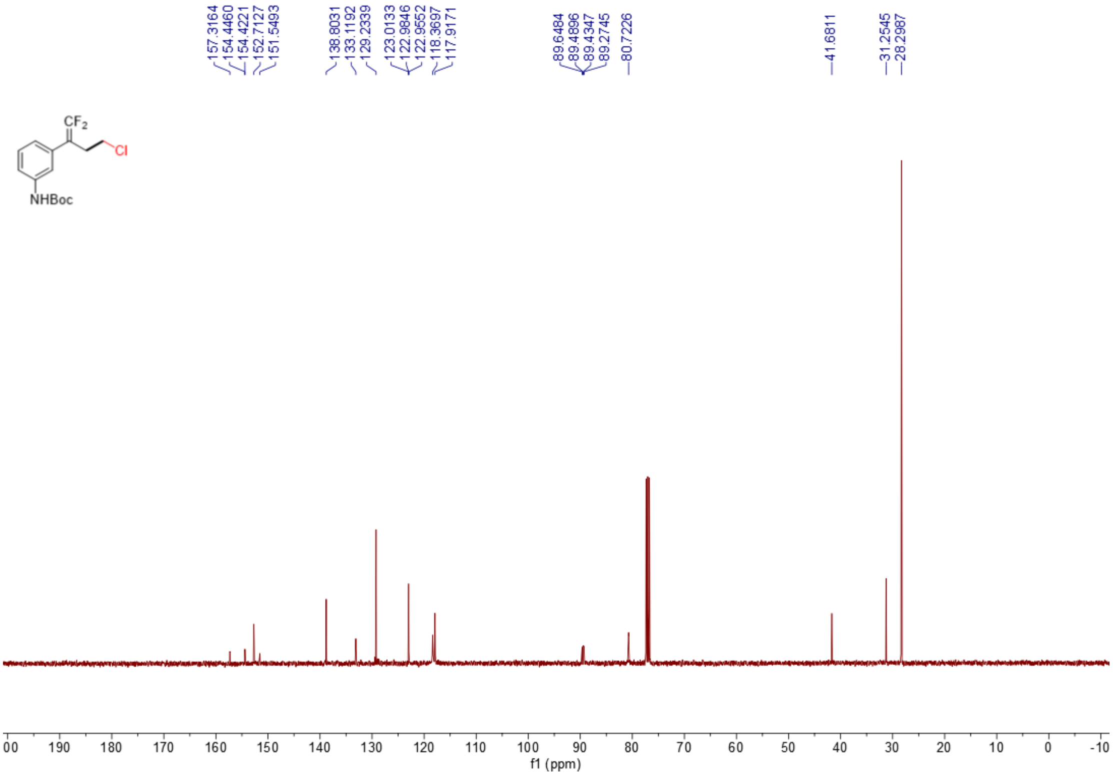

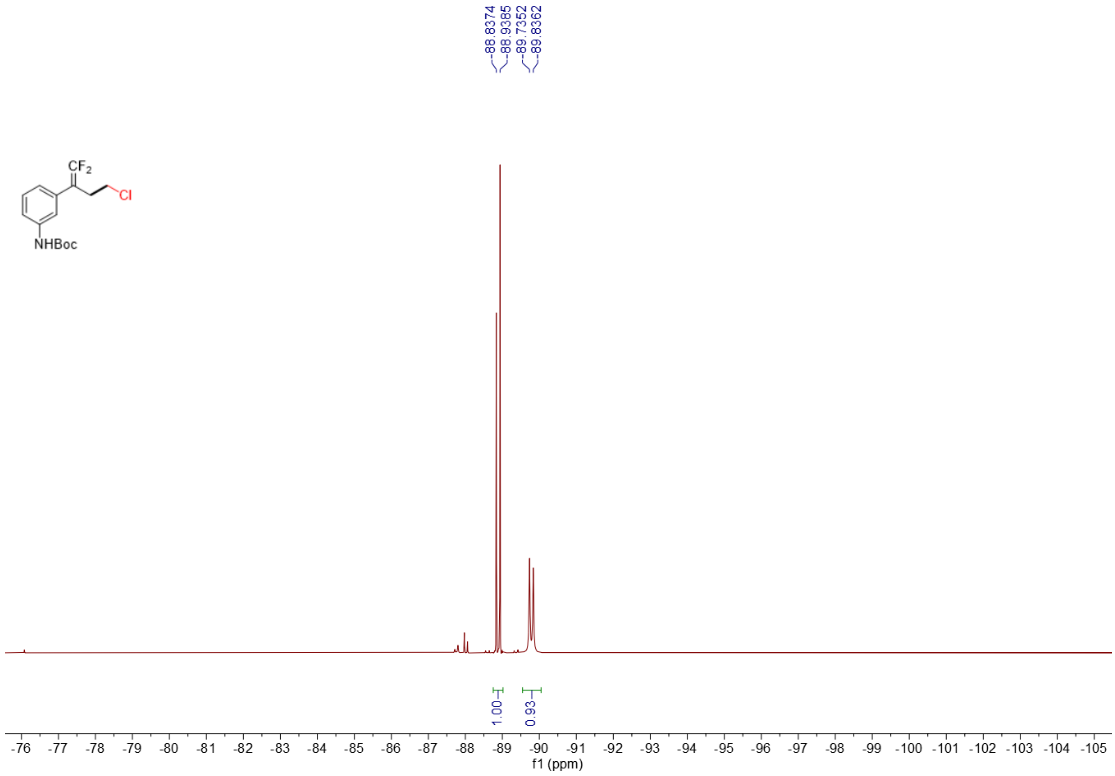

NMR spectrum of 10

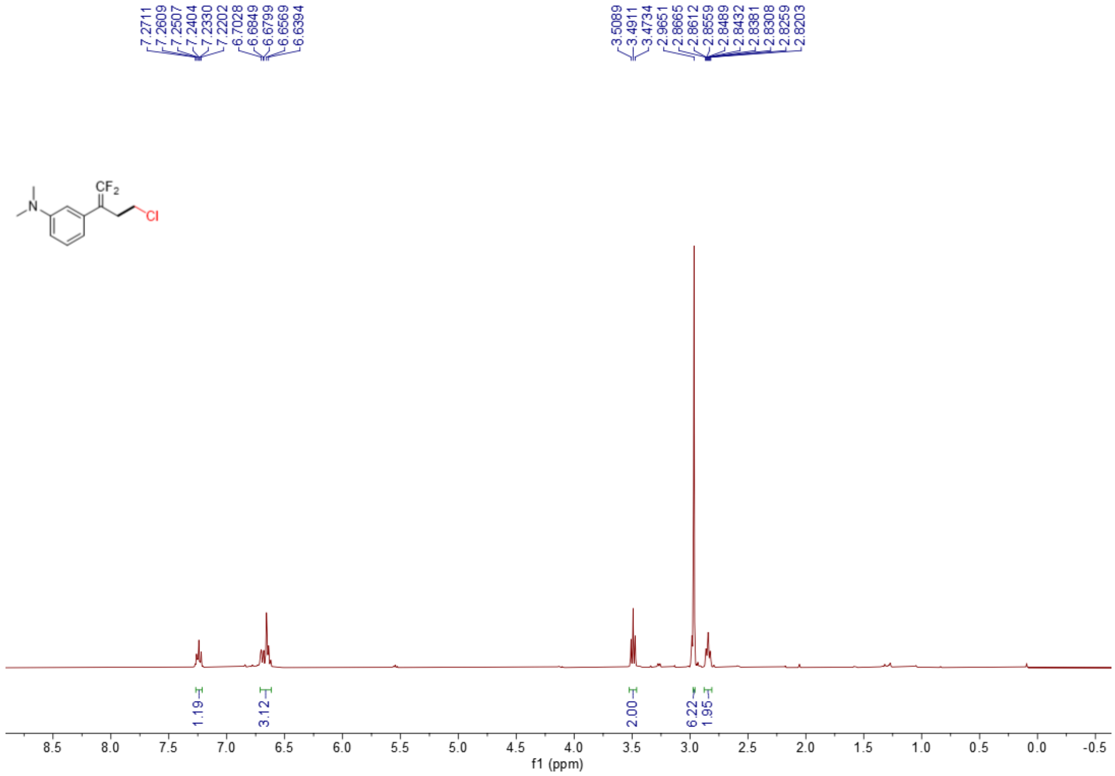

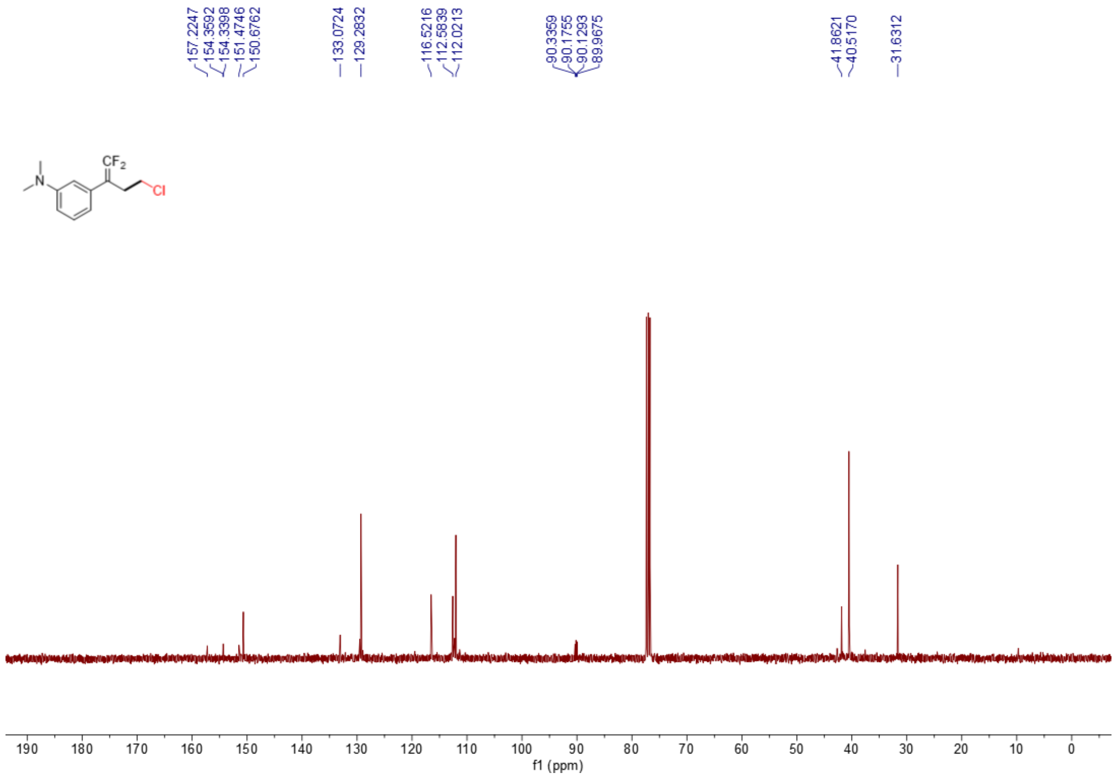
157.2247
154.3592
154.3398
151.4746
150.6762
133.0724
129.2832
116.5216
112.5839
112.0213
90.3359
90.1755
90.1293
89.9675
41.8621
40.5170
31.6312
CF2
N
Cl
190 180 170 160 150 140 130 120 110 100 90 80 70 60 50 40 30 20 10 0
f1 (ppm)

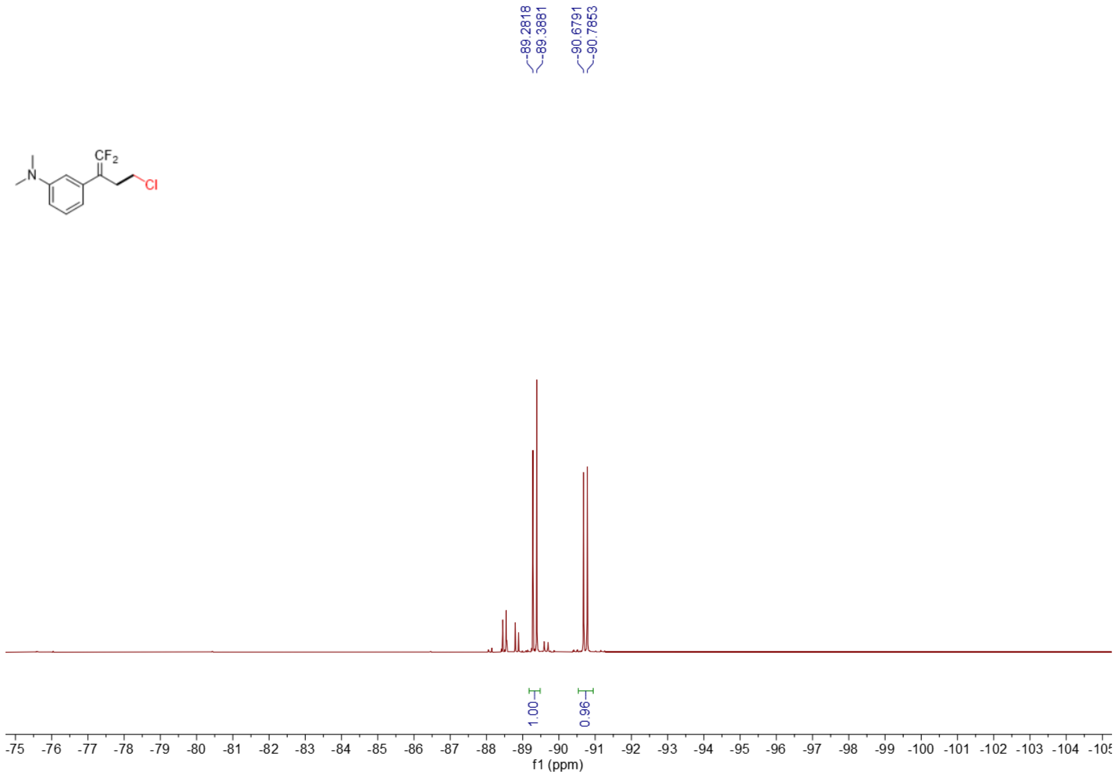
-89.2818
-89.3881
-90.6791
-90.7853
CF2
N
Cl
1.00
0.96
-75 -76 -77 -78 -79 -80 -81 -82 -83 -84 -85 -86 -87 -88 -89 -90 -91 -92 -93 -94 -95 -96 -97 -98 -99 -100 -101 -102 -103 -104
f1 (ppm)

NMR spectrum of 11

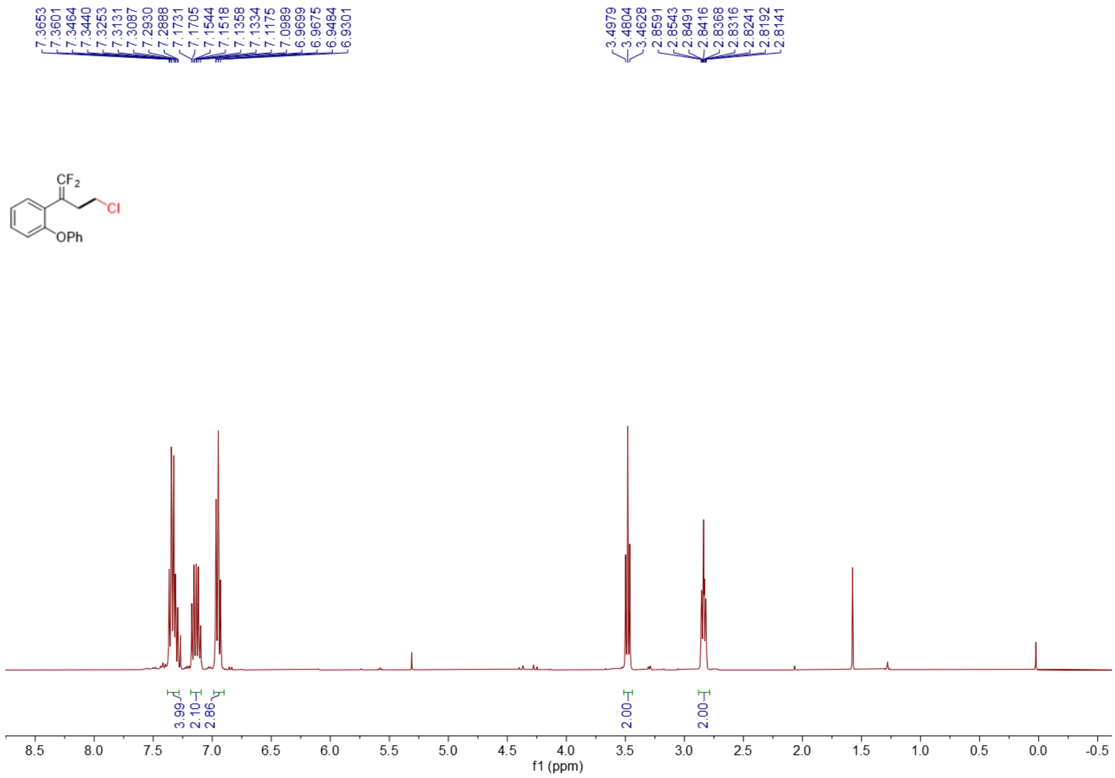

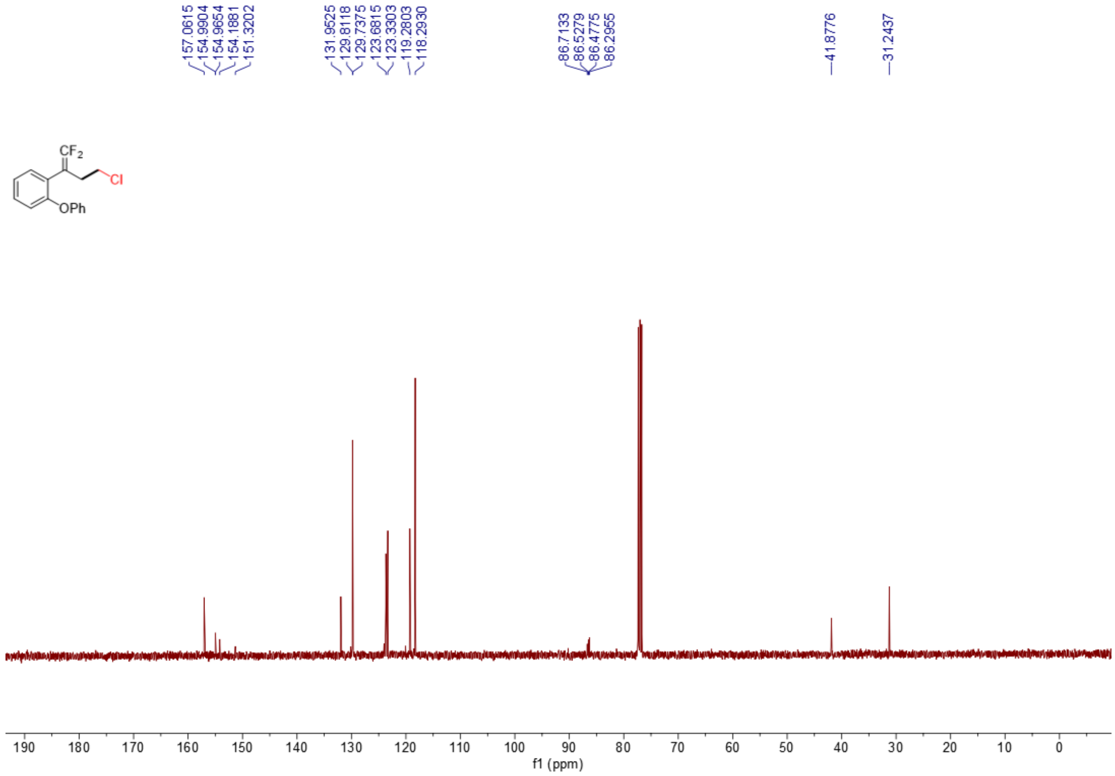

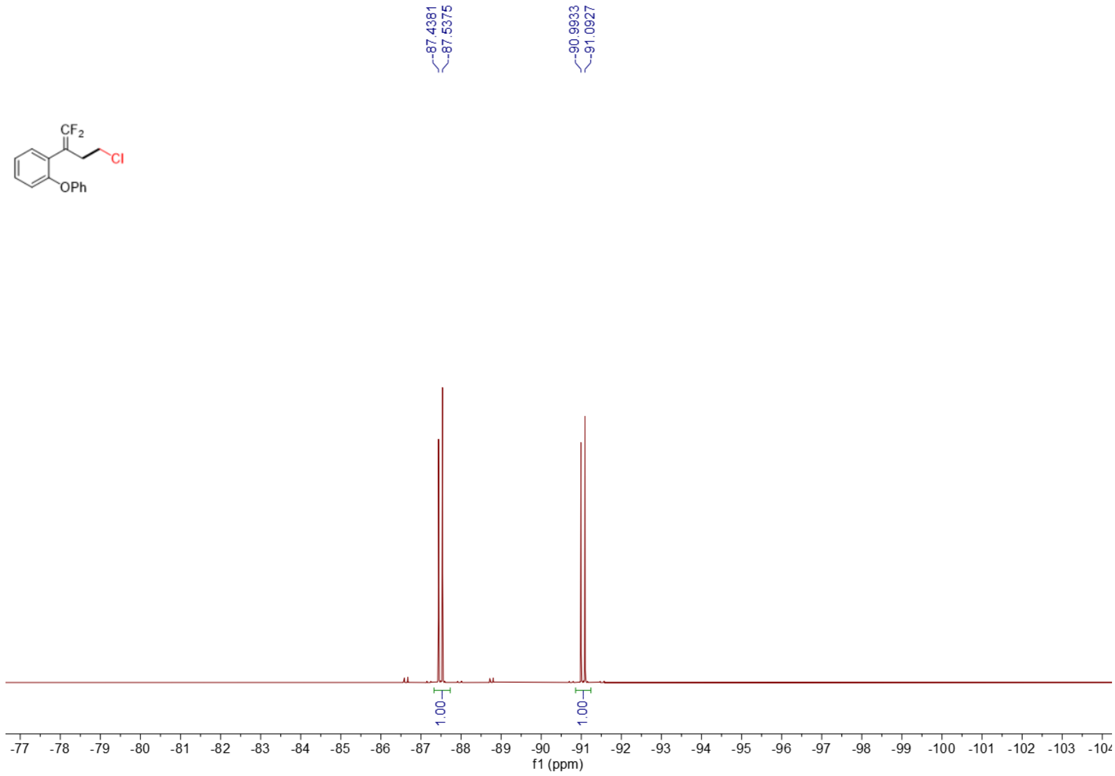

NMR spectrum of 12

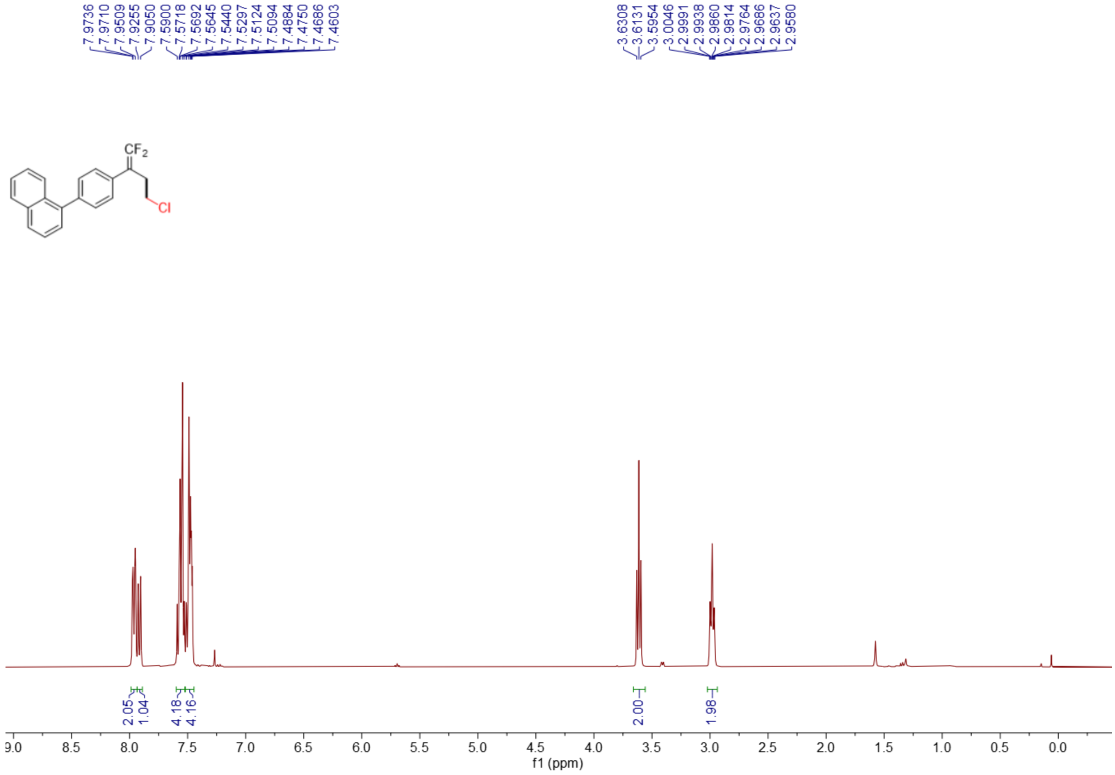

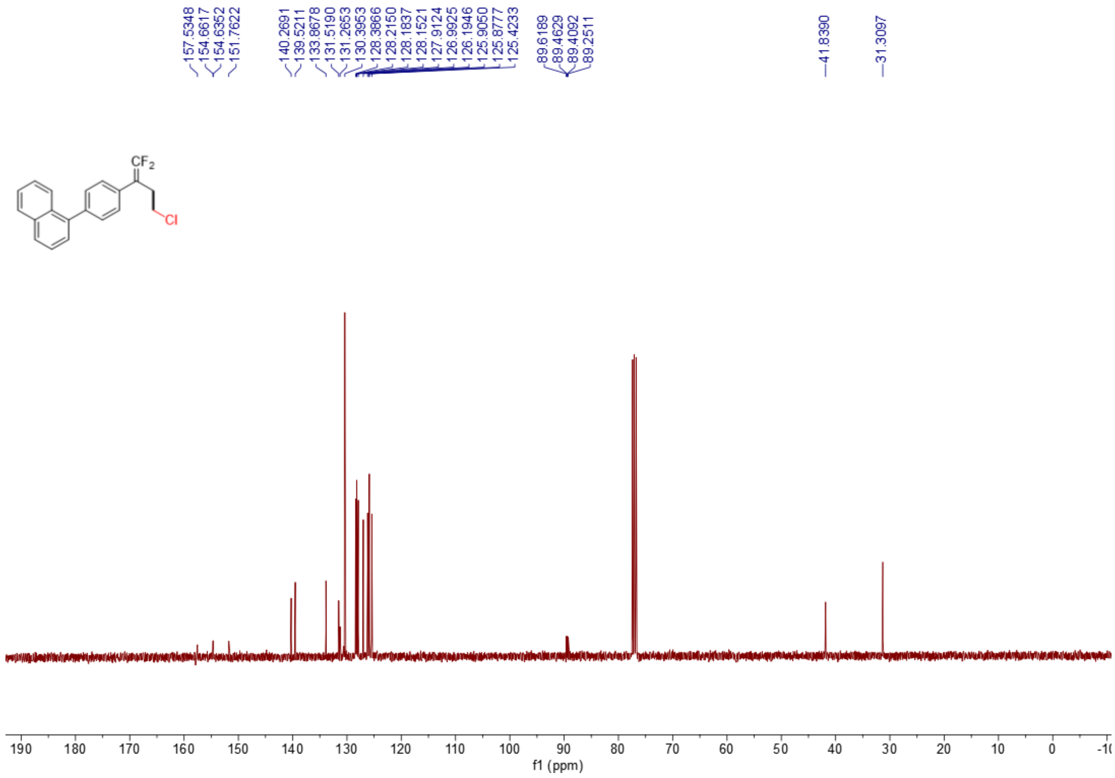
157.5348
154.6617
154.6352
151.7622
140.2691
139.5211
133.8678
131.5190
131.2653
130.3953
128.3866
128.2150
128.1837
128.1521
127.9124
126.9925
126.1946
125.9050
125.8777
125.4233
89.6189
89.4629
89.4092
89.2511
41.8390
31.3097
CF2
Cl
f1 (ppm)

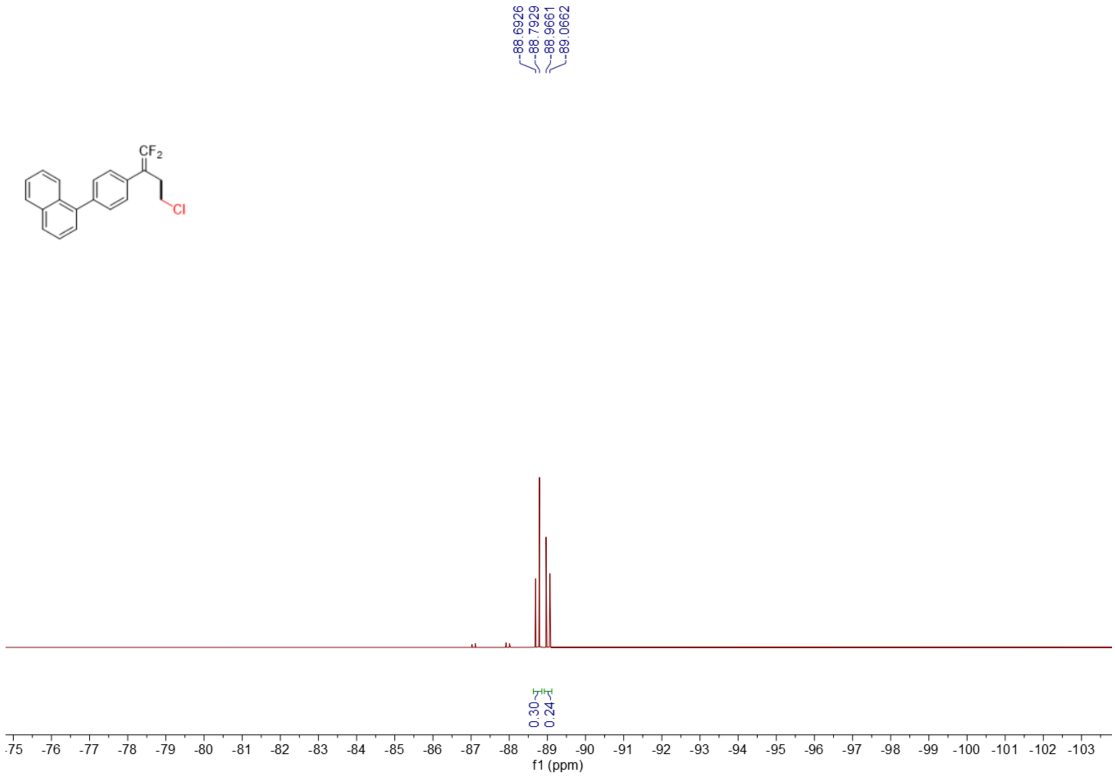
-88.6926
-88.7929
-88.9661
-89.0662
CF2
Cl
0.30
0.24
f1 (ppm)

NMR spectrum of 13

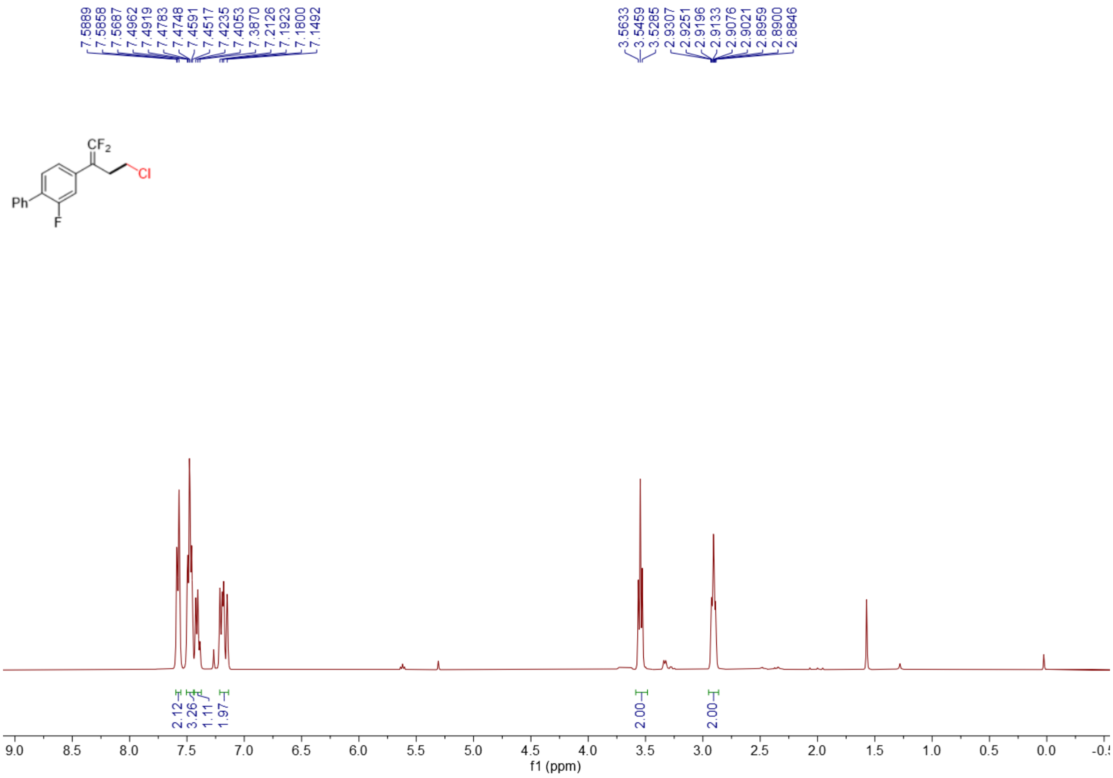

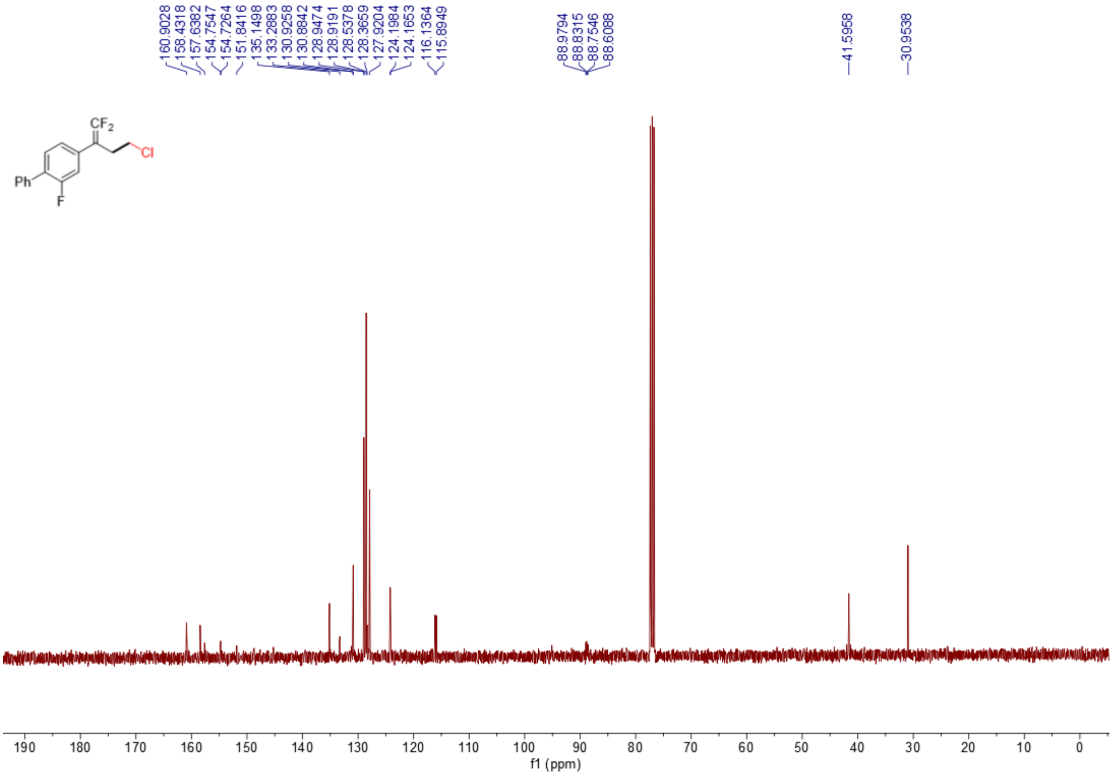

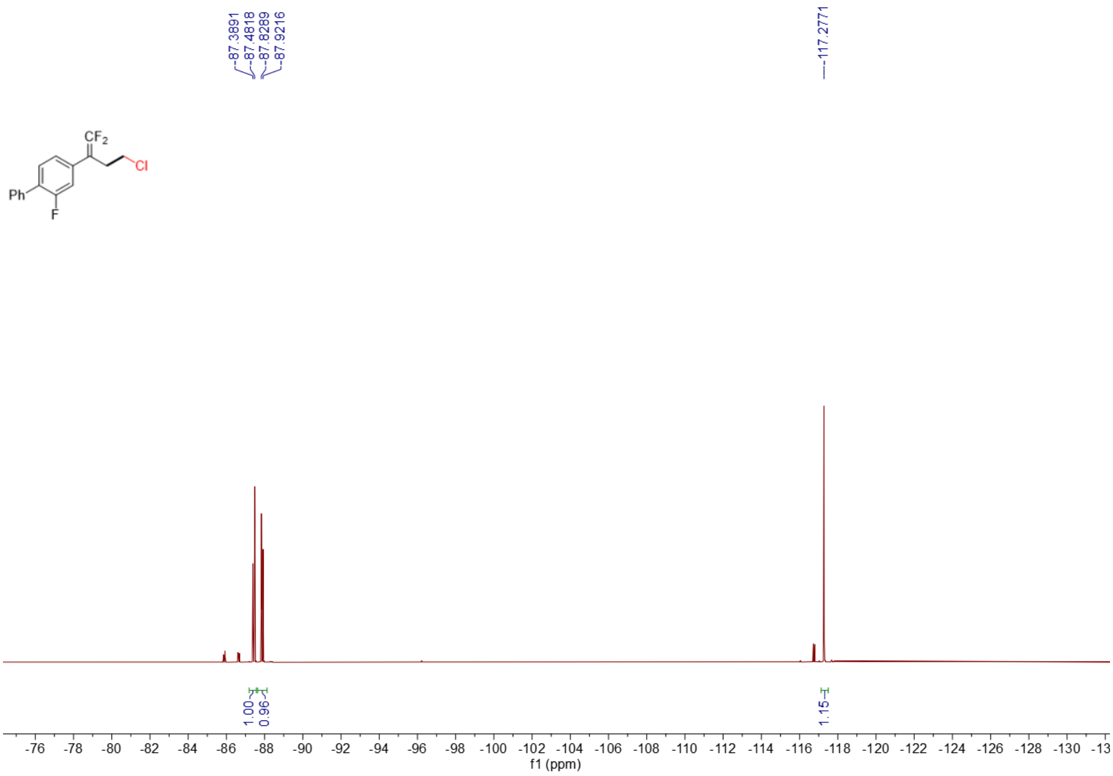

NMR spectrum of 14

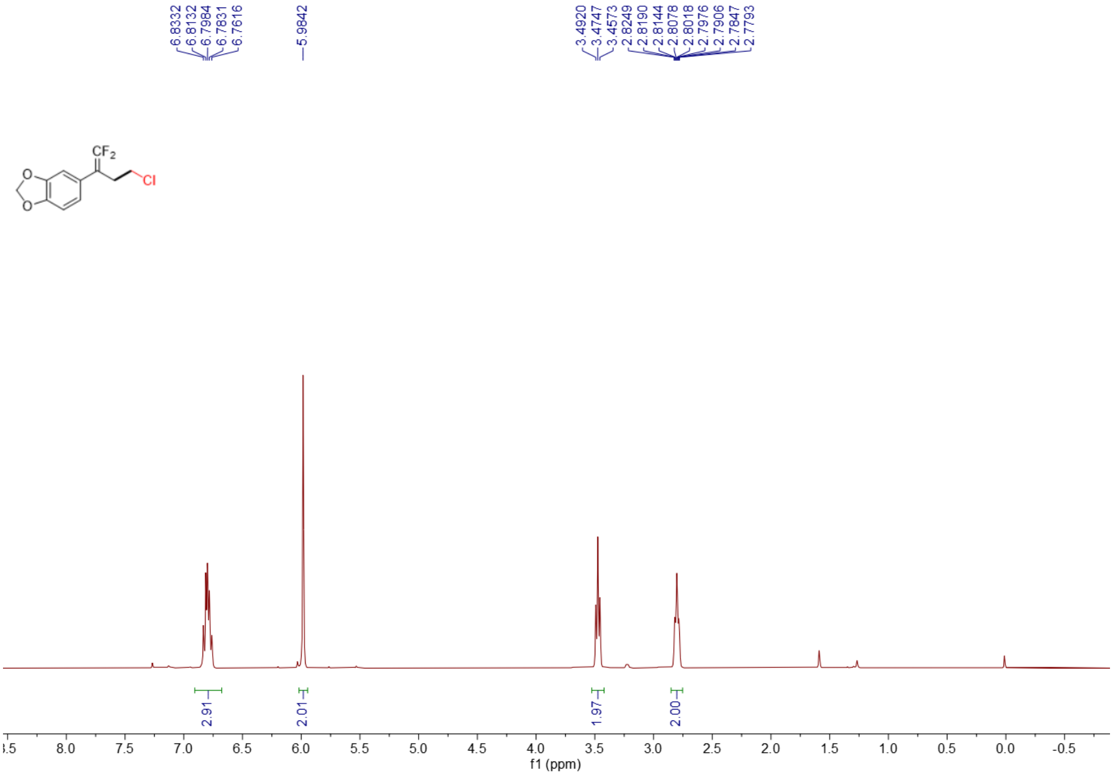

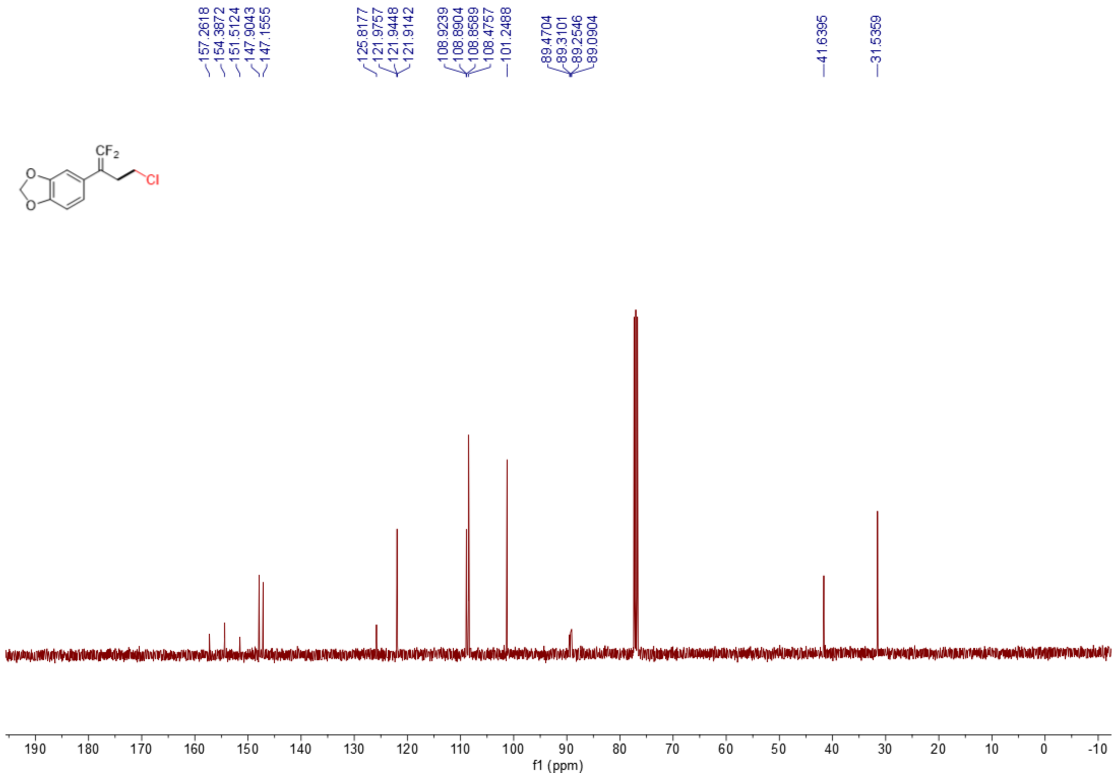
157.2618
154.3872
151.5124
147.9043
147.1555
125.8177
121.9757
121.9448
121.9142
108.9239
108.8904
108.8589
108.4757
101.2488
89.4704
89.3101
89.2546
89.0904
41.6395
31.5359
CF2
O
O
Cl
190 180 170 160 150 140 130 120 110 100 90 80 70 60 50 40 30 20 10 0 -10
f1 (ppm)

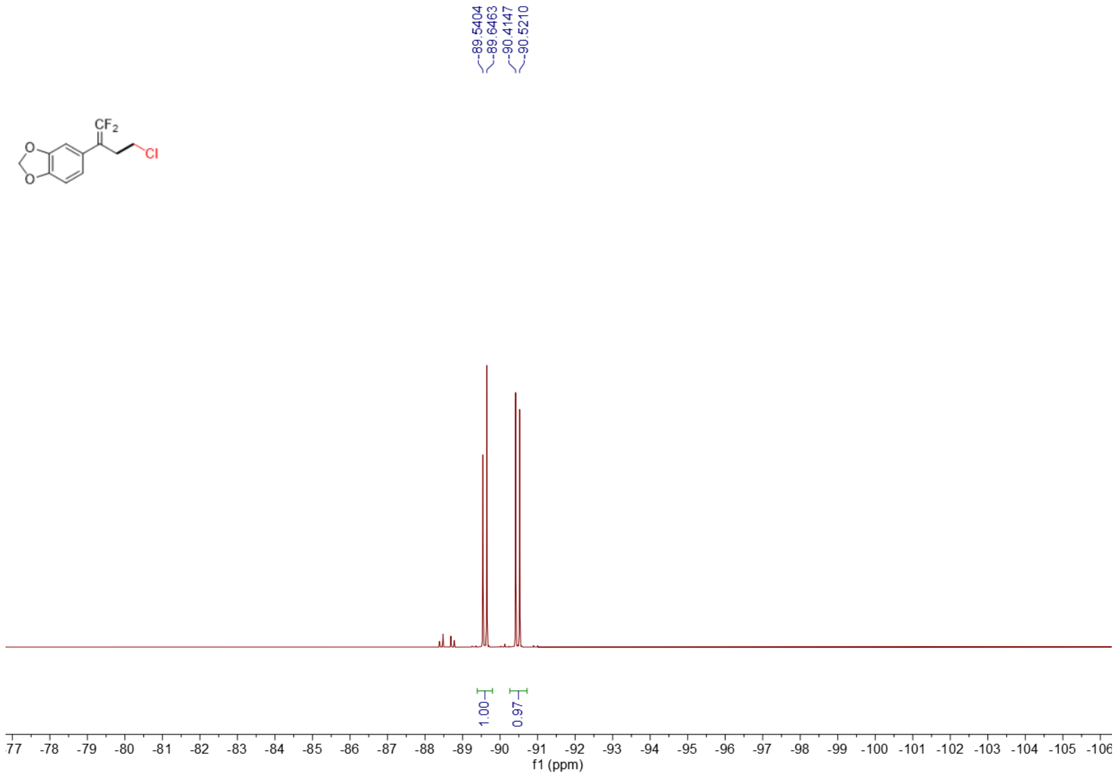
-89.5404
-89.6463
-90.4147
-90.5210
CF2
O
O
Cl
1.00
0.97
-77 -78 -79 -80 -81 -82 -83 -84 -85 -86 -87 -88 -89 -90 -91 -92 -93 -94 -95 -96 -97 -98 -99 -100 -101 -102 -103 -104 -105 -106
f1 (ppm)

NMR spectrum of 15

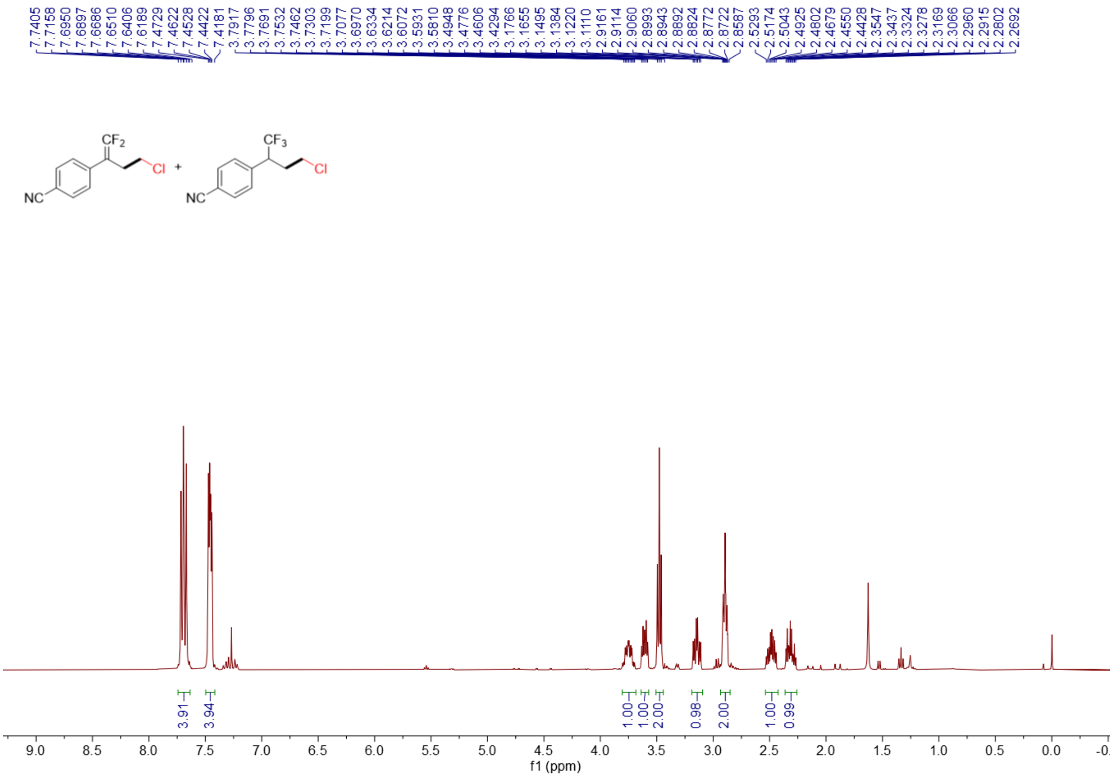

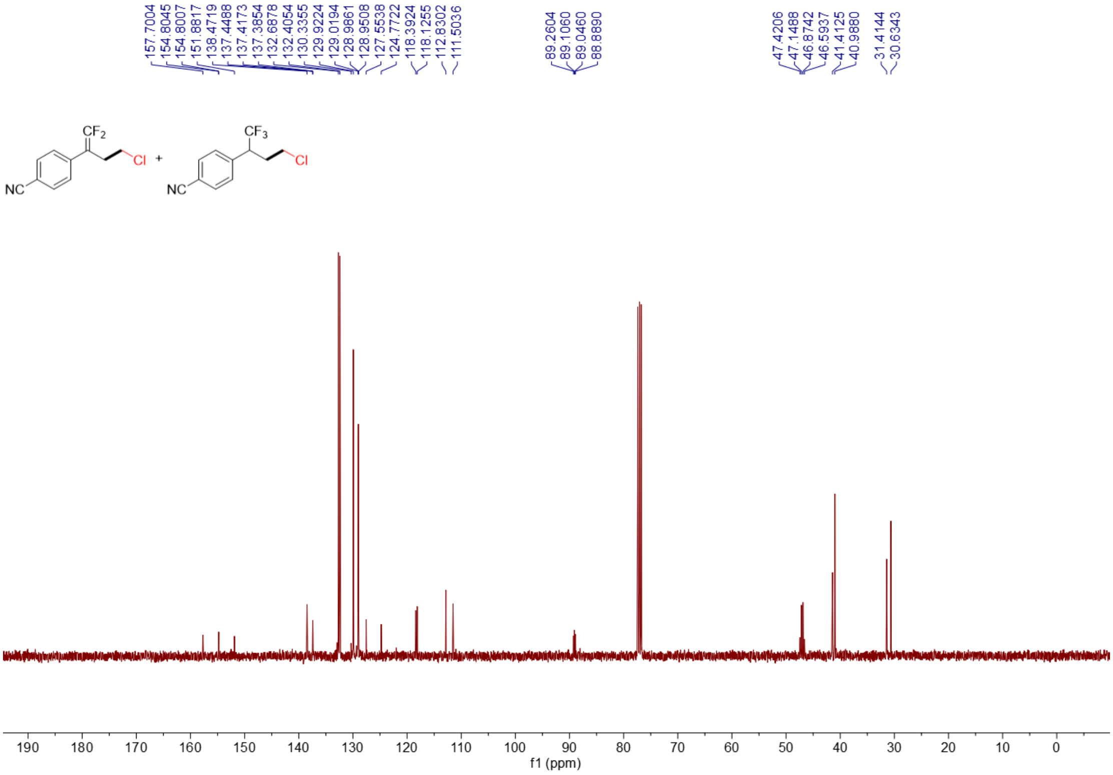

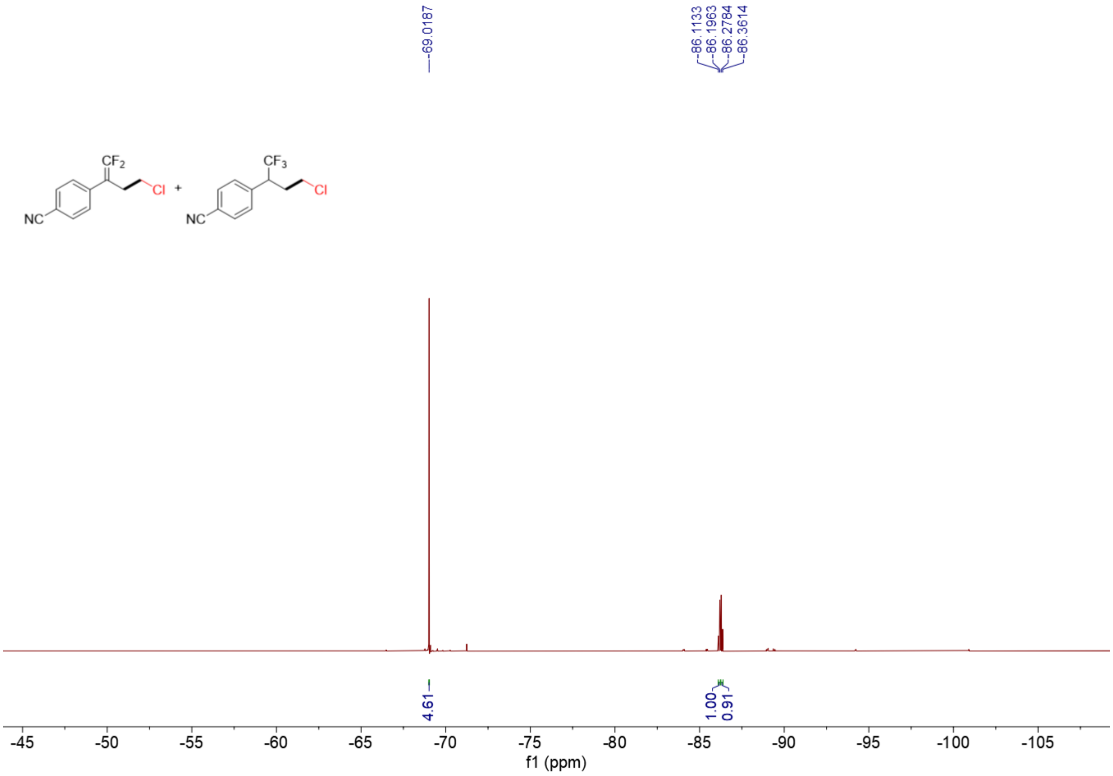

NMR spectrum of 16

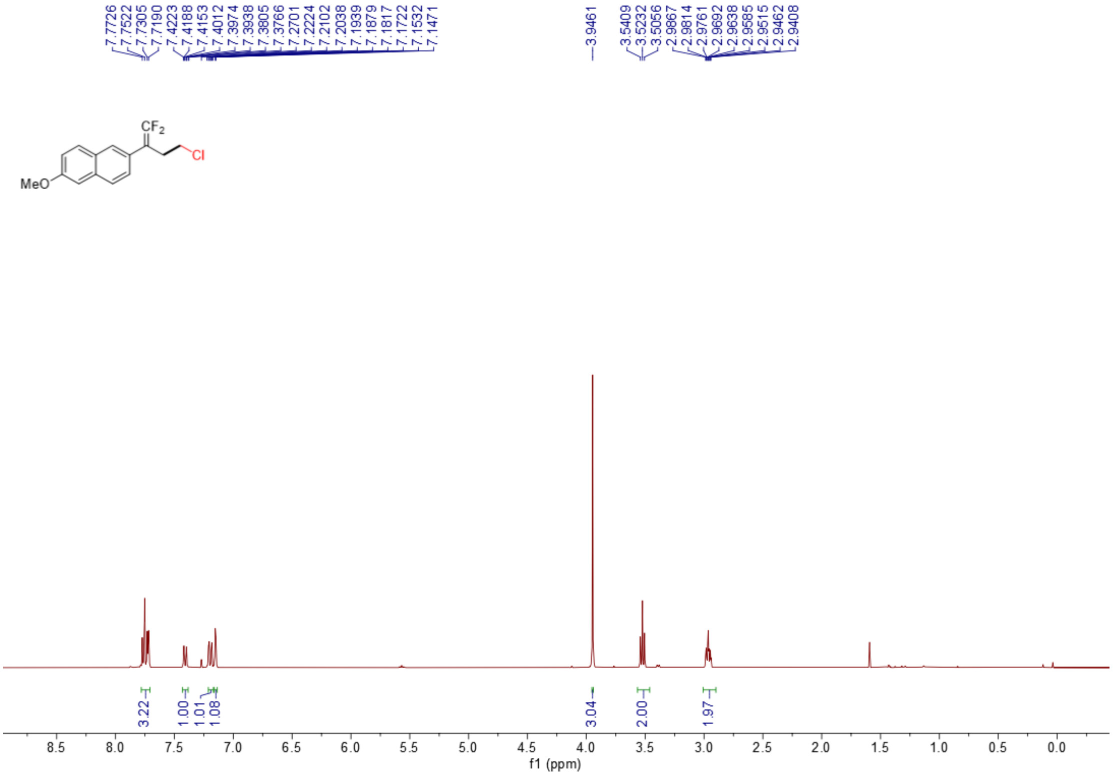

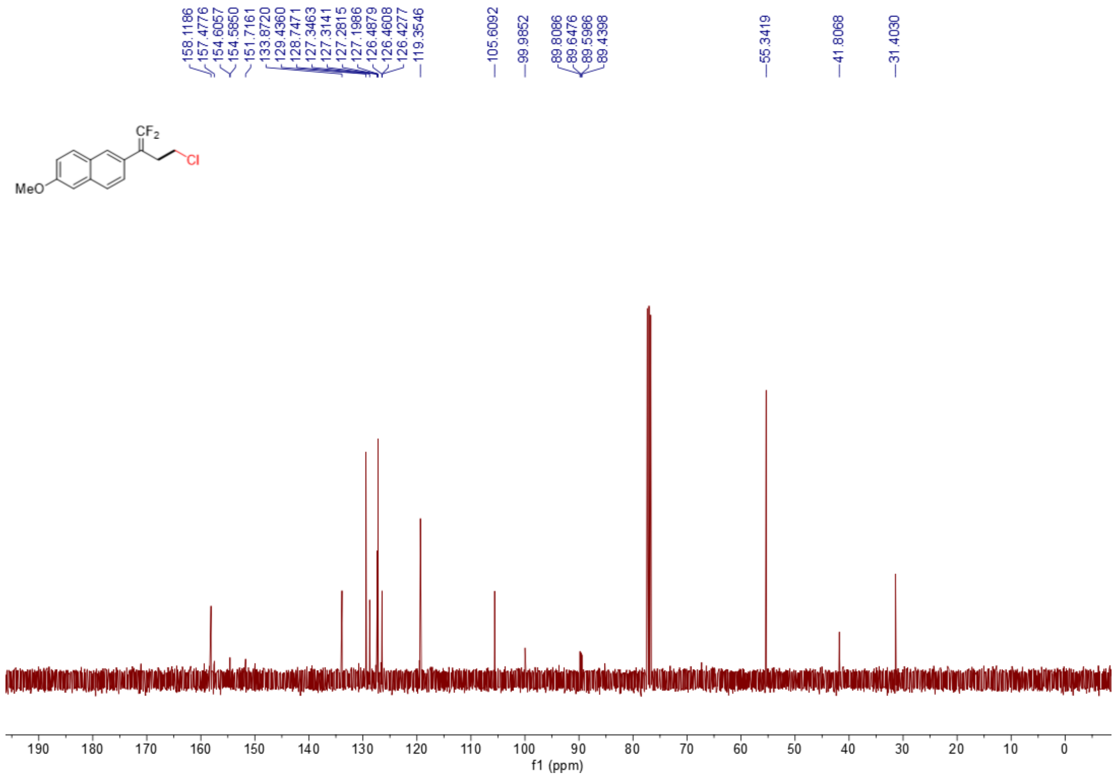
158.1186
157.4776
154.6057
154.5850
151.7161
133.8720
129.4360
128.7471
127.3463
127.3141
127.2815
127.1986
126.4879
126.4608
126.4277
119.3546
105.6092
99.9852
89.8086
89.6476
89.5986
89.4398
55.3419
41.8068
31.4030
CF2
Cl
MeO
f1 (ppm)

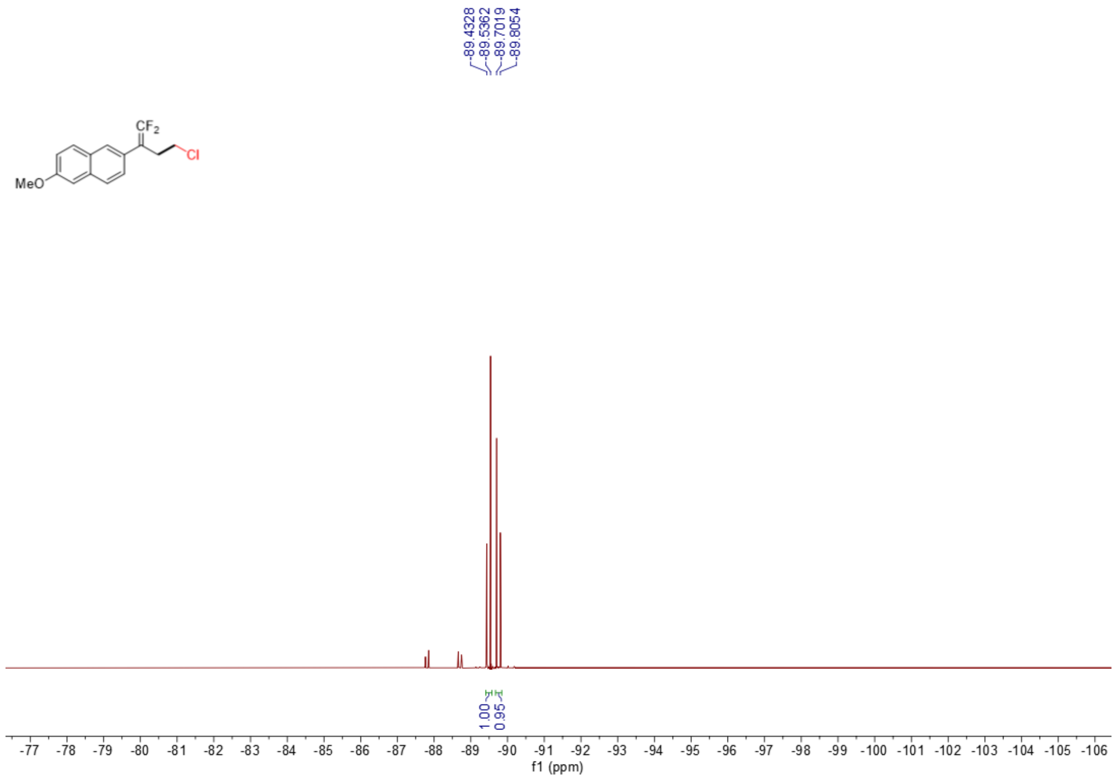
-89.4328
-89.5362
-89.7019
-89.8054
CF2
Cl
MeO
1.00
0.95
f1 (ppm)

NMR spectrum of 17

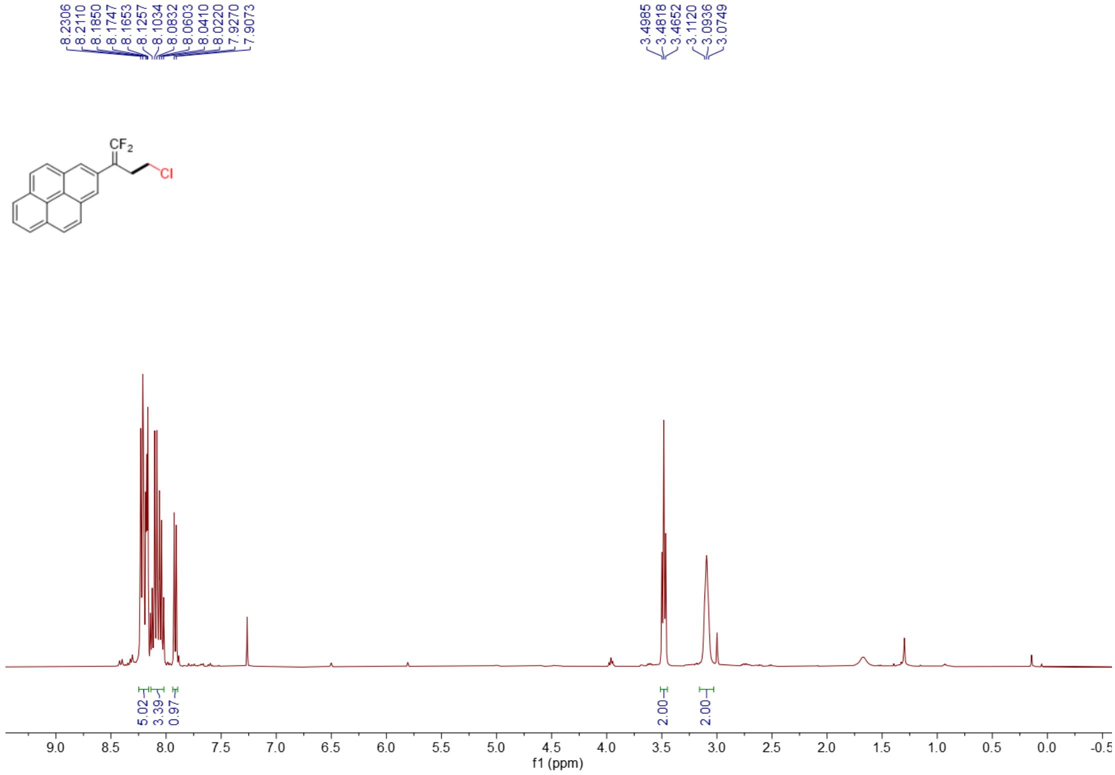

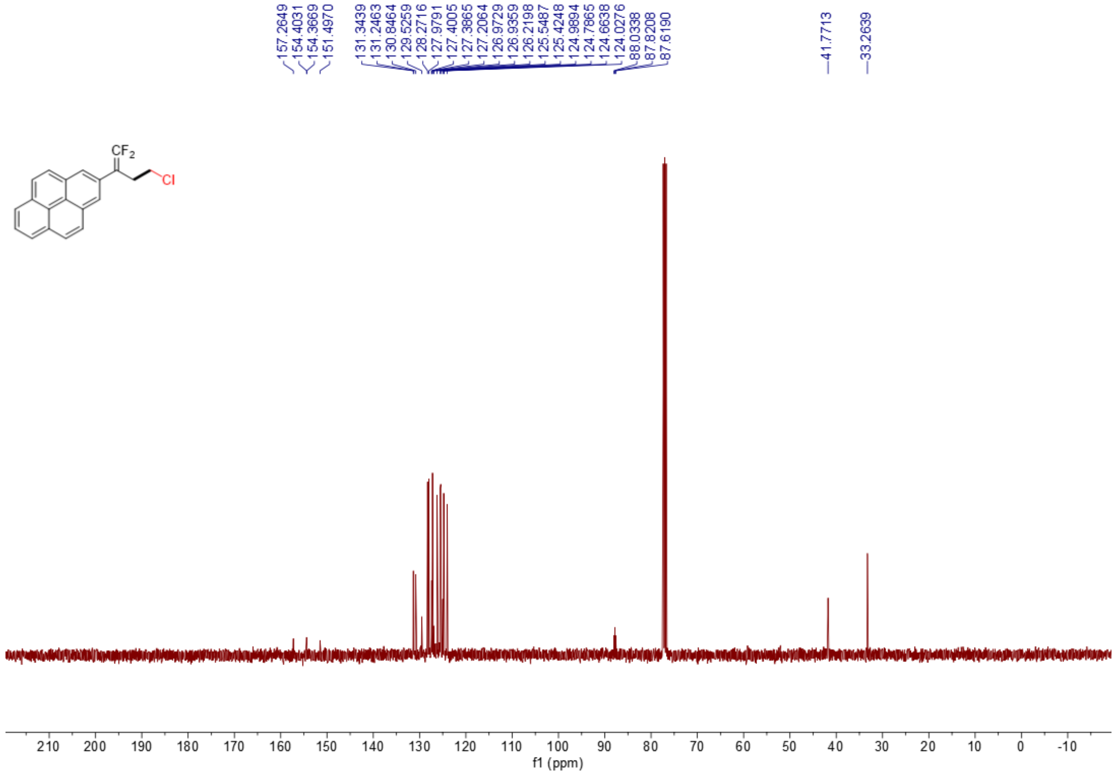

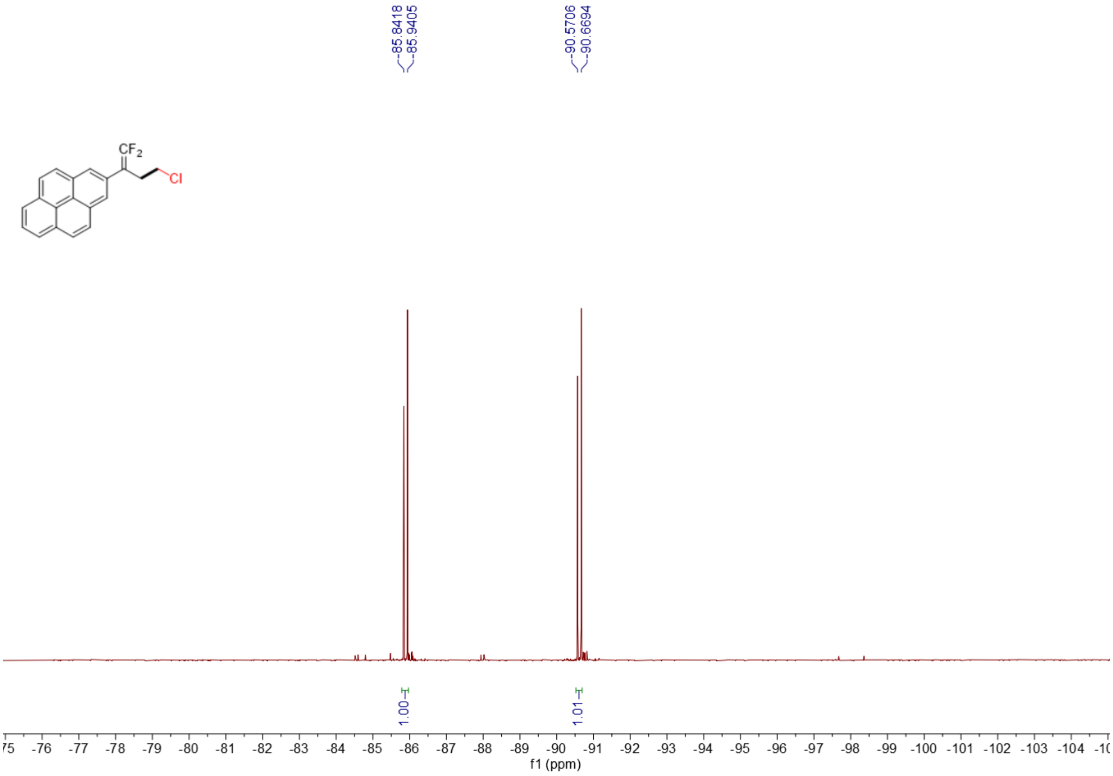

NMR spectrum of 18

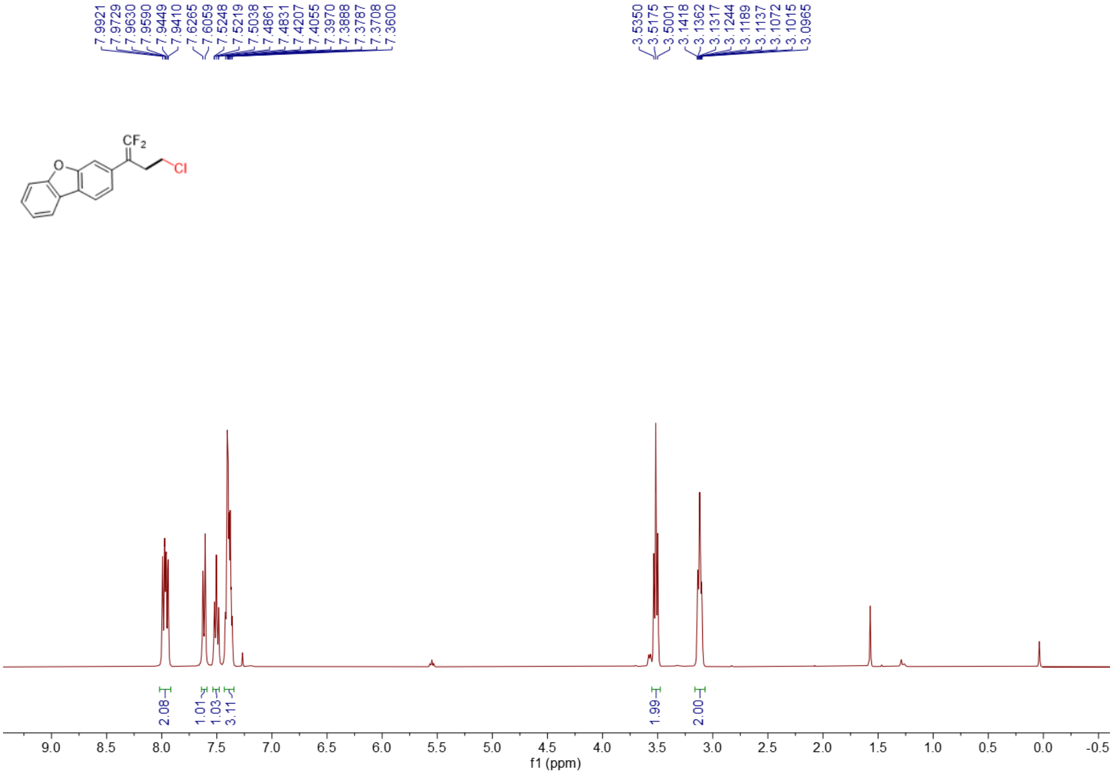

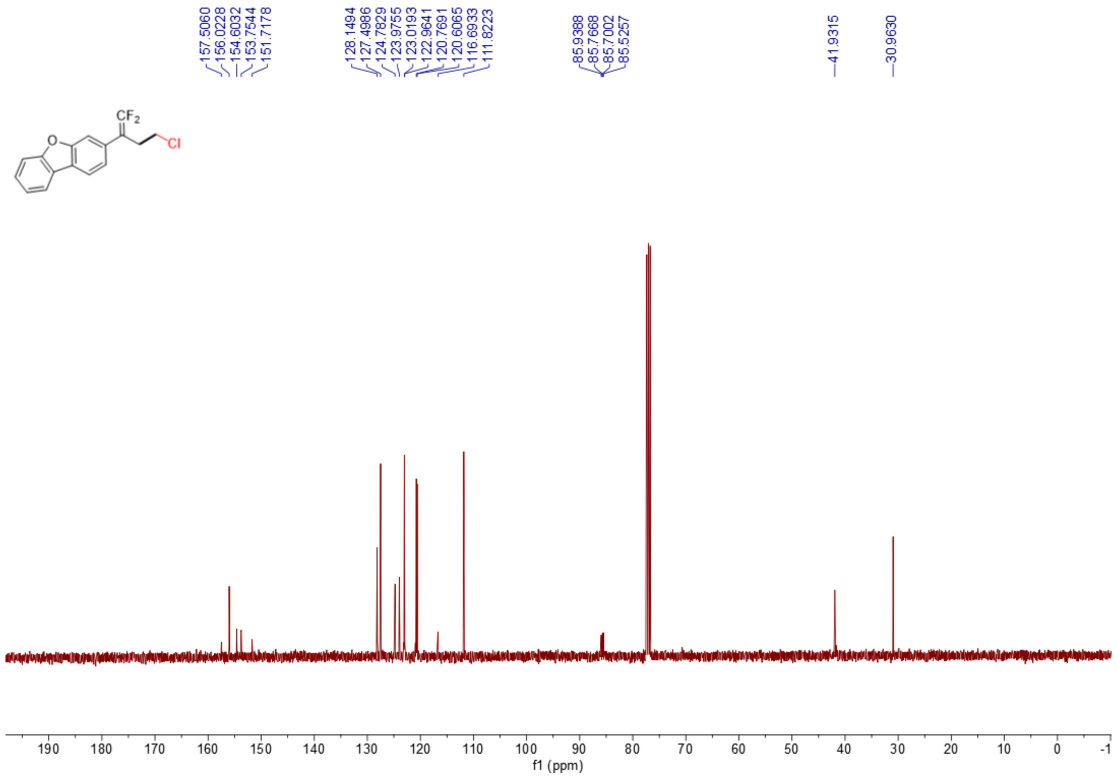
157.5060
156.0228
154.6032
153.7544
151.7178
128.1494
127.4986
124.7829
123.9755
123.0193
122.9641
120.7691
120.6065
116.6933
111.8223
85.9388
85.7668
85.7002
85.5257
41.9315
30.9630
CF2
O
Cl
f1 (ppm)

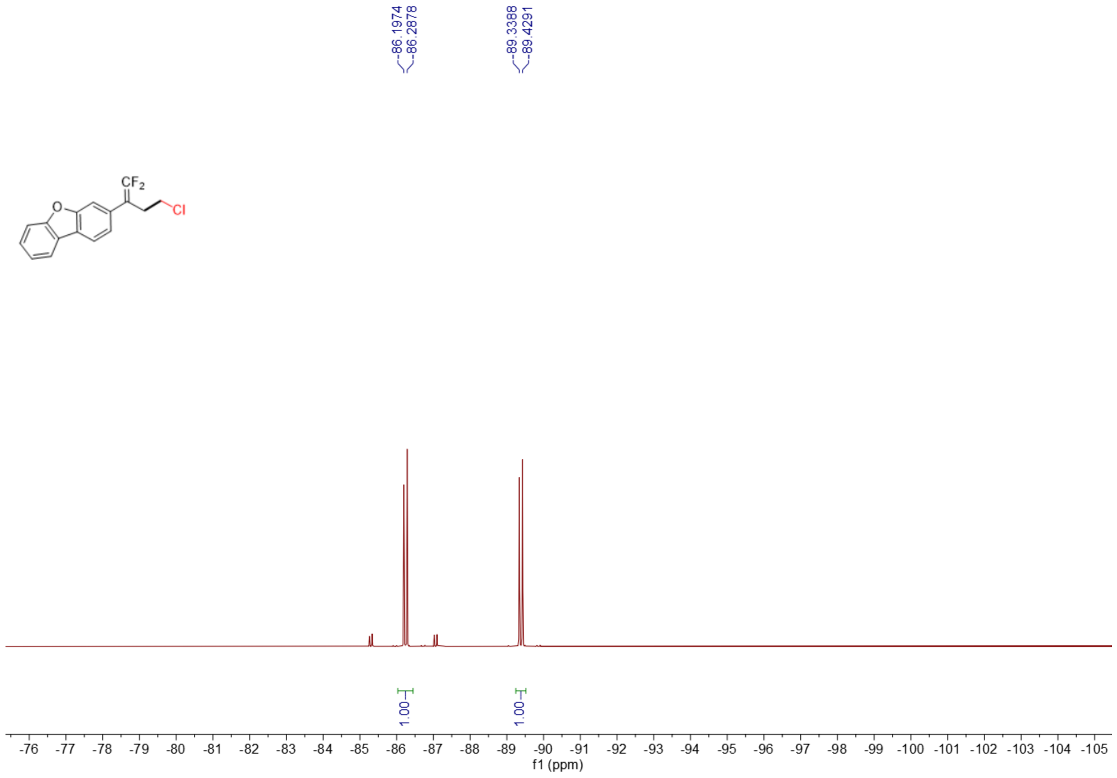
-86.1974
-86.2878
-89.3388
-89.4291
CF2
O
Cl
1.00
1.00
f1 (ppm)

NMR spectrum of 19

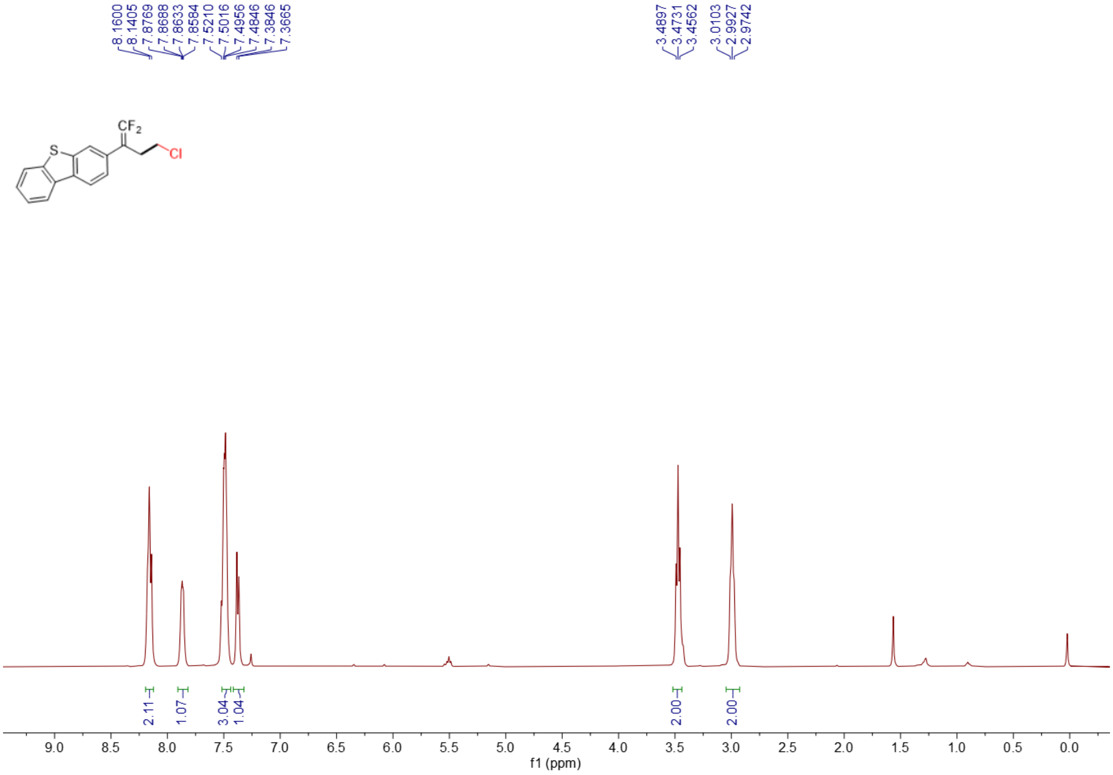

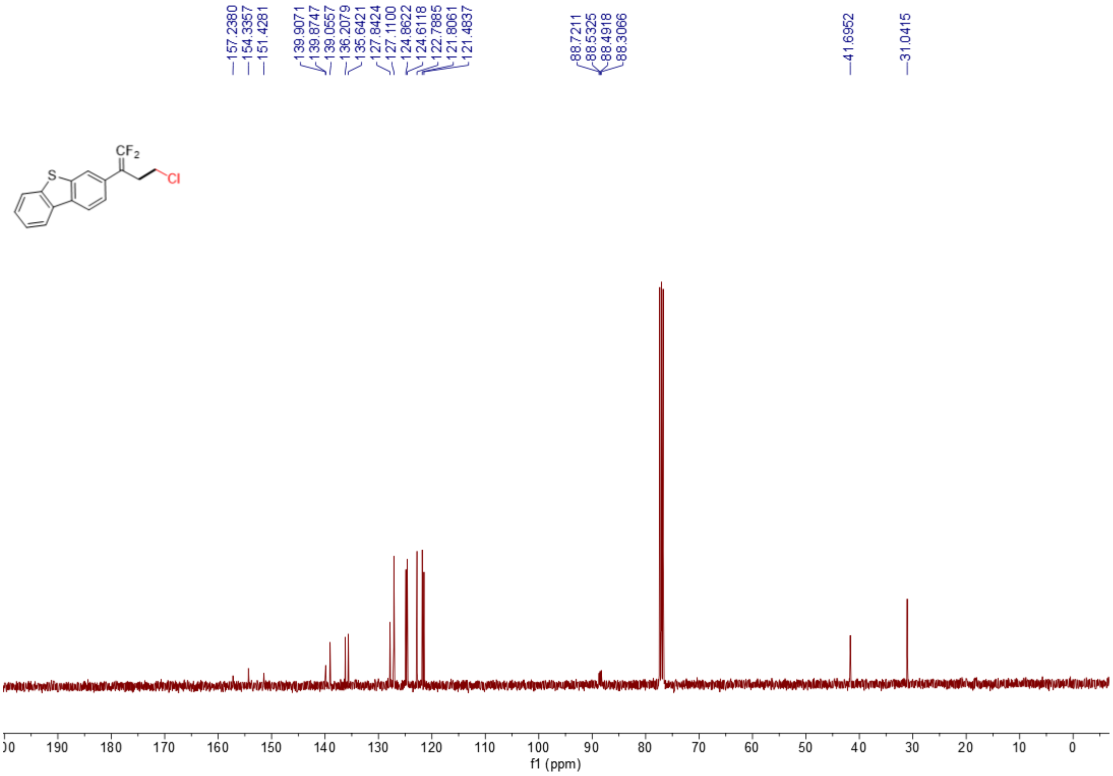

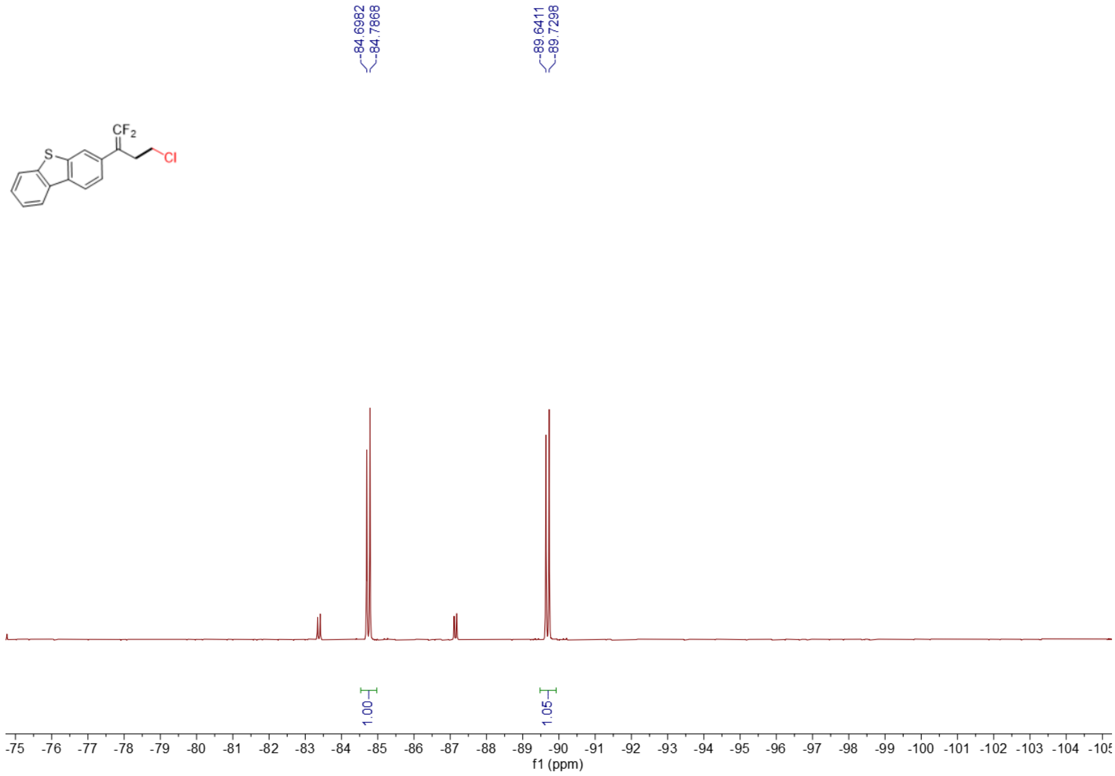

NMR spectrum of 20

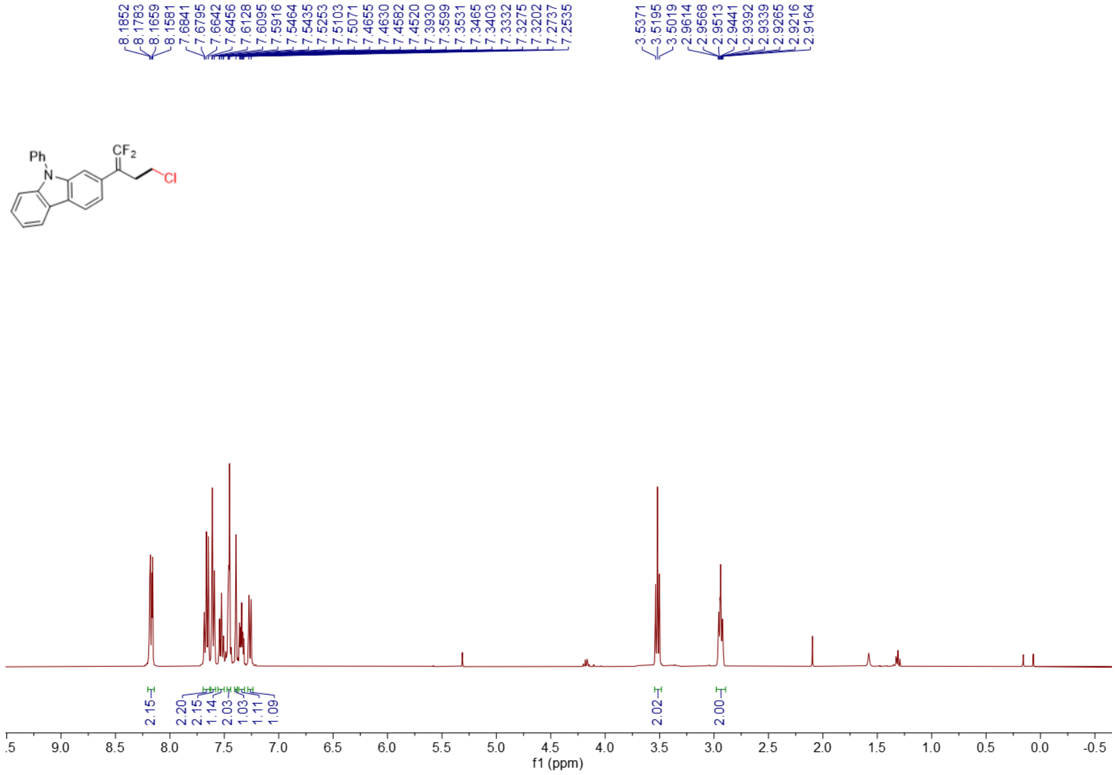

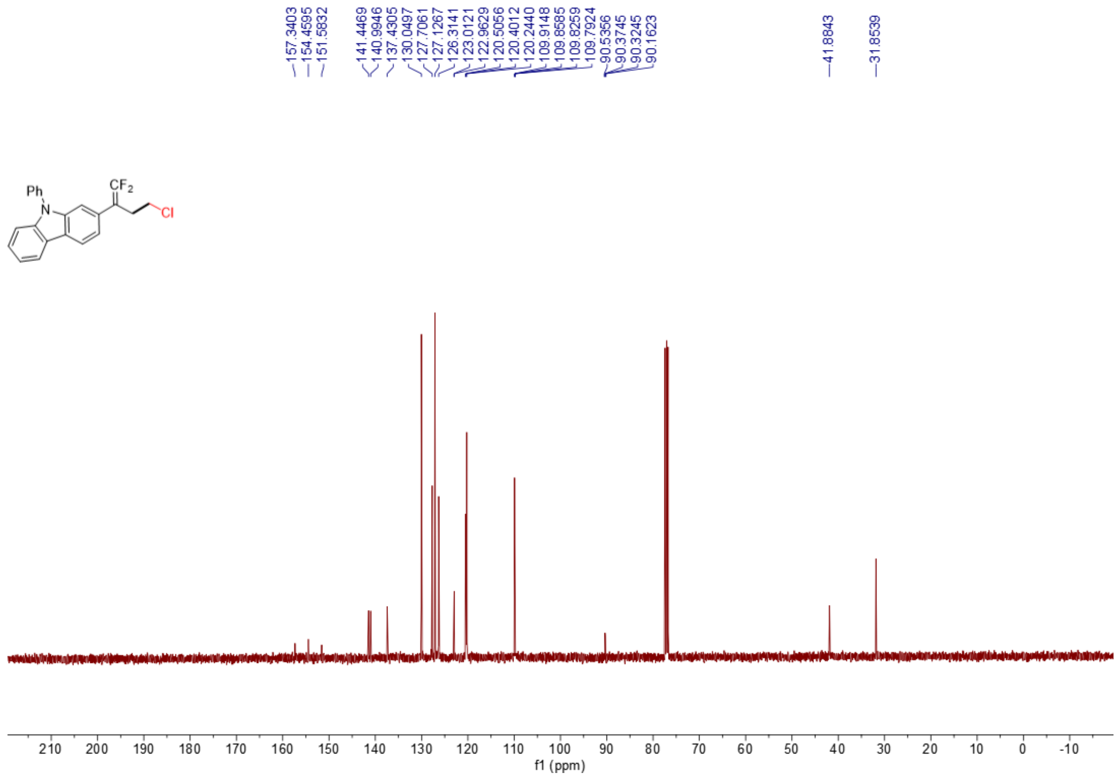

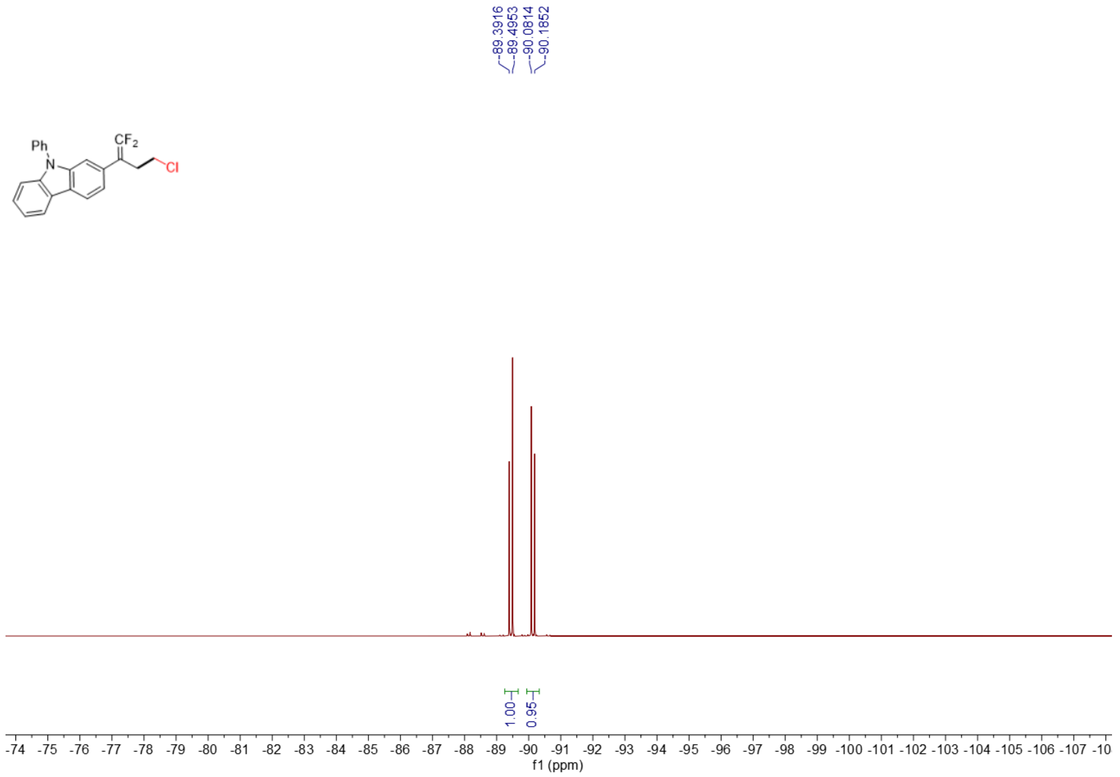

NMR spectrum of 21

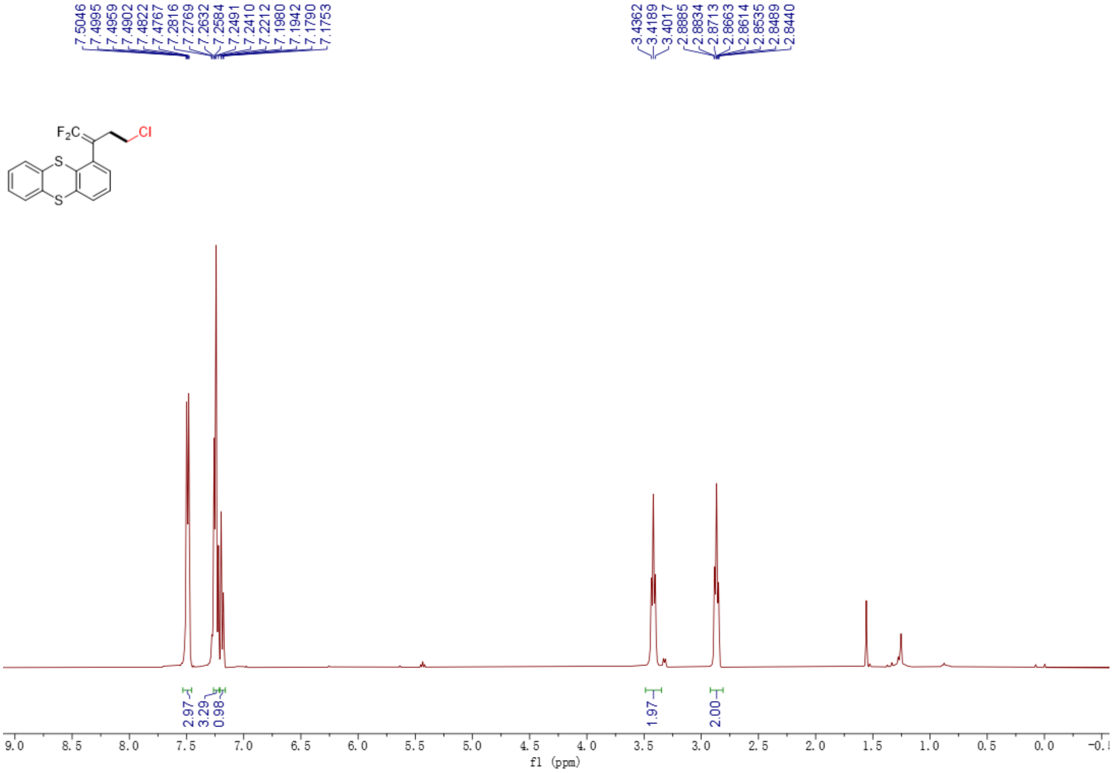

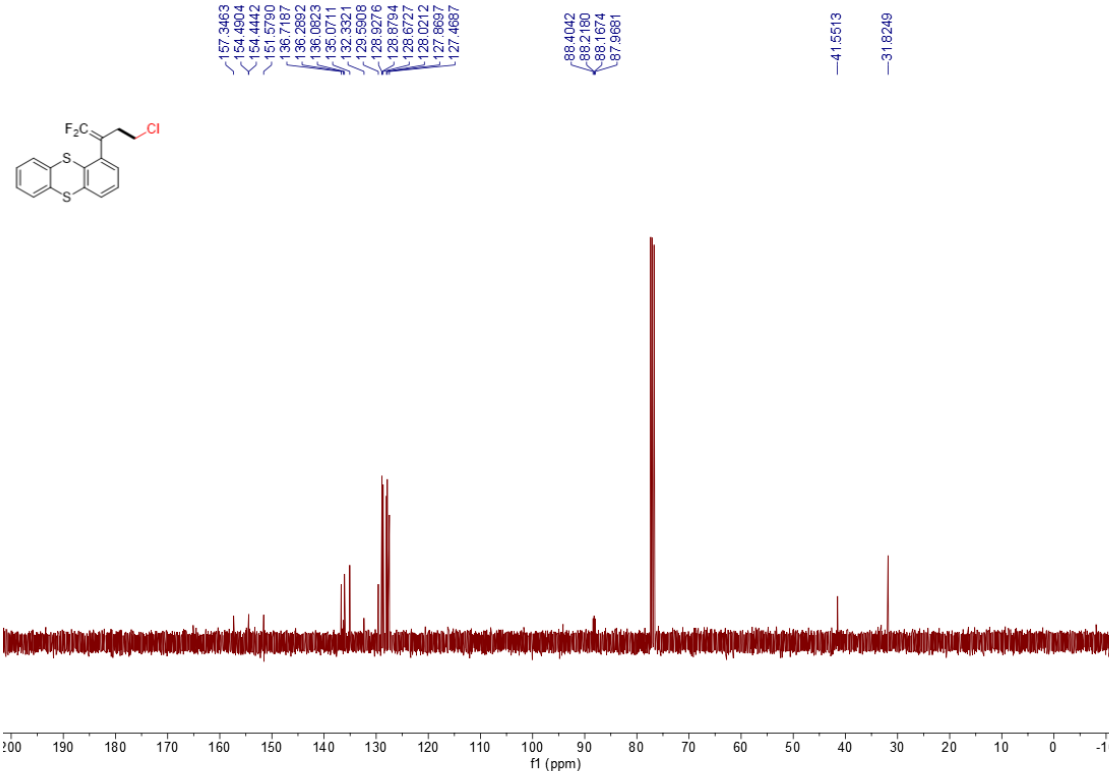

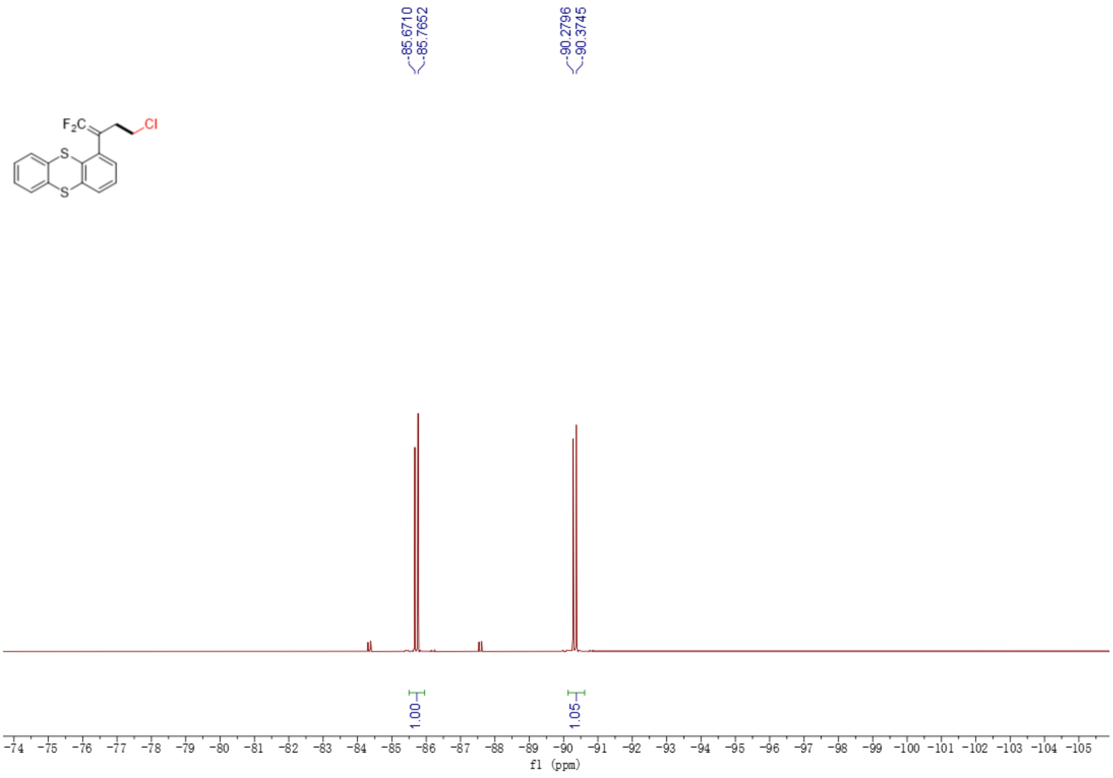

NMR spectrum of 22

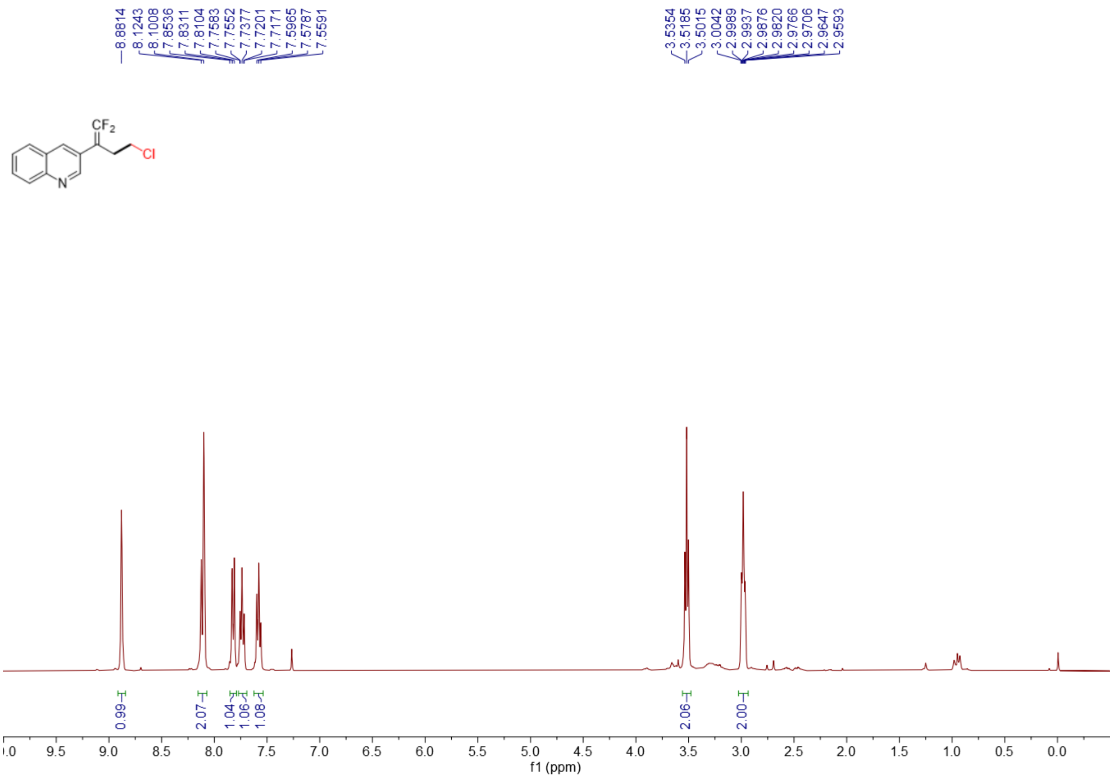

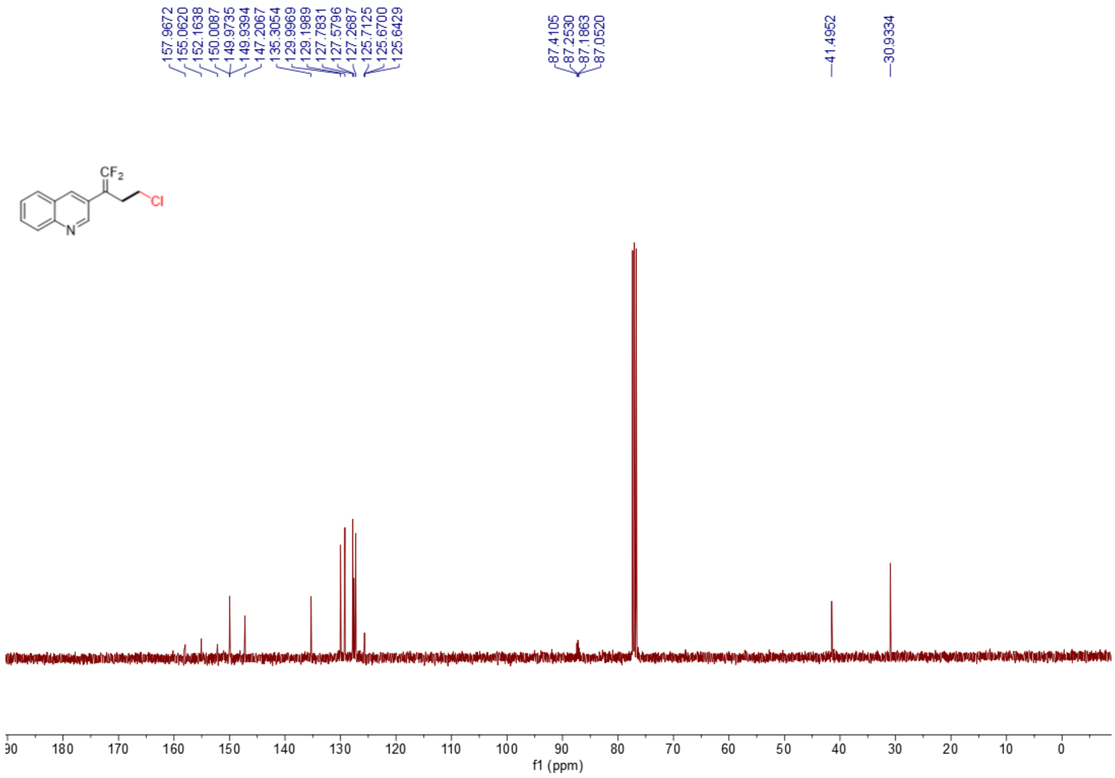
157.9672
155.0620
152.1638
150.0087
149.9735
149.9394
147.2067
135.3054
129.9969
129.1989
127.7831
127.5796
127.2687
125.7125
125.6700
125.6429
87.4105
87.2530
87.1863
87.0520
41.4952
30.9334
CF2
Cl
N
f1 (ppm)

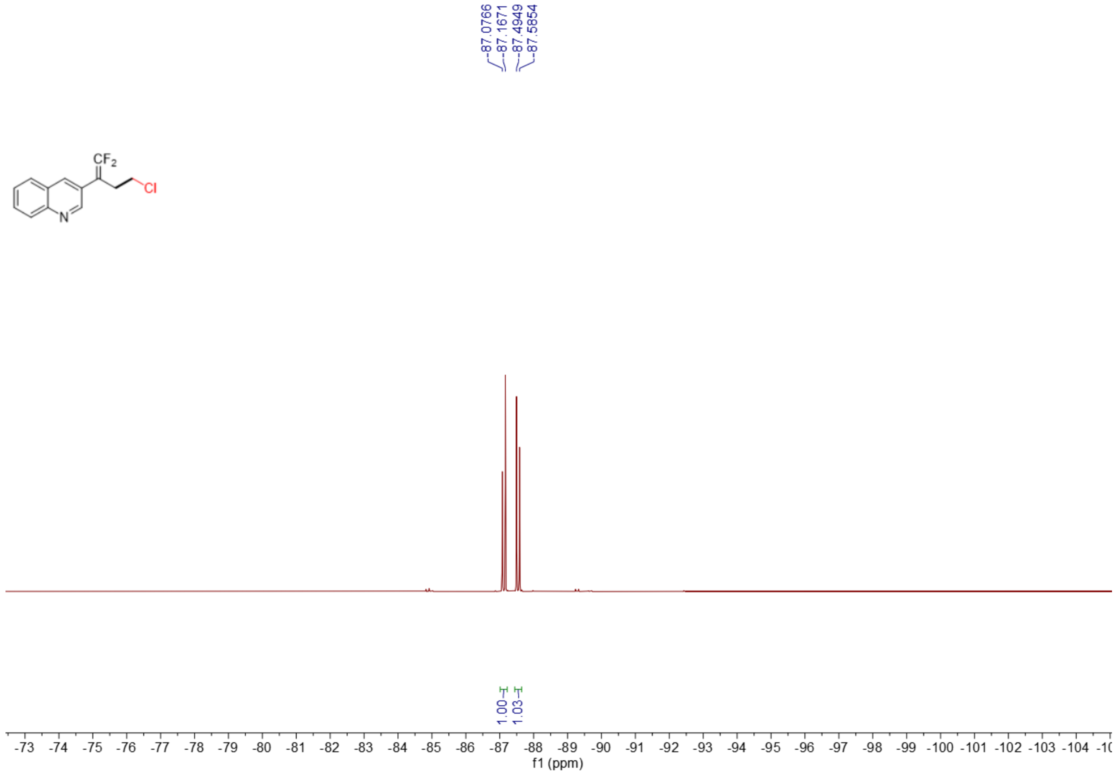
-87.0766
-87.1671
-87.4949
-87.5854
CF2
Cl
N
1.00
1.03
f1 (ppm)

NMR spectrum of 23

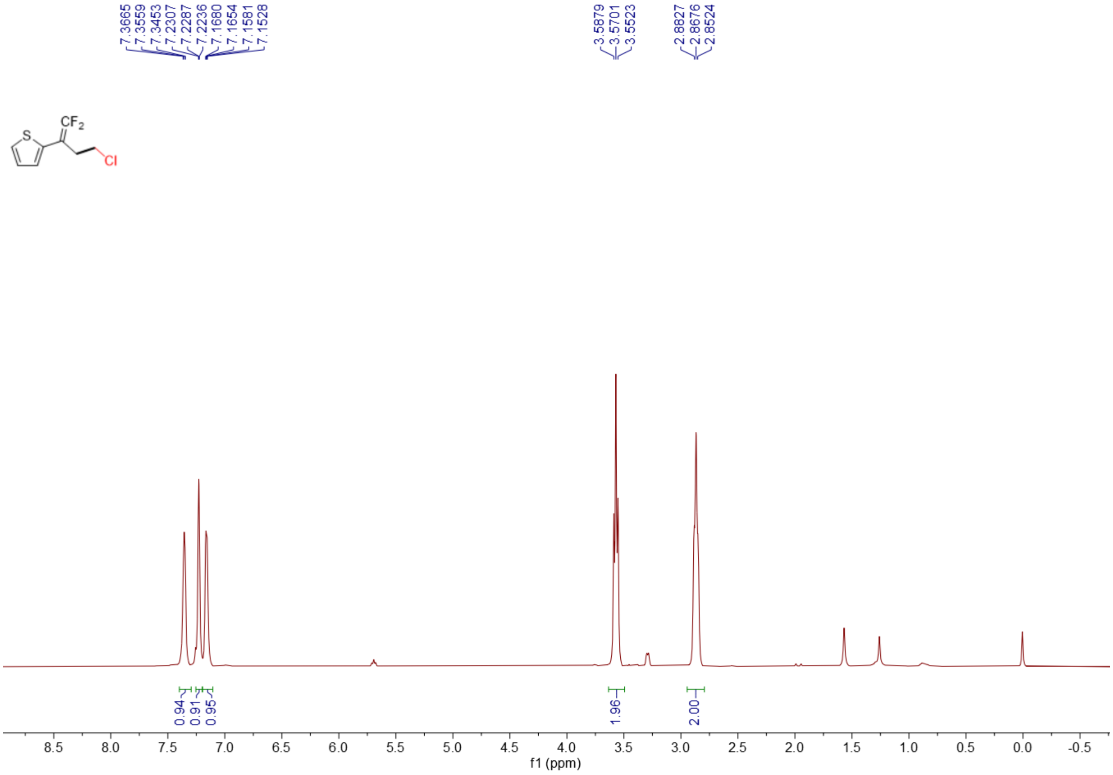

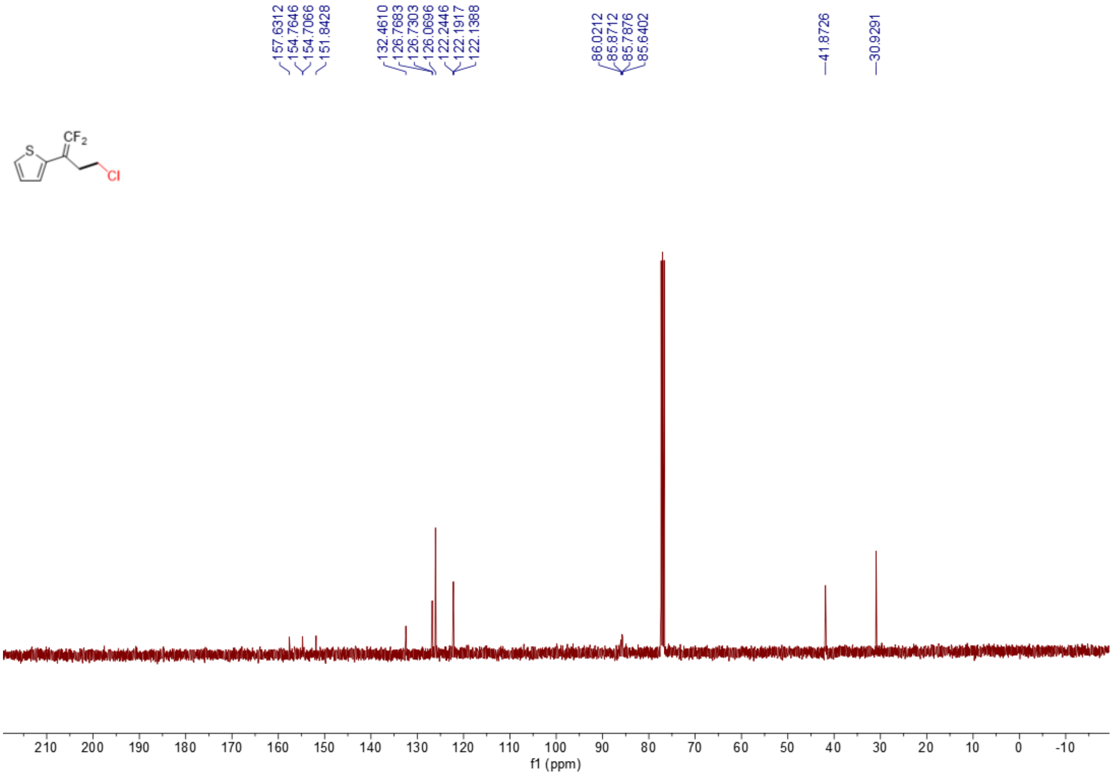

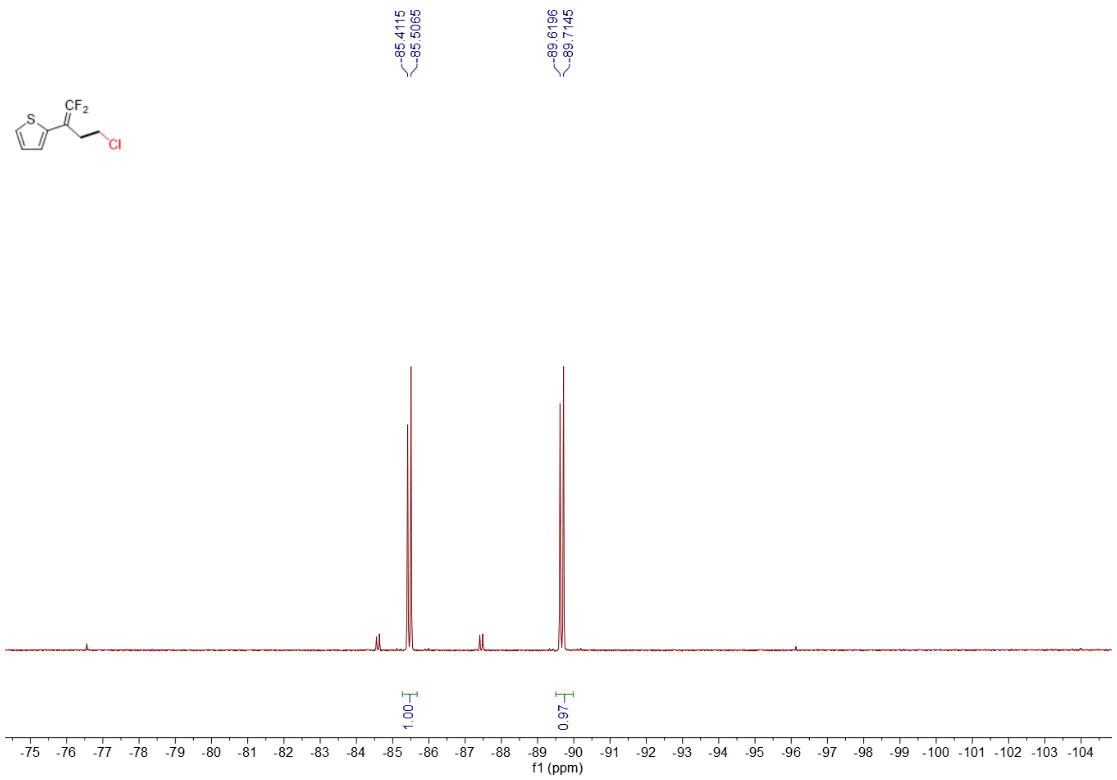

NMR spectrum of 24

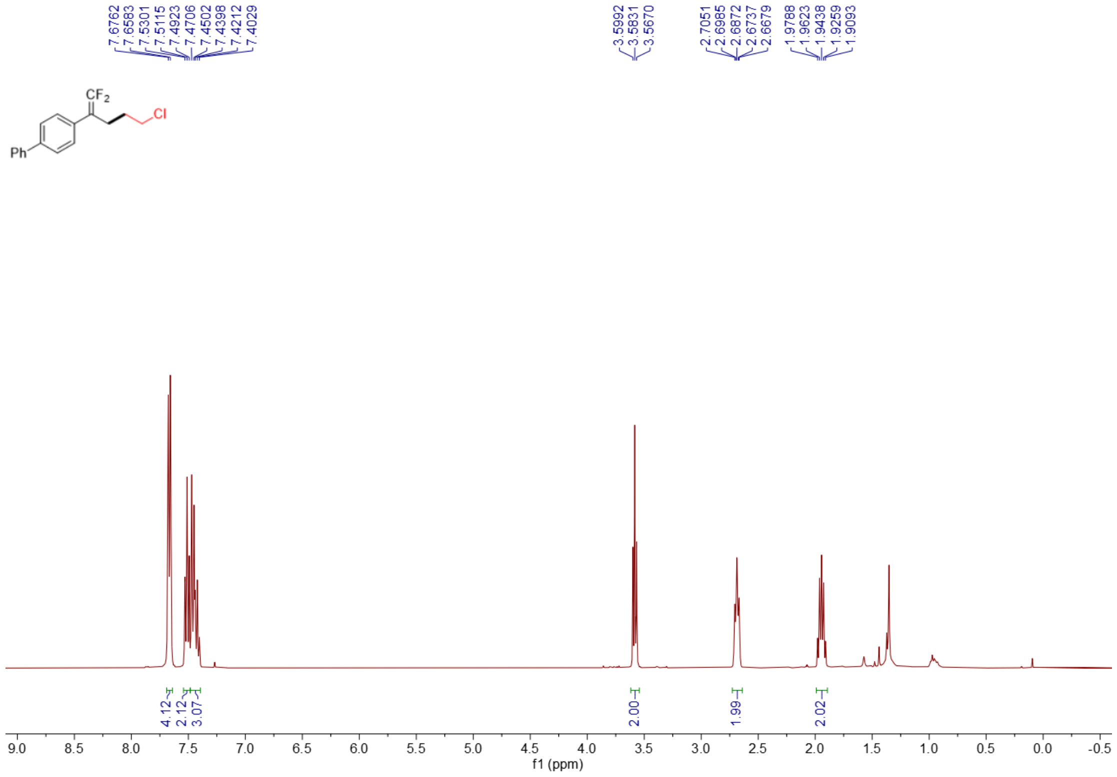

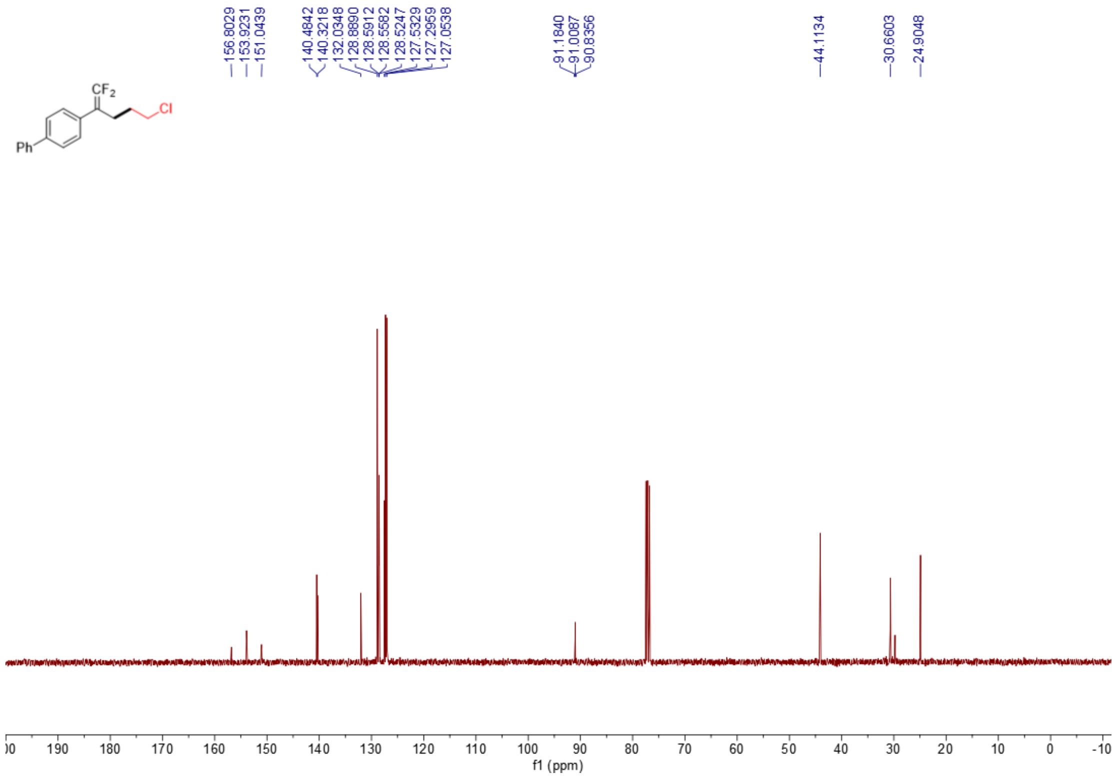

156.8029
153.9231
151.0439
140.4842
140.3218
132.0348
128.8890
128.5912
128.5562
128.5247
127.5329
127.2959
127.0538
91.1840
91.0087
90.8356
44.1134
30.6603
24.9048
CF2
Cl
Ph
f1 (ppm)

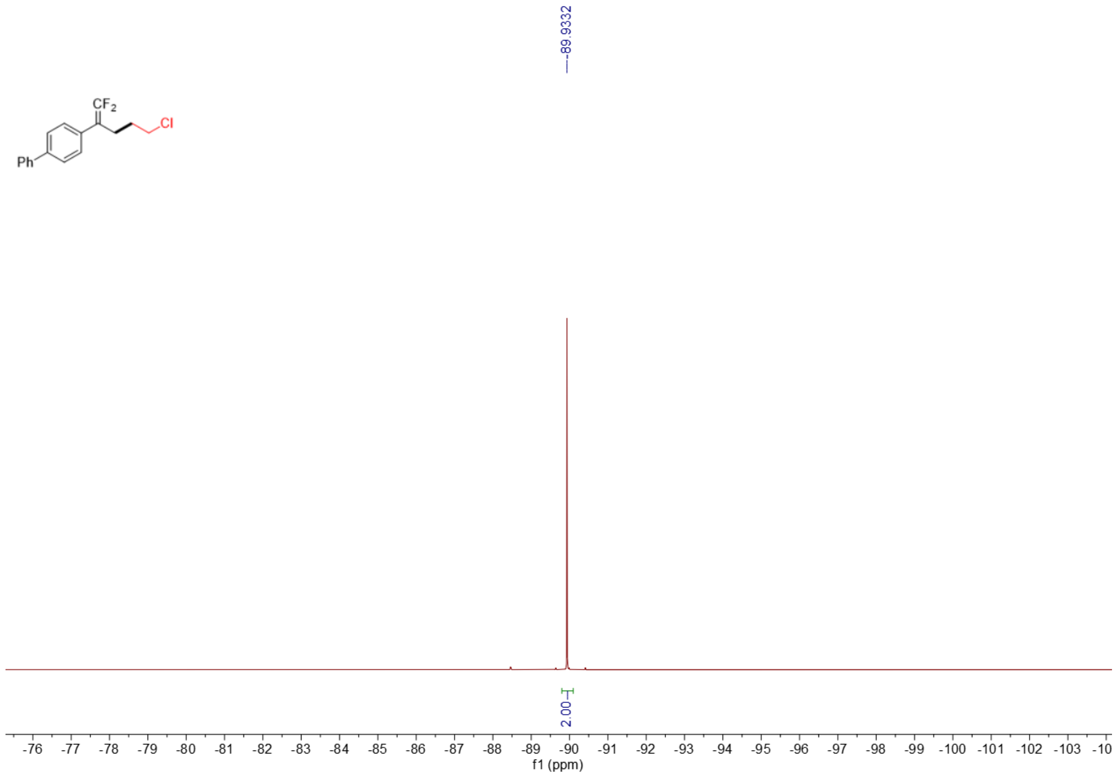

-89.9332
CF2
Cl
Ph
2.00
f1 (ppm)

NMR spectrum of 25

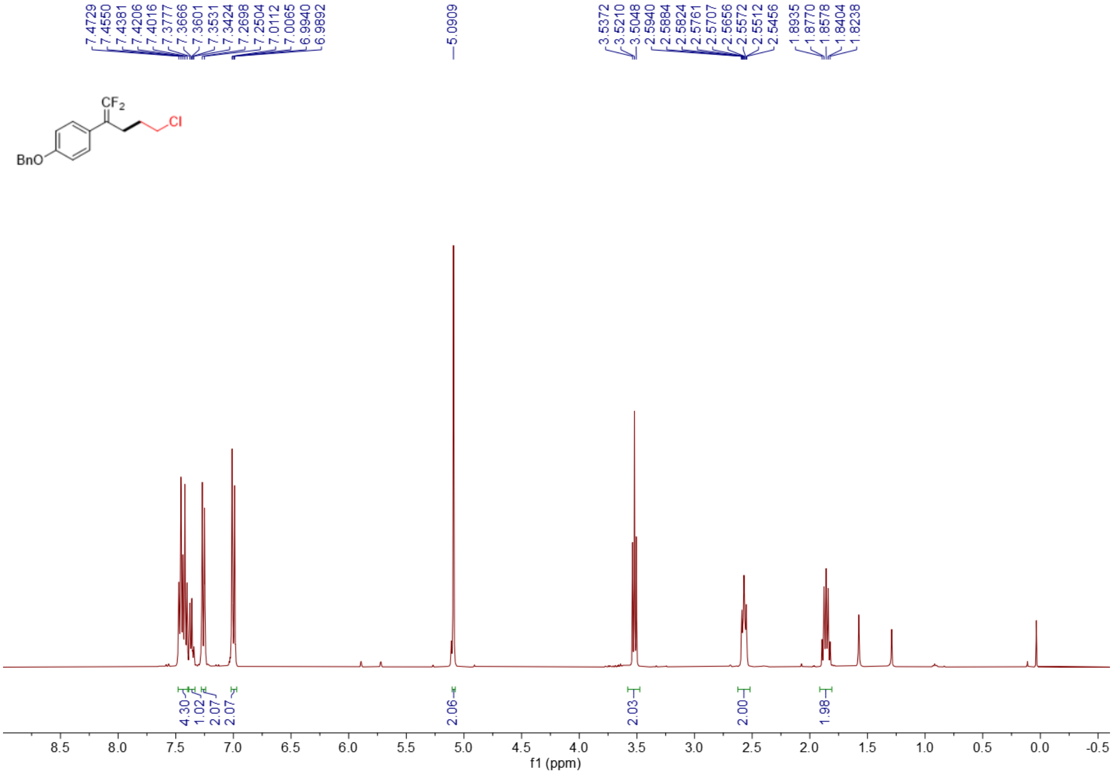

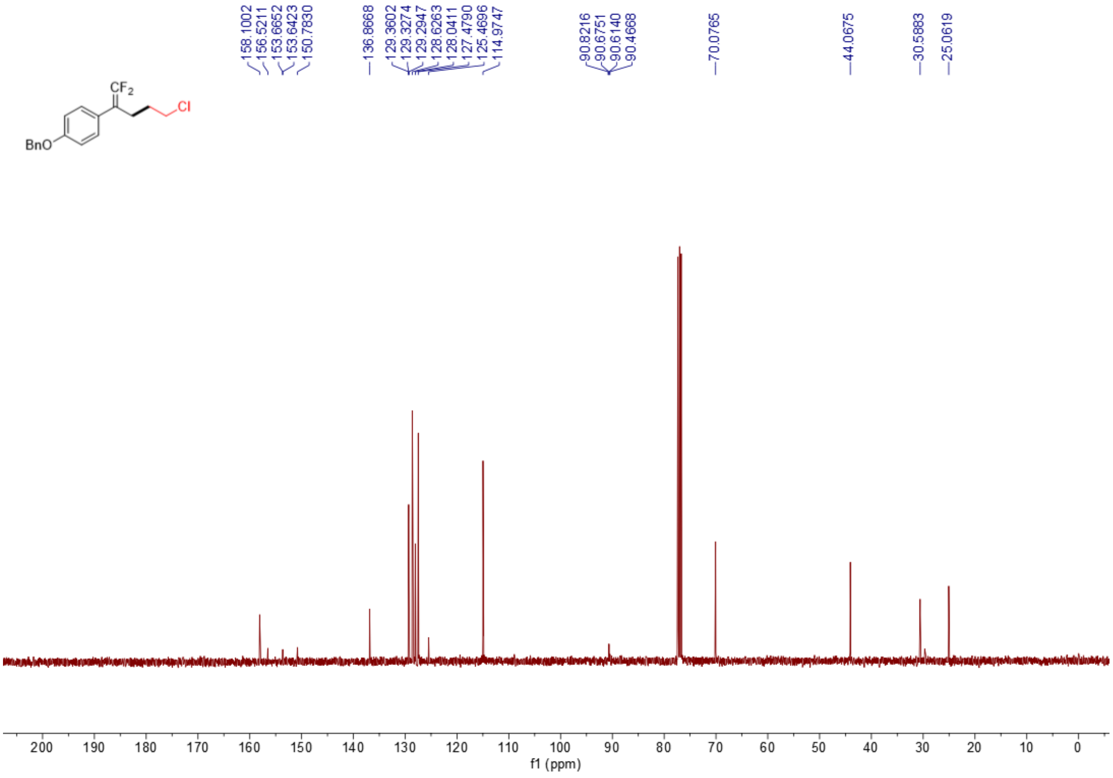

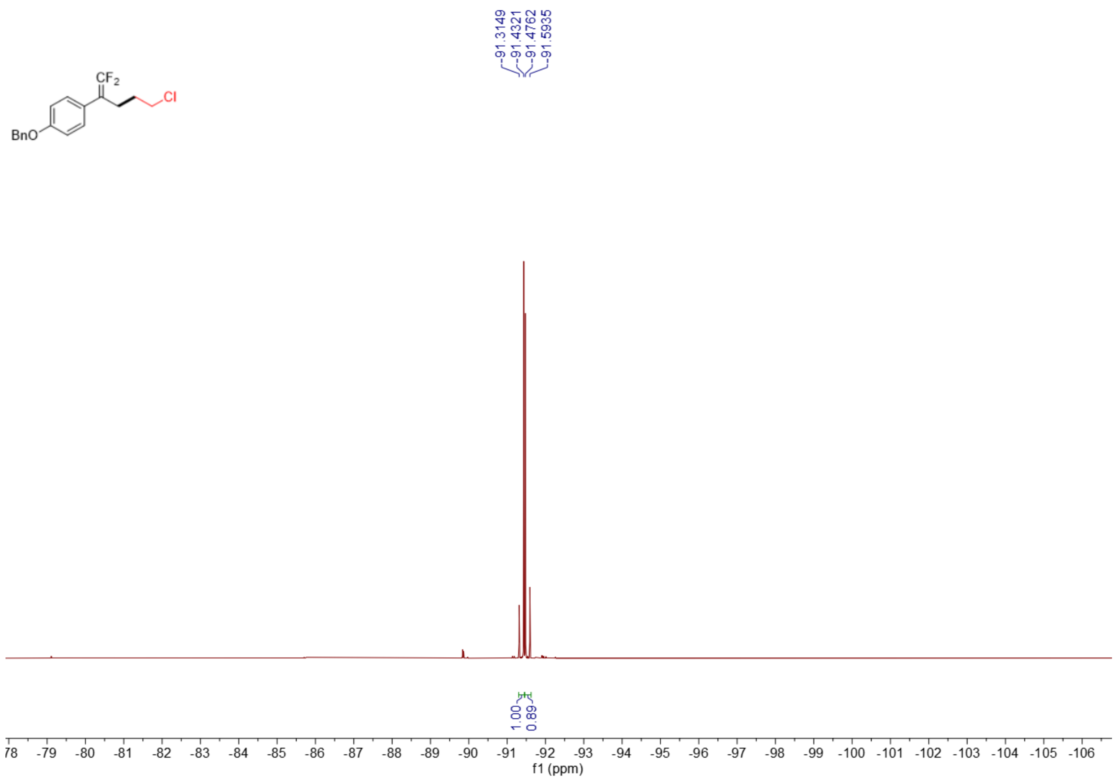

NMR spectrum of 26

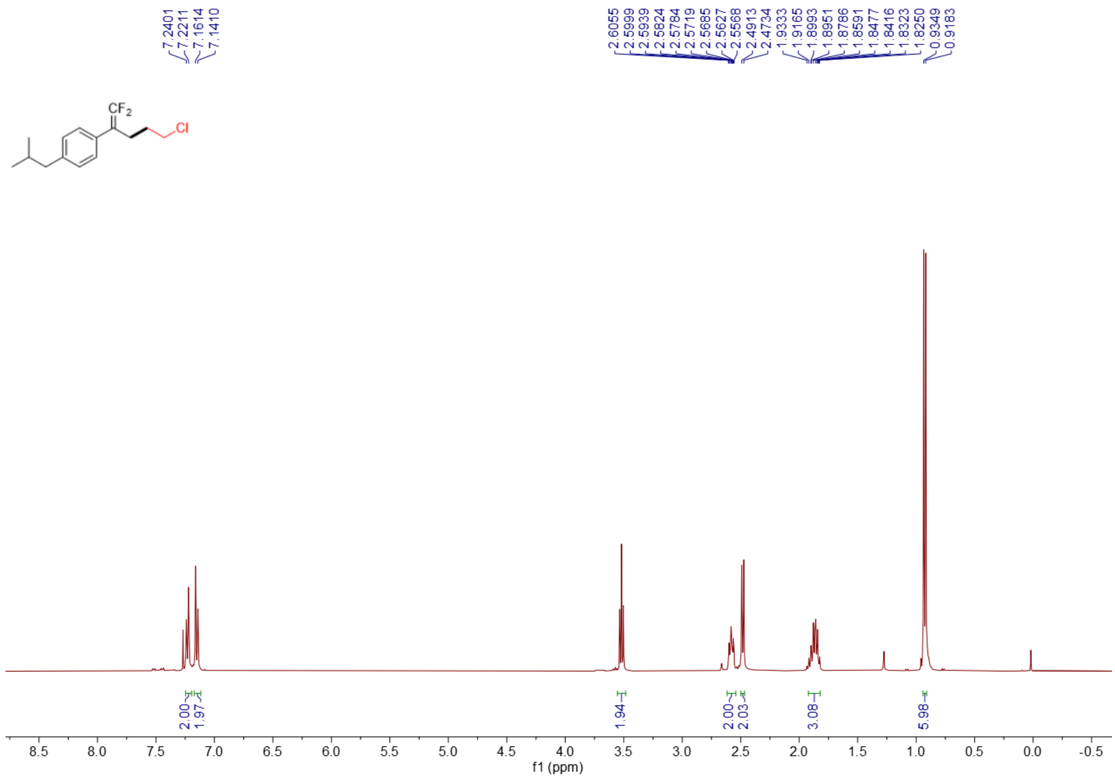

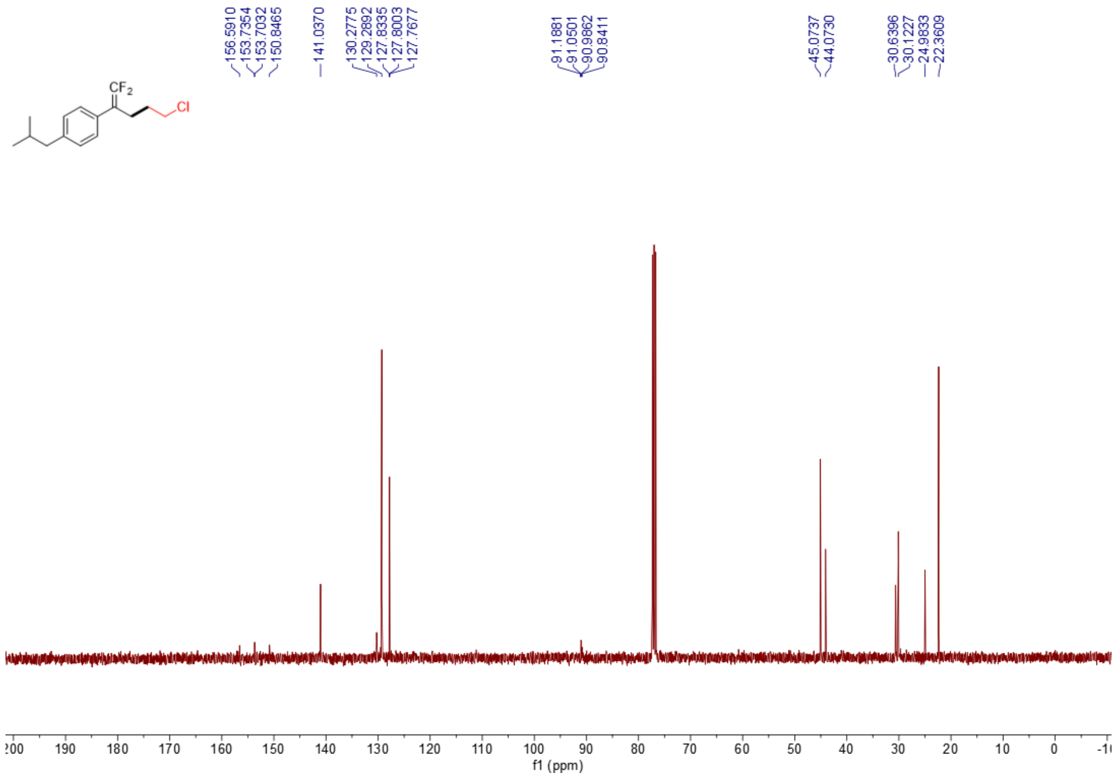
156.5910
153.7354
153.7032
150.8465
141.0370
130.2775
129.2892
127.8335
127.8003
127.7677
91.1881
91.0501
90.9862
90.8411
45.0737
44.0730
30.6396
30.1227
24.9833
22.3609
CF2
Cl
f1 (ppm)

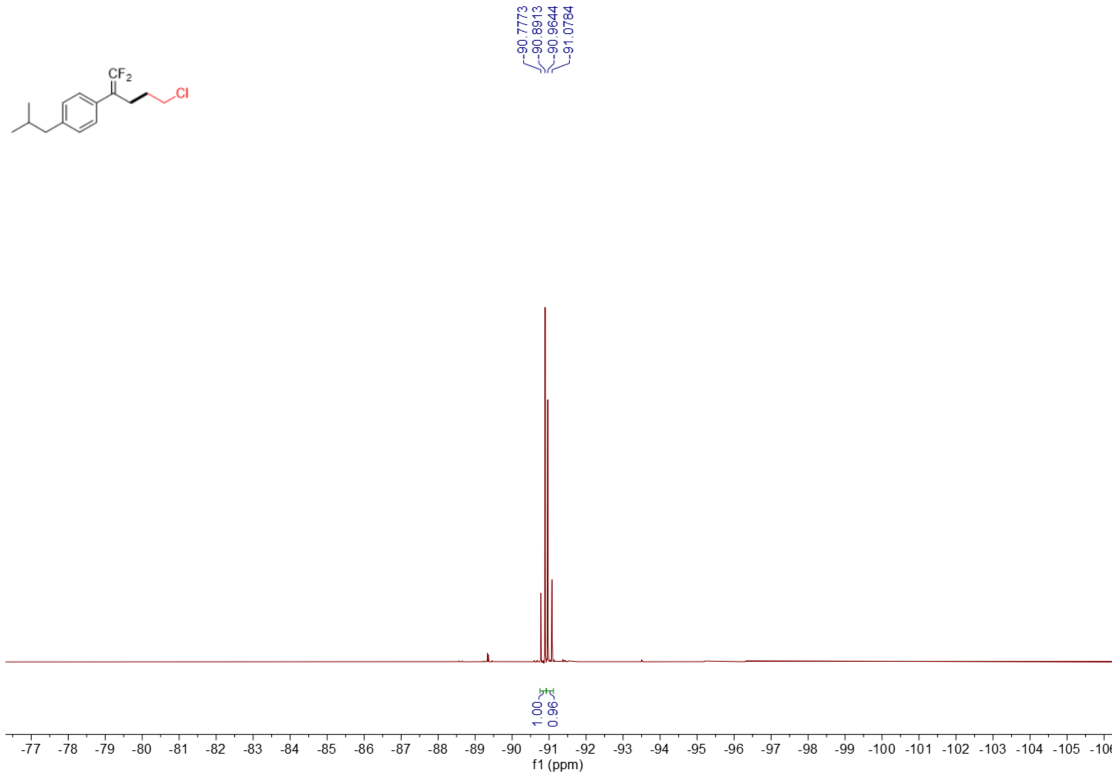
-90.7773
-90.8913
-90.9644
-91.0784
CF2
Cl
1.00
0.96
f1 (ppm)

NMR spectrum of 27

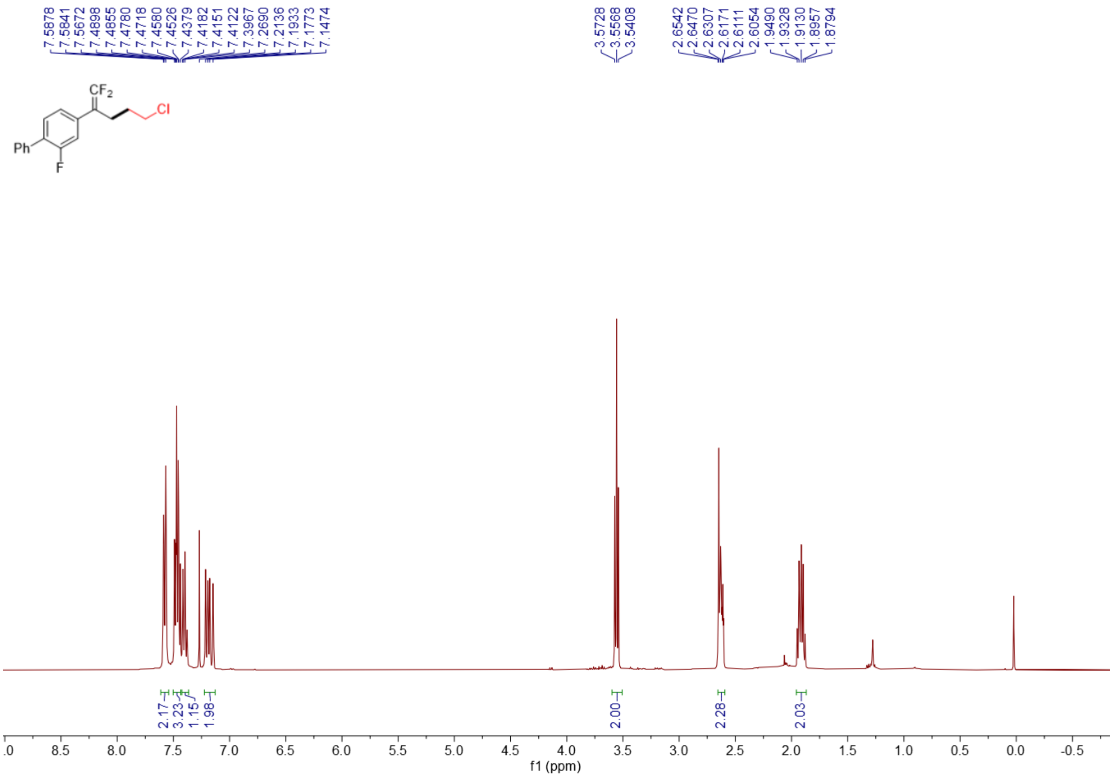

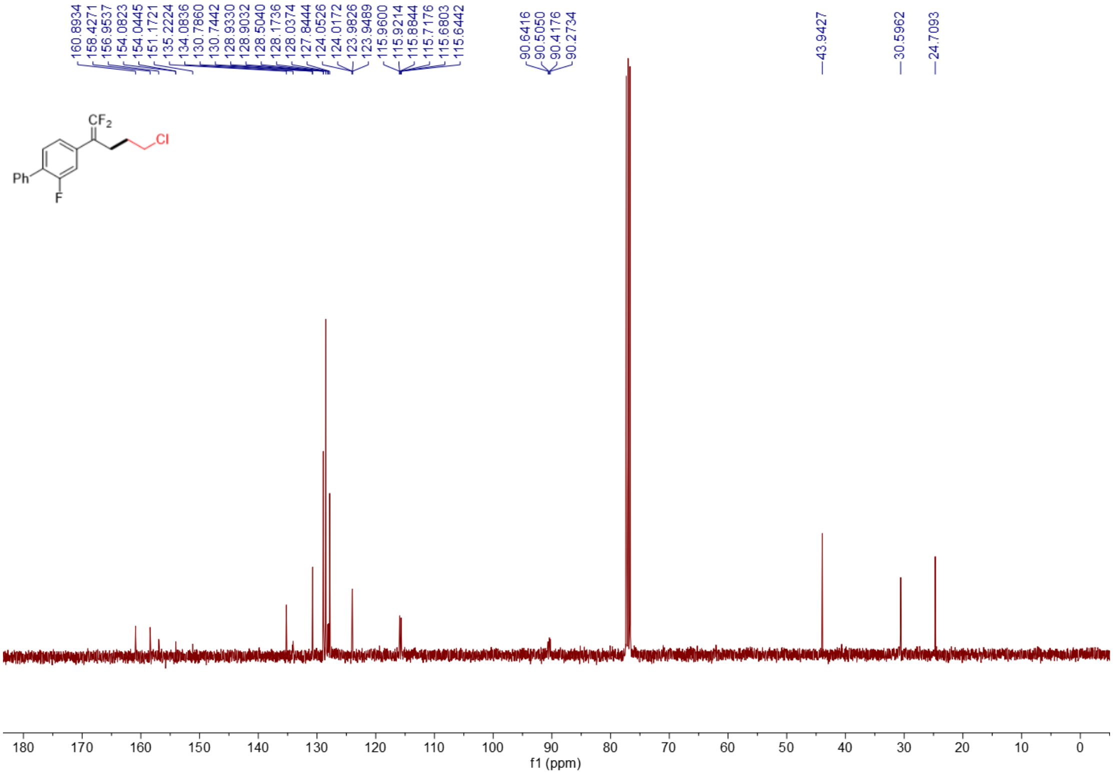

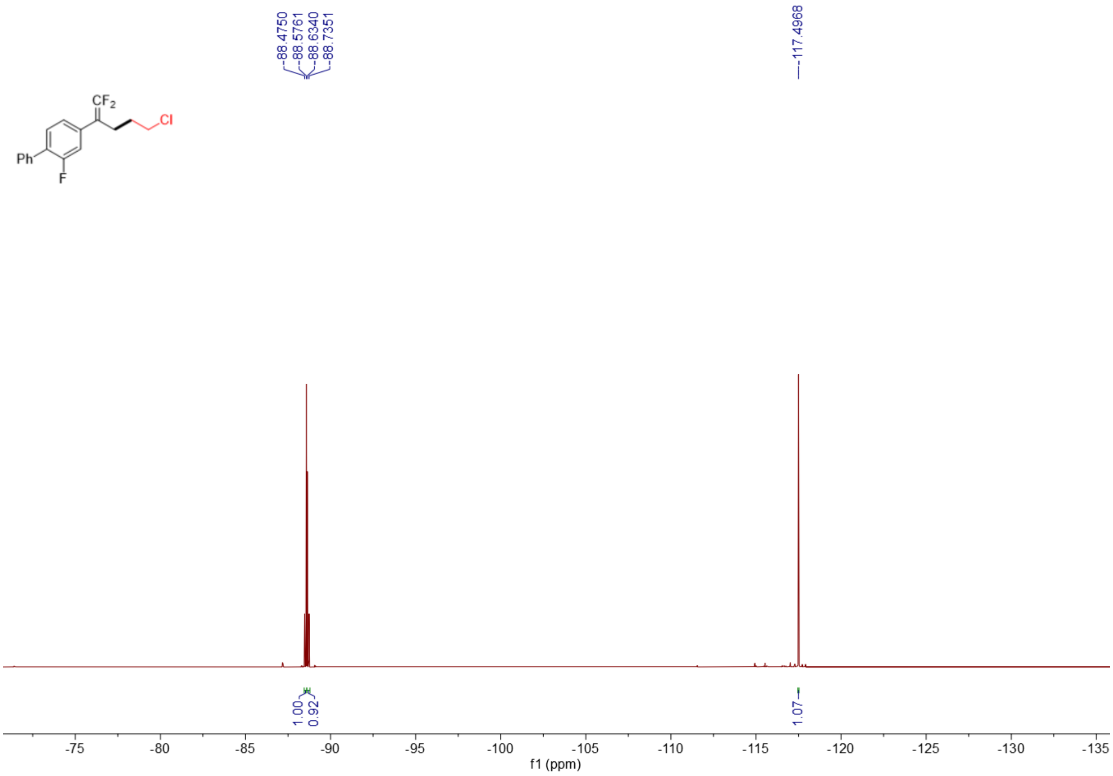

NMR spectrum of 28

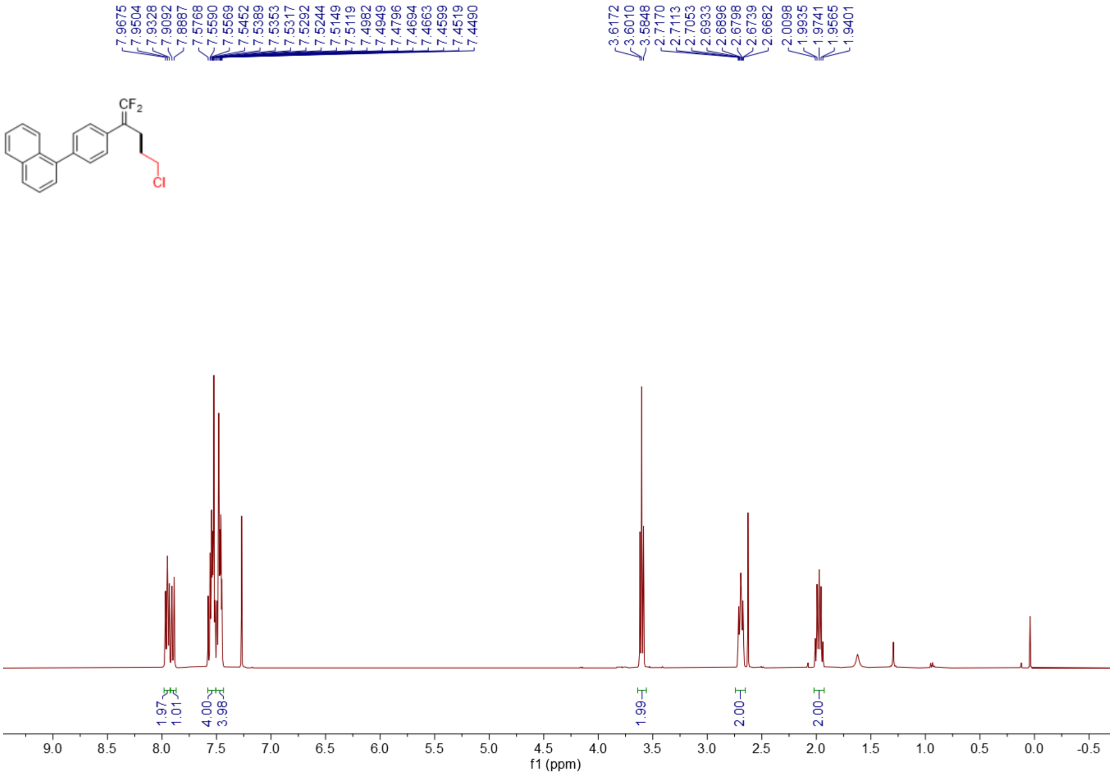

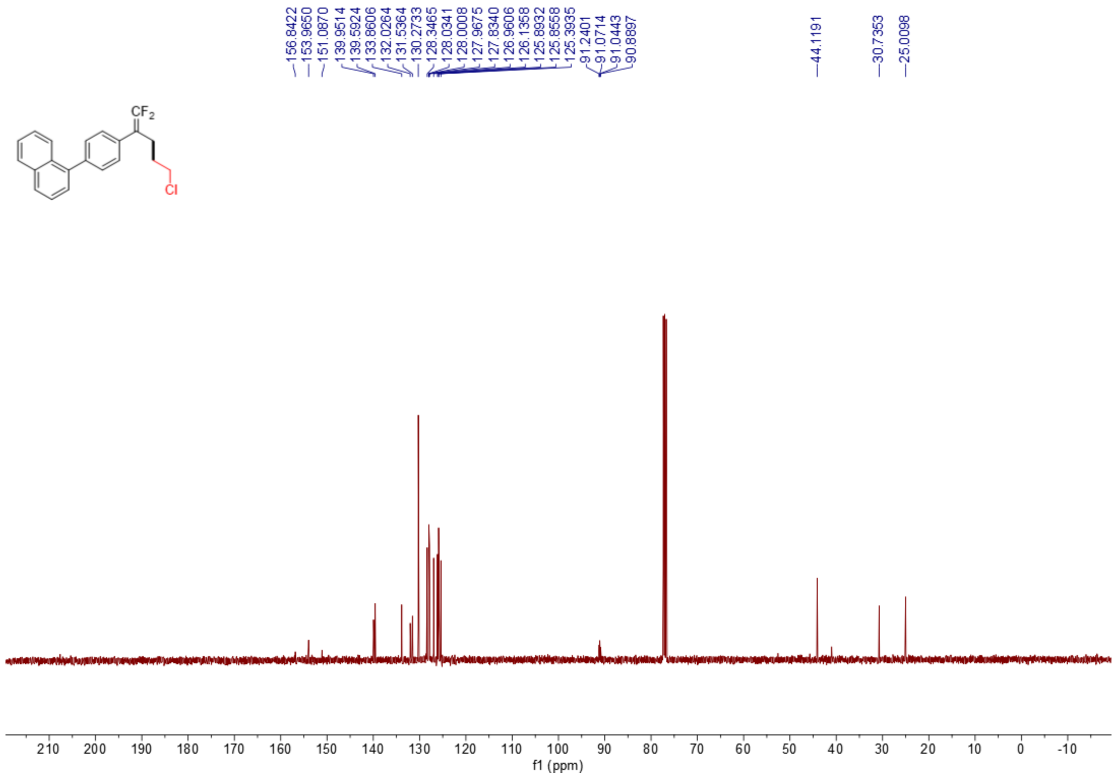
CF2
Cl
156.8422
153.9650
151.0870
139.9514
139.5924
133.8606
132.0264
131.5364
130.2733
128.3465
128.0341
128.0008
127.9675
127.8340
126.9606
126.1358
125.8932
125.8558
125.3935
91.2401
91.0714
91.0443
90.8897
44.1191
30.7353
25.0098
210 200 190 180 170 160 150 140 130 120 110 100 90 80 70 60 50 40 30 20 10 0 -10
f1 (ppm)

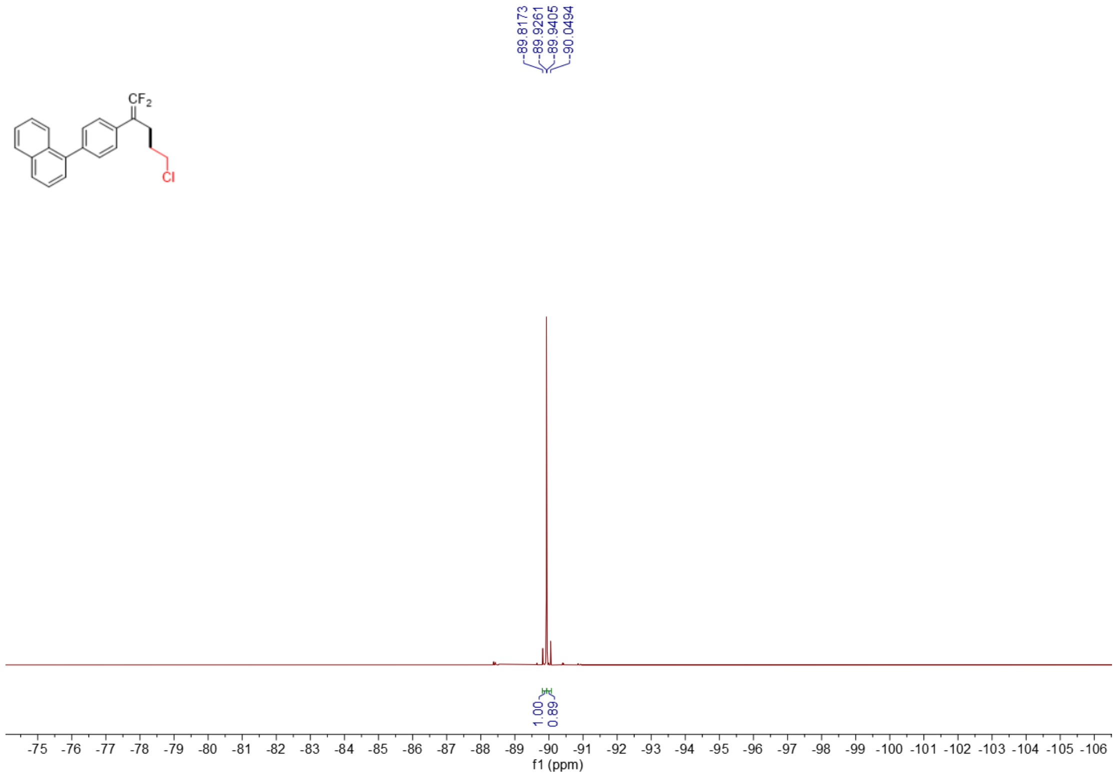
CF2
Cl
-89.8173
-89.9261
-89.9405
-90.0494
1.00
0.89
-75 -76 -77 -78 -79 -80 -81 -82 -83 -84 -85 -86 -87 -88 -89 -90 -91 -92 -93 -94 -95 -96 -97 -98 -99 -100 -101 -102 -103 -104 -105 -106
f1 (ppm)

NMR spectrum of 29

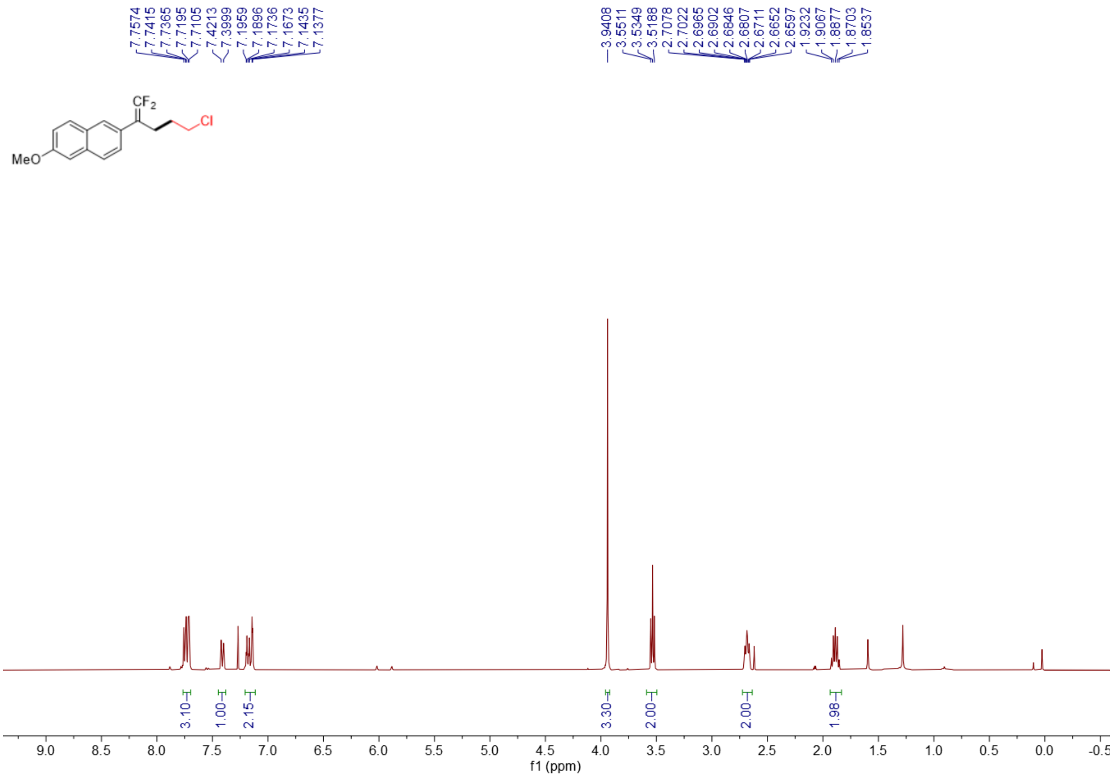

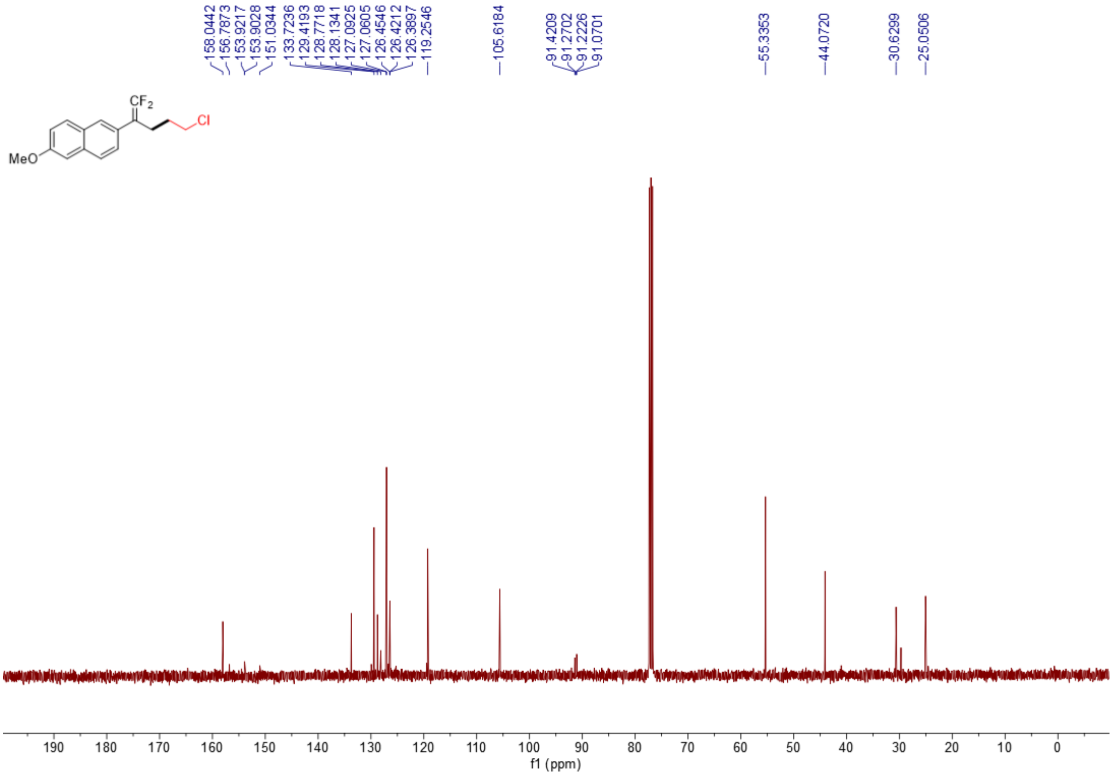

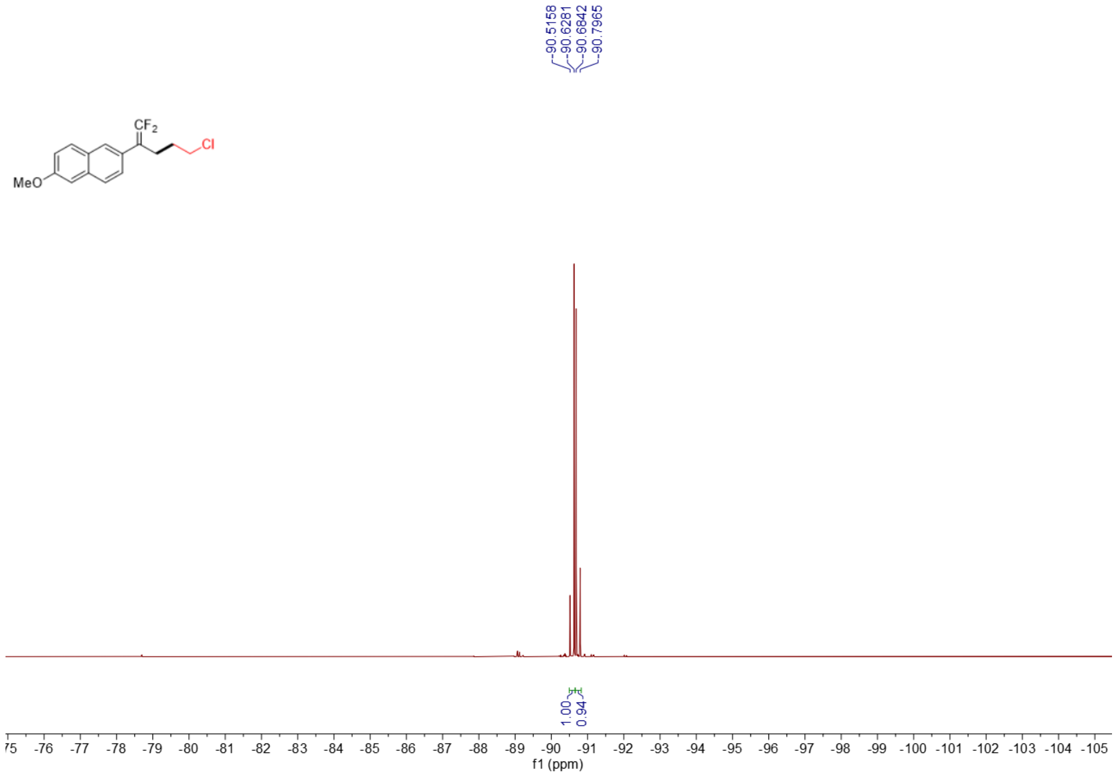

NMR spectrum of 30

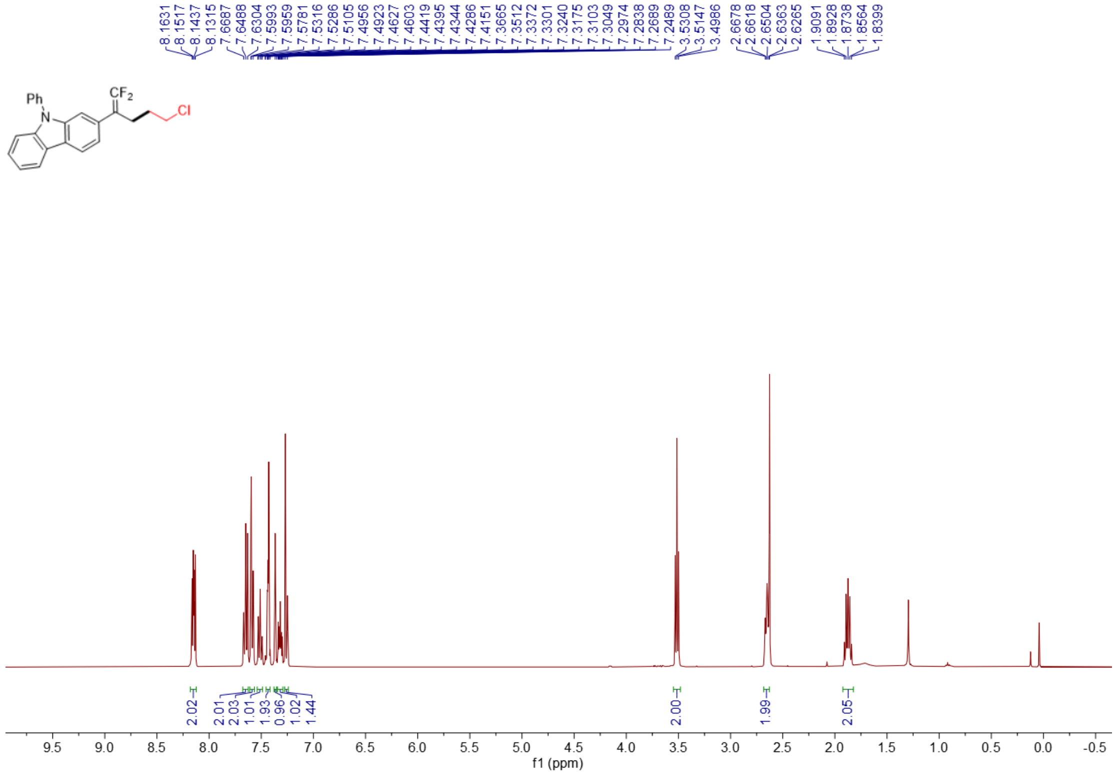

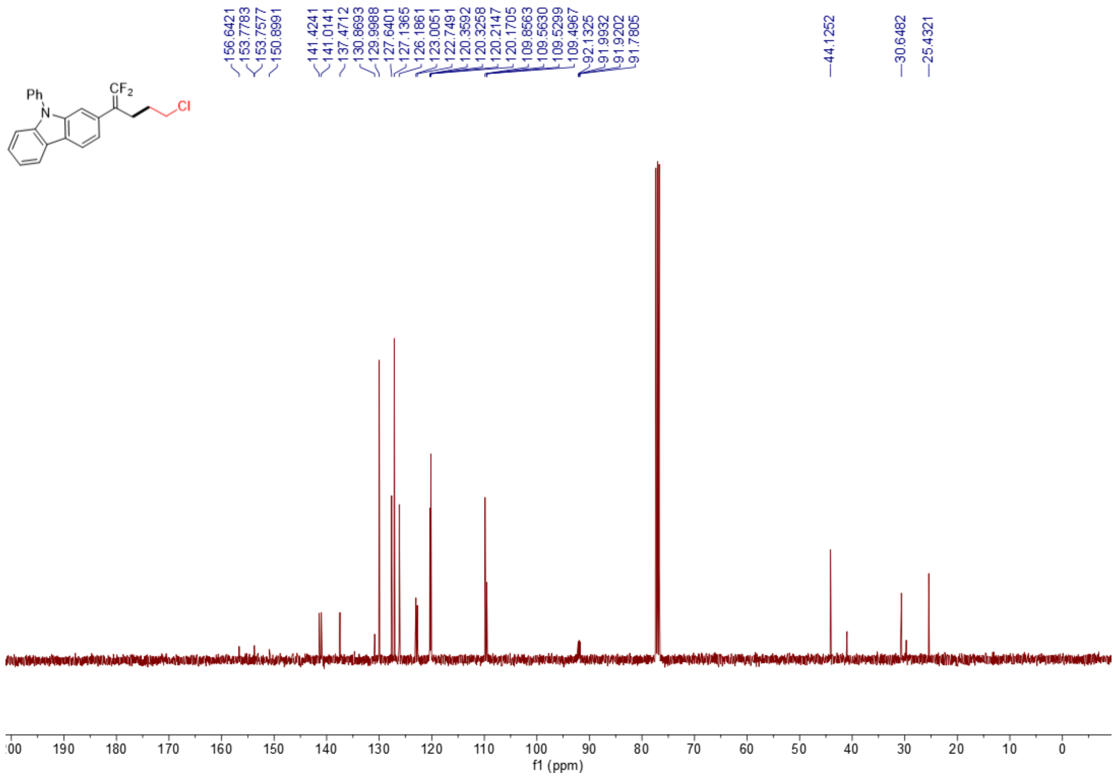
156.6421
153.7783
153.7577
150.8991
141.4241
141.0141
137.4712
130.8693
129.9988
127.6401
127.1365
126.1861
123.0051
122.7491
120.3592
120.3258
120.2147
120.1705
109.8563
109.5630
109.5299
109.4967
92.1325
91.9932
91.9202
91.7805
44.1252
30.6482
25.4321
Ph
N
CF2
Cl
f1 (ppm)

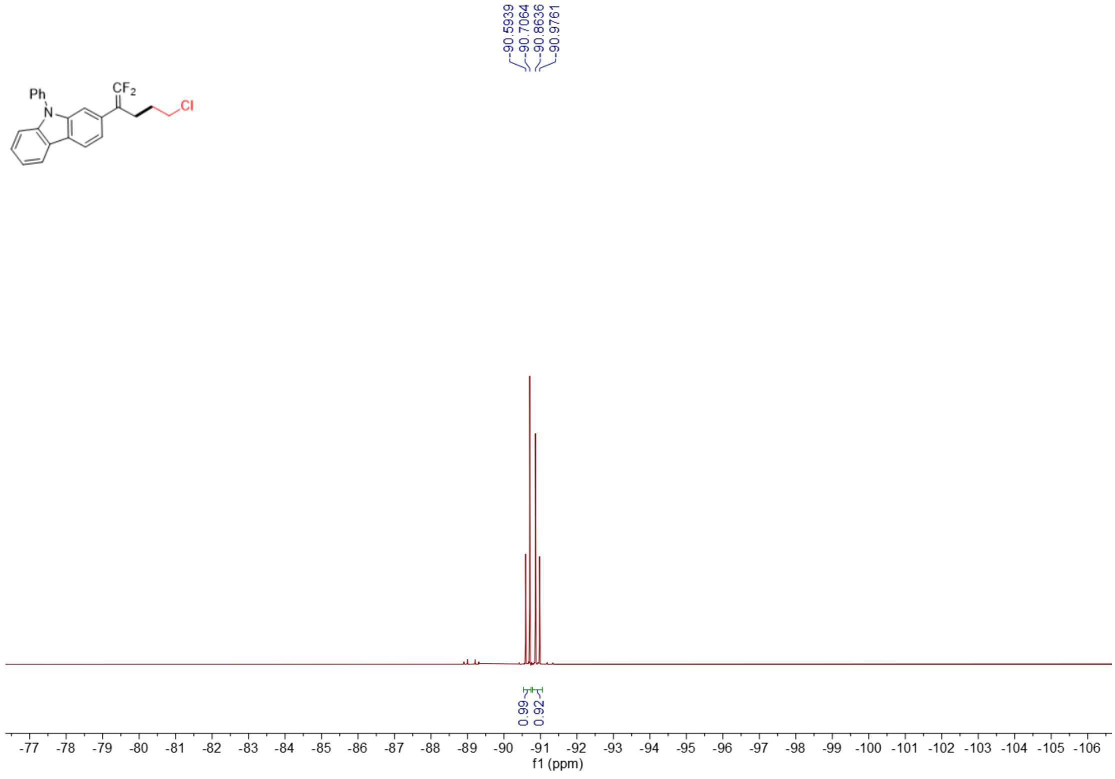
-90.5939
-90.7064
-90.8636
-90.9761
Ph
N
CF2
Cl
0.99
0.92
f1 (ppm)

NMR spectrum of 31

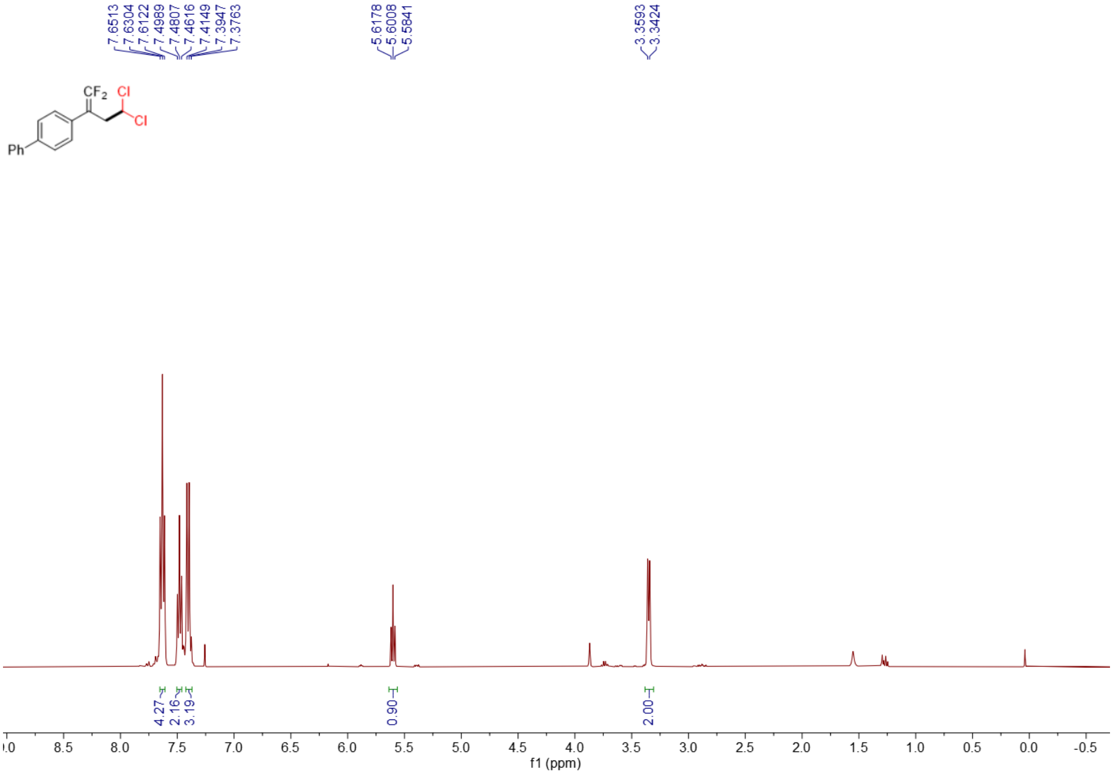

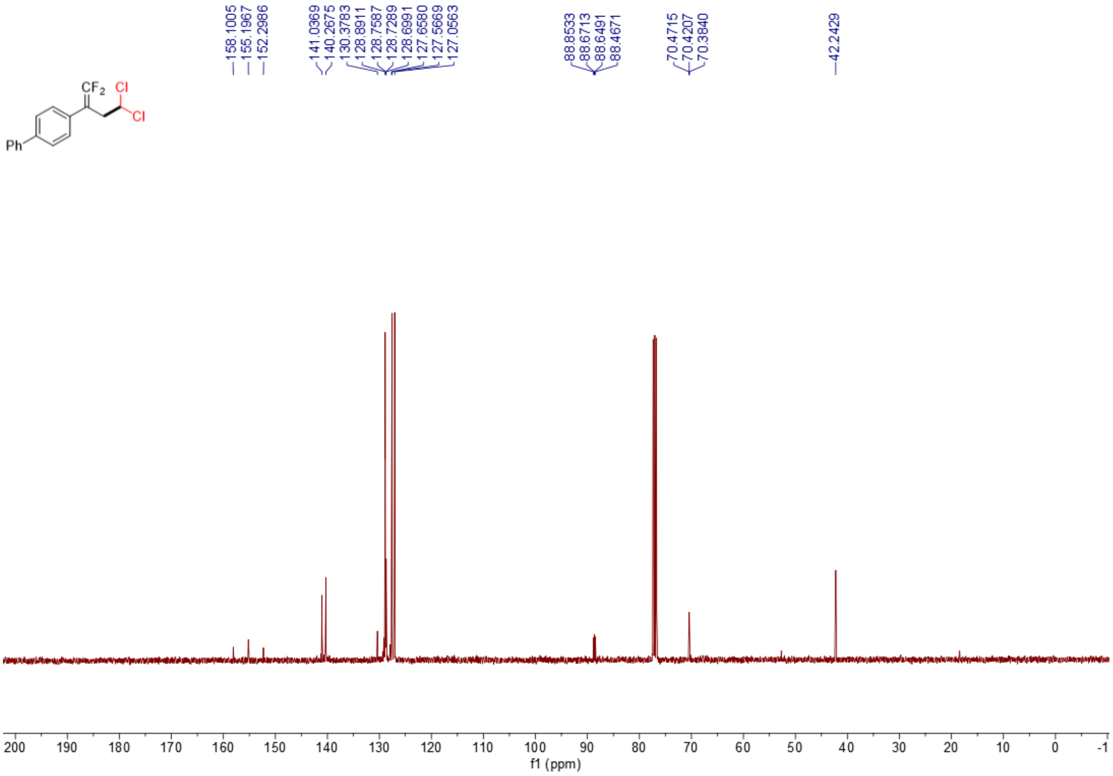

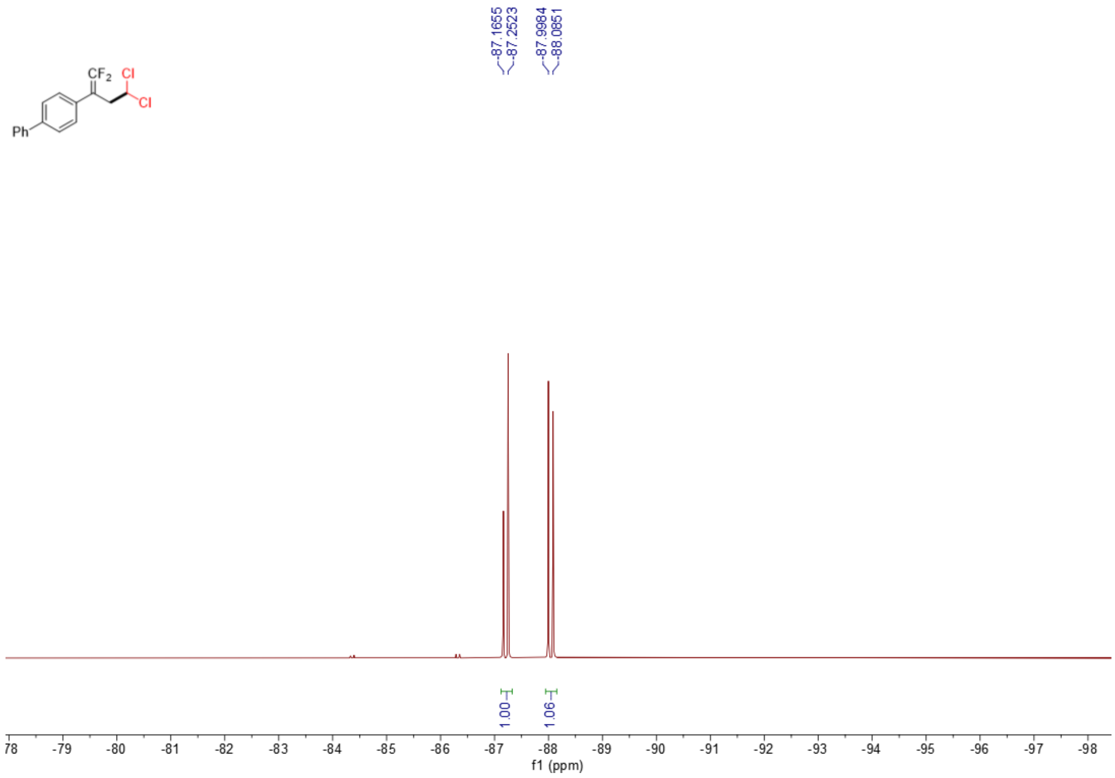

NMR spectrum of 32

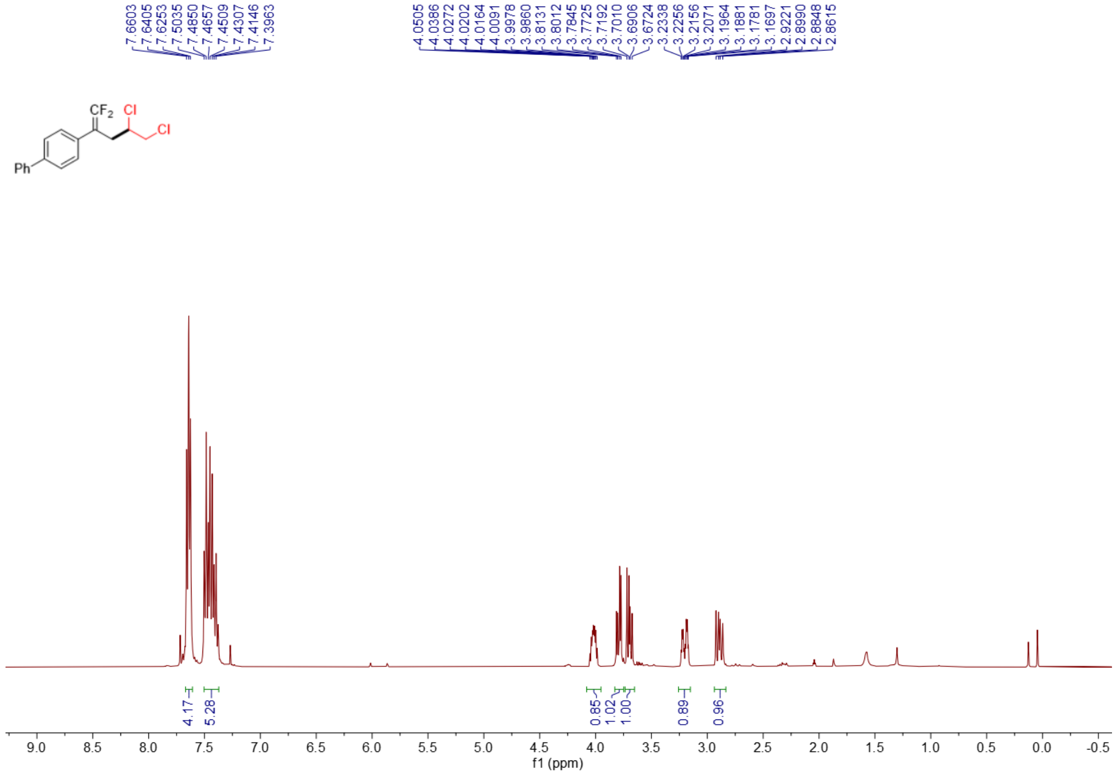

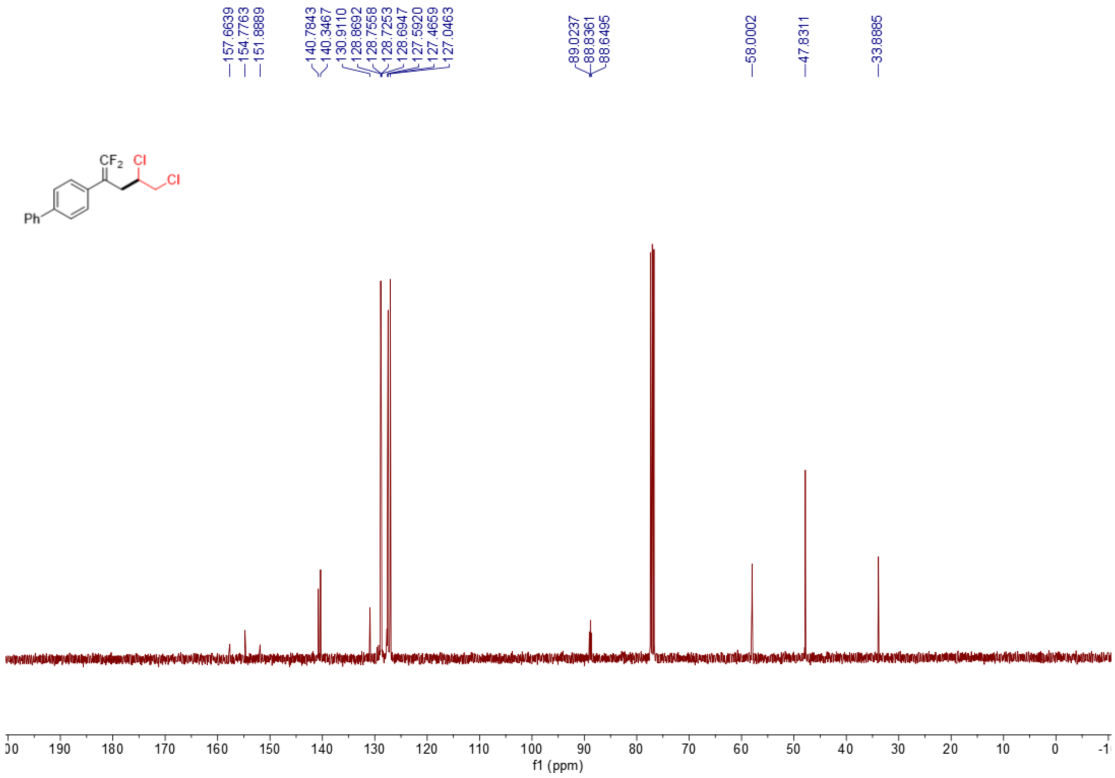

157.6639
154.7763
151.8889
140.7843
140.3467
130.9110
128.8692
128.7558
128.7253
128.6947
127.5920
127.4659
127.0463
89.0237
88.8361
88.6495
58.0002
47.8311
33.8885
CF2
Cl
Cl
Ph
f1 (ppm)

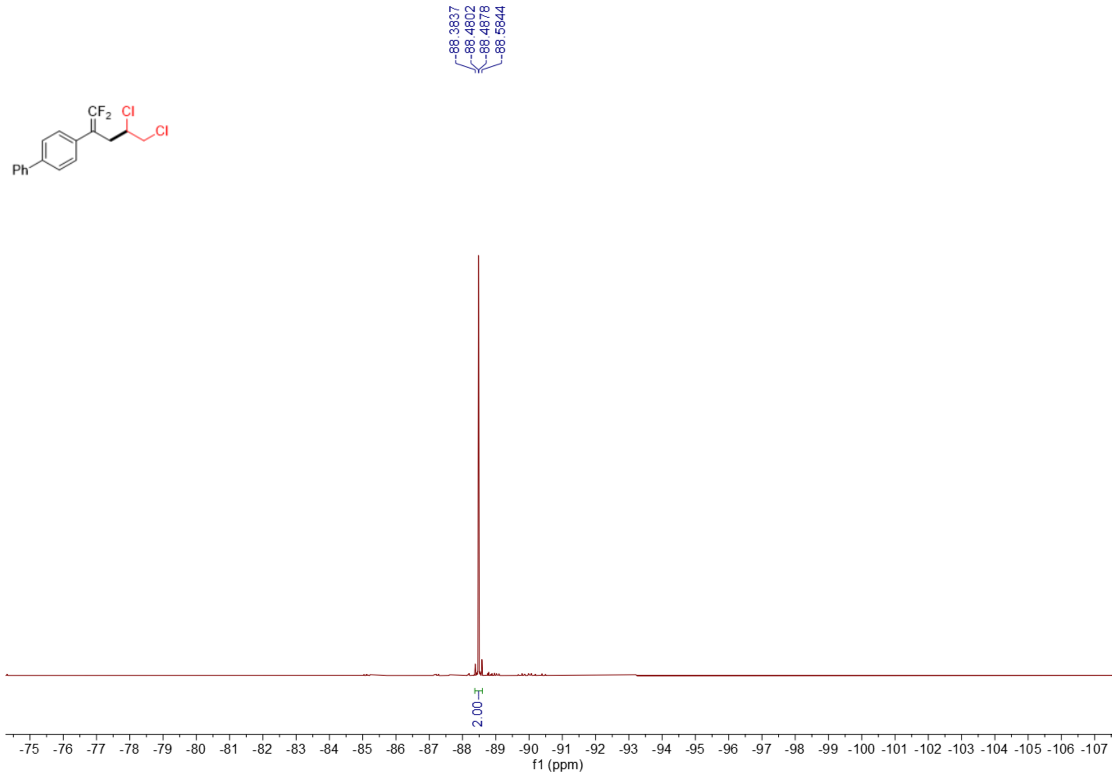

-88.3837
-88.4802
-88.4878
-88.5844
CF2
Cl
Cl
Ph
2.00
f1 (ppm)

NMR spectrum of 33

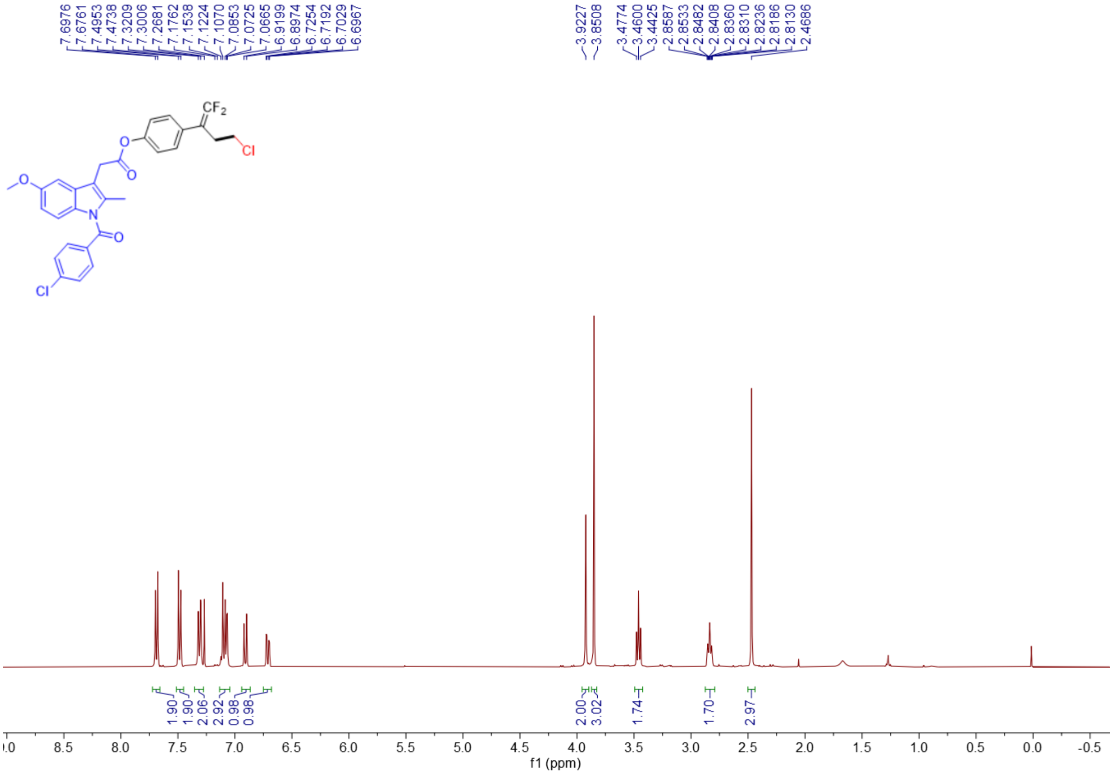

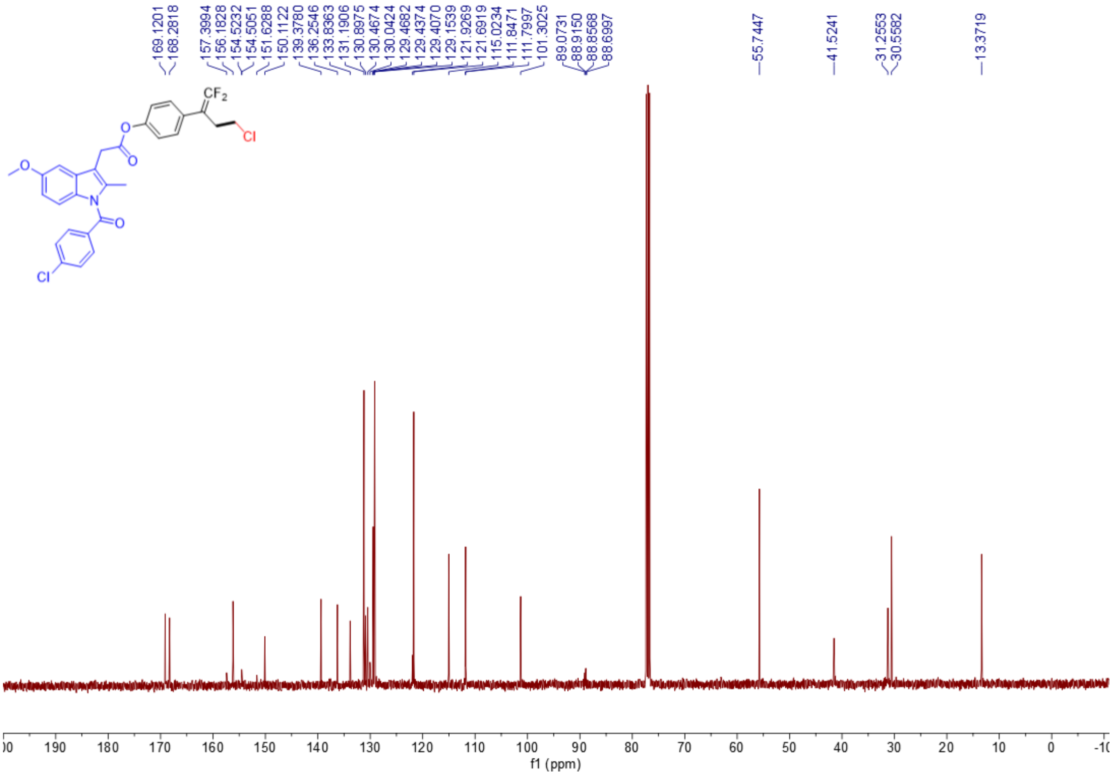

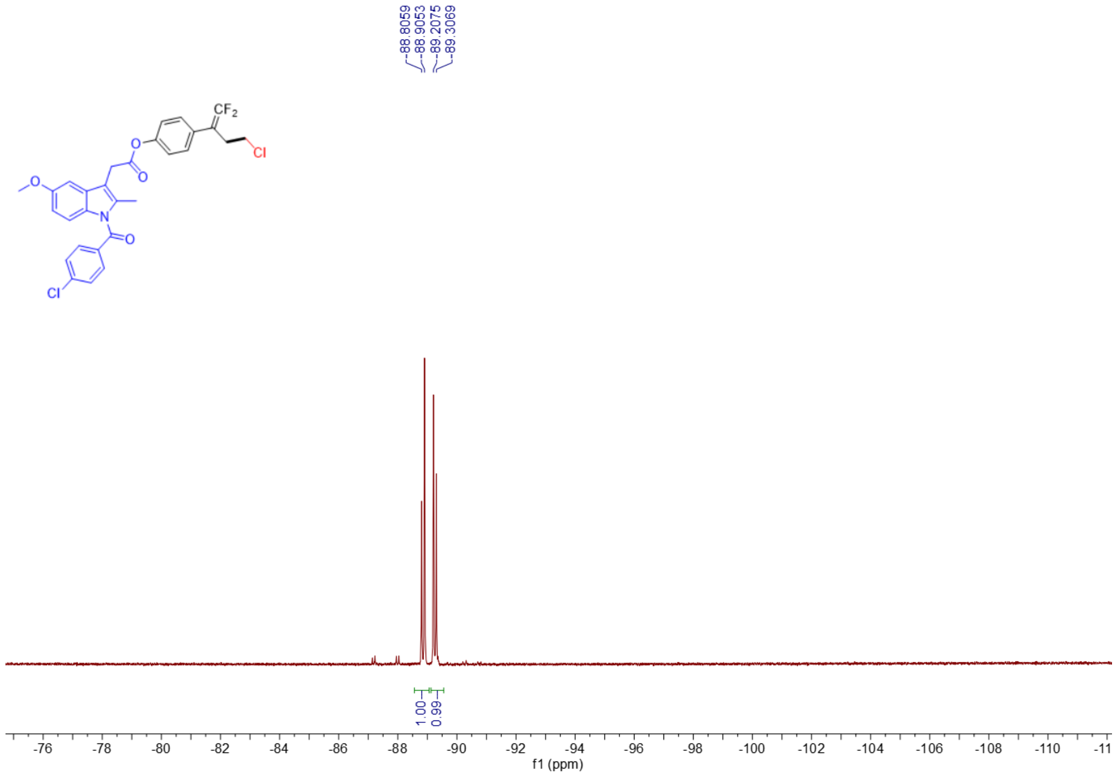

NMR spectrum of 34

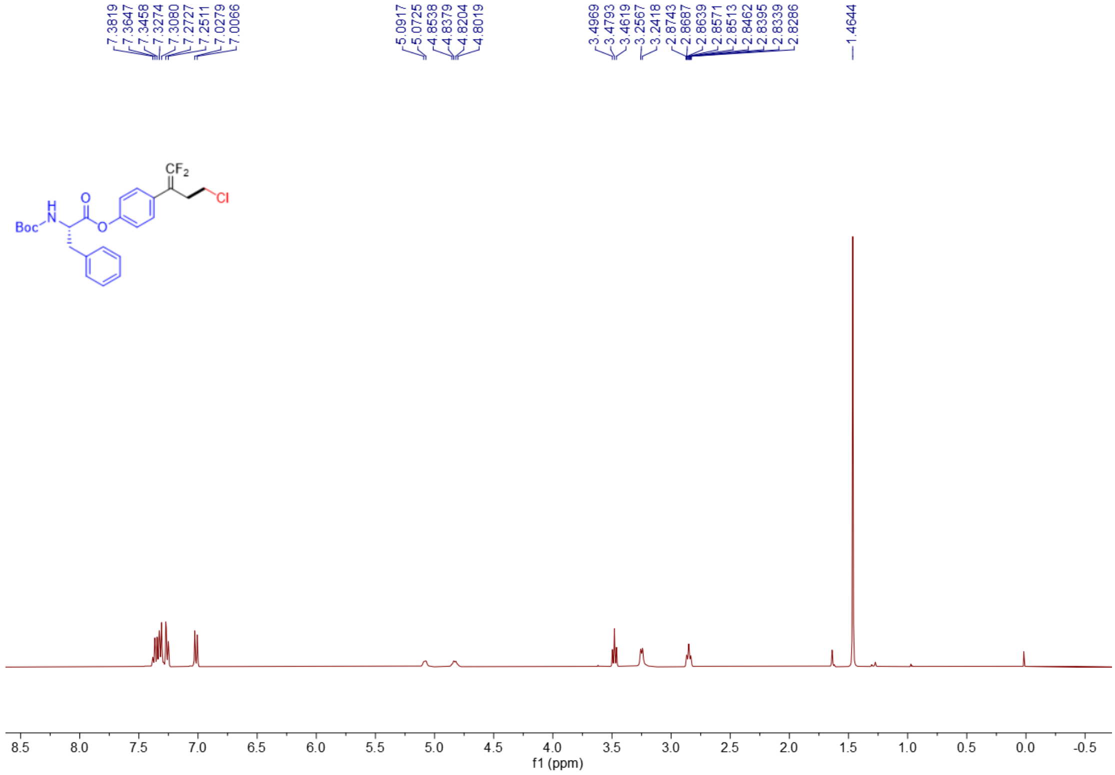

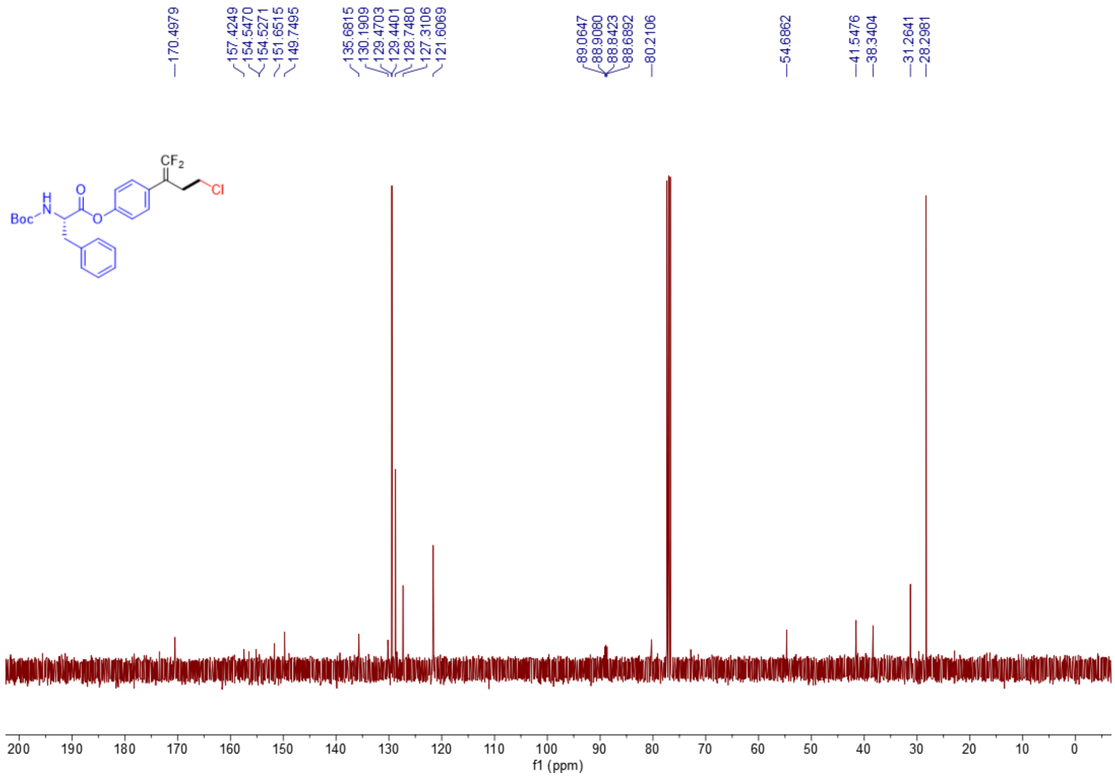
170.4979
157.4249
154.5470
154.5271
151.6515
149.7495
135.6815
130.1909
129.4703
129.4401
128.7480
127.3106
121.6069
89.0647
88.9080
88.8423
88.6892
80.2106
54.6862
41.5476
38.3404
31.2641
28.2981
CF2
H
N
O
O
Cl
Boc
200 190 180 170 160 150 140 130 120 110 100 90 80 70 60 50 40 30 20 10 0
f1 (ppm)

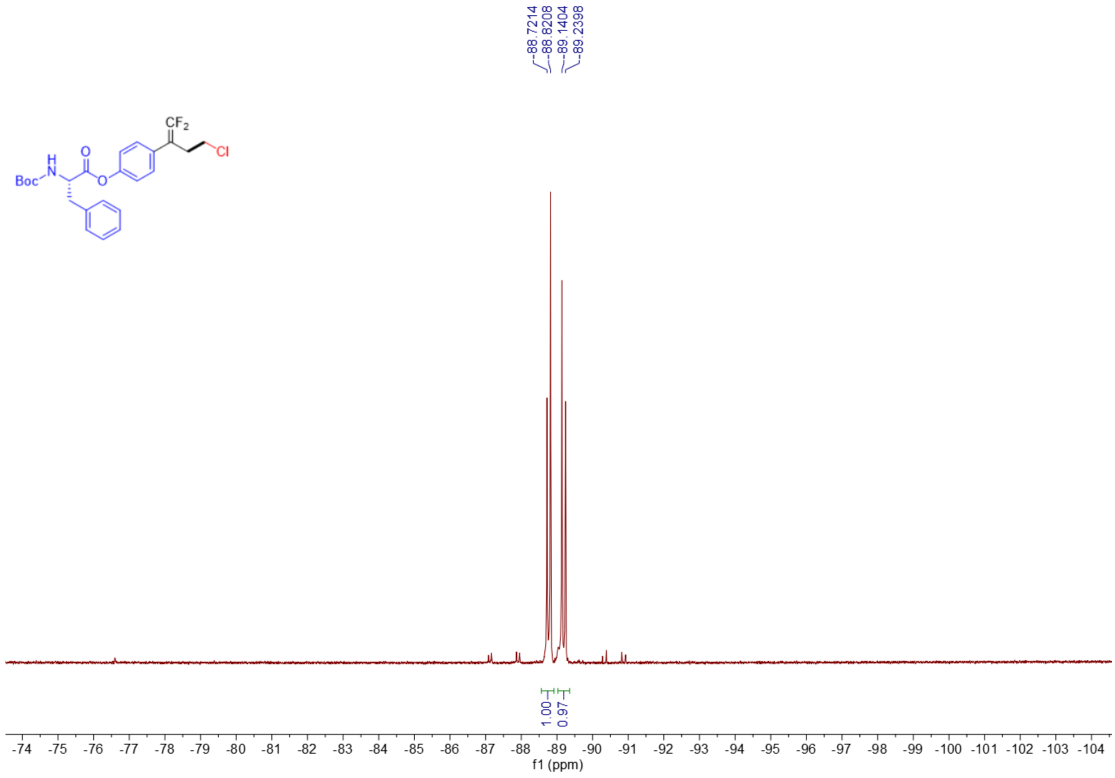
-88.7214
-88.8208
-89.1404
-89.2398
CF2
H
N
O
O
Cl
Boc
1.00
0.97
-74 -75 -76 -77 -78 -79 -80 -81 -82 -83 -84 -85 -86 -87 -88 -89 -90 -91 -92 -93 -94 -95 -96 -97 -98 -99 -100 -101 -102 -103 -104
f1 (ppm)

NMR spectrum of 35

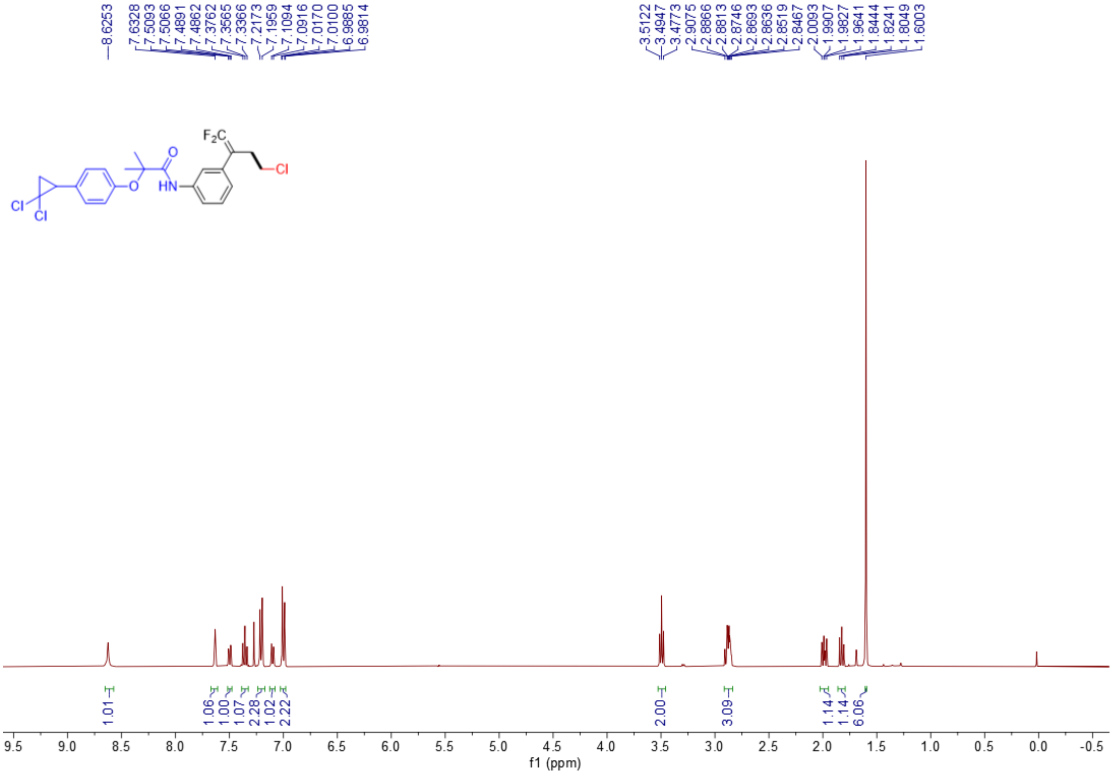

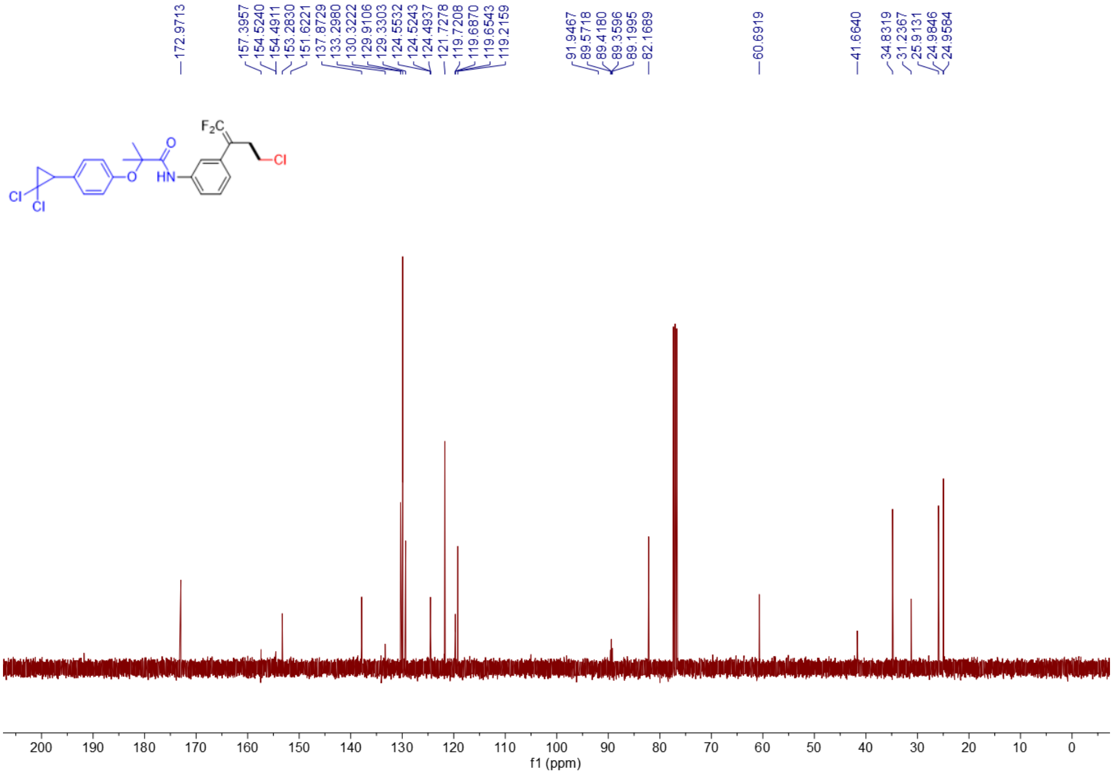

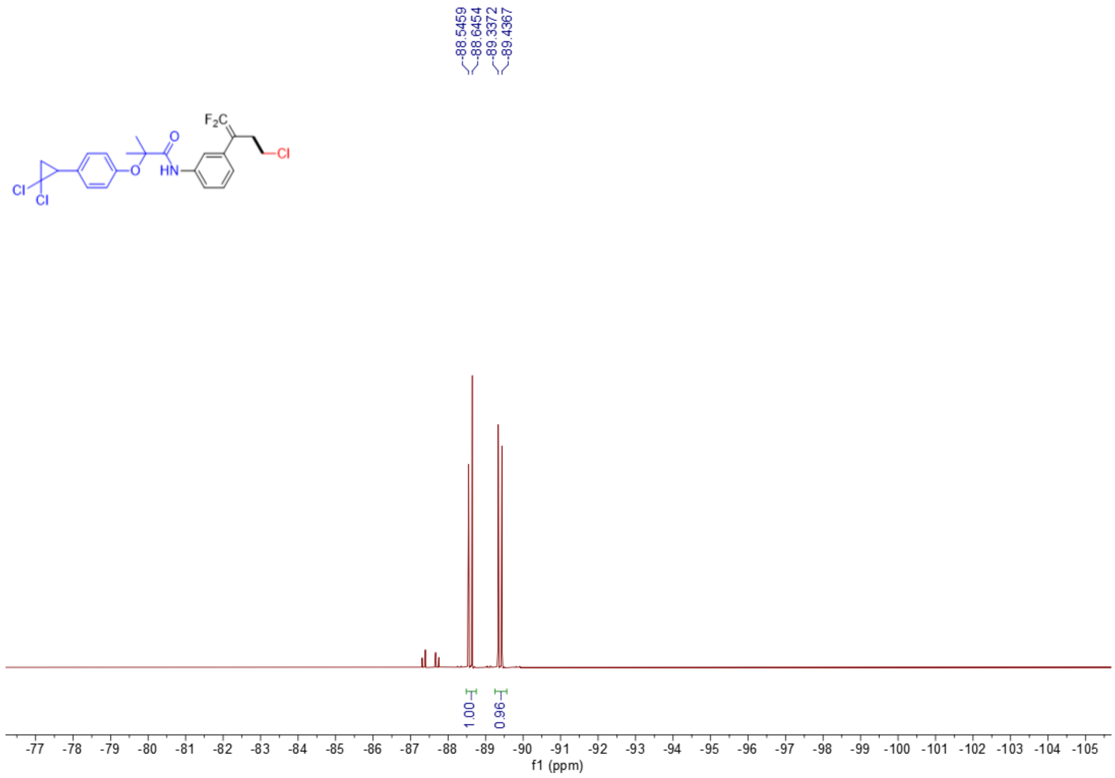

NMR spectrum of 36

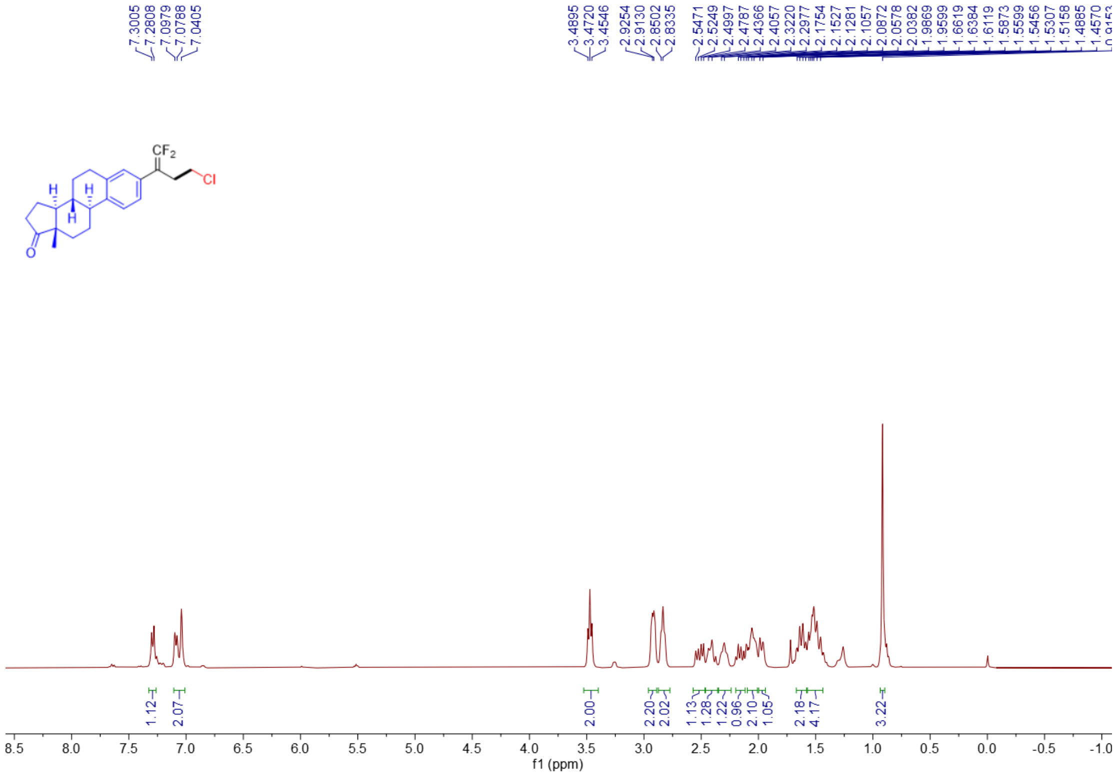

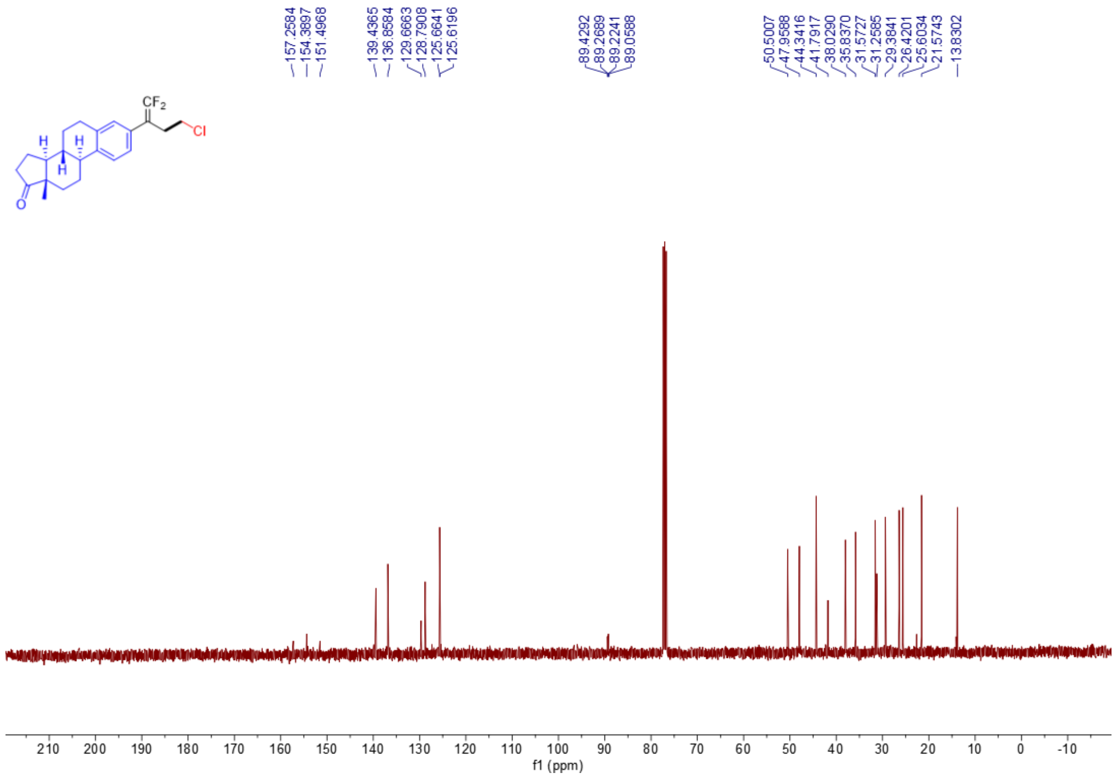

157.2584
154.3897
151.4968
139.4365
136.8584
129.6663
128.7908
125.6641
125.6196
89.4292
89.2689
89.2241
89.0588
50.5007
47.9588
44.3416
41.7917
38.0290
35.8370
31.5727
31.2585
29.3841
26.4201
25.6034
21.5743
13.8302
CF2
Cl
H
H
H
O
f1 (ppm)

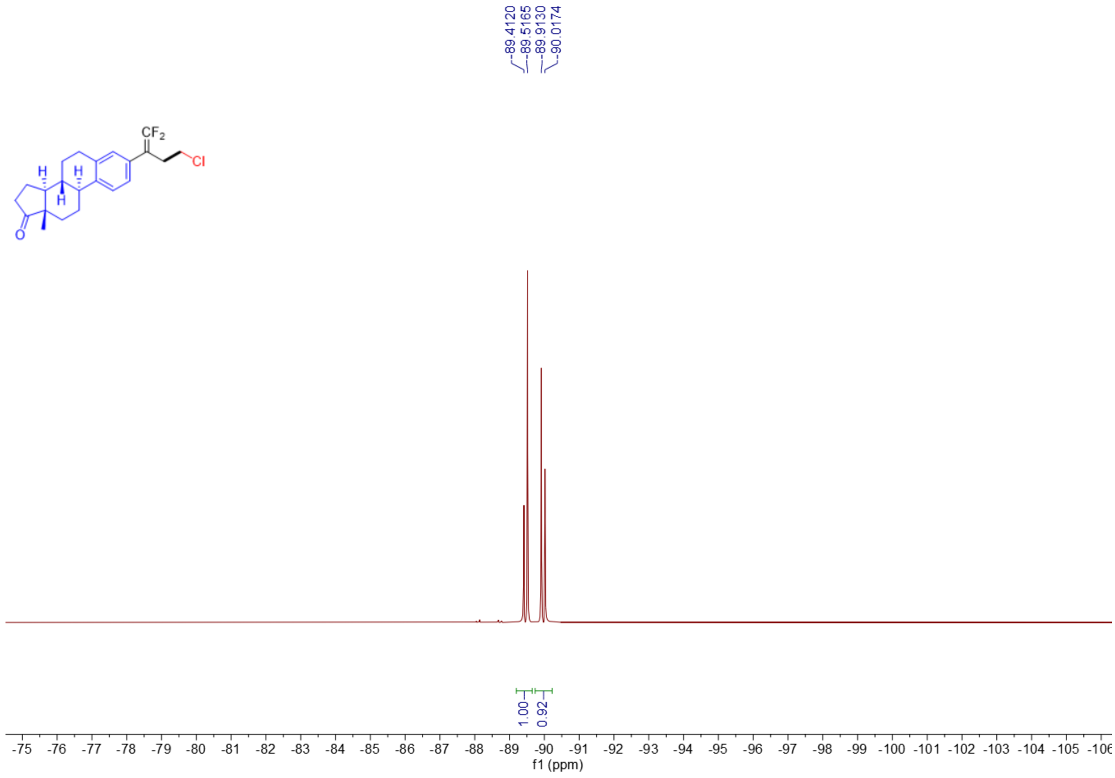

-89.4120
-89.5165
-89.9130
-90.0174
CF2
Cl
H
H
H
O
1.00
0.92
f1 (ppm)

NMR spectrum of 37

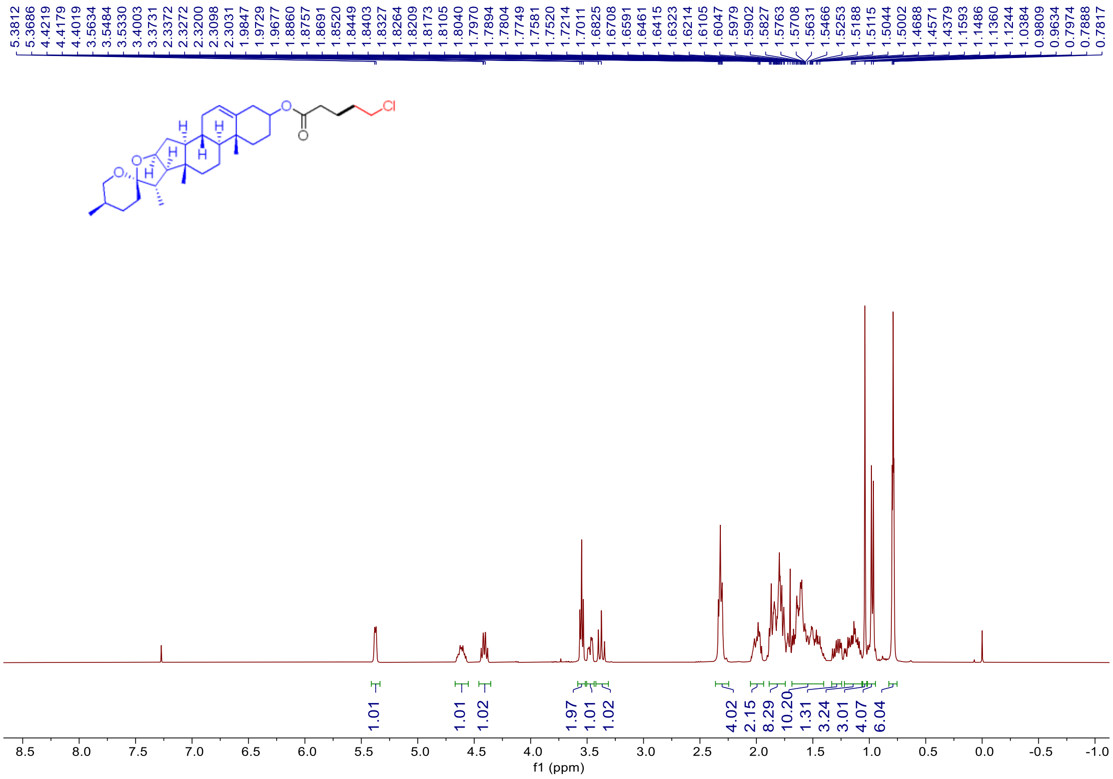

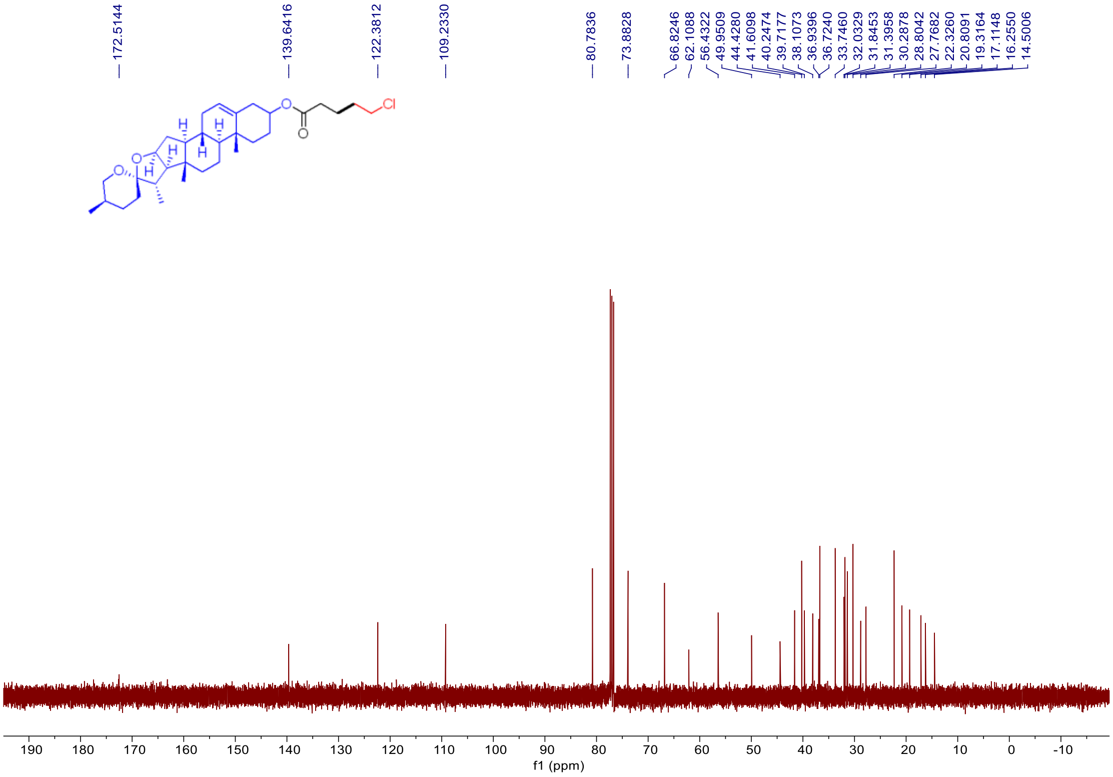

NMR spectrum of 38

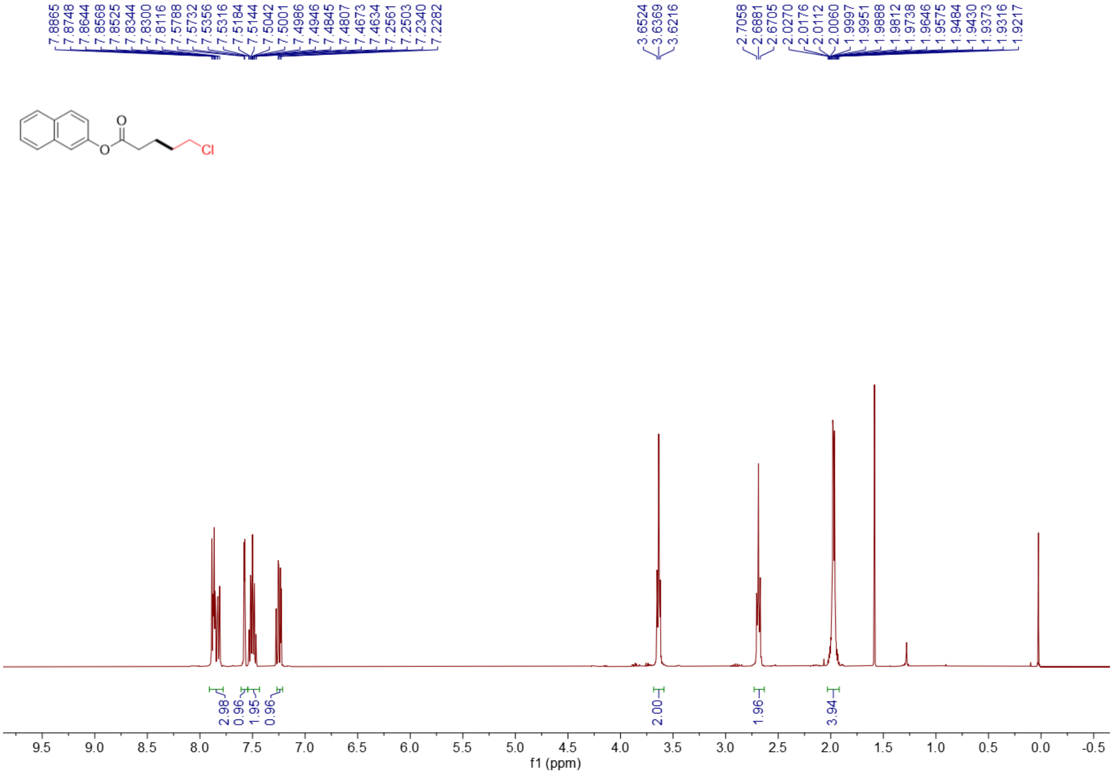

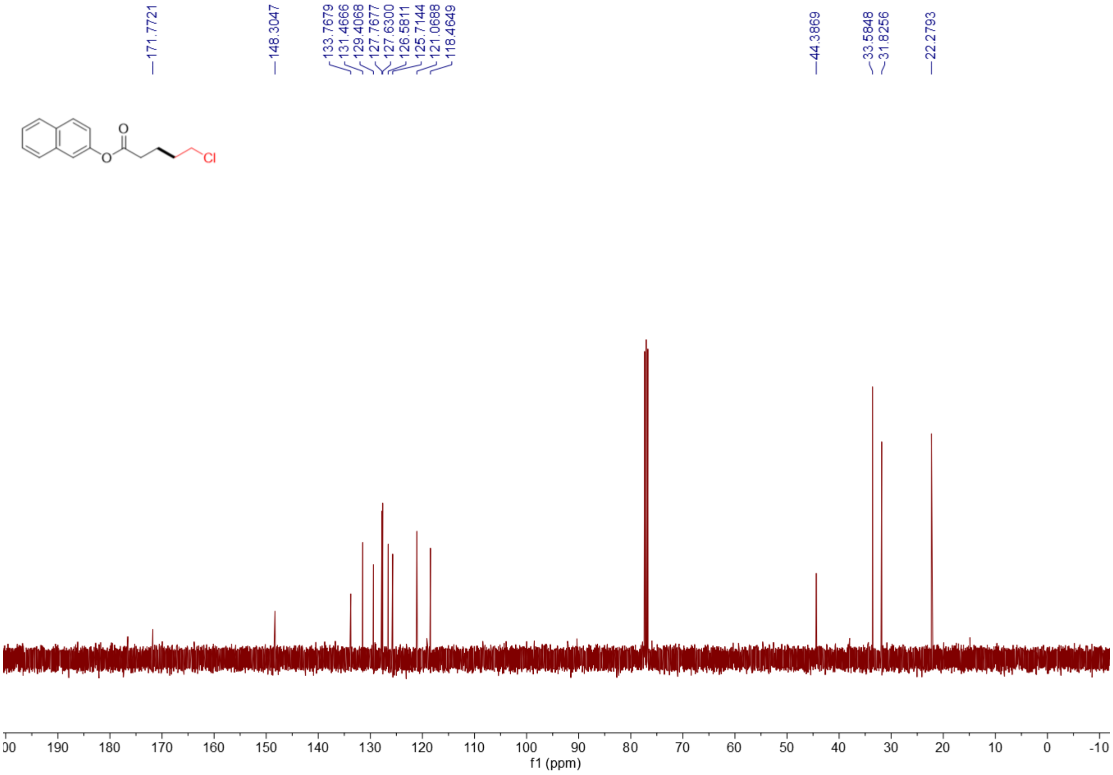

NMR spectrum of 39

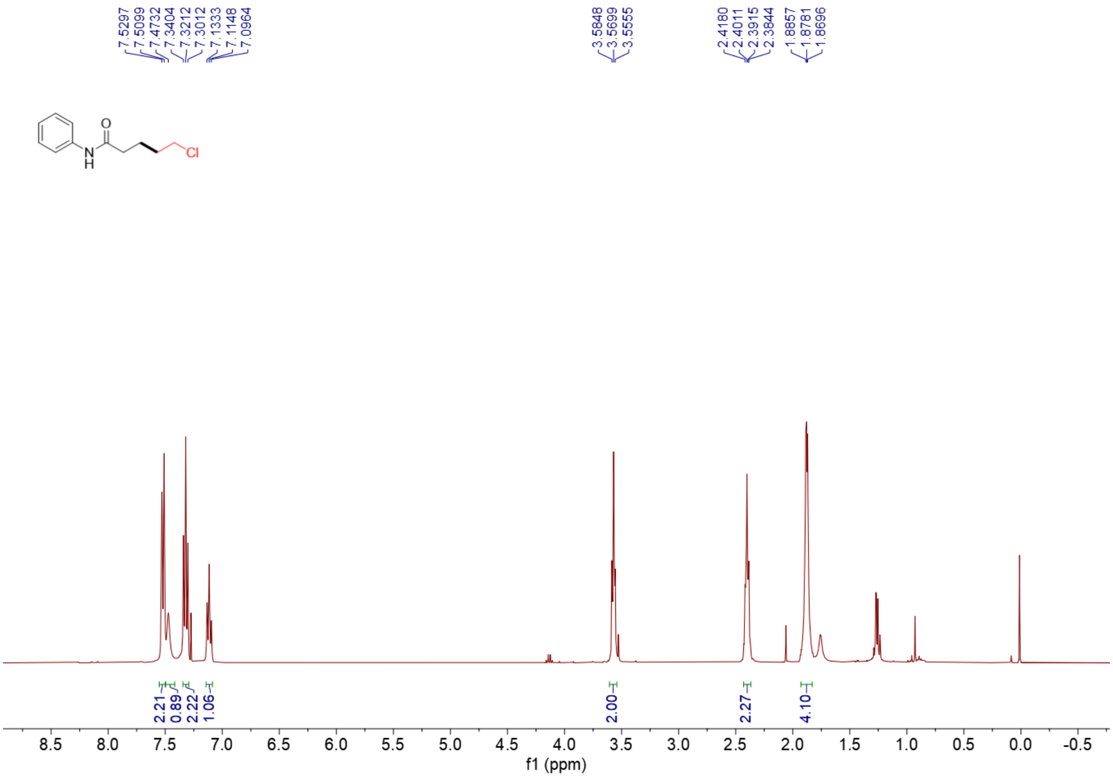

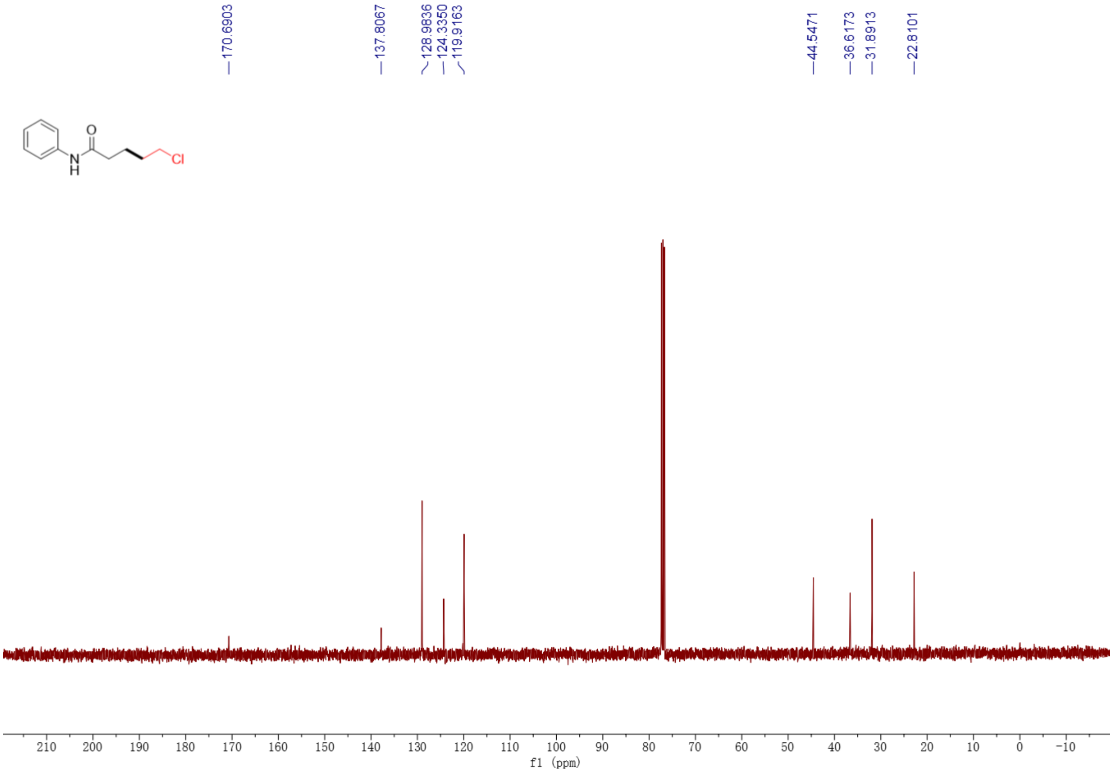

NMR spectrum of 40

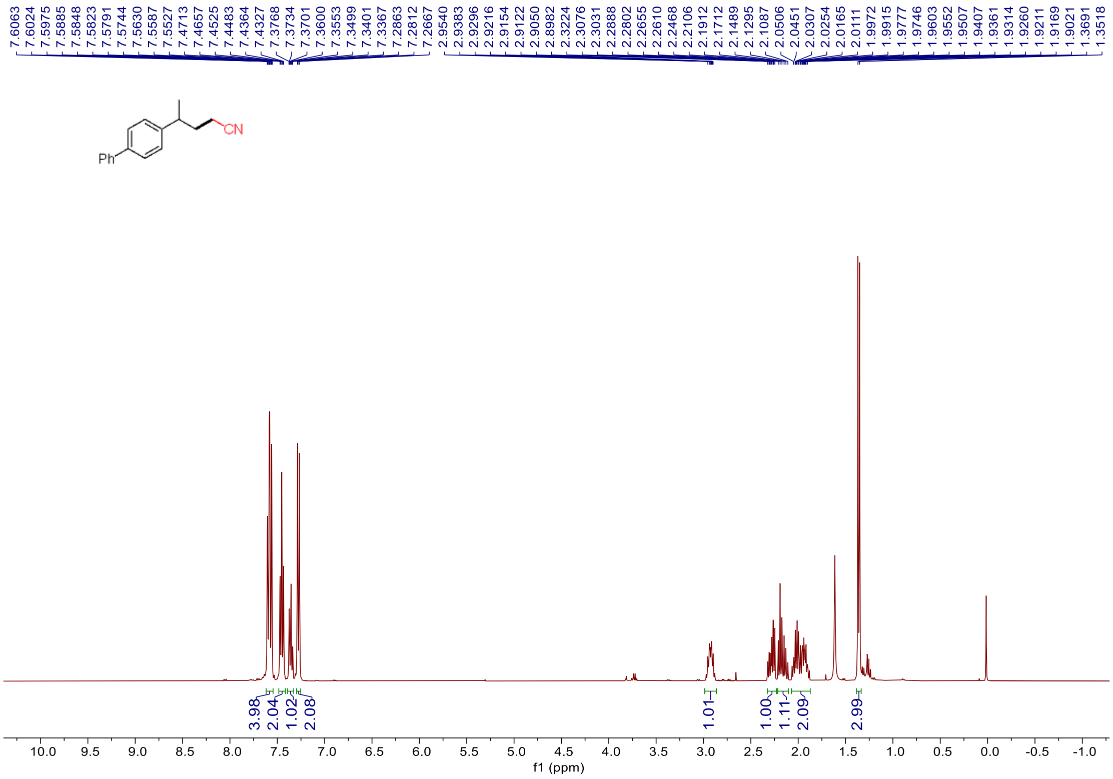

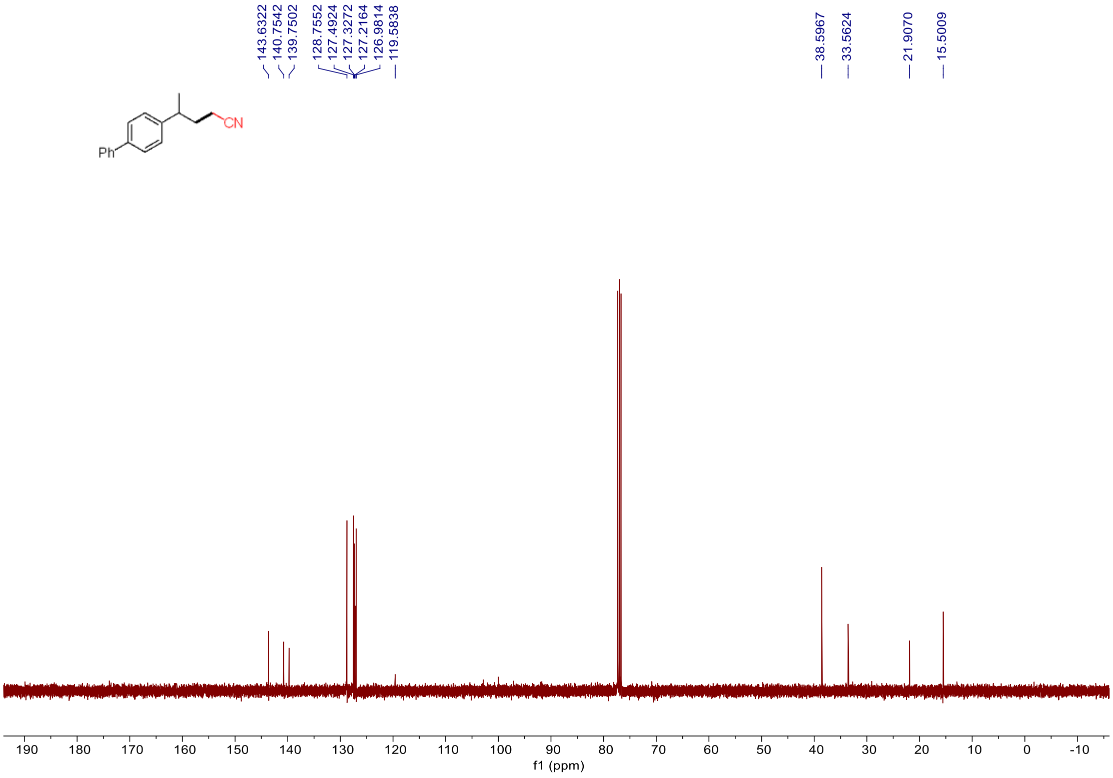

NMR spectrum of 41

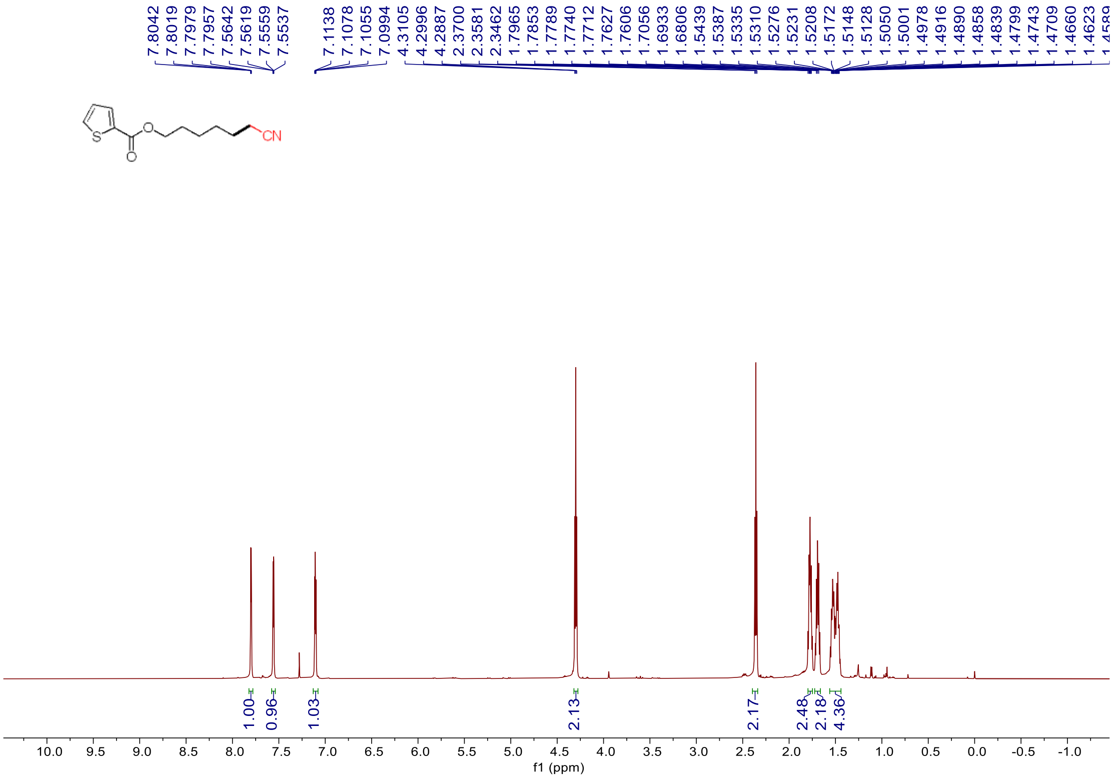

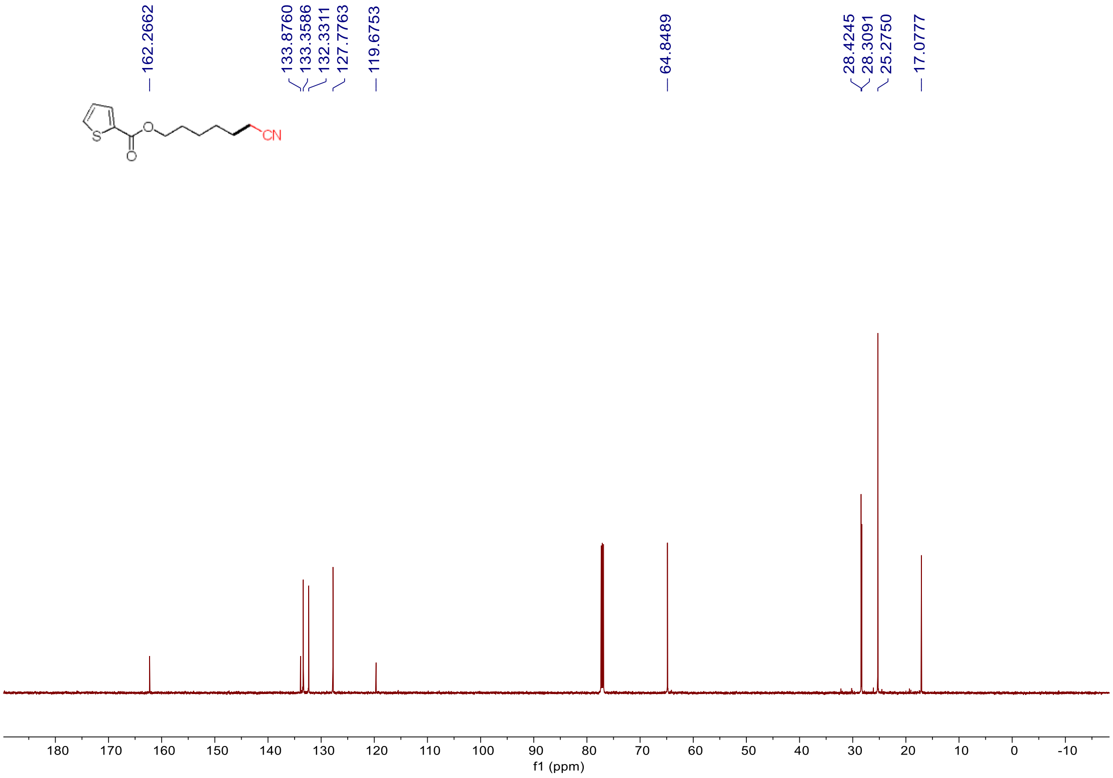

NMR spectrum of 42

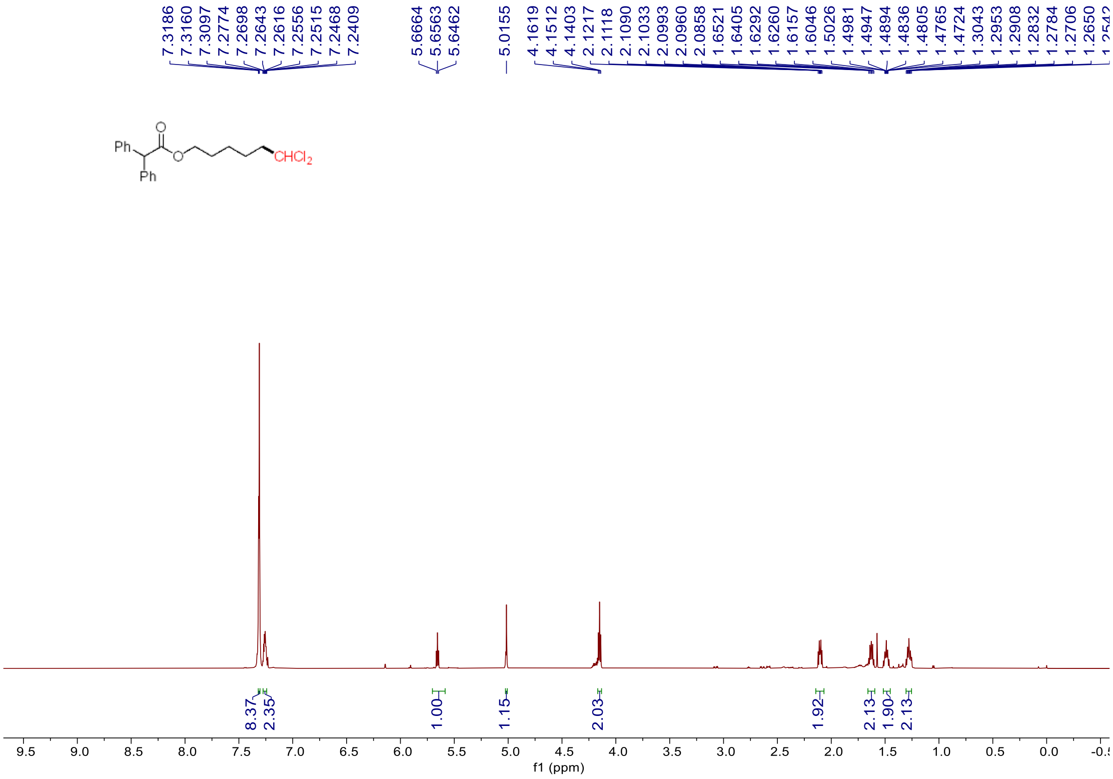

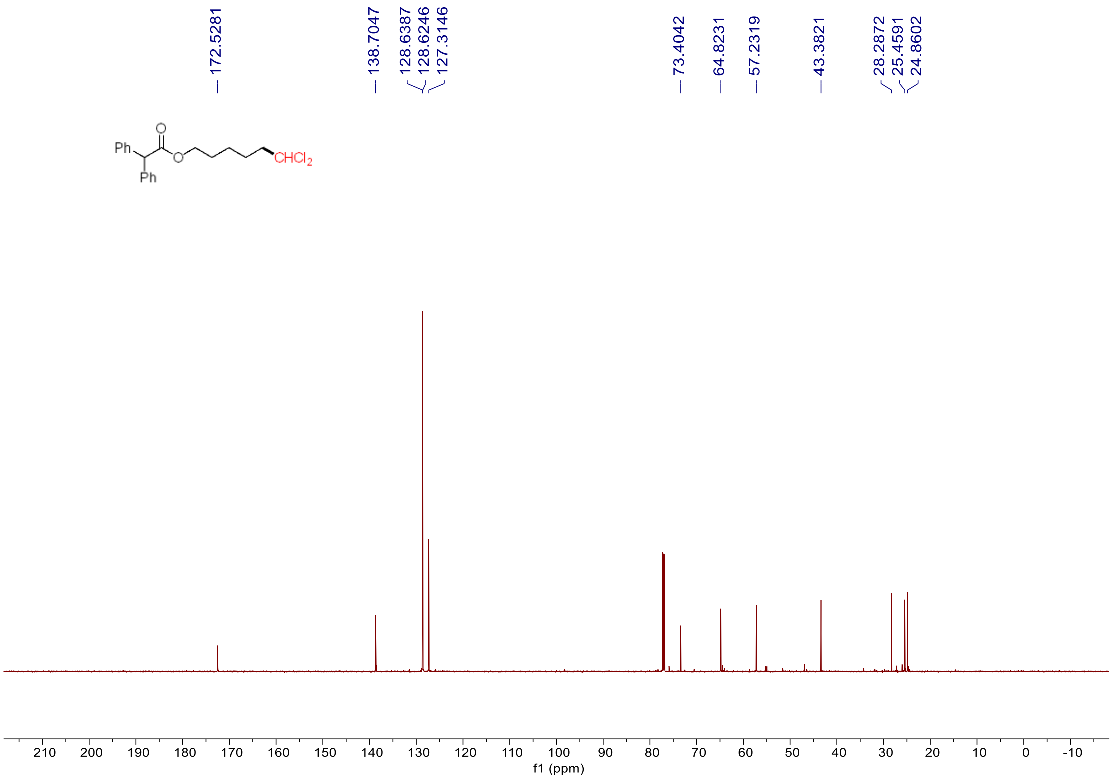

NMR spectrum of 43

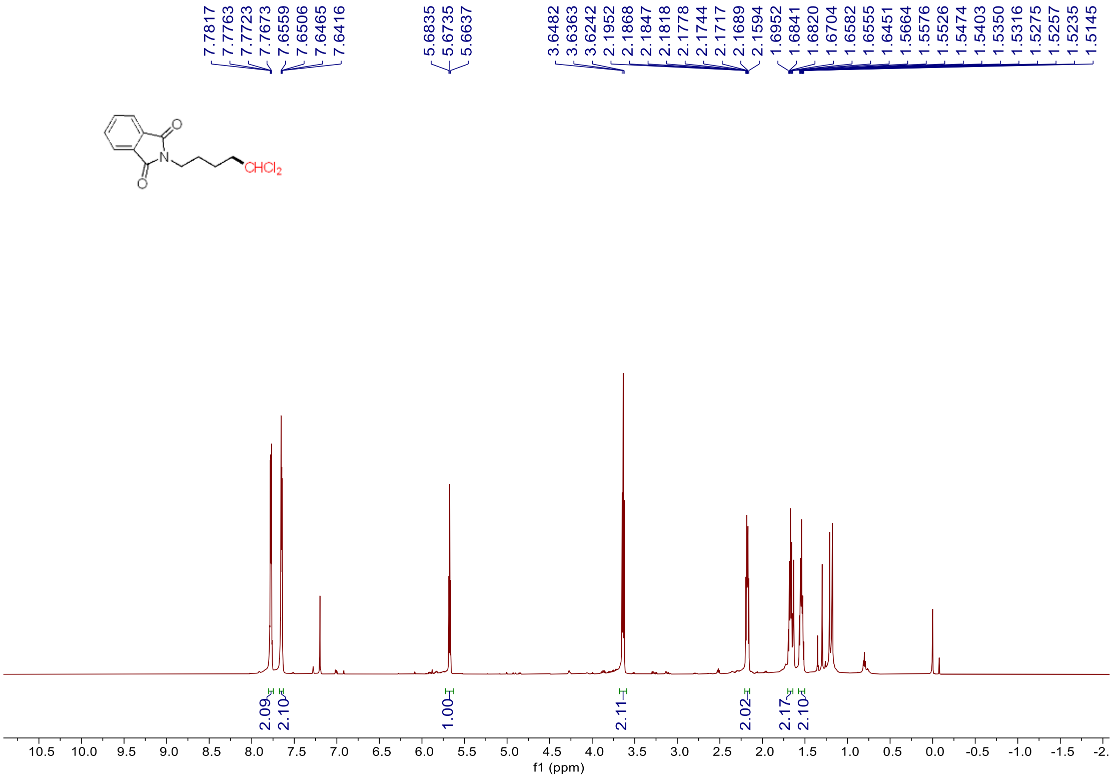

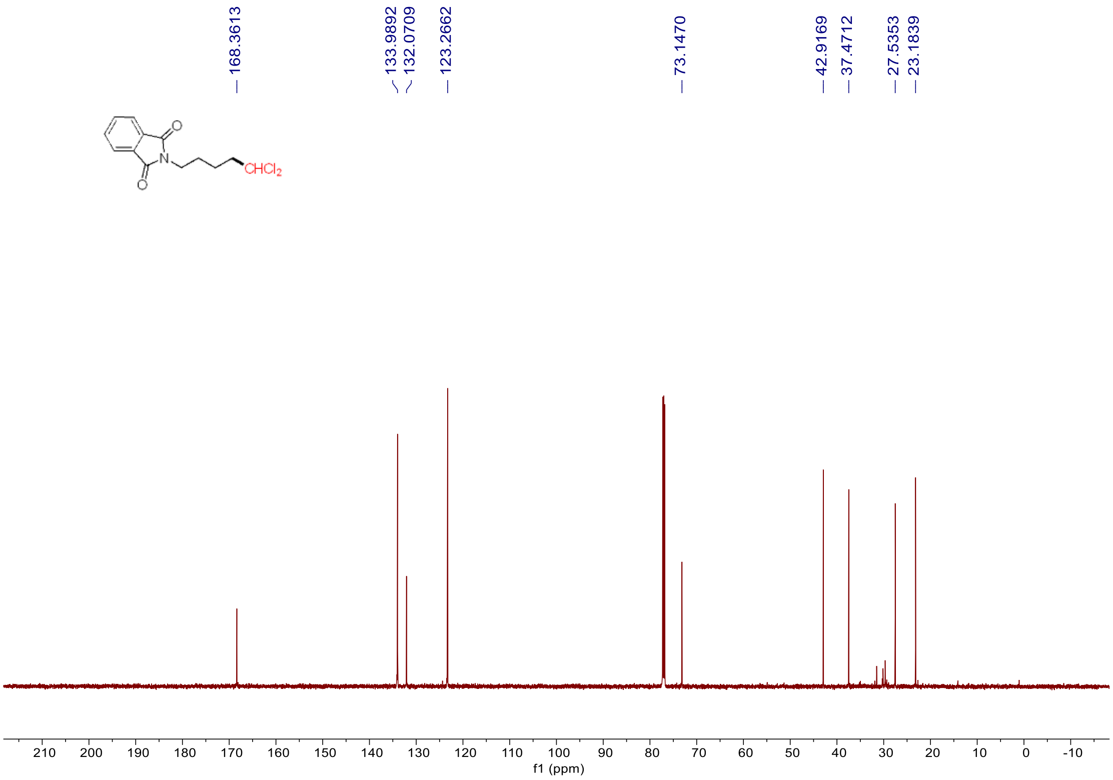

NMR spectrum of 44

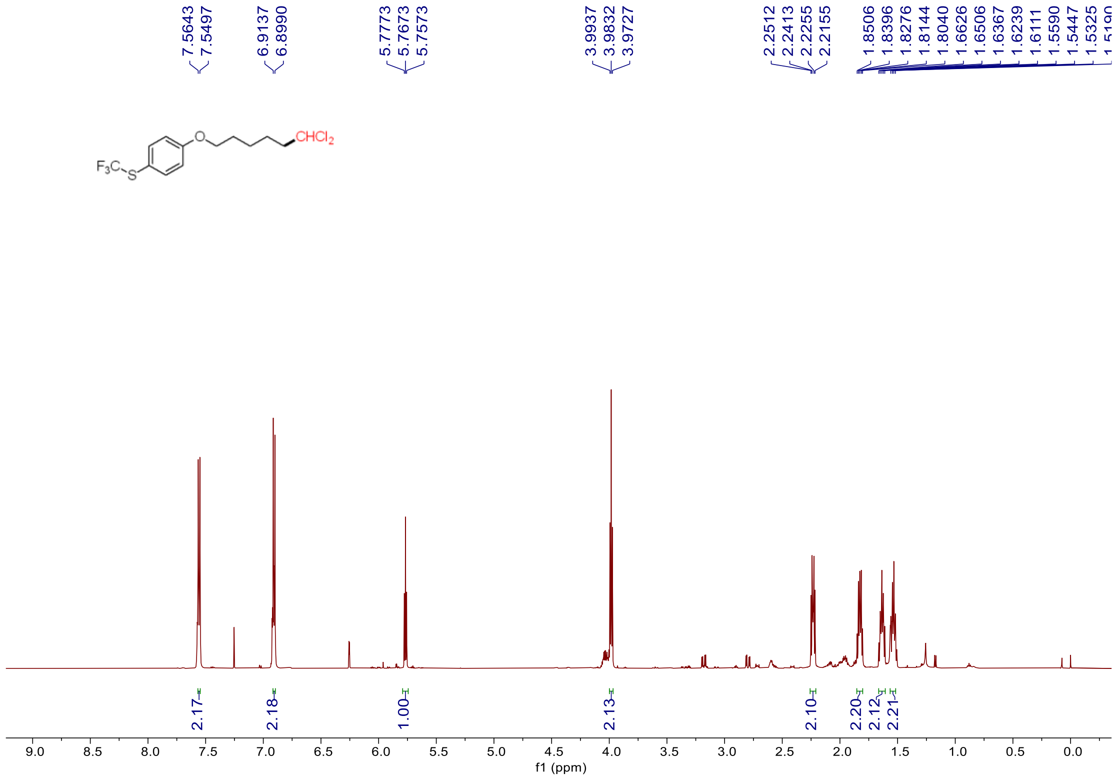

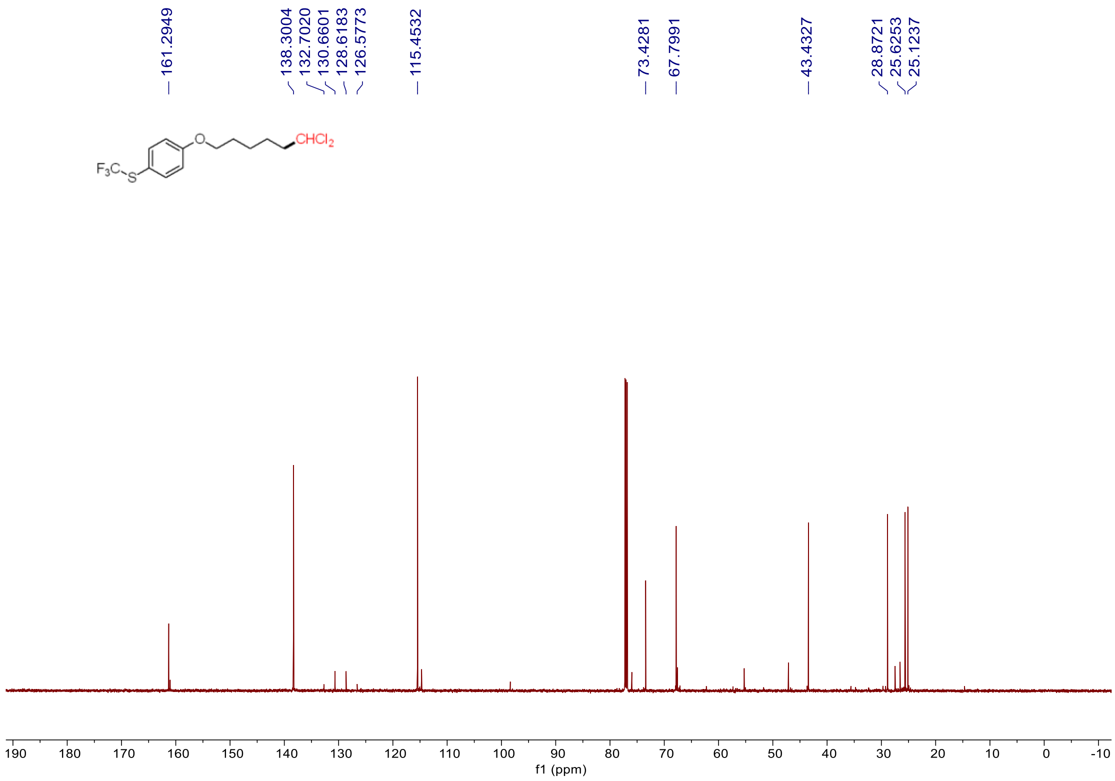

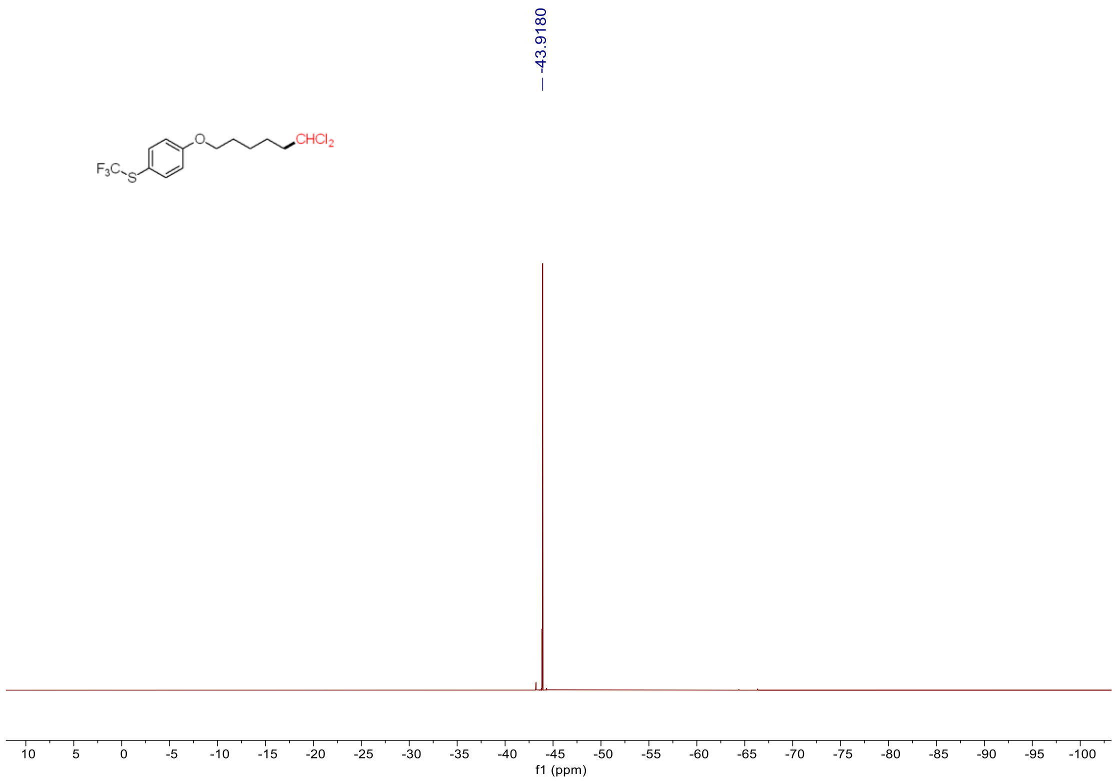

NMR spectrum of 45

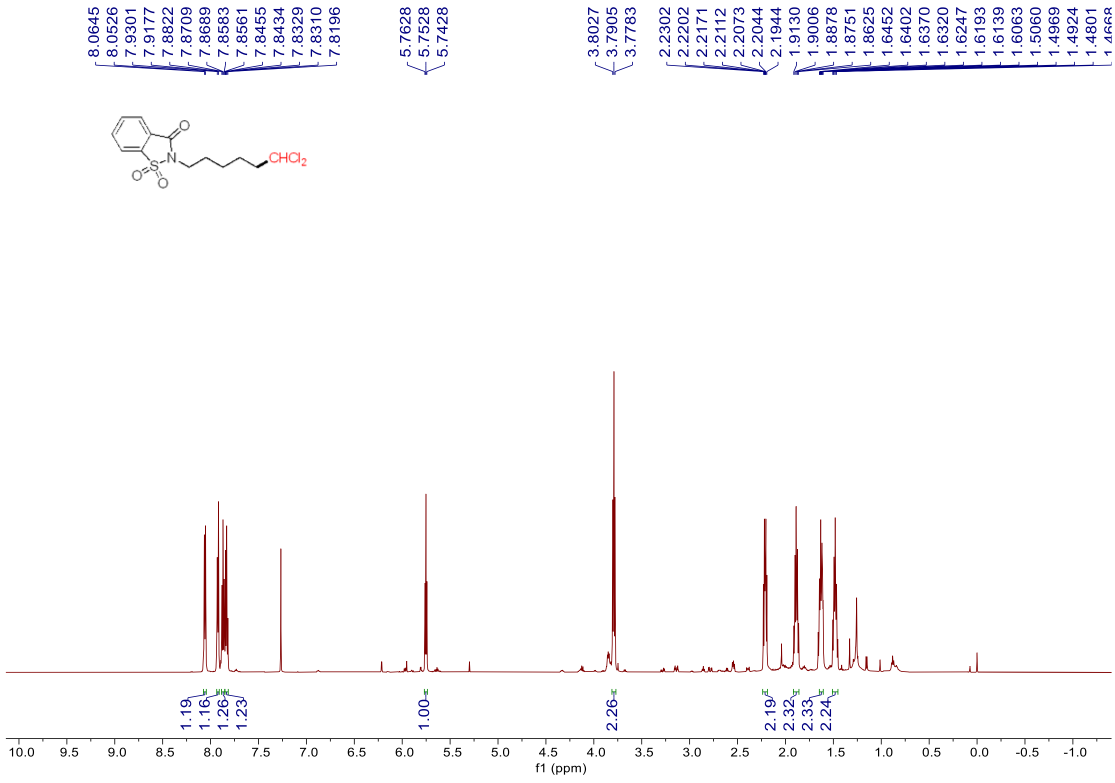

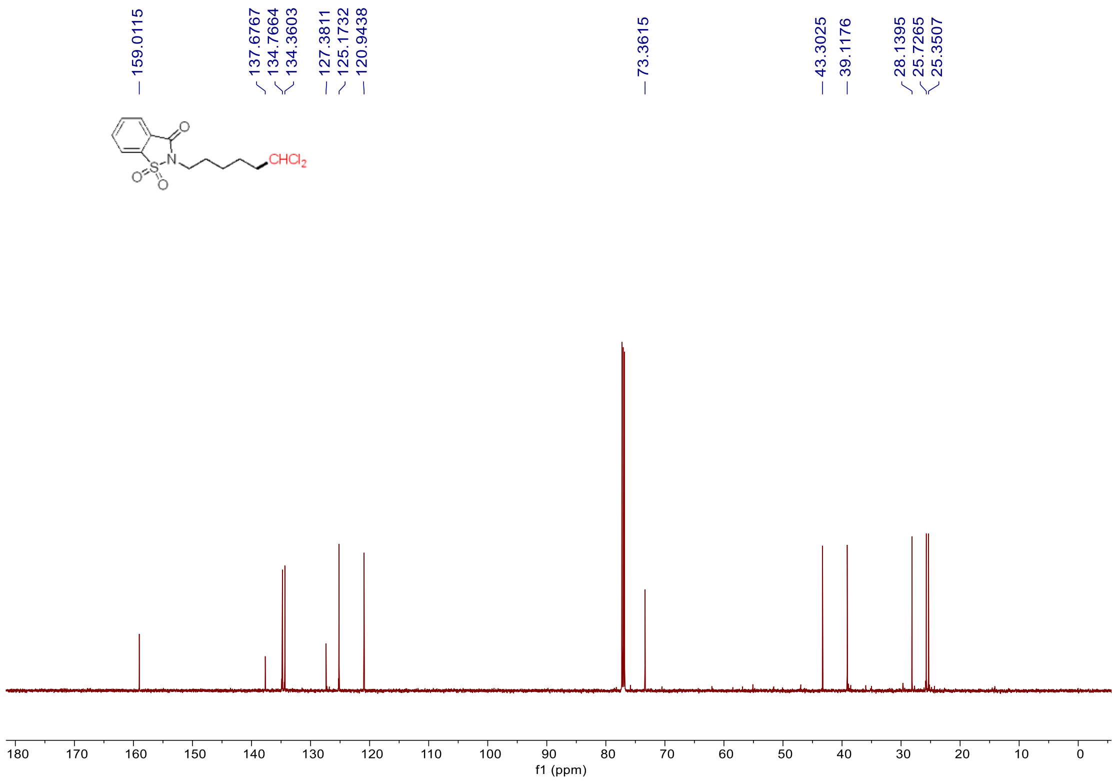

NMR spectrum of 46

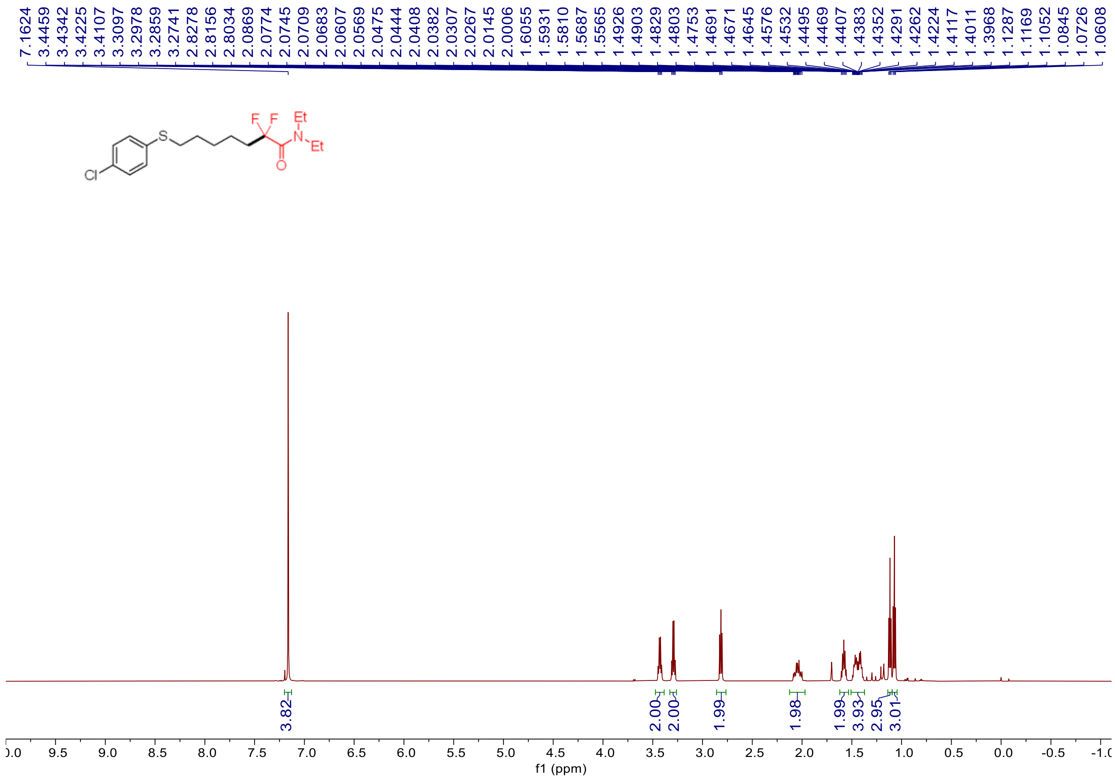

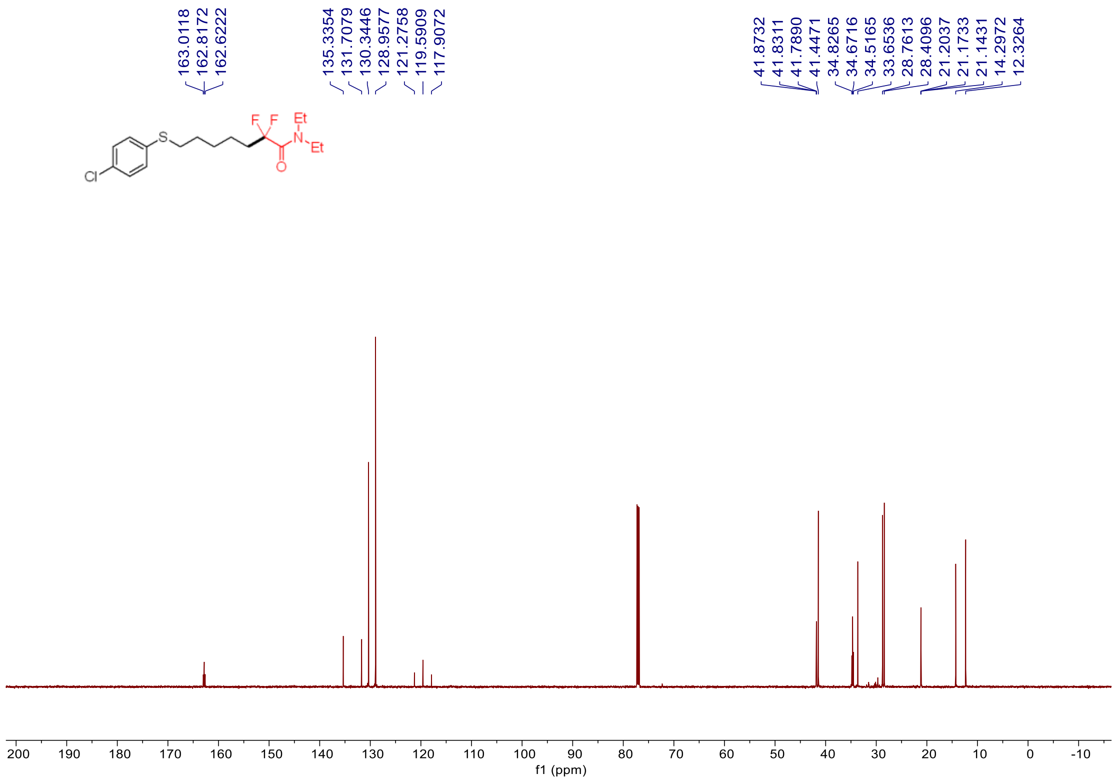
163.0118
162.8172
162.6222
135.3354
131.7079
130.3446
128.9577
121.2758
119.5909
117.9072
41.8732
41.8311
41.7890
41.4471
34.8265
34.6716
34.5165
33.6536
28.7613
28.4096
21.2037
21.1733
21.1431
14.2972
12.3264
S
F F
Et
N
Et
O
Cl
f1 (ppm)

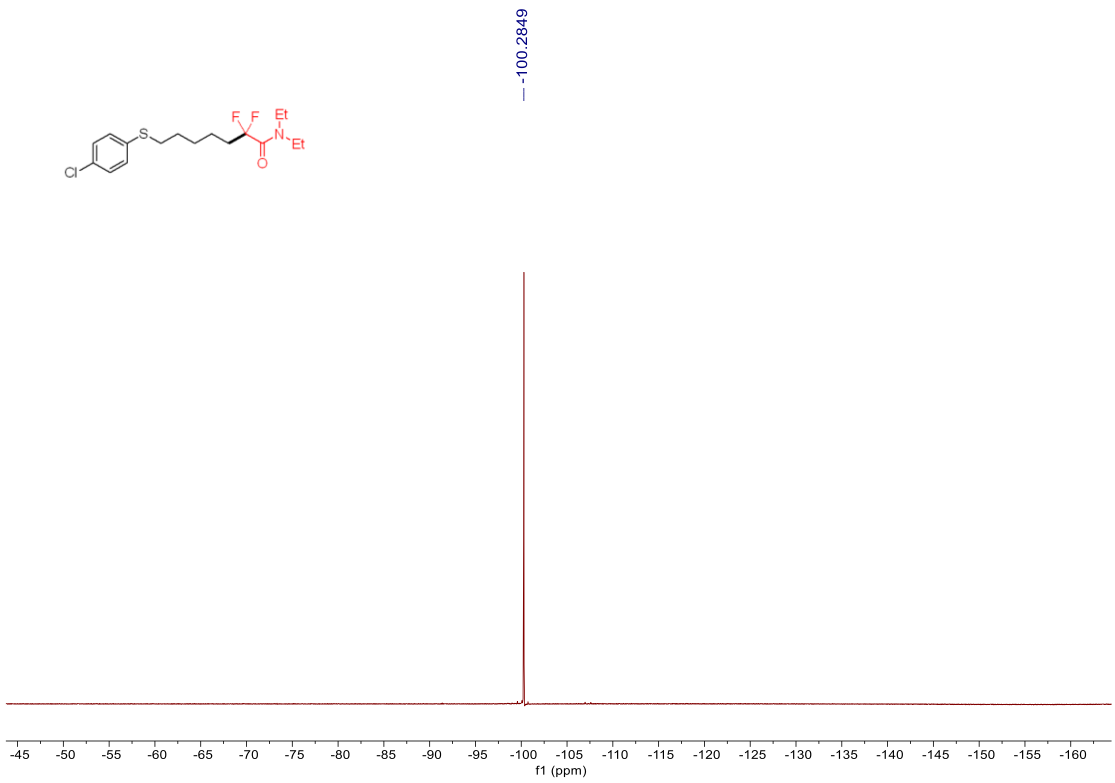
-100.2849
S
F F
Et
N
Et
O
Cl
f1 (ppm)

NMR spectrum of 47

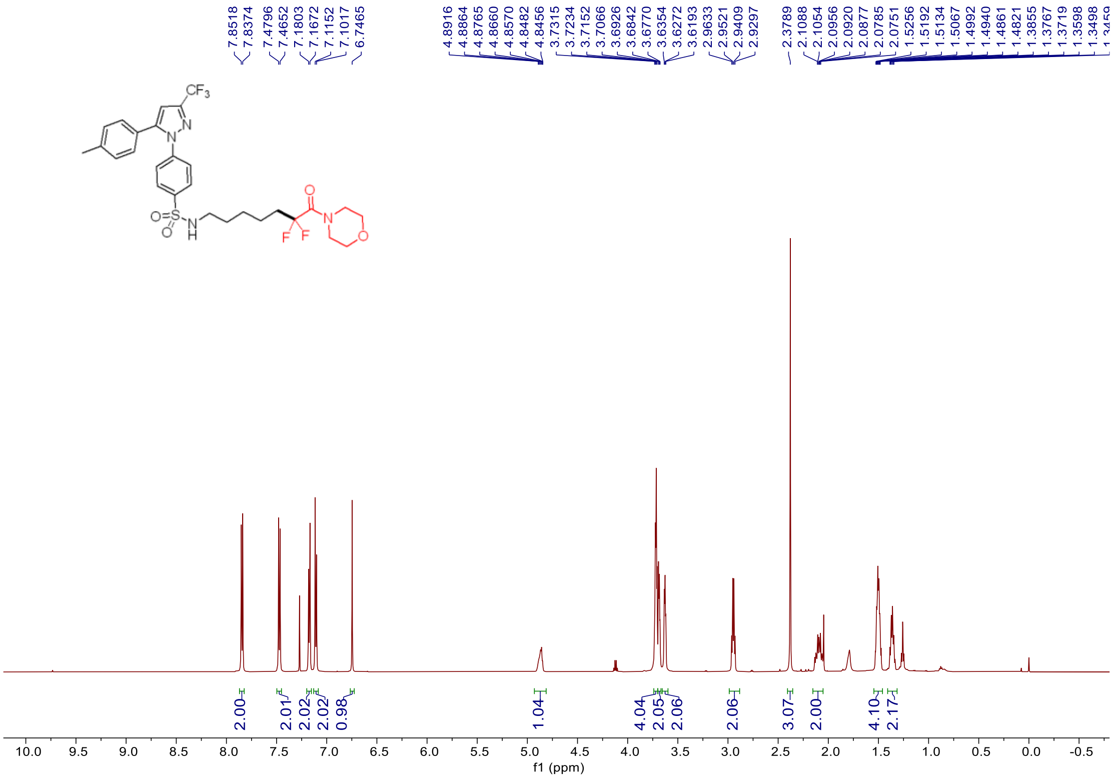

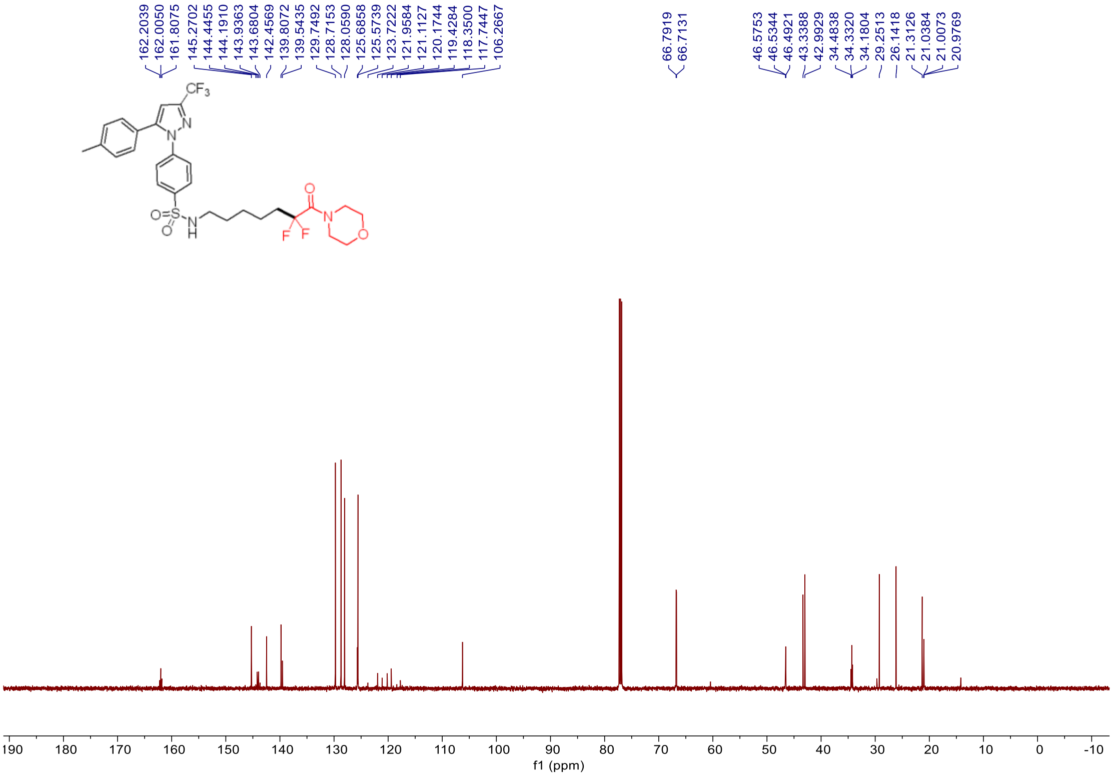

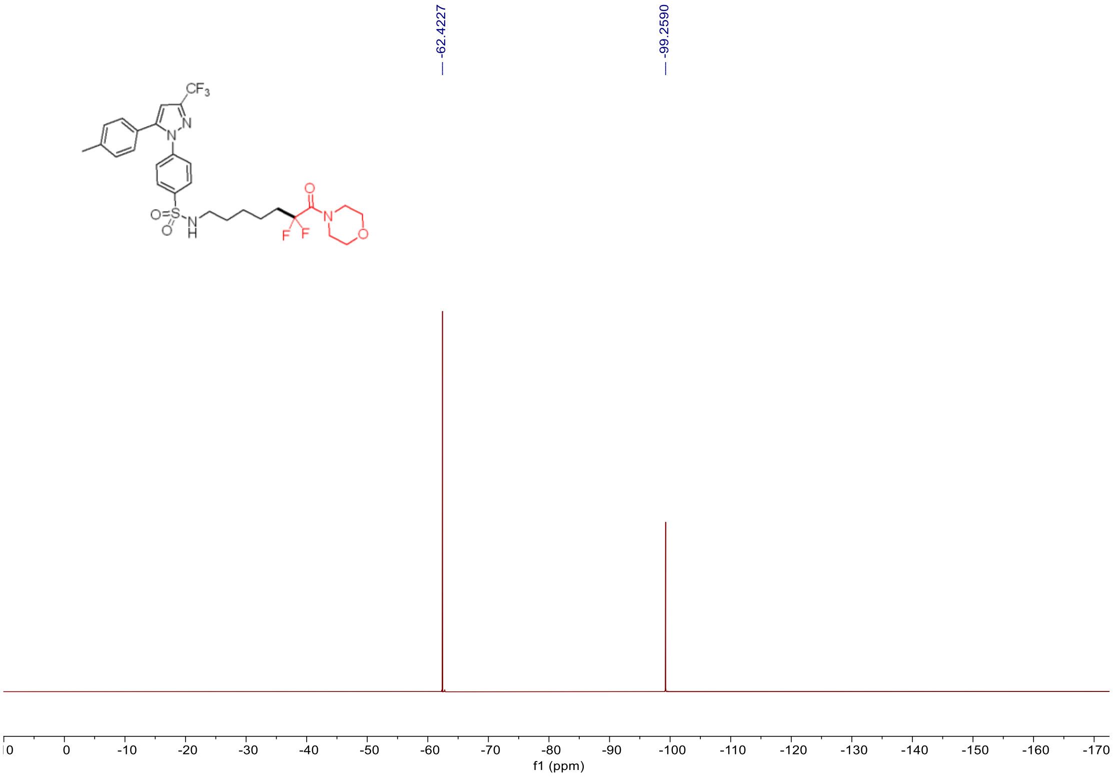

NMR spectrum of 48

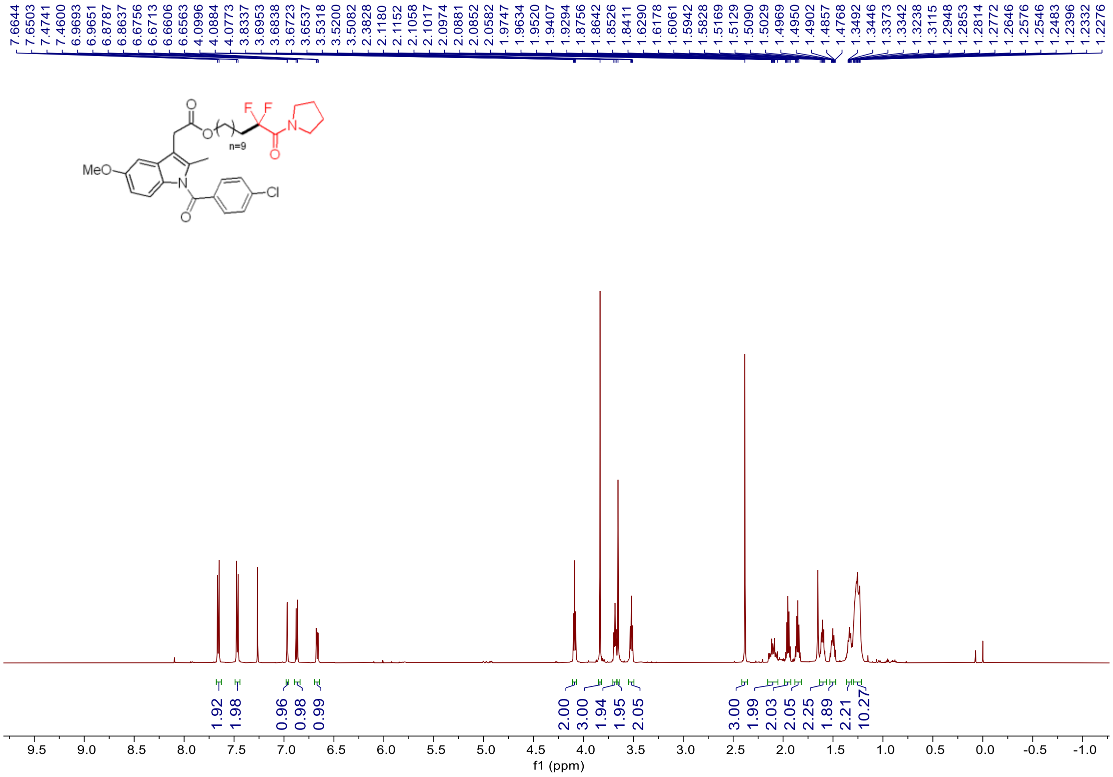

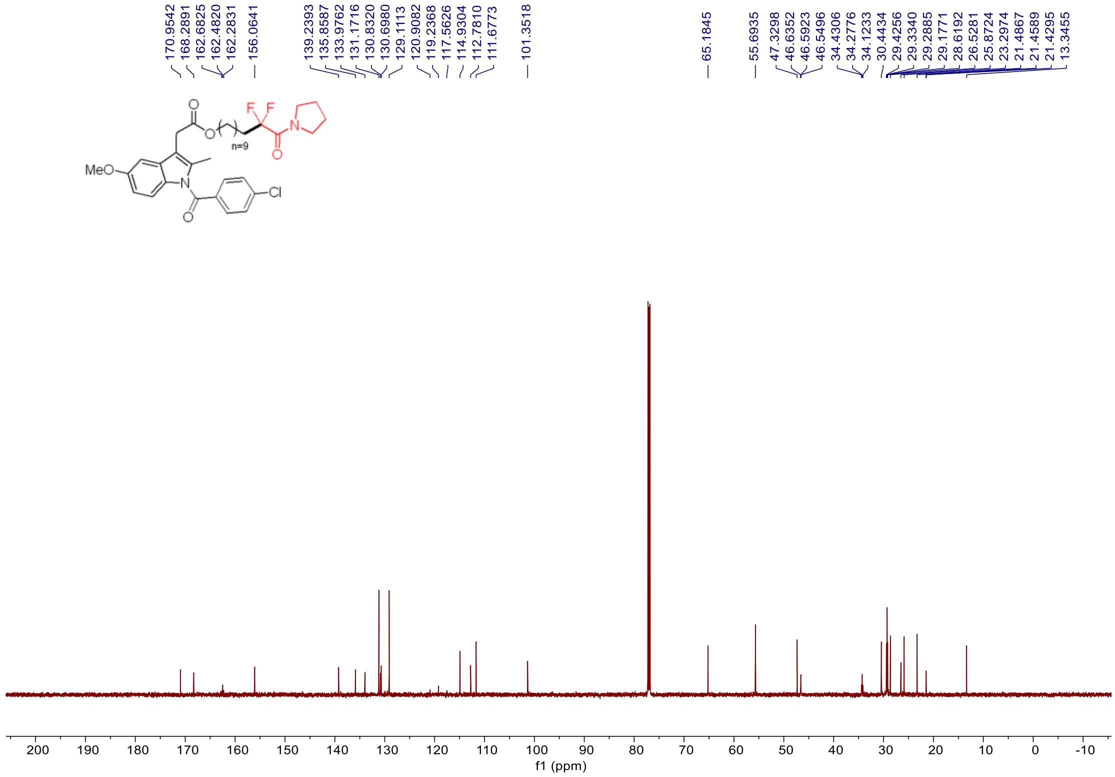
170.9542
168.2891
162.6825
162.4820
162.2831
156.0641
139.2393
135.8587
133.9762
131.1716
130.8320
130.6980
129.1113
120.9082
119.2368
117.5626
114.9304
112.7810
111.6773
101.3518
65.1845
55.6935
47.3298
46.6352
46.5923
46.5496
34.4306
34.2776
34.1233
30.4434
29.4256
29.3340
29.2885
29.1771
28.6192
26.5281
25.8724
23.2974
21.4867
21.4589
21.4295
13.3455
MeO
N
O
Cl
F F
n=9
f1 (ppm)

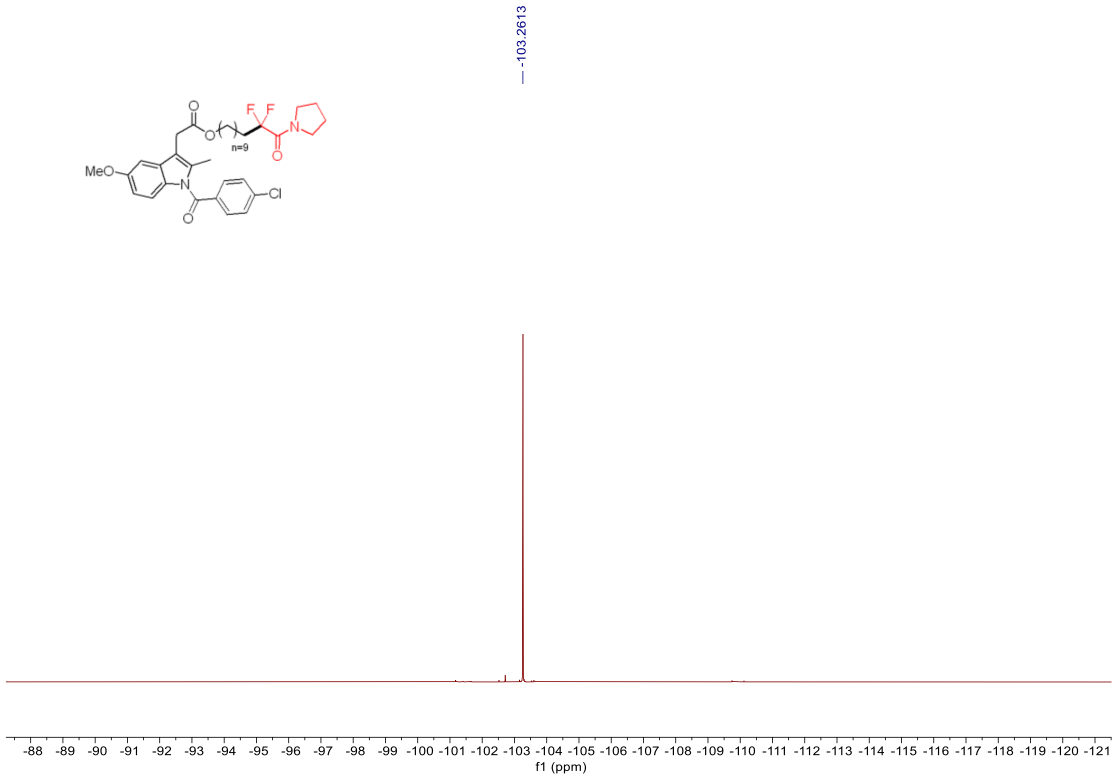
-103.2613
MeO
N
O
Cl
F F
n=9
f1 (ppm)

NMR spectrum of 49

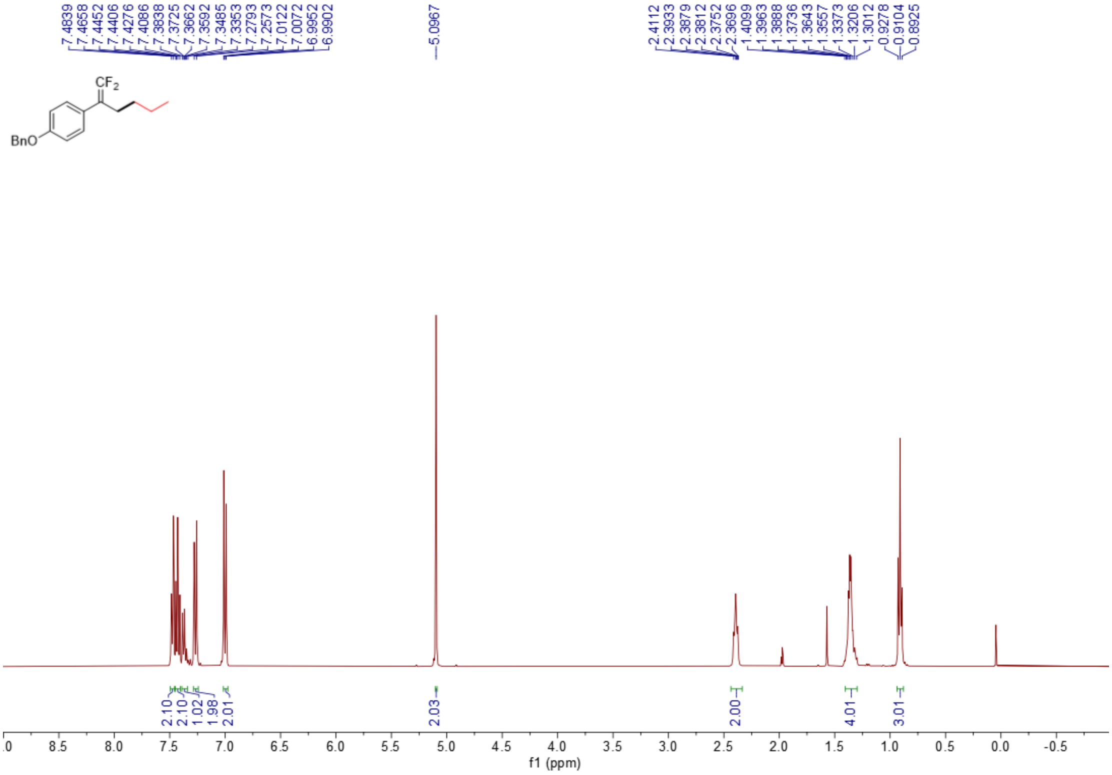

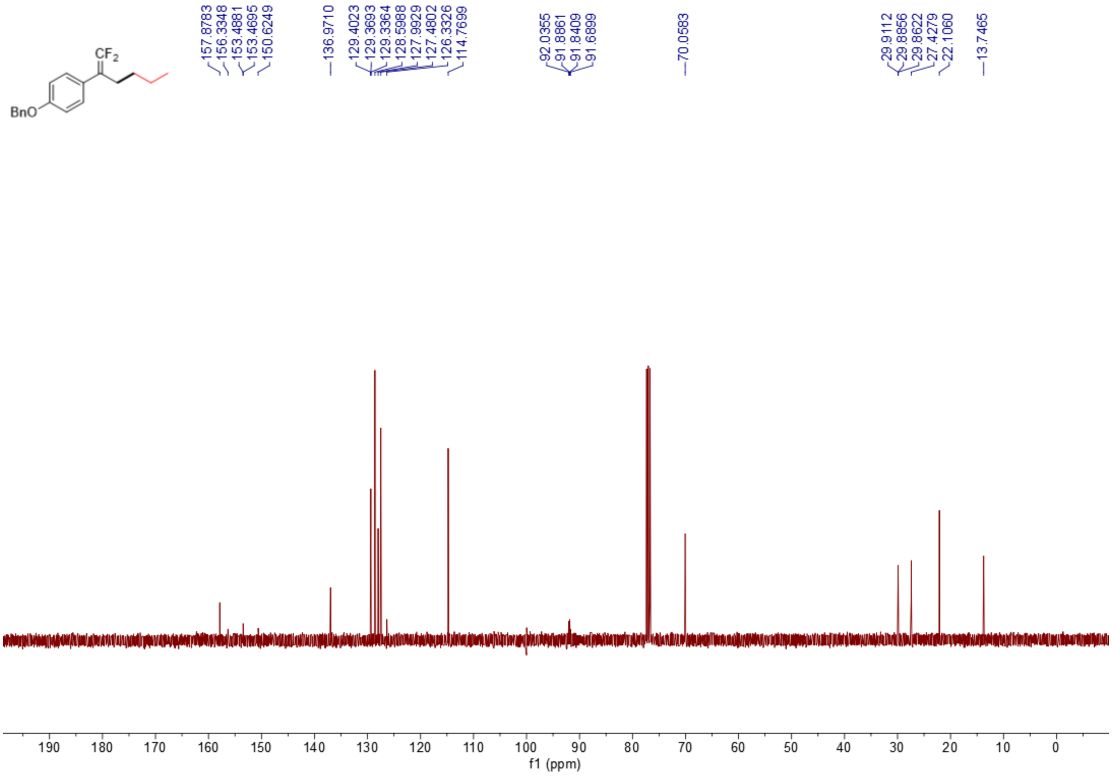

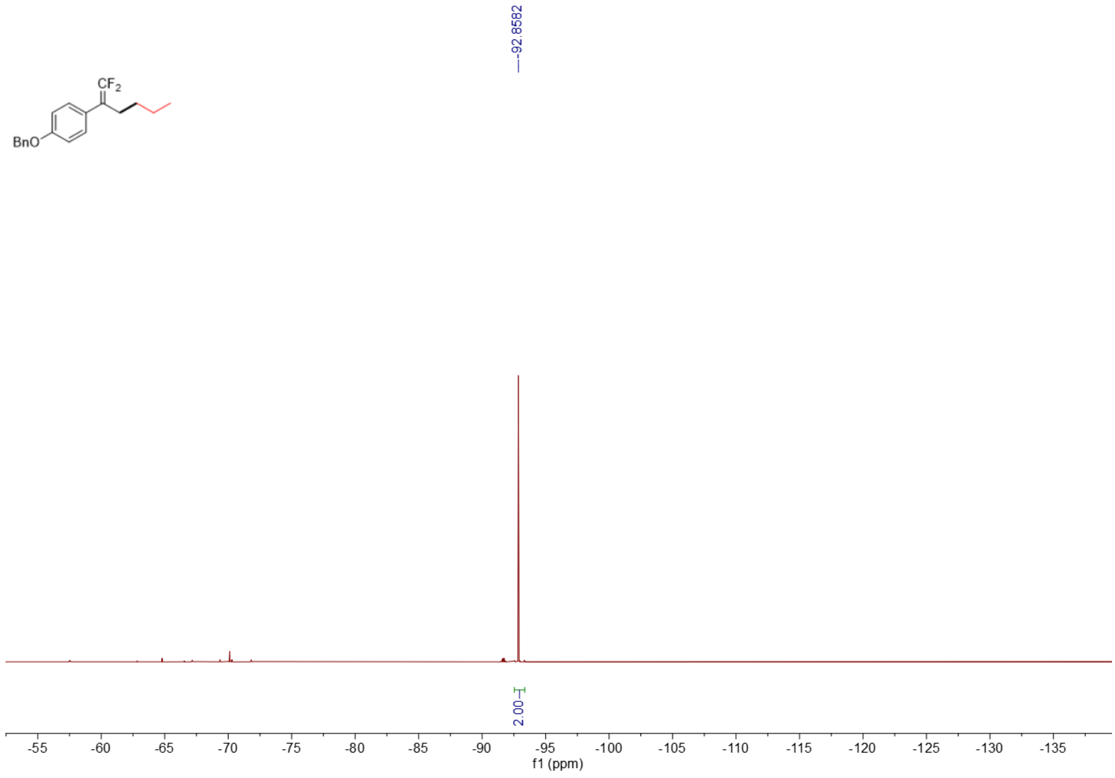

NMR spectrum of 50

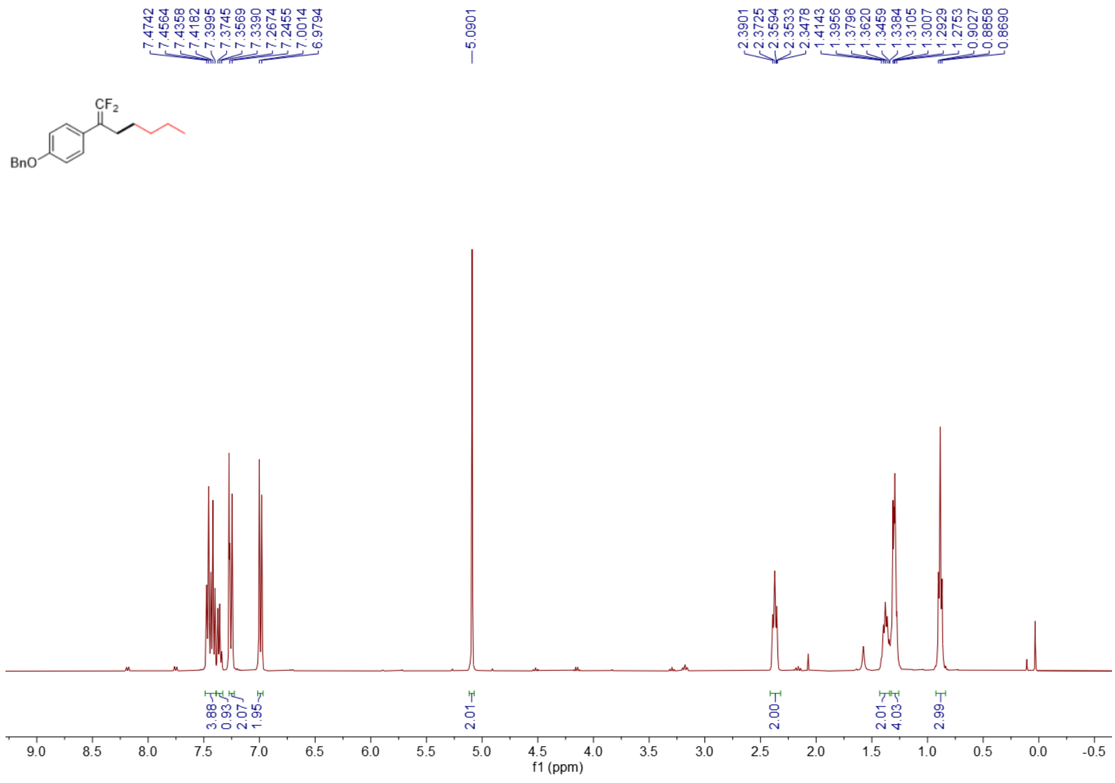

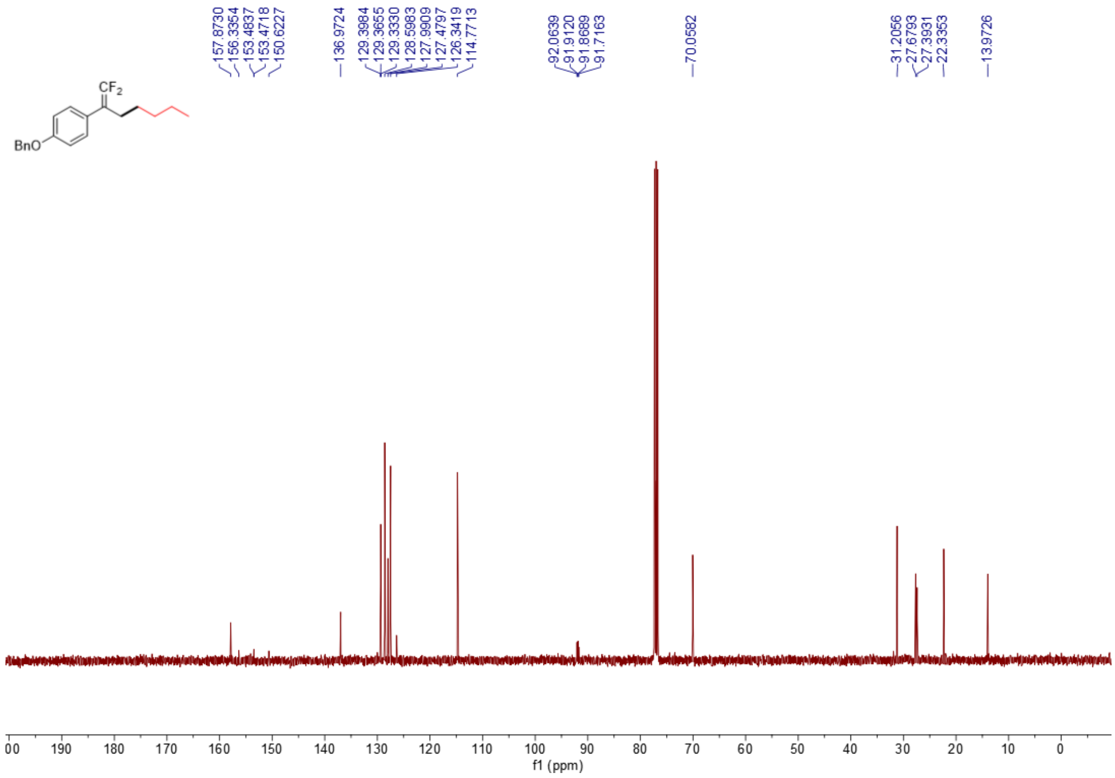
CF2
BnO
157.8730
156.3354
153.4837
153.4718
150.6227
136.9724
129.3984
129.3655
129.3330
128.5983
127.9909
127.4797
126.3419
114.7713
92.0639
91.9120
91.8689
91.7163
70.0582
31.2056
27.6793
27.3931
22.3353
13.9726
f1 (ppm)

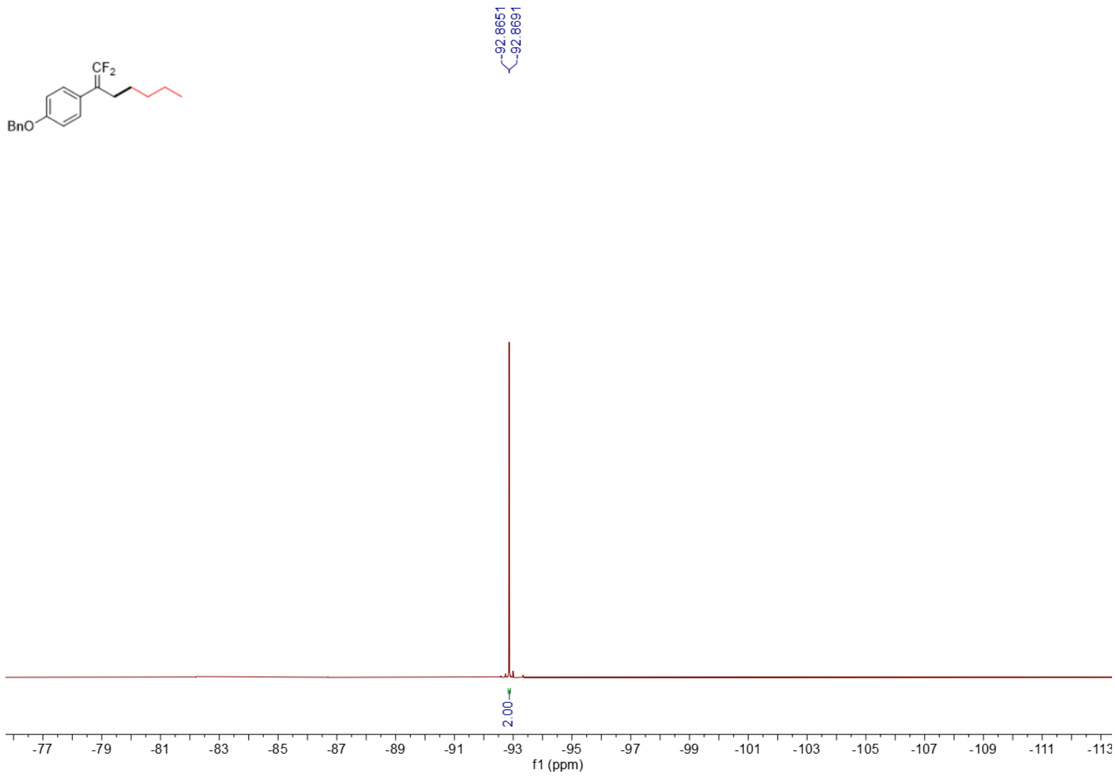
CF2
BnO
-92.8651
-92.8691
2.00
f1 (ppm)

NMR spectrum of 51

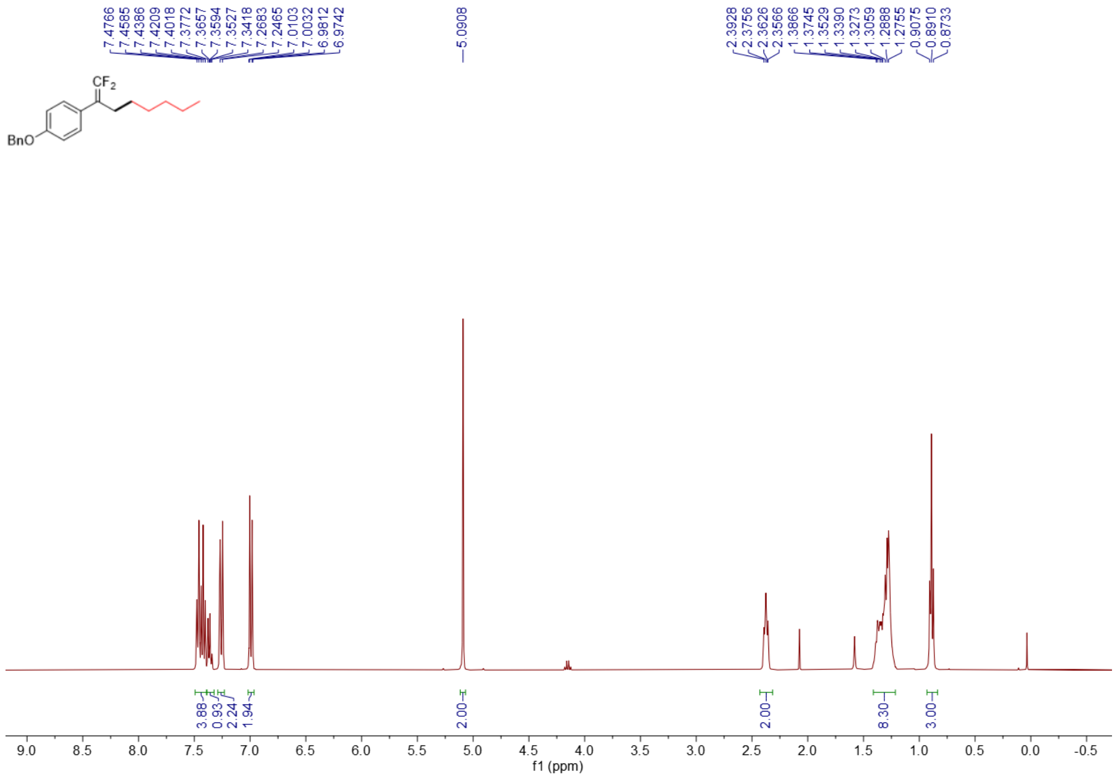

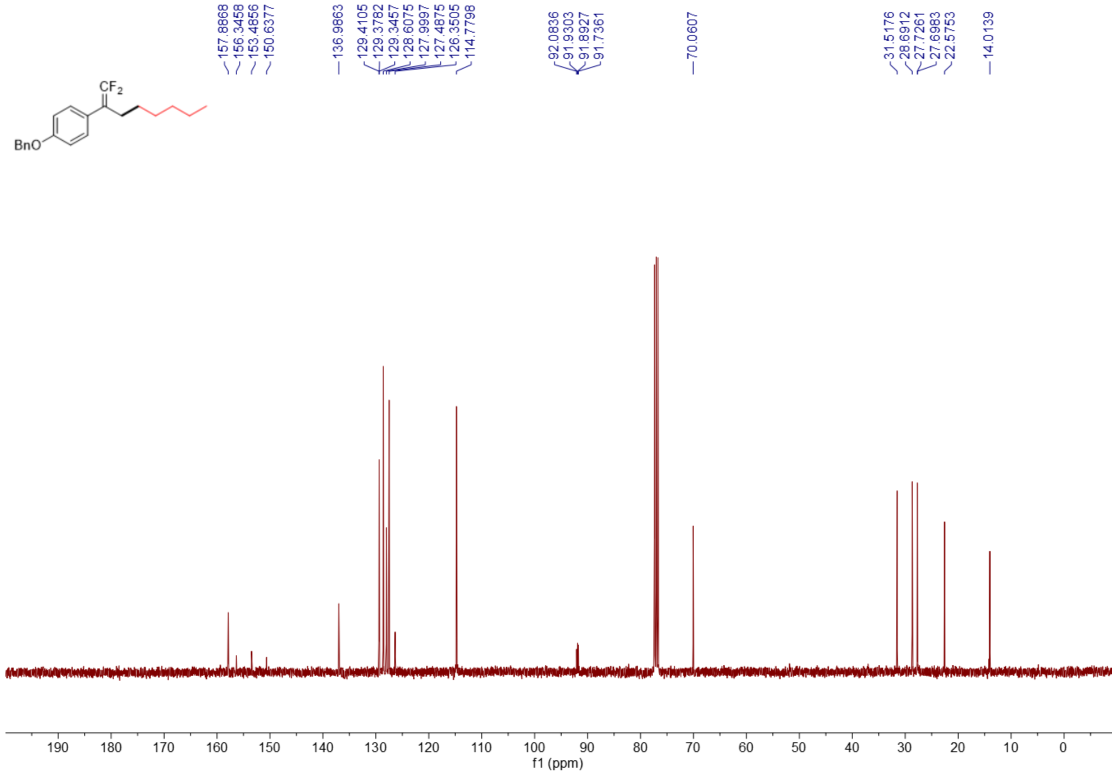

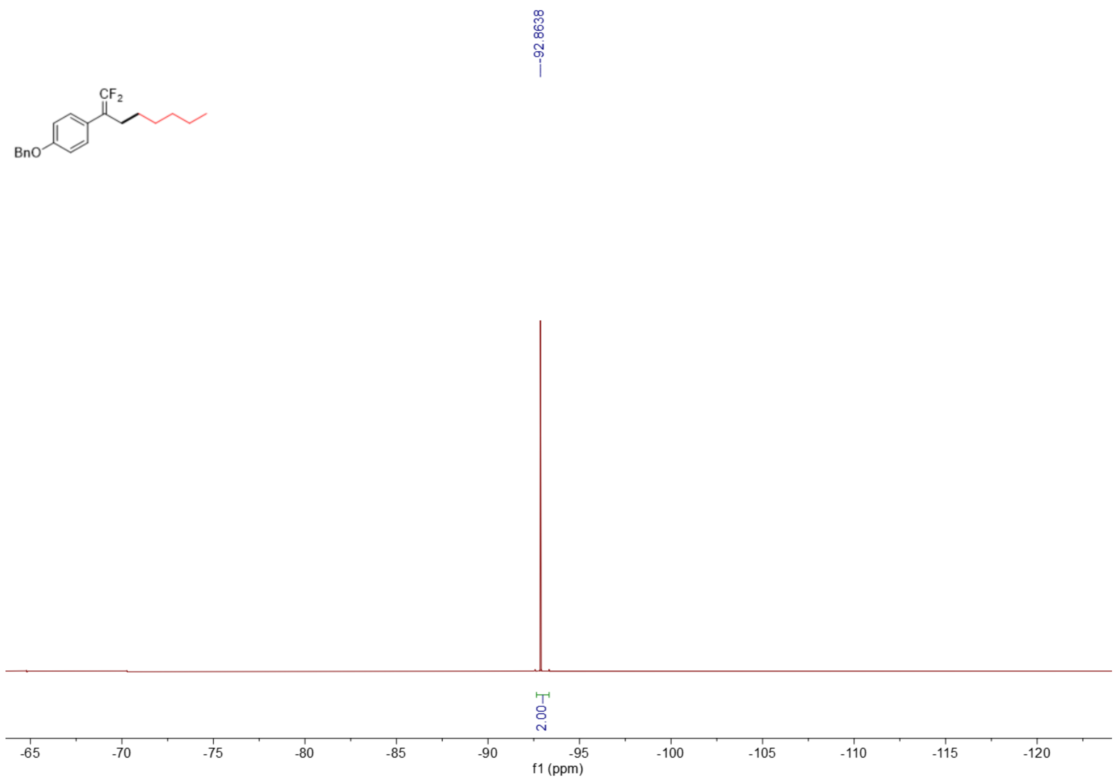

NMR spectrum of 52

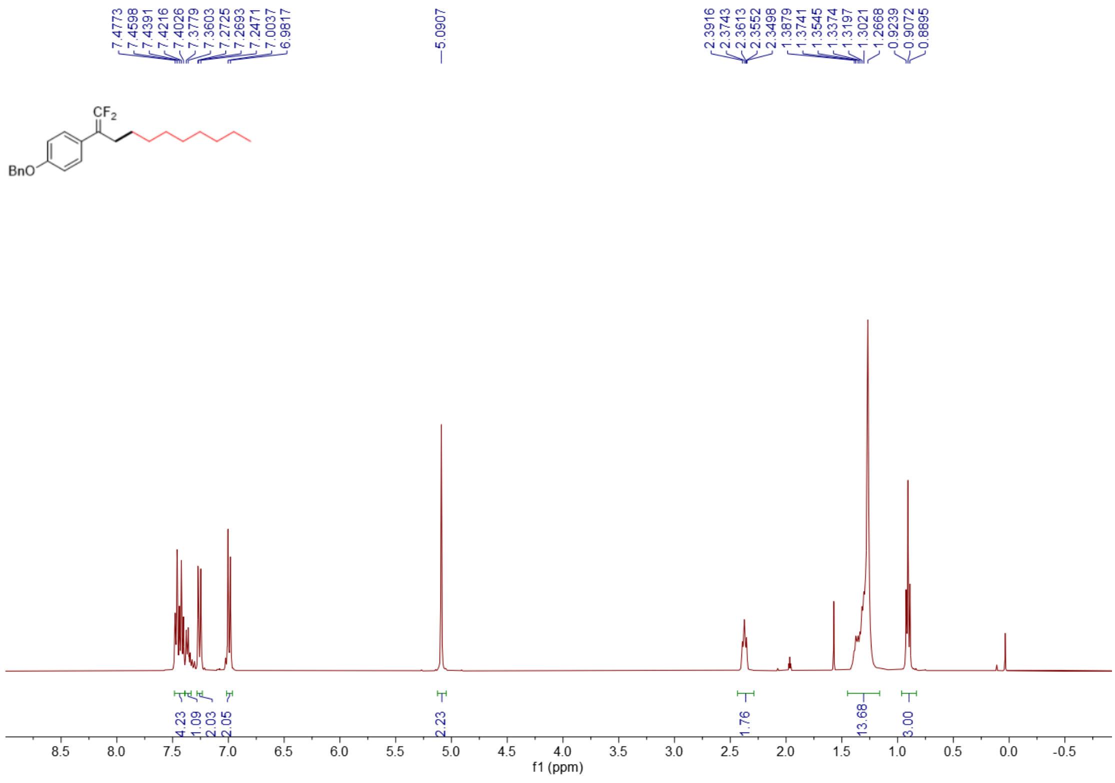

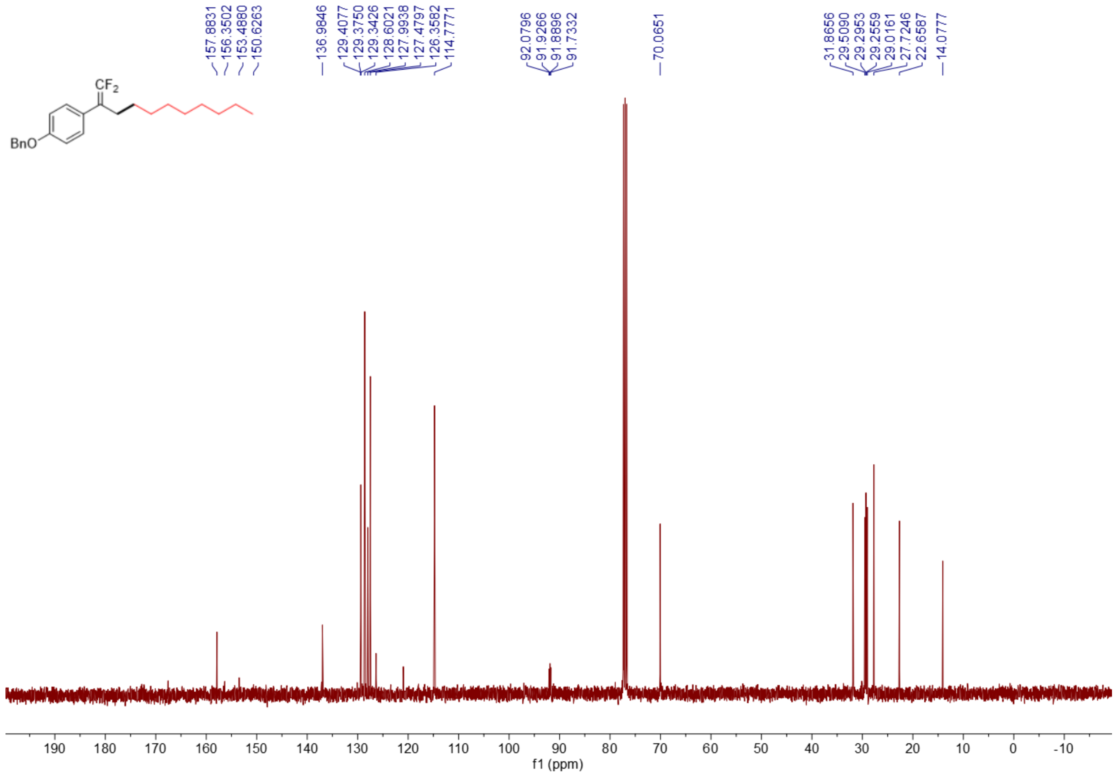
157.8831
156.3502
153.4880
150.6263
136.9846
129.4077
129.3750
129.3426
128.6021
127.9938
127.4797
126.3582
114.7771
92.0796
91.9266
91.8896
91.7332
70.0651
31.8656
29.5090
29.2953
29.2559
29.0161
27.7246
22.6587
14.0777
CF2
BnO
f1 (ppm)

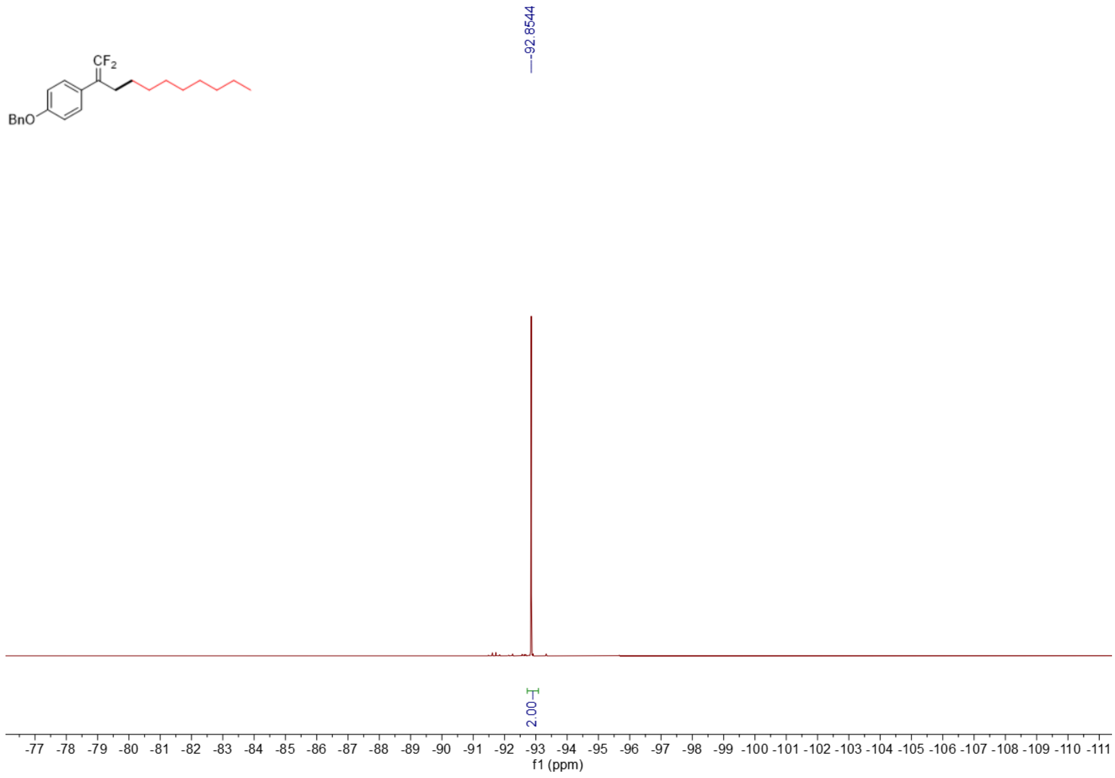
-92.8544
CF2
BnO
2.00
f1 (ppm)

NMR spectrum of 53

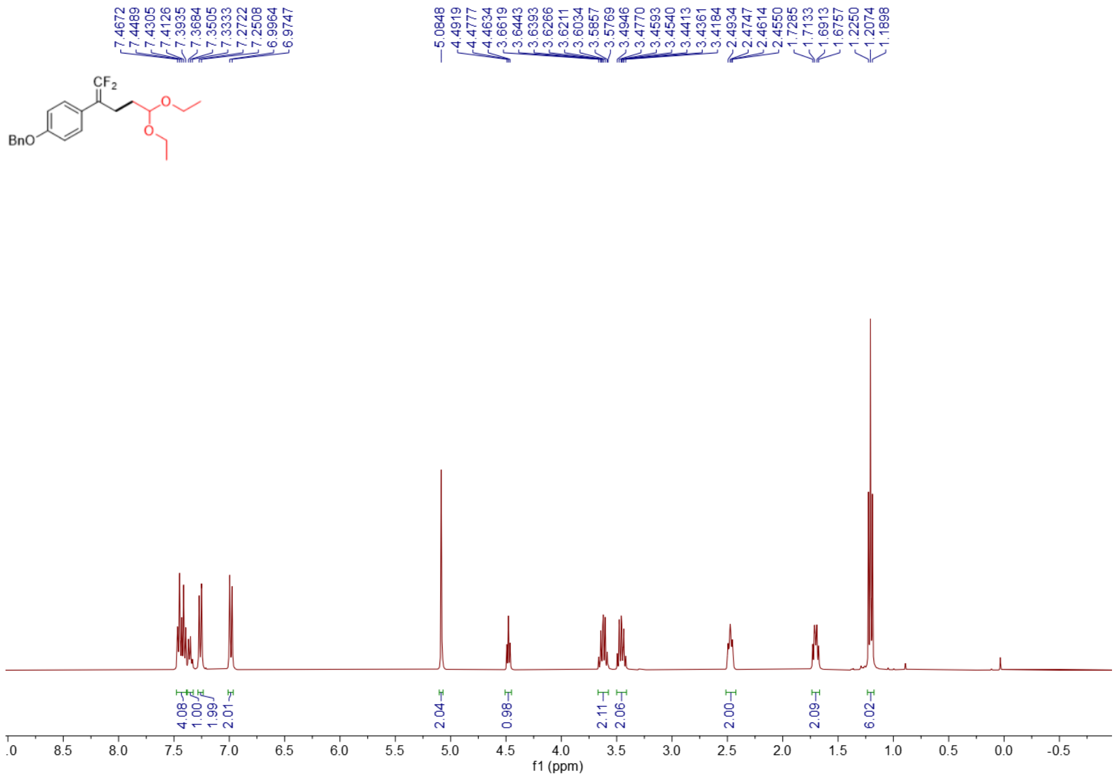

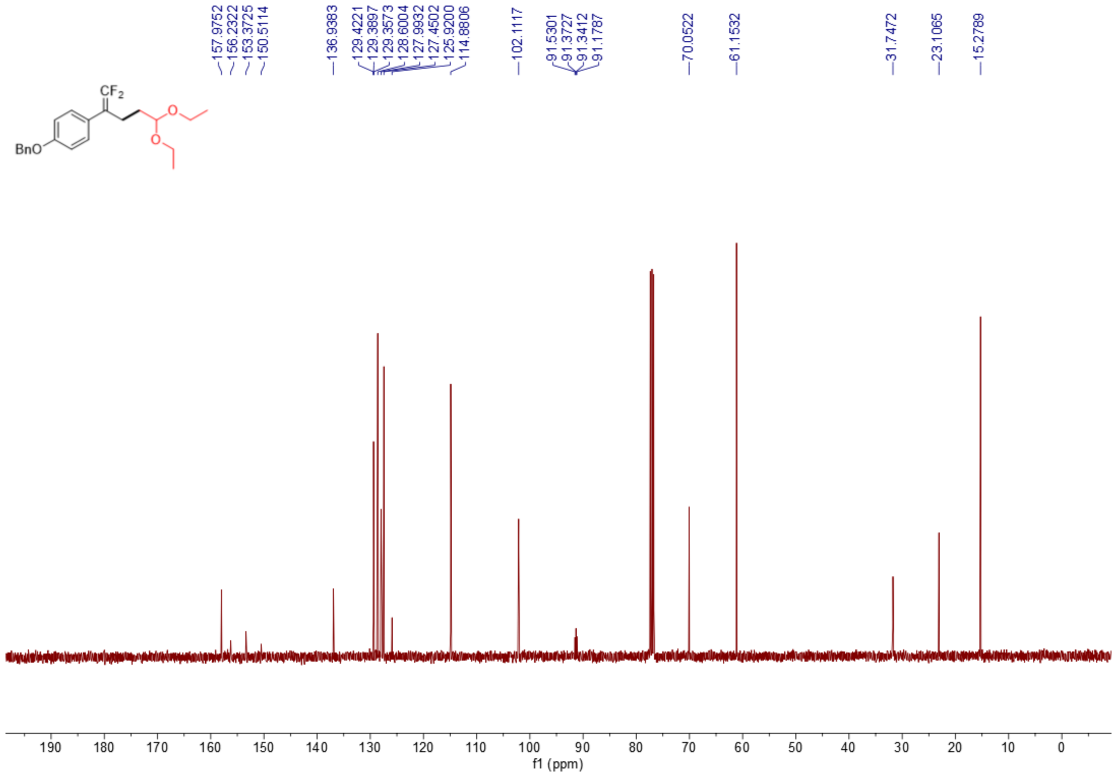

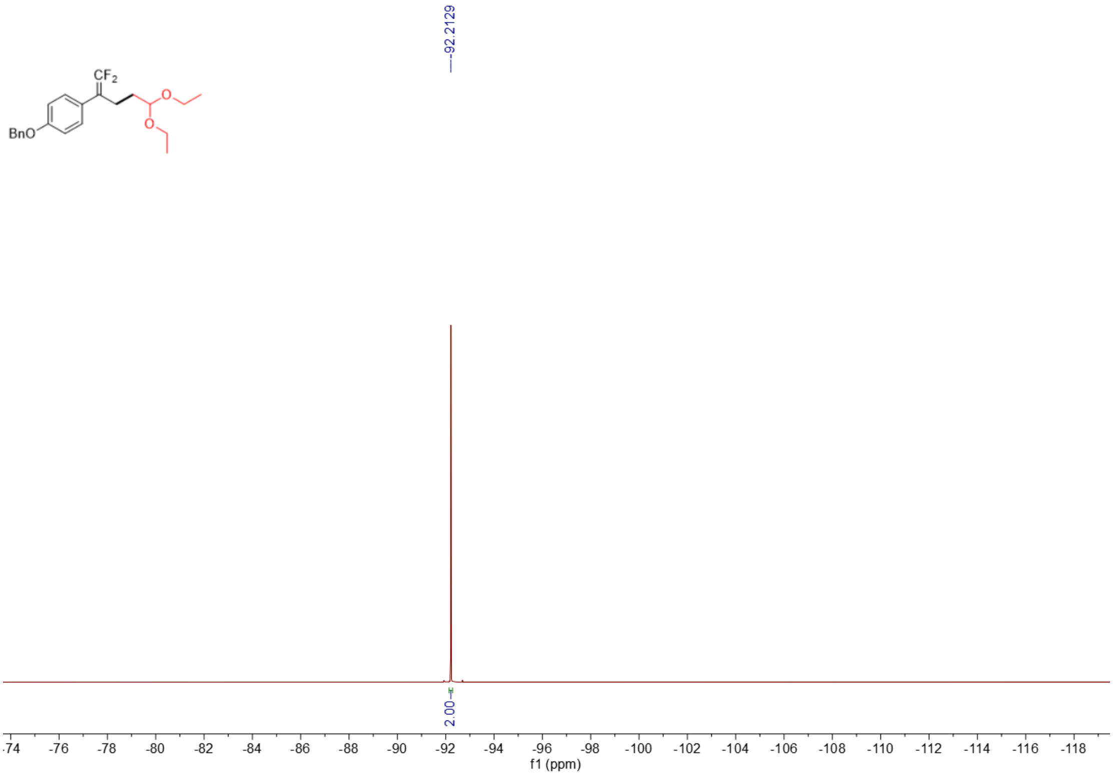

NMR spectrum of 54

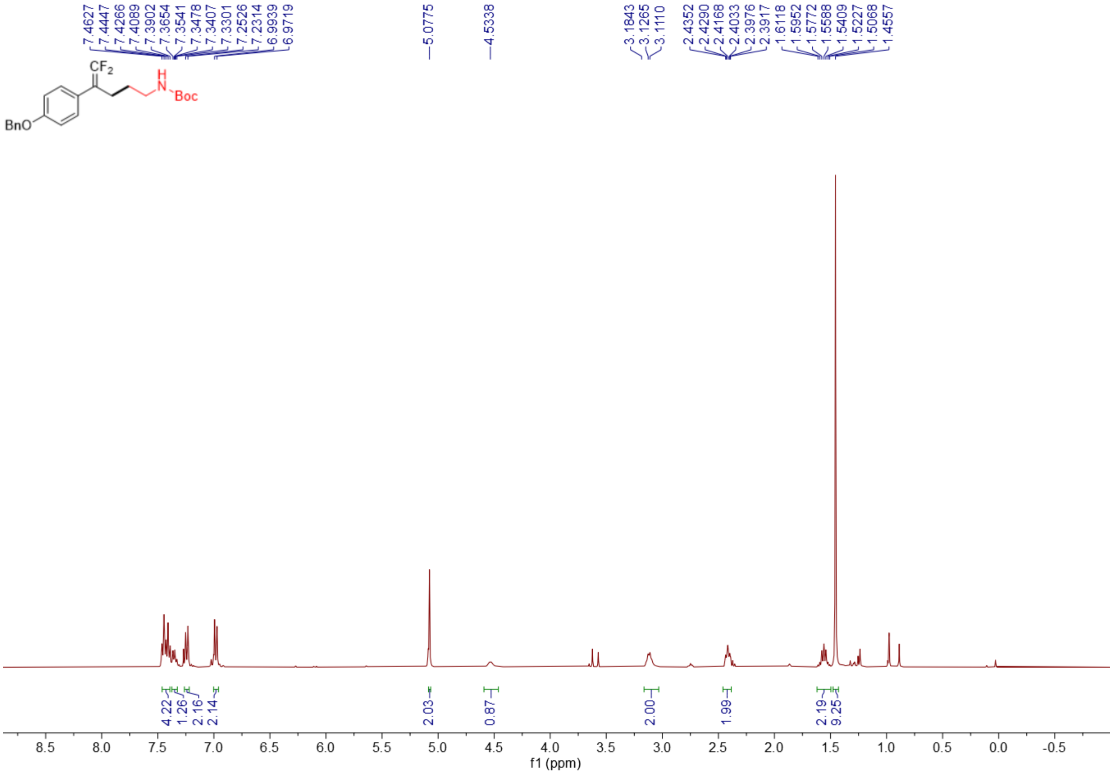

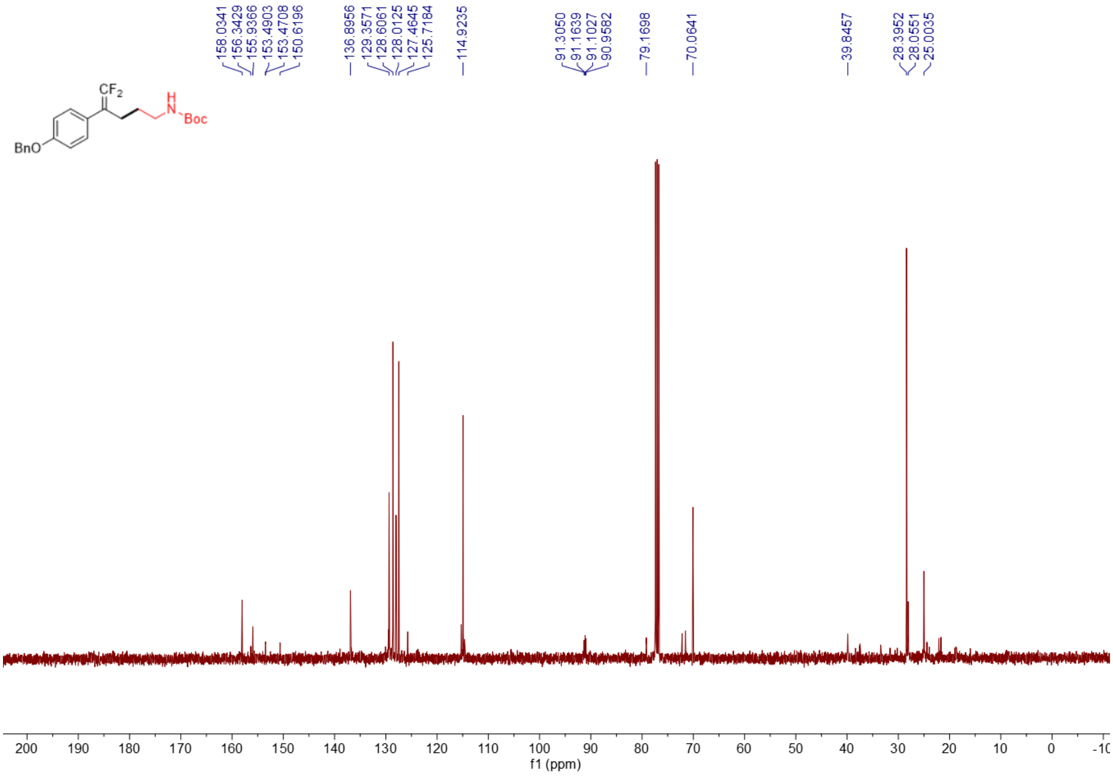
158.0341
156.3429
155.9366
153.4903
153.4708
150.6196
136.8956
129.3571
128.6061
128.0125
127.4645
125.7184
114.9235
91.3050
91.1639
91.1027
90.9582
79.1698
70.0641
39.8457
28.3952
28.0551
25.0035
CF2
H
N
Boc
BnO
f1 (ppm)

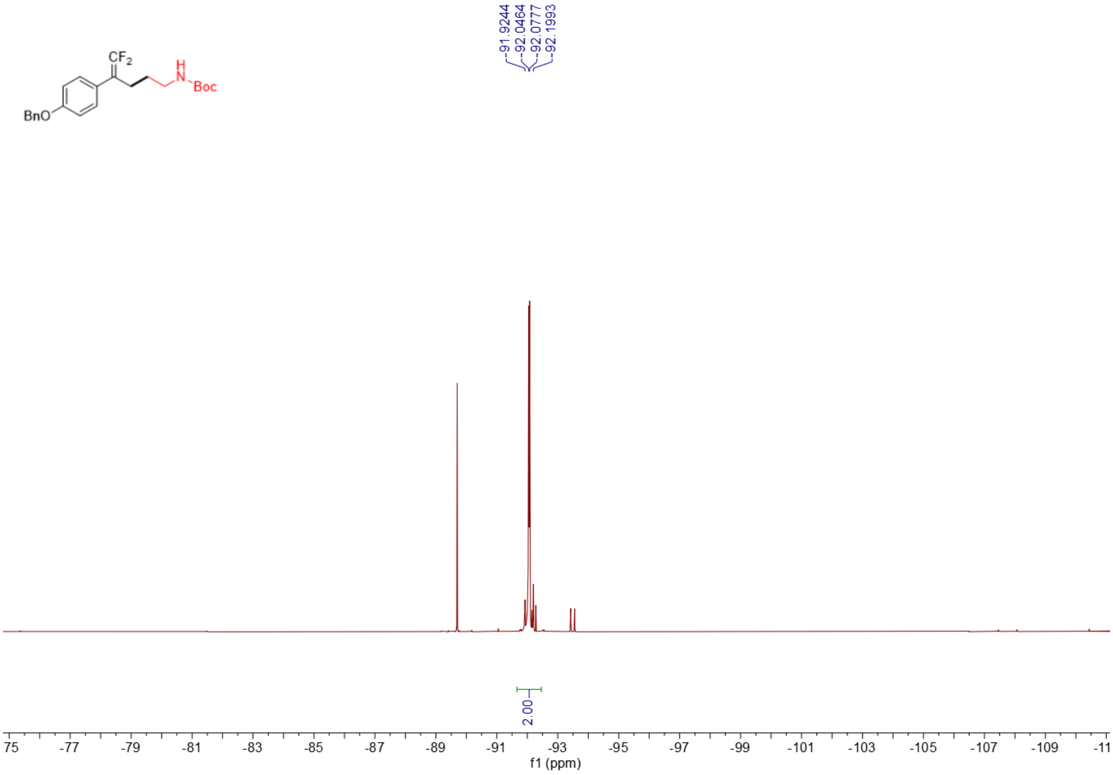
-91.9244
-92.0464
-92.0777
-92.1993
CF2
H
N
Boc
BnO
2.00
f1 (ppm)

NMR spectrum of 55

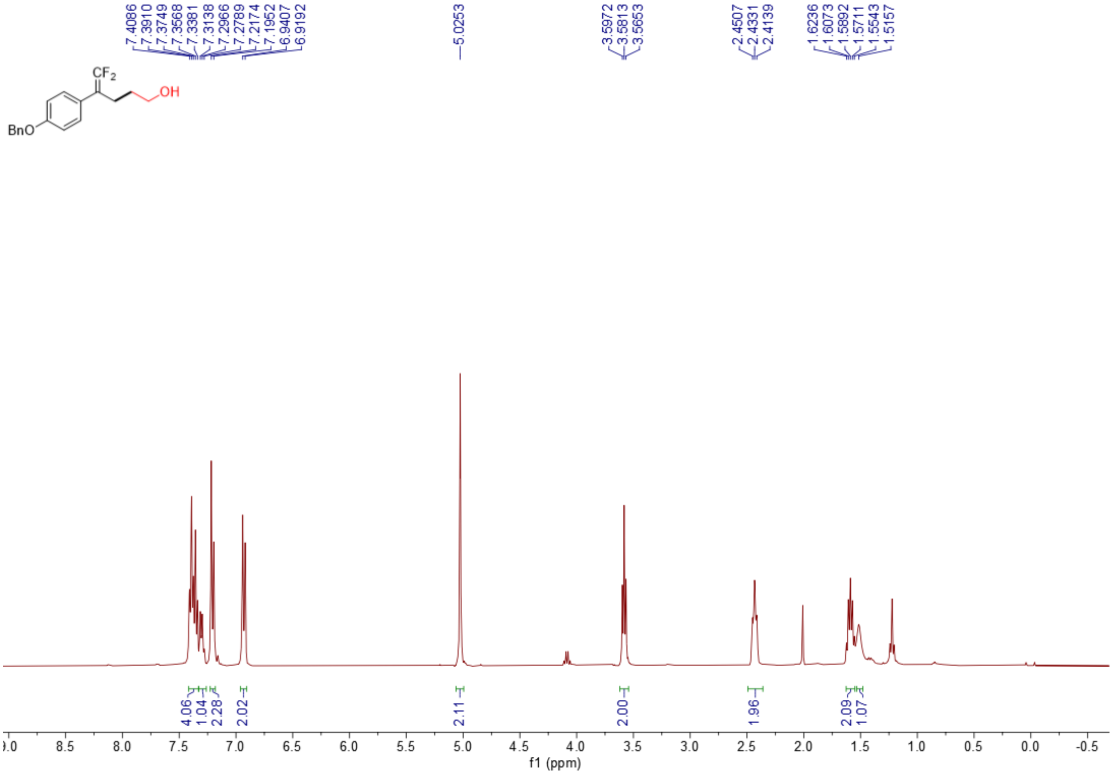

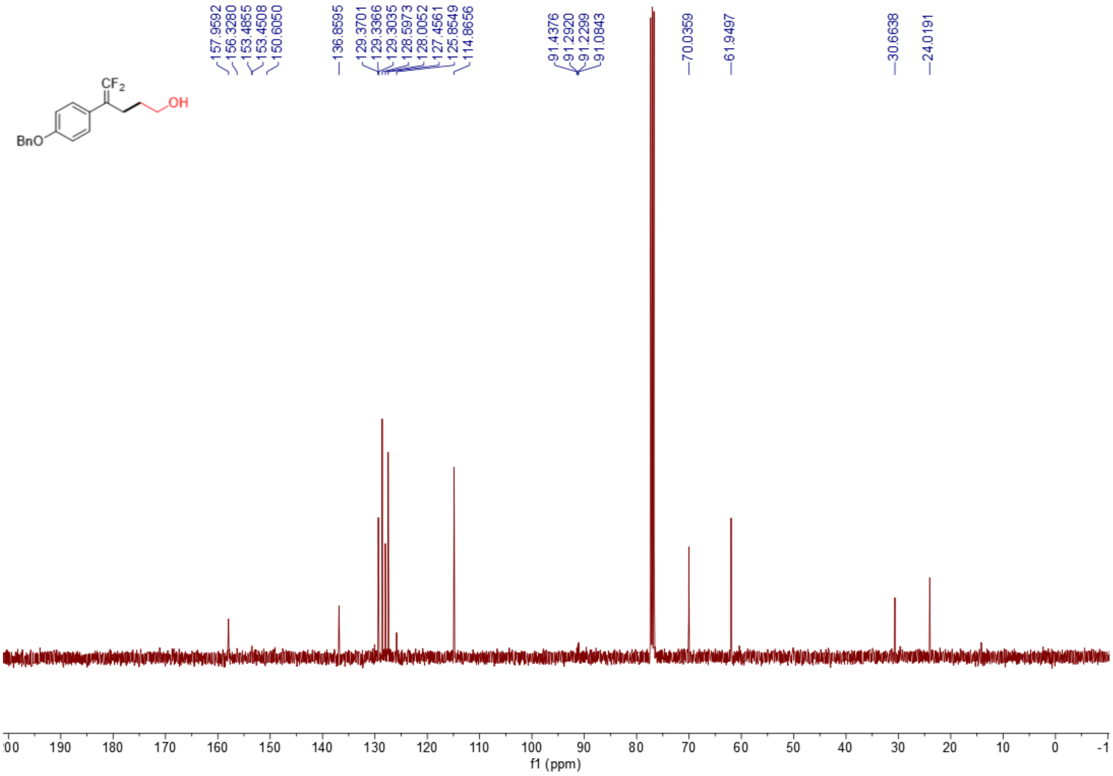

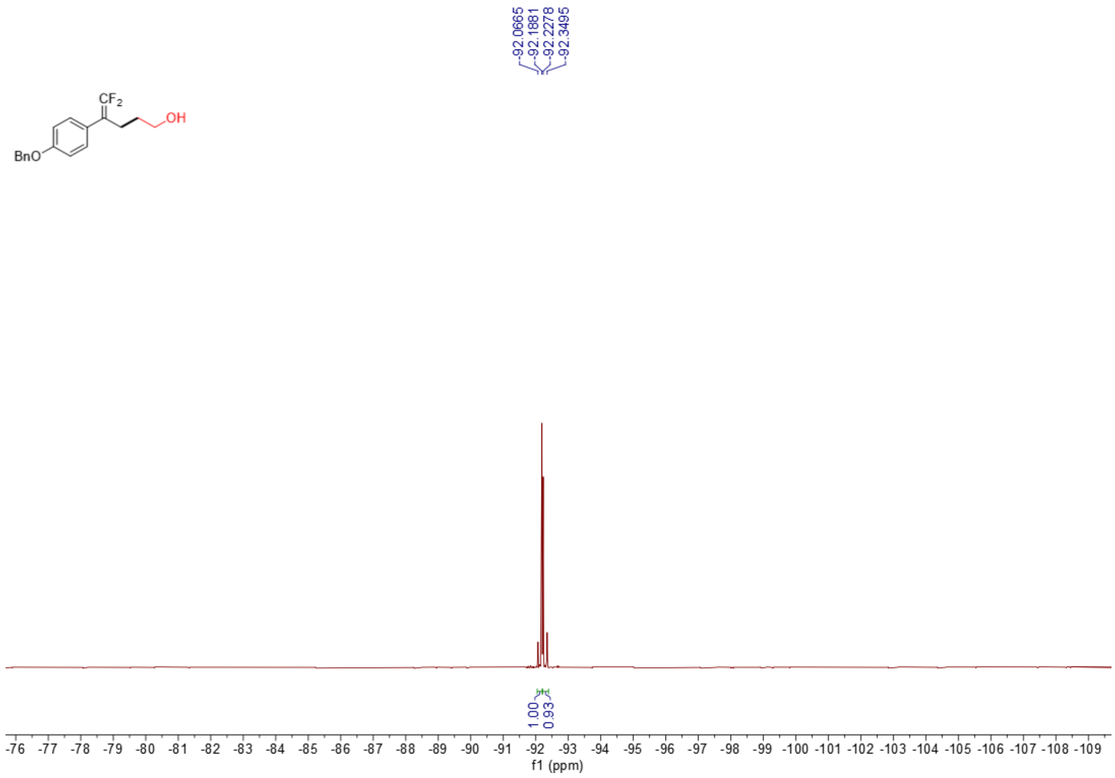

NMR spectrum of 56

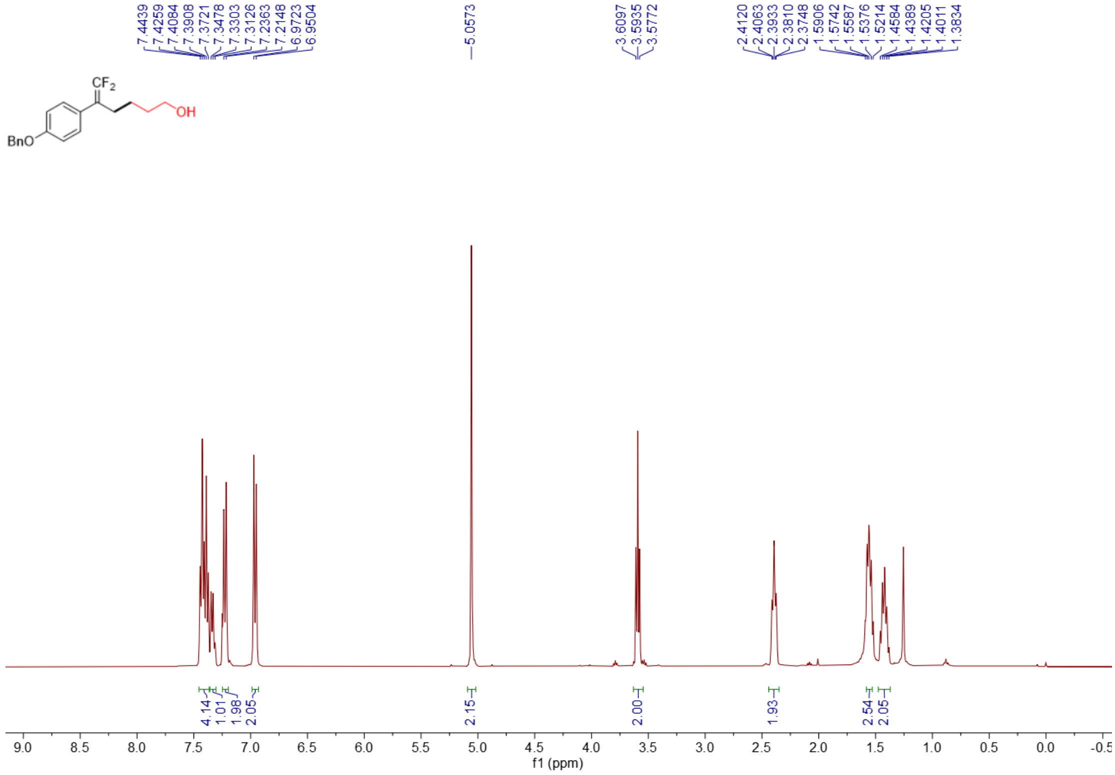

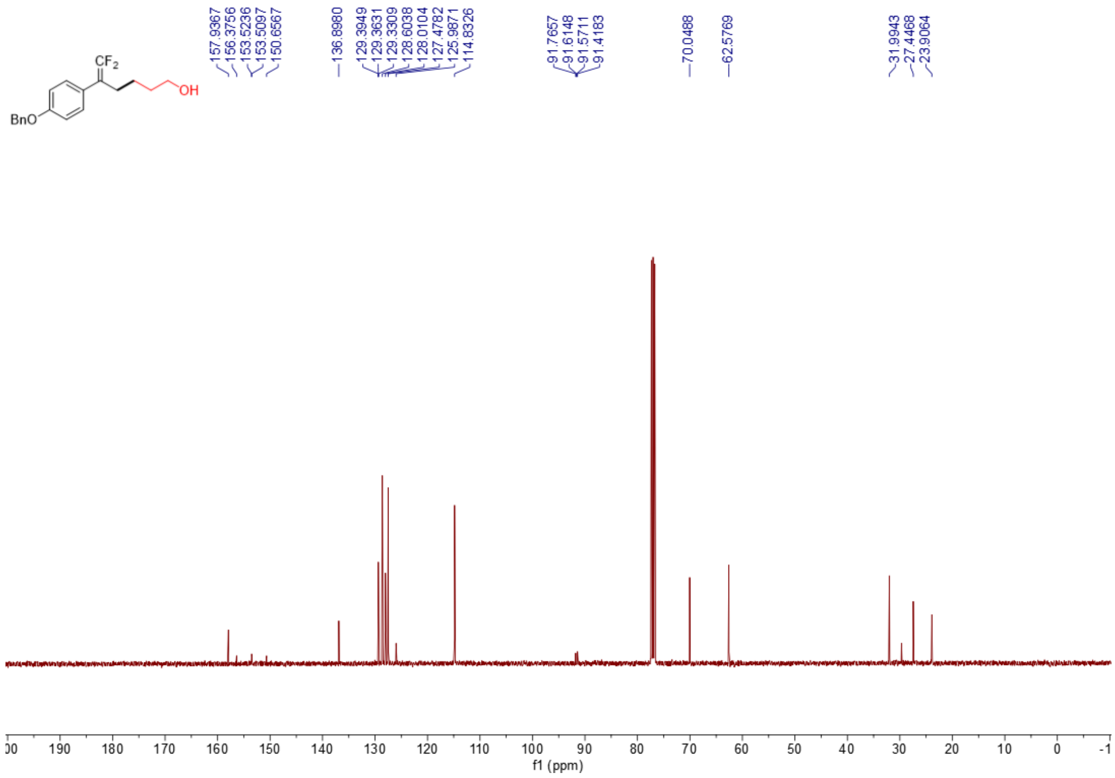
CF2
OH
BnO
157.9367
156.3756
153.5236
153.5097
150.6567
136.8980
129.3949
129.3631
129.3309
128.6038
128.0104
127.4782
125.9871
114.8326
91.7657
91.6148
91.5711
91.4183
70.0488
62.5769
31.9943
27.4468
23.9064
f1 (ppm)

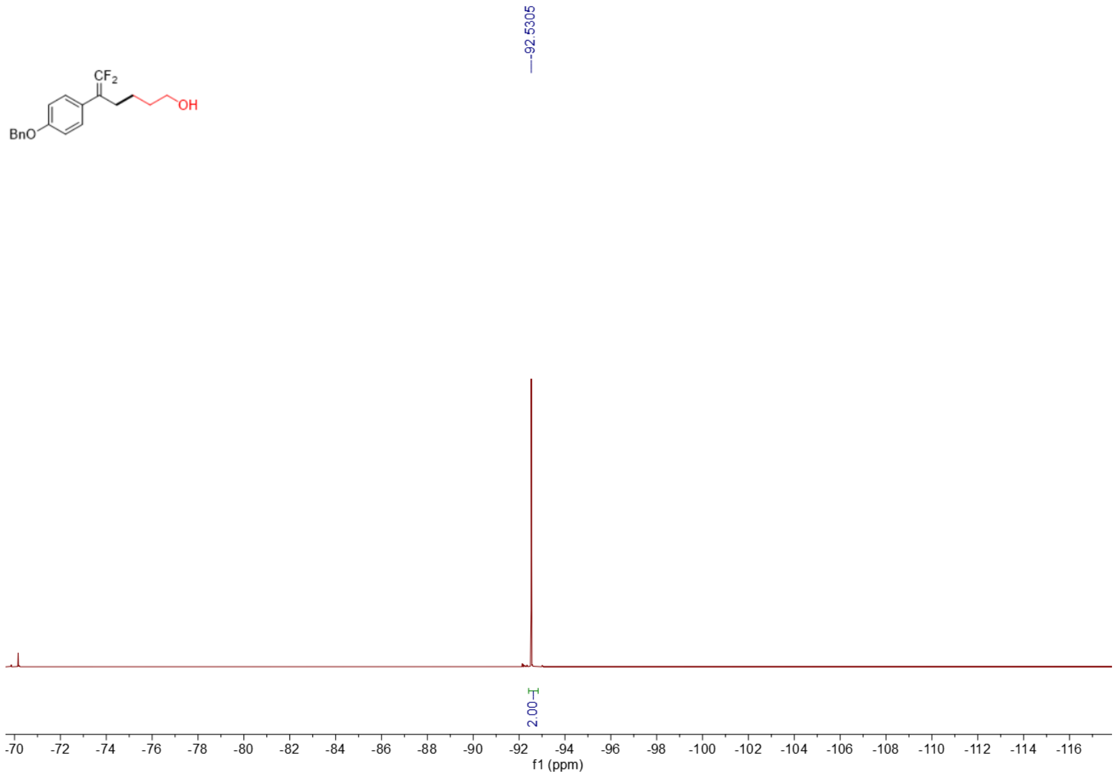
CF2
OH
BnO
-92.5305
2.00
f1 (ppm)

NMR spectrum of 57

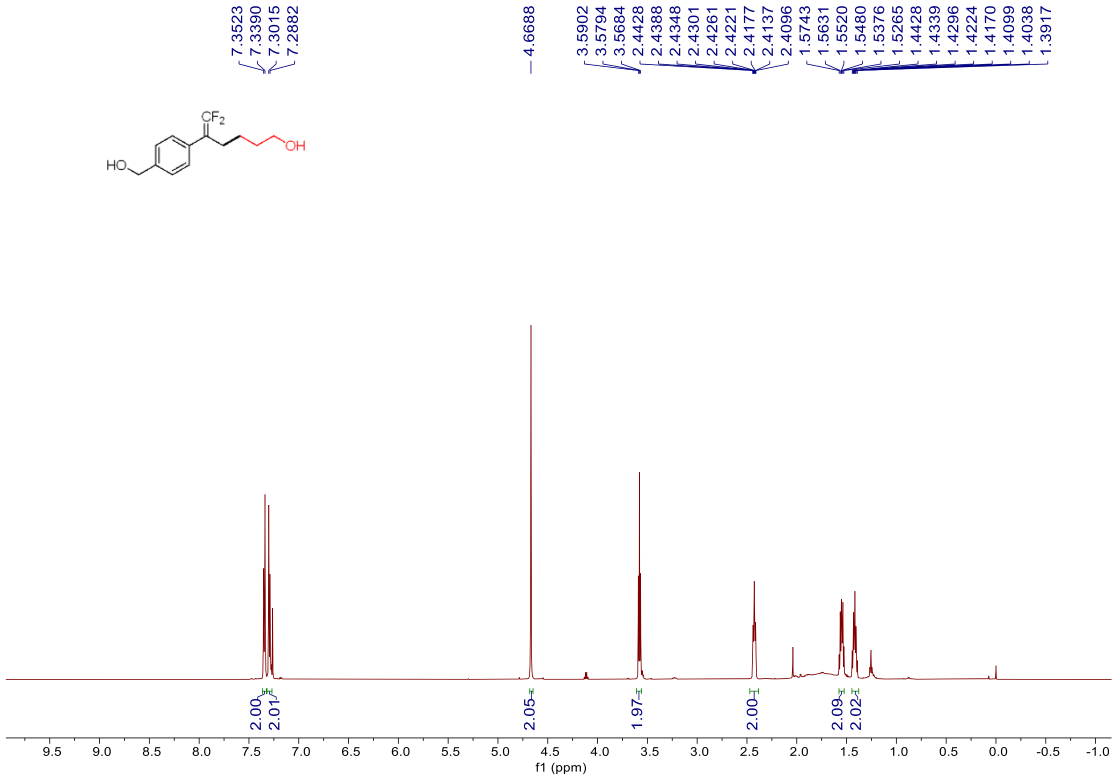

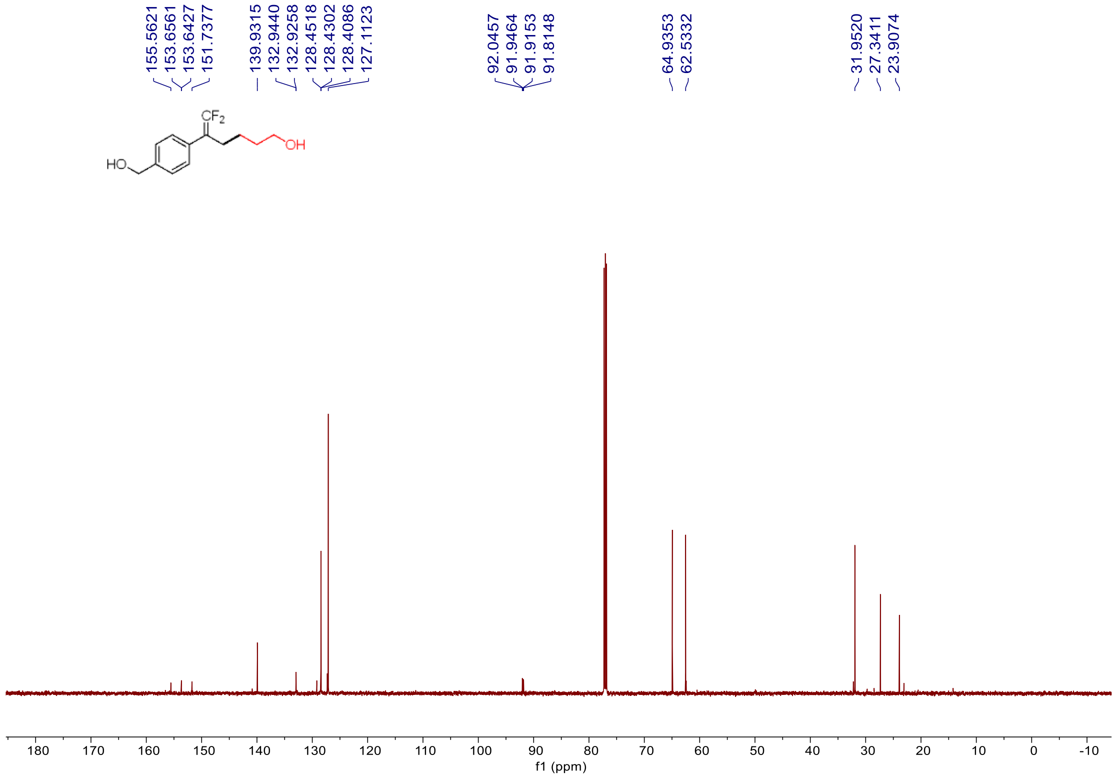

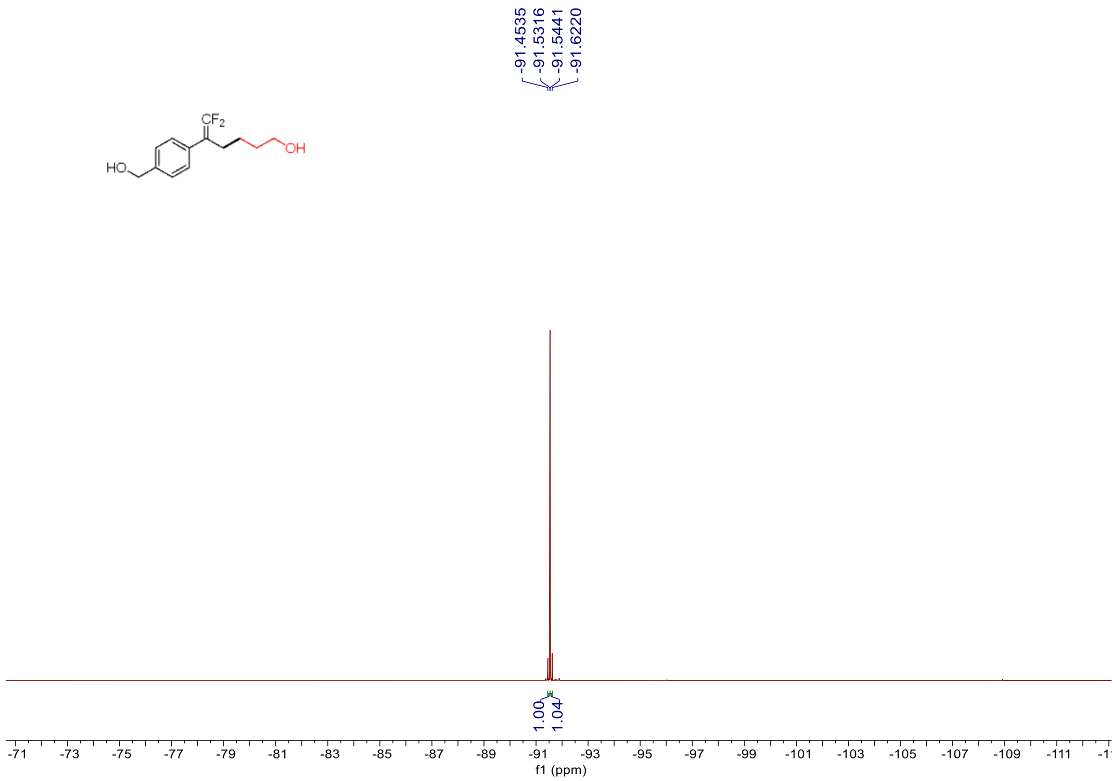

NMR spectrum of 58

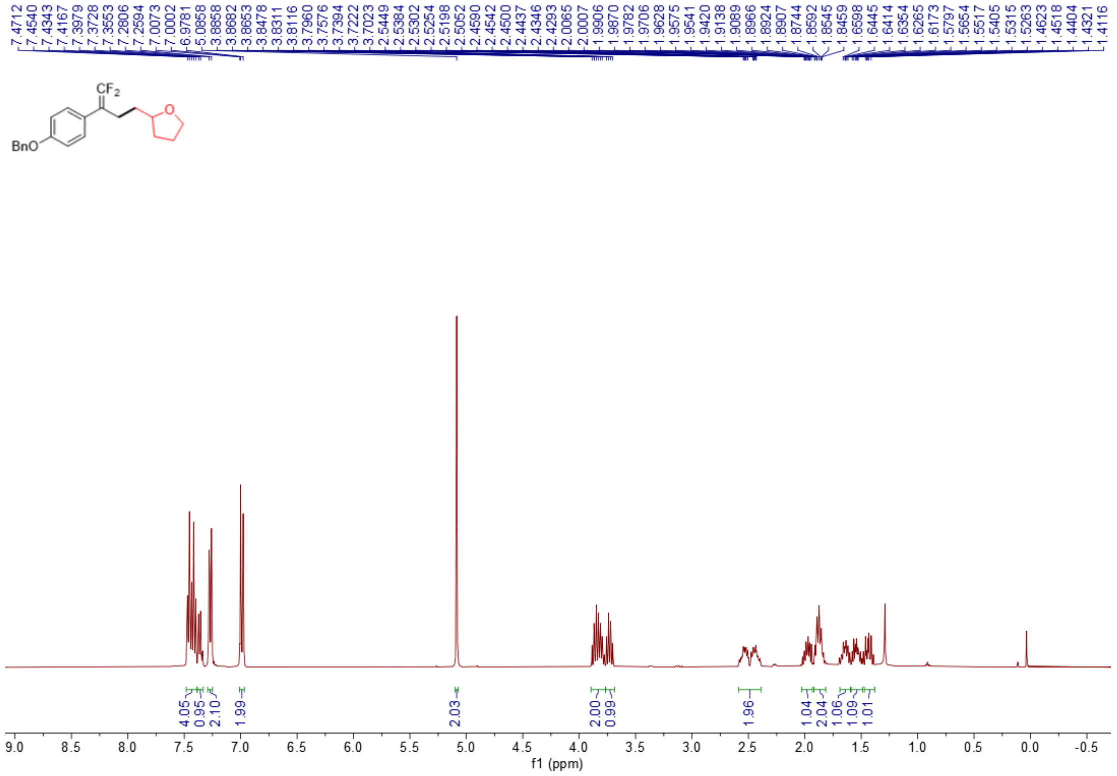

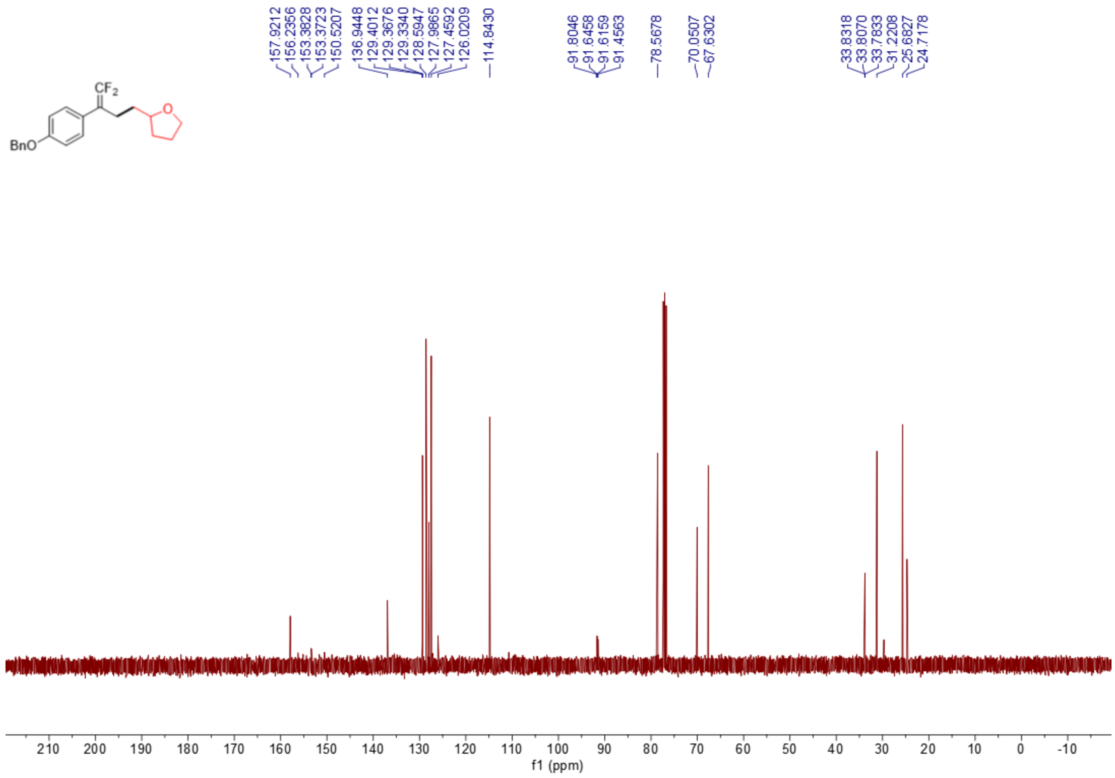
157.9212
156.2356
153.3828
153.3723
150.5207
136.9448
129.4012
129.3676
129.3340
128.5947
127.9865
127.4592
126.0209
114.8430
91.8046
91.6458
91.6159
91.4563
78.5678
70.0507
67.6302
33.8318
33.8070
33.7833
31.2208
25.6827
24.7178
CF2
BnO
O
210 200 190 180 170 160 150 140 130 120 110 100 90 80 70 60 50 40 30 20 10 0 -10
f1 (ppm)

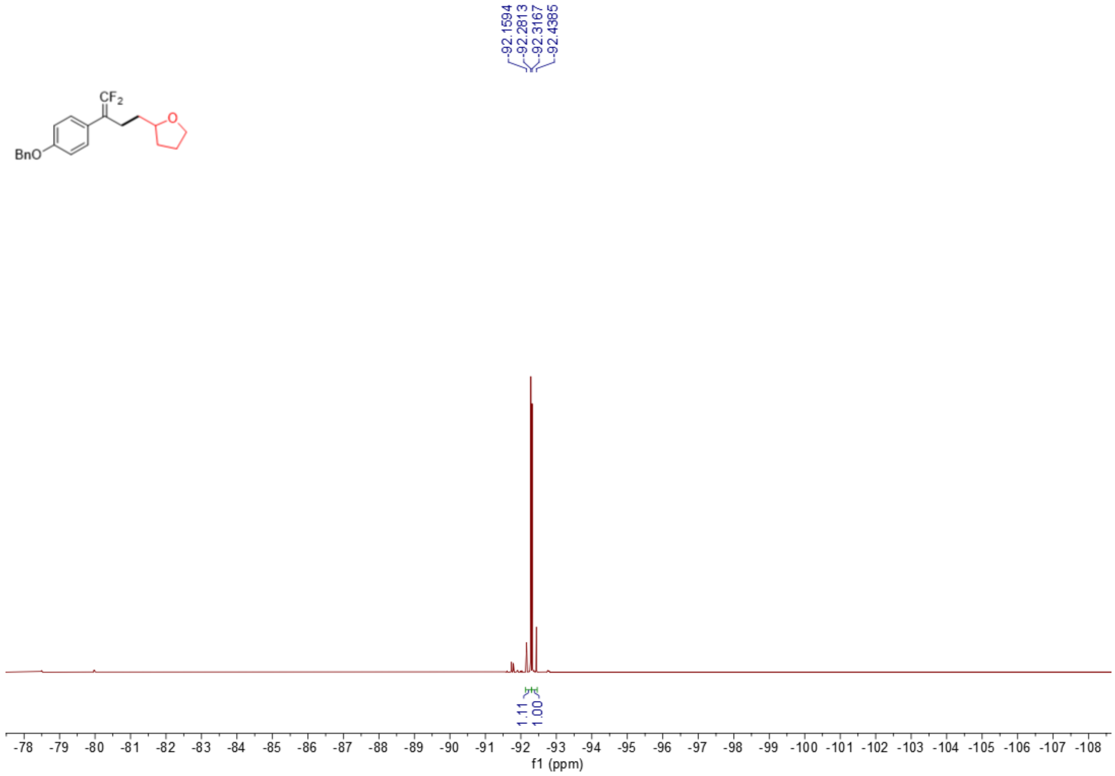
-92.1594
-92.2813
-92.3167
-92.4385
CF2
BnO
O
1.11
1.00
-78 -79 -80 -81 -82 -83 -84 -85 -86 -87 -88 -89 -90 -91 -92 -93 -94 -95 -96 -97 -98 -99 -100 -101 -102 -103 -104 -105 -106 -107 -108
f1 (ppm)

NMR spectrum of 59

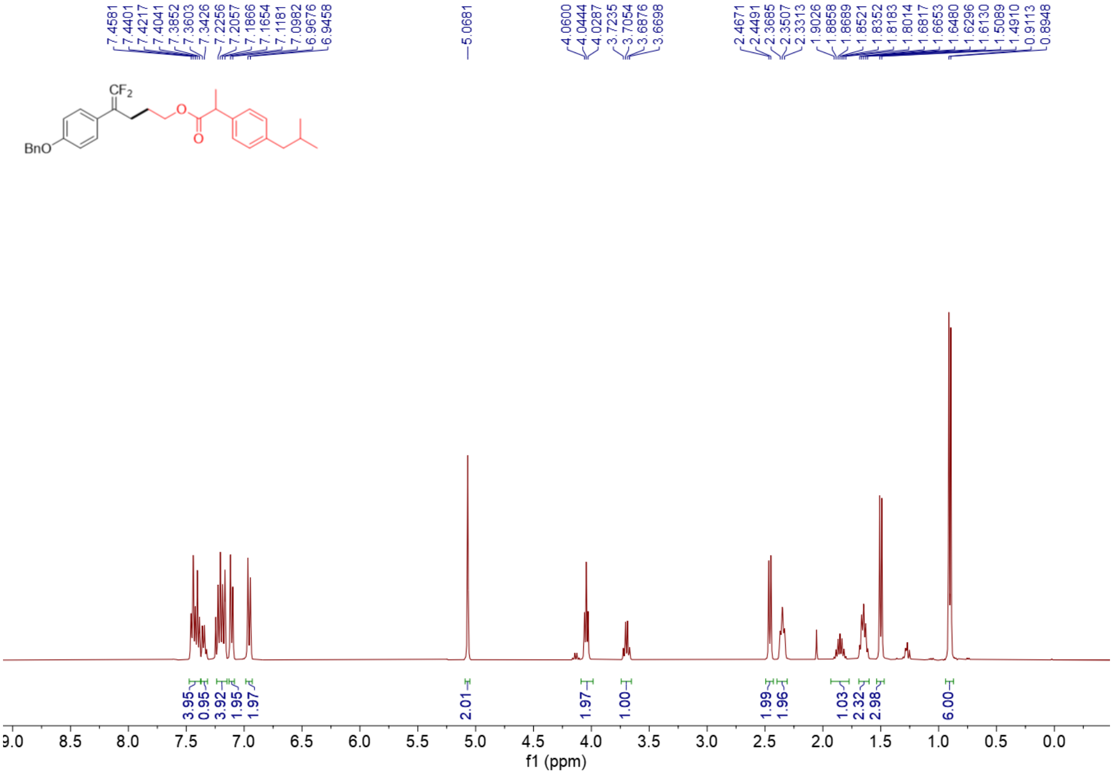

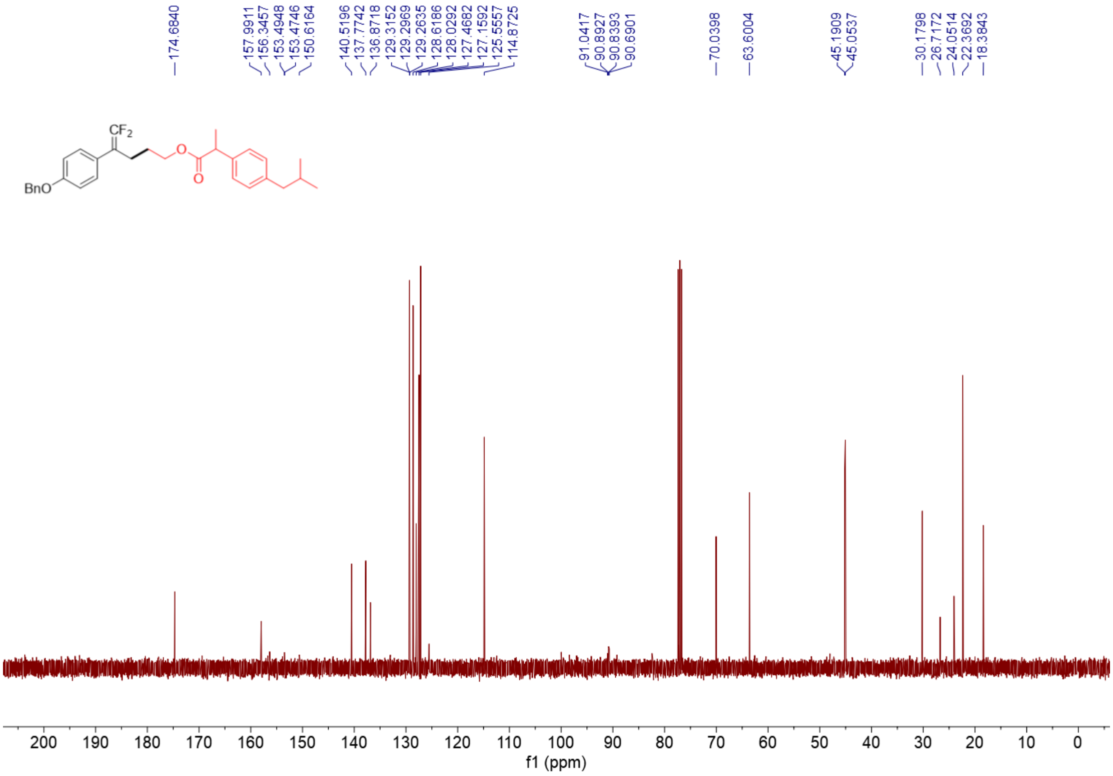

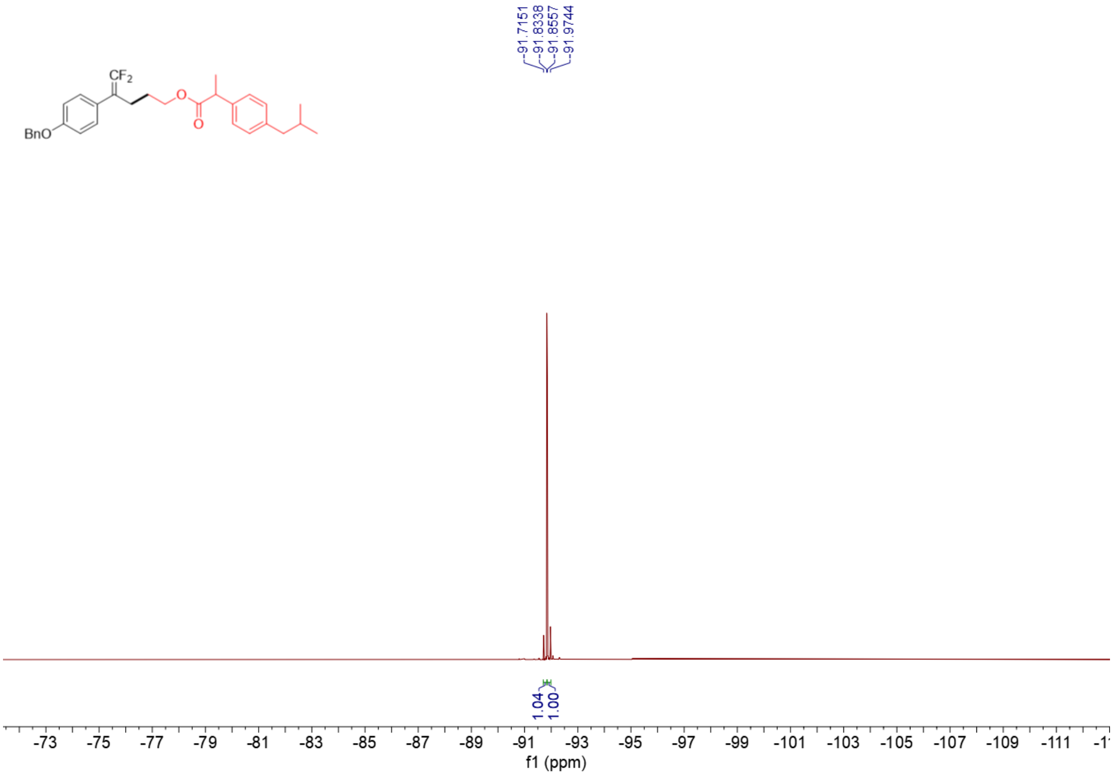

NMR spectrum of 60

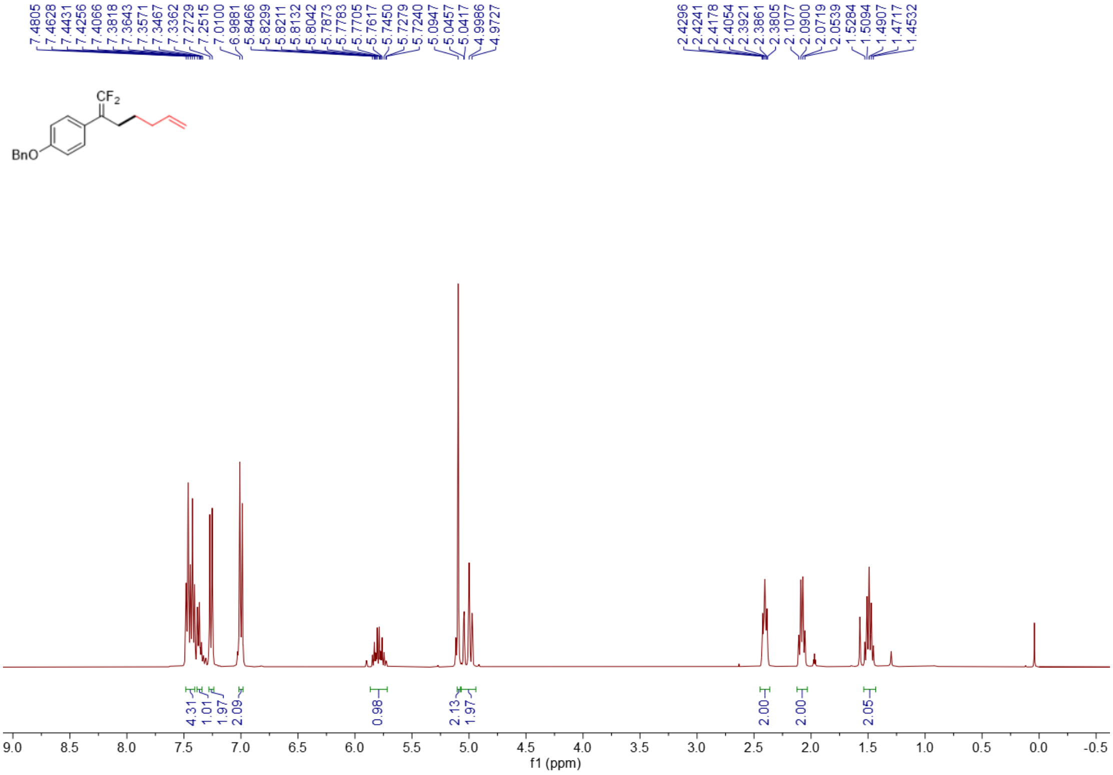

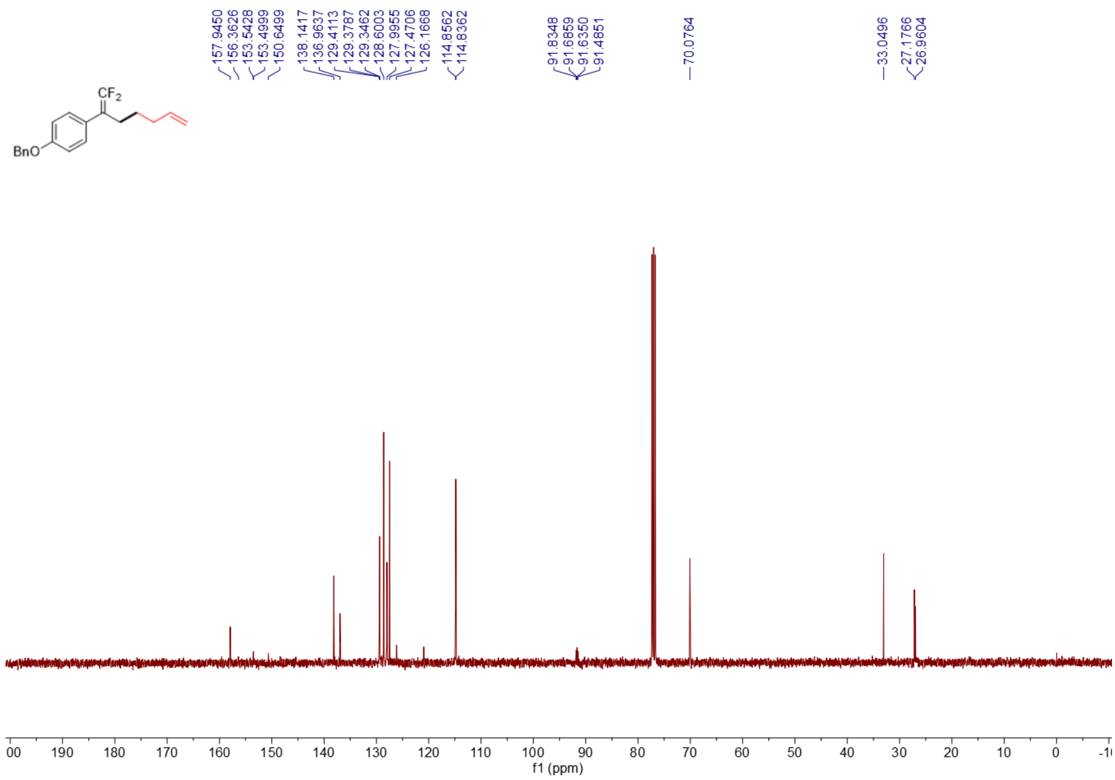

CF2
BnO
157.9450
156.3626
153.5428
153.4999
150.6499
138.1417
136.9637
129.4113
129.3787
129.3462
128.6003
127.9955
127.4706
126.1668
114.8562
114.8362
91.8348
91.6859
91.6350
91.4851
70.0764
33.0496
27.1766
26.9604
f1 (ppm)

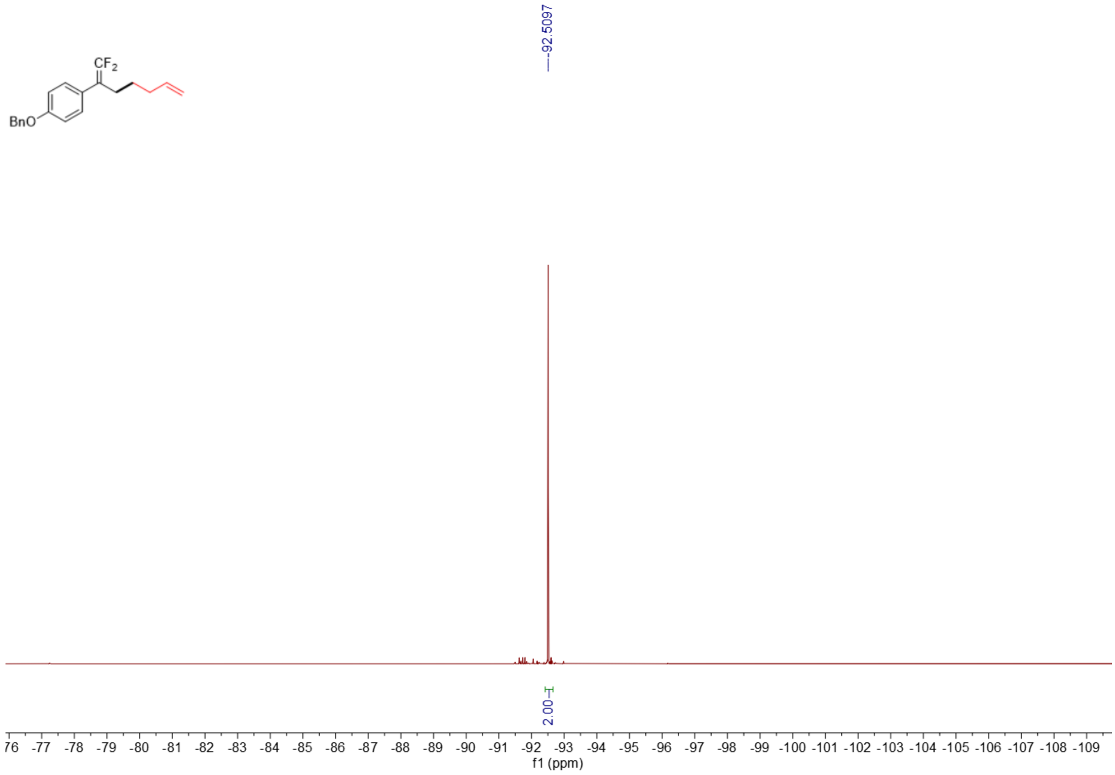

CF2
BnO
-92.5097
2.00
f1 (ppm)

NMR spectrum of 61

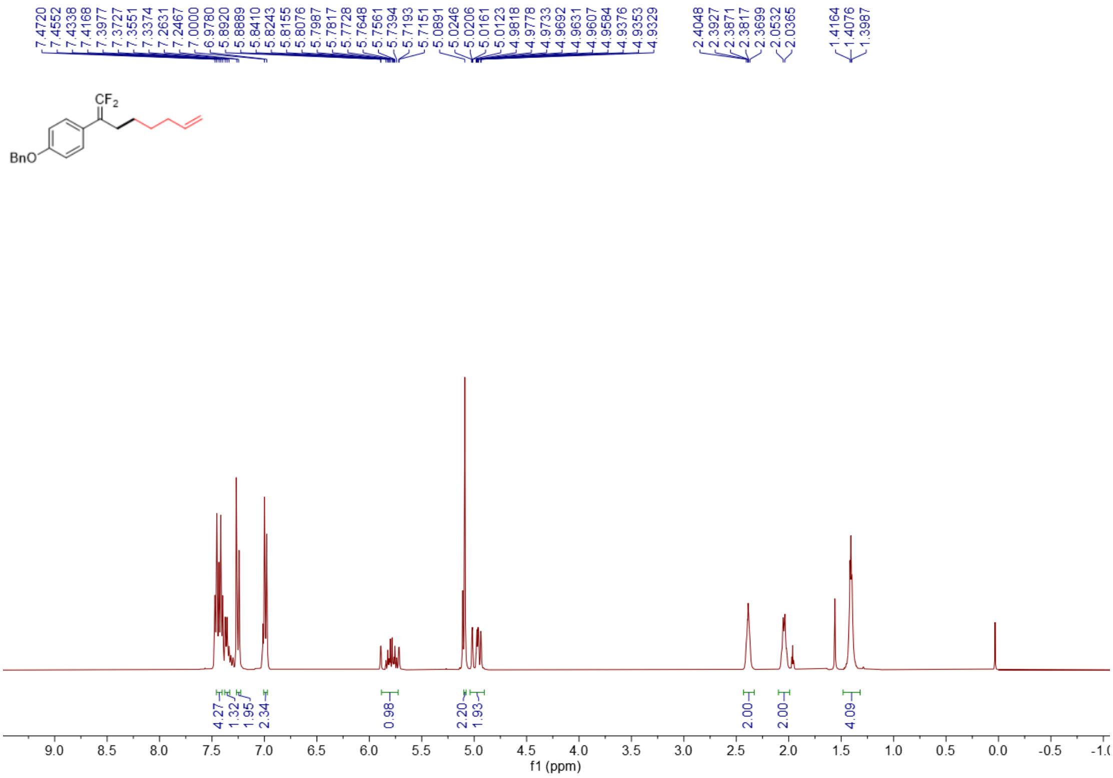

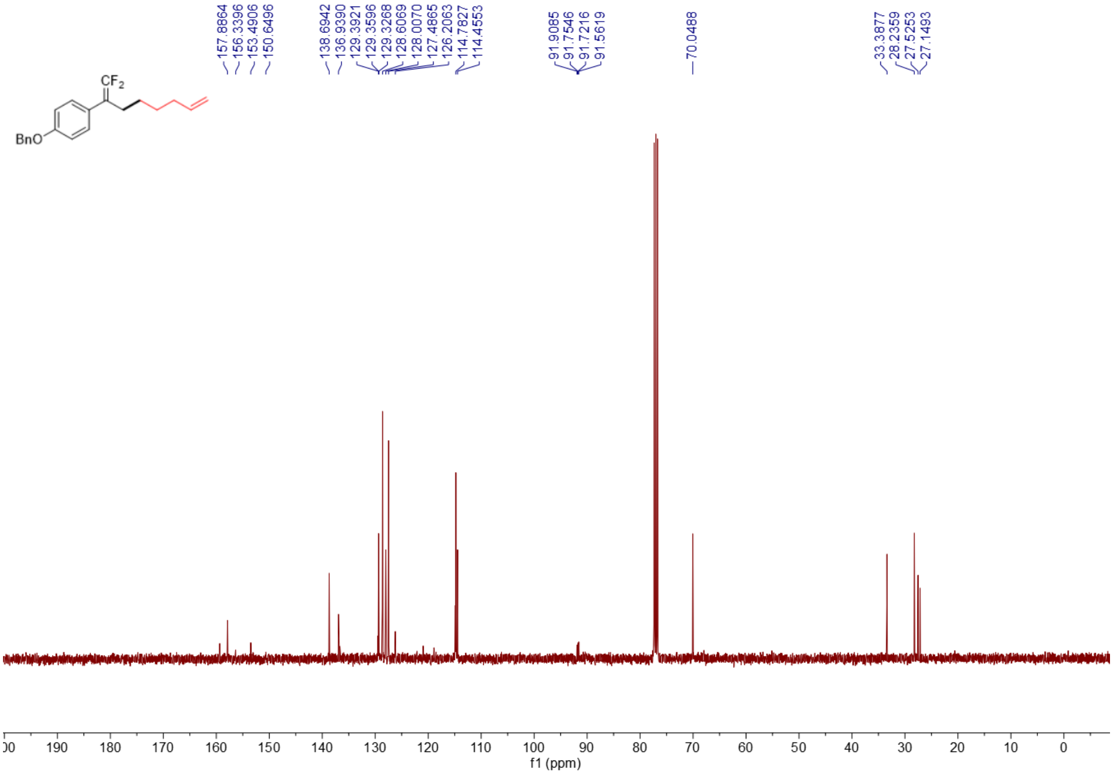

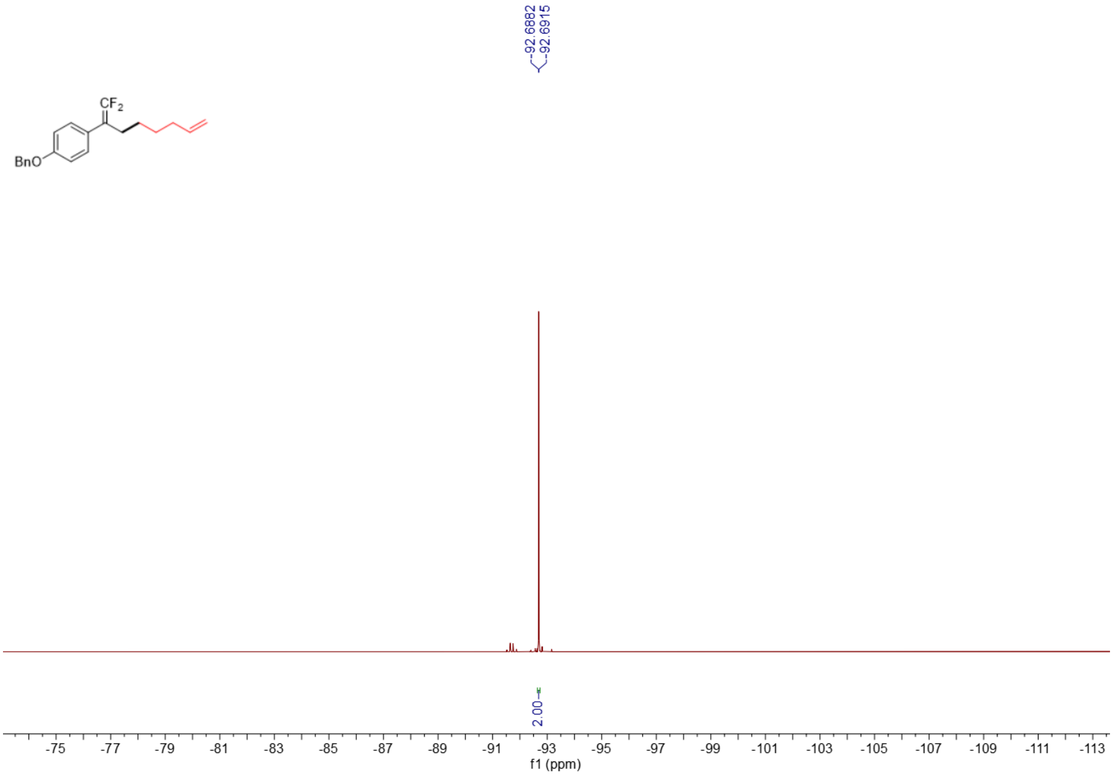

NMR spectrum of 62

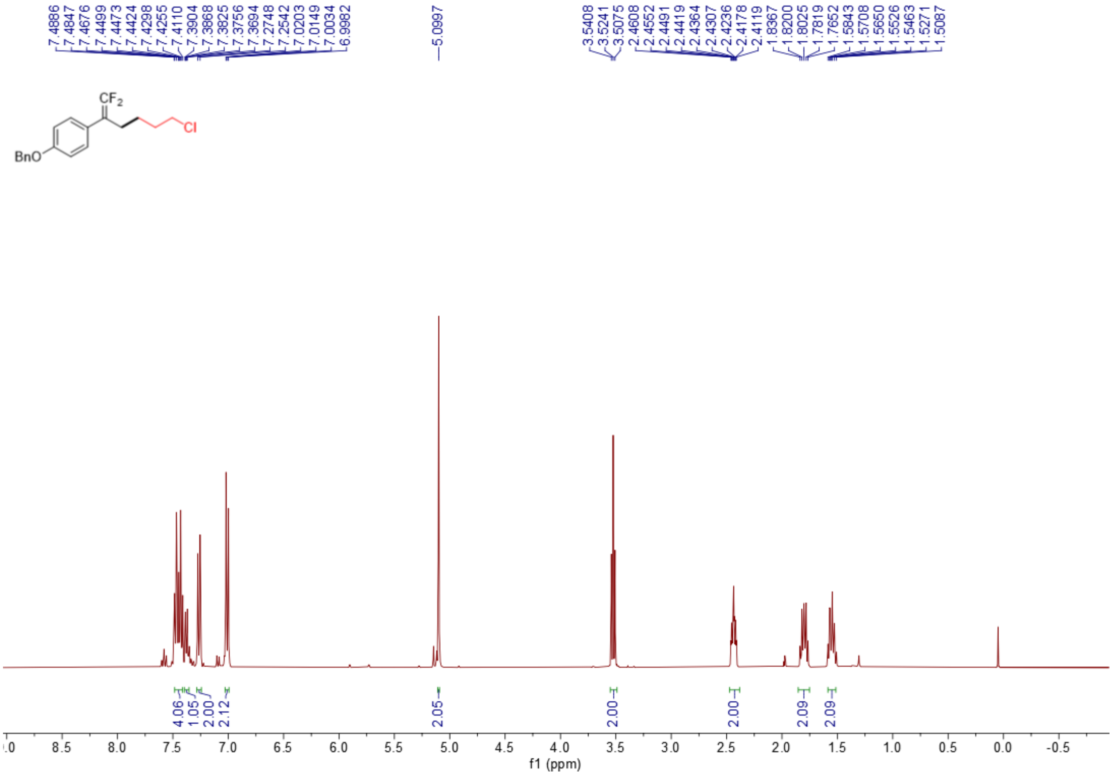

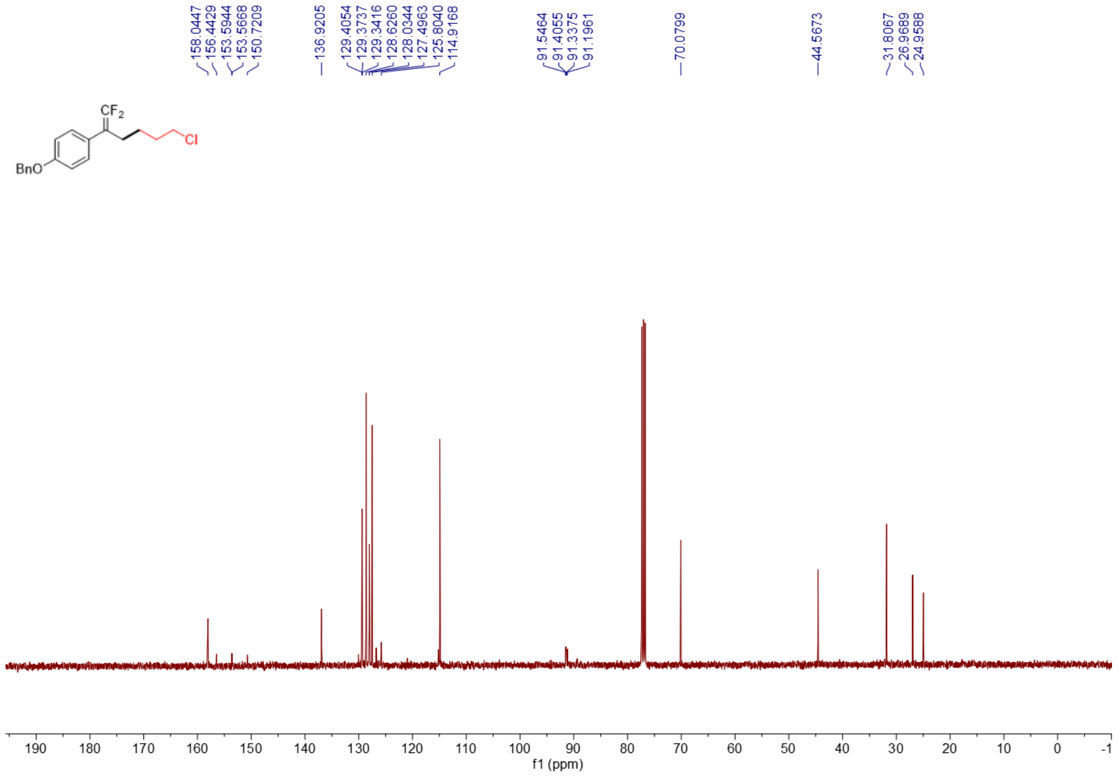

158.0447
156.4429
153.5944
153.5668
150.7209
136.9205
129.4054
129.3737
129.3416
128.6260
128.0344
127.4963
125.8040
114.9168
91.5464
91.4055
91.3375
91.1961
70.0799
44.5673
31.8067
26.9689
24.9588
CF2
BnO
Cl
f1 (ppm)

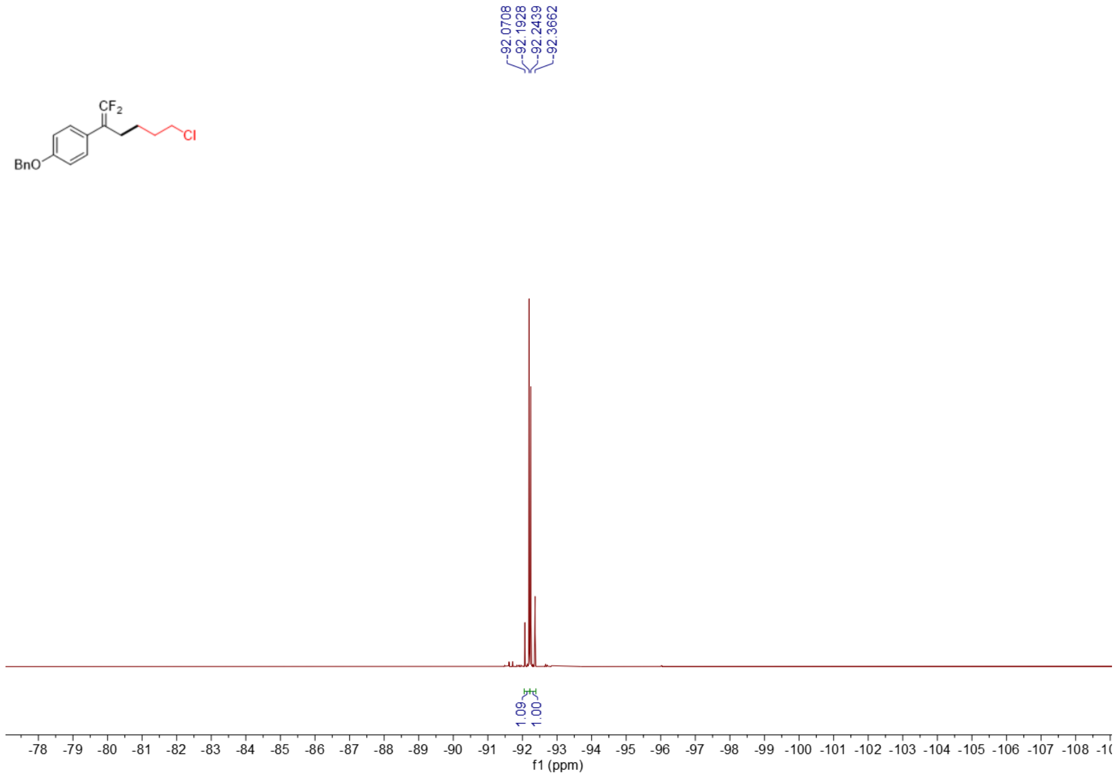

-92.0708
-92.1928
-92.2439
-92.3662
CF2
BnO
Cl
1.09
1.00
f1 (ppm)

NMR spectrum of 63

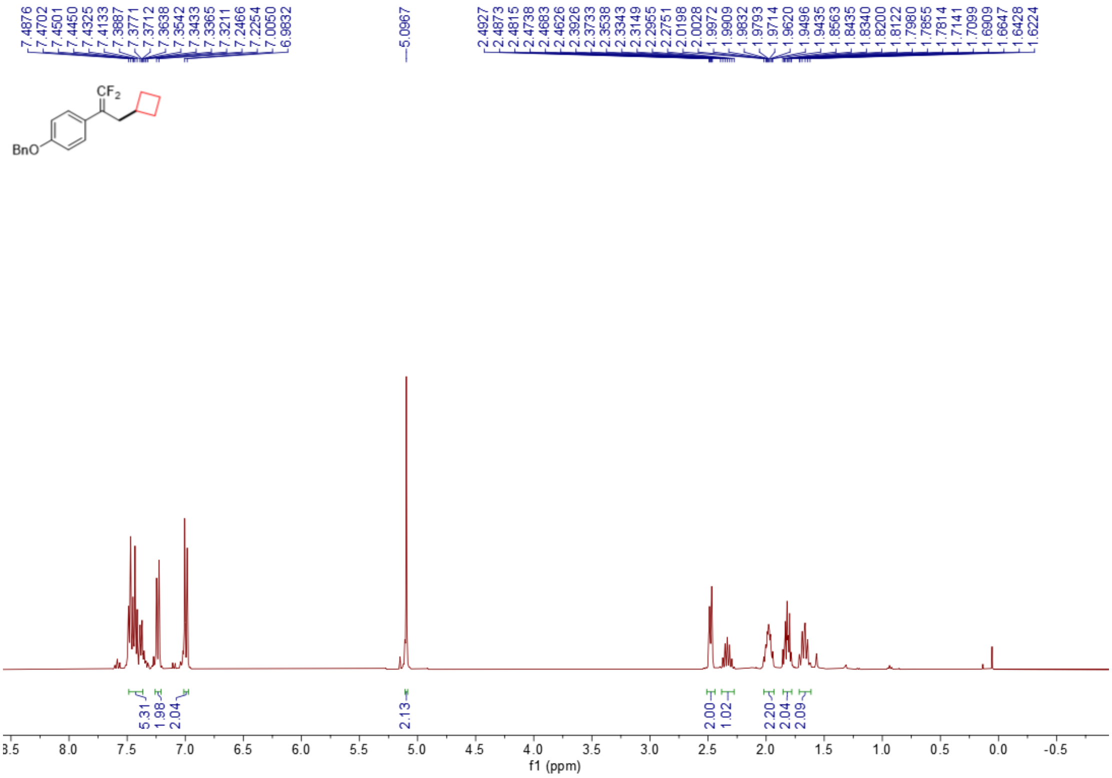

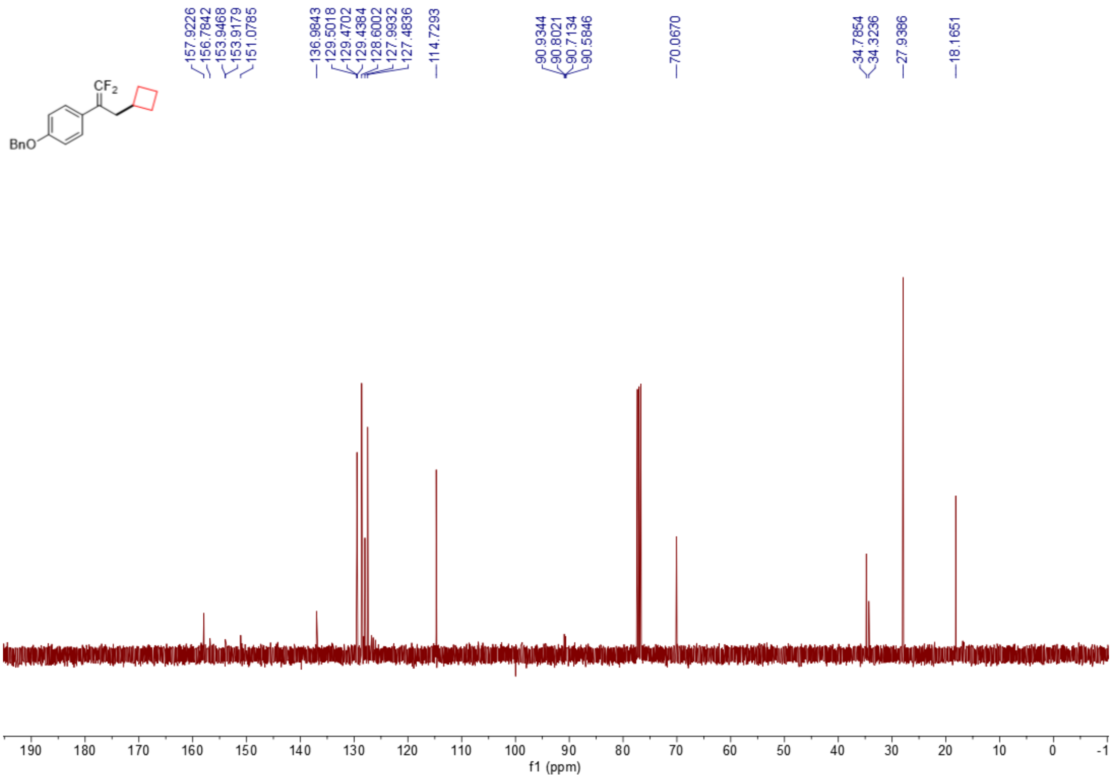

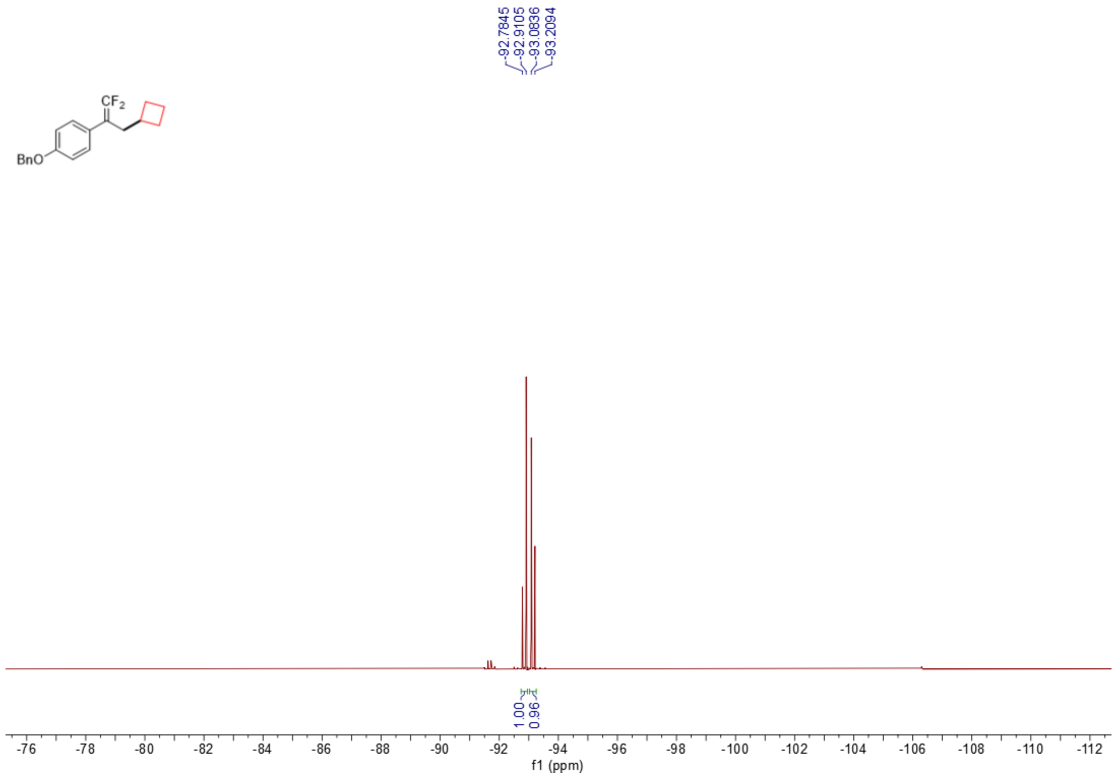

NMR spectrum of 64

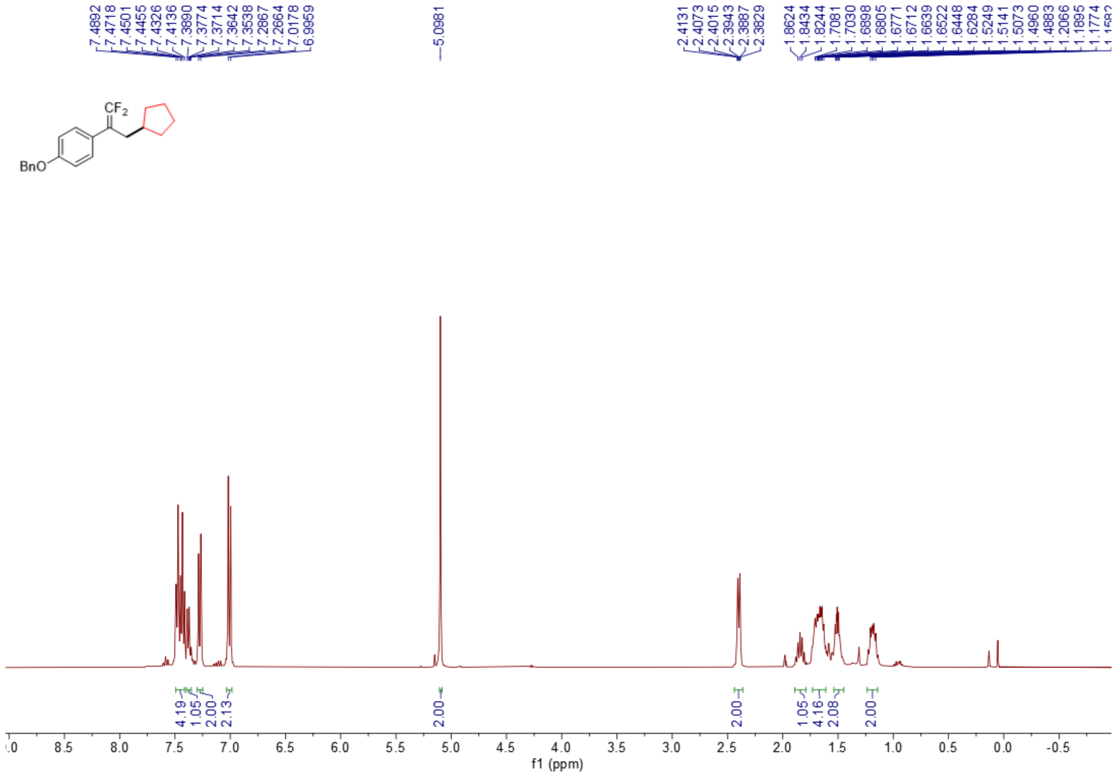

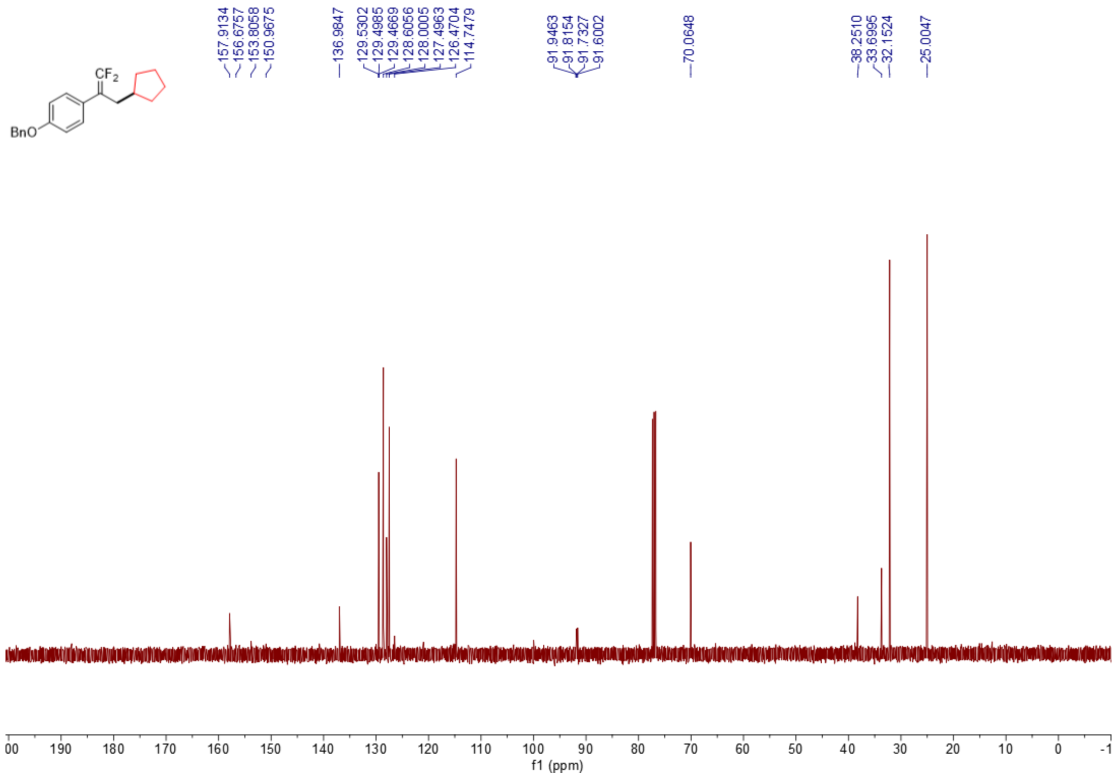

157.9134
156.6757
153.8058
150.9675
136.9847
129.5302
129.4985
129.4669
128.6056
128.0005
127.4963
126.4704
114.7479
91.9463
91.8154
91.7327
91.6002
70.0648
38.2510
33.6995
32.1524
25.0047
BnO
CF_2
f1 (ppm)

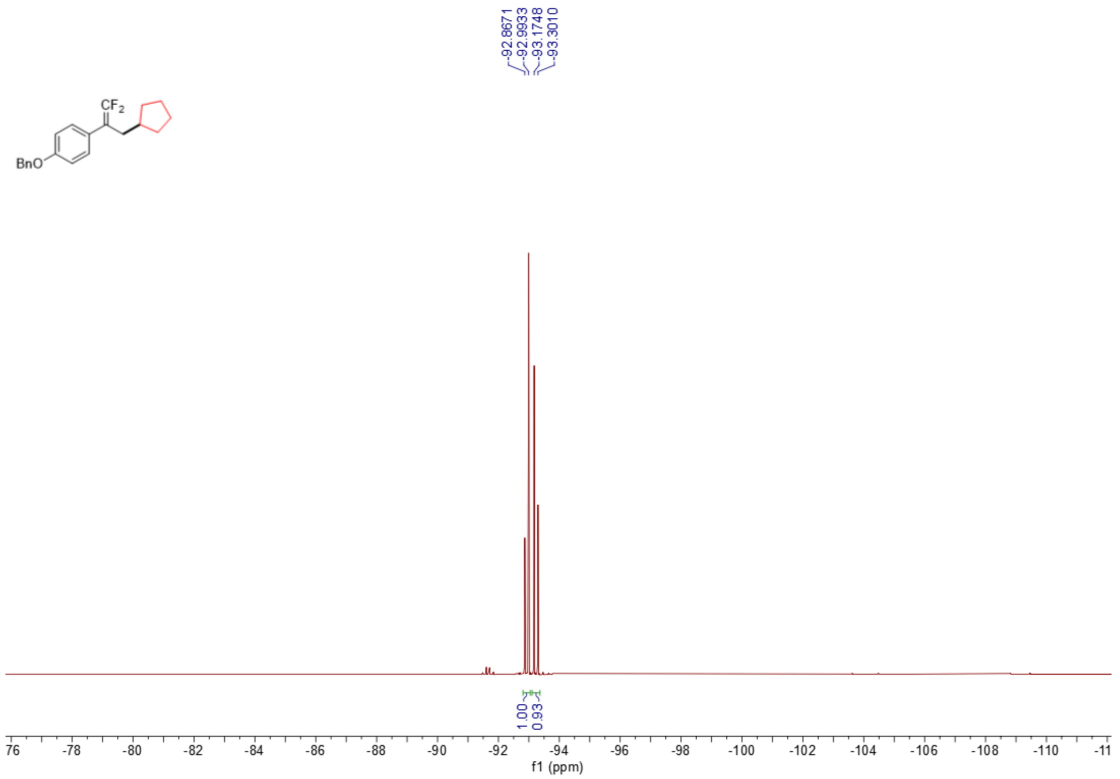

-92.8671
-92.9933
-93.1748
-93.3010
BnO
CF_2
1.00
0.93
f1 (ppm)

NMR spectrum of 65

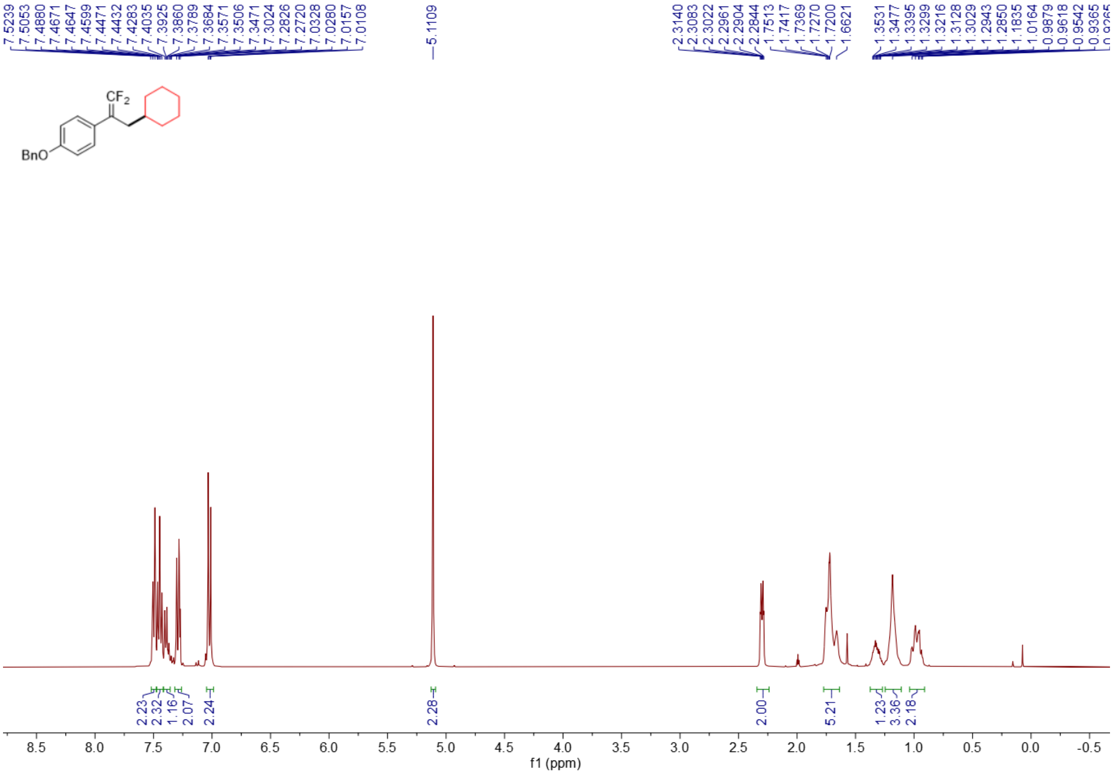

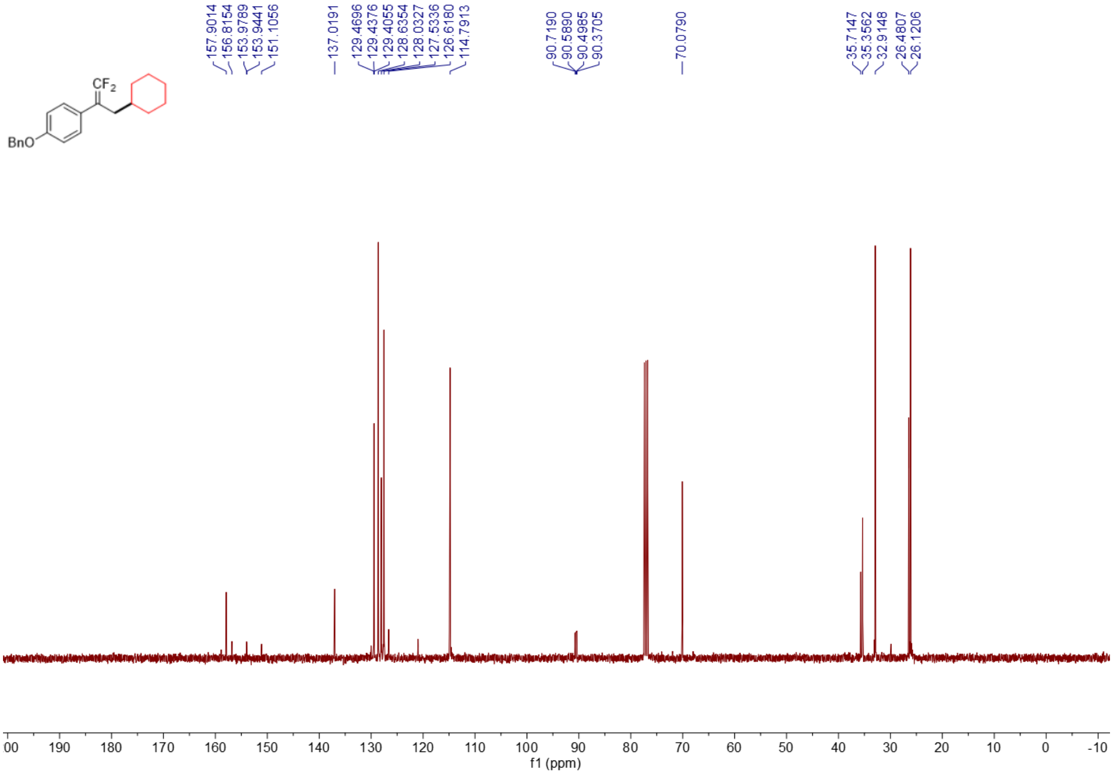

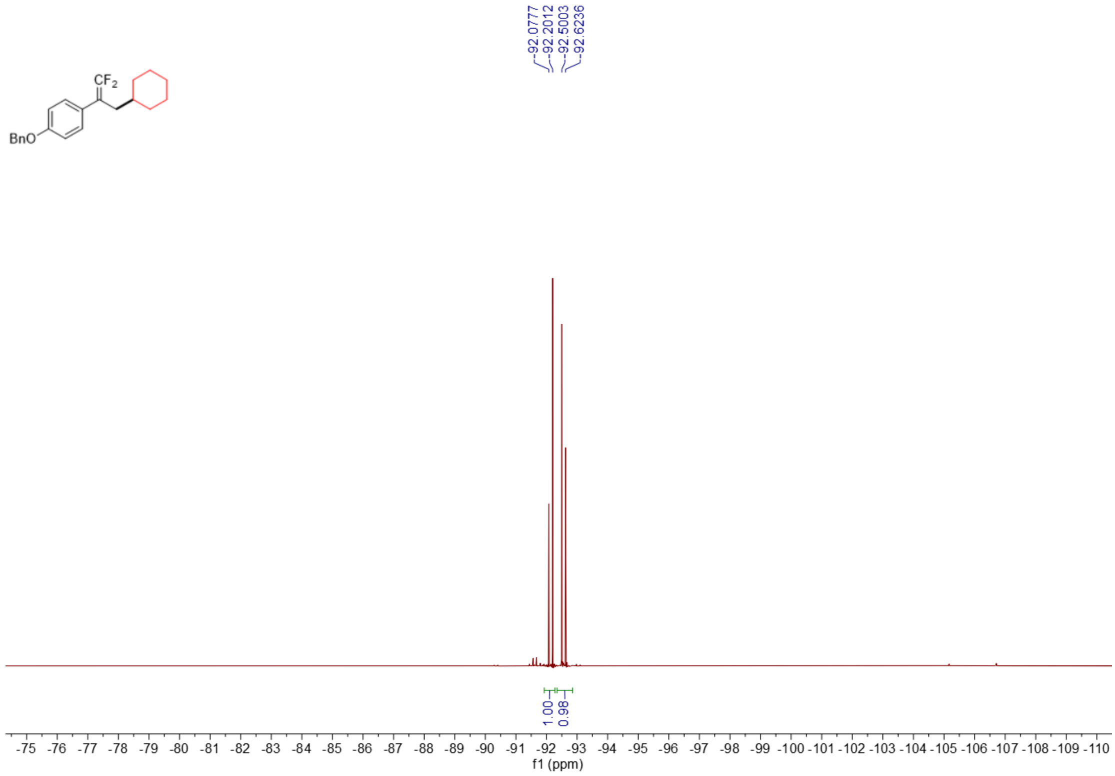

NMR spectrum of 66

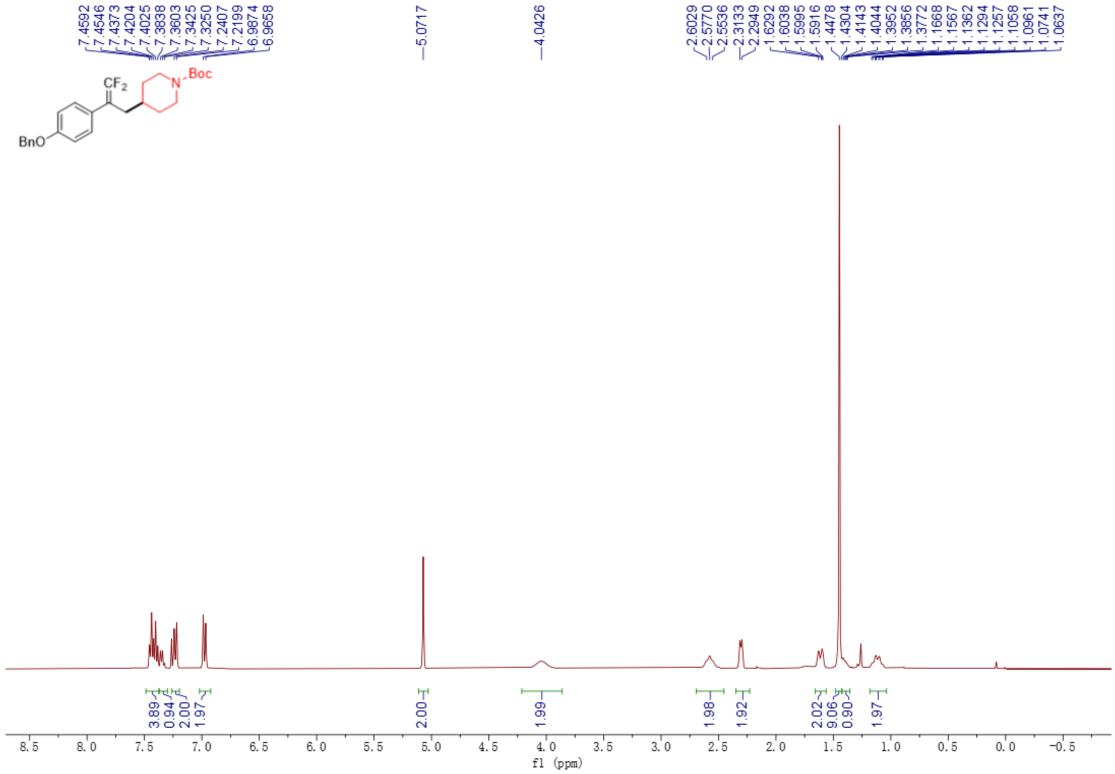

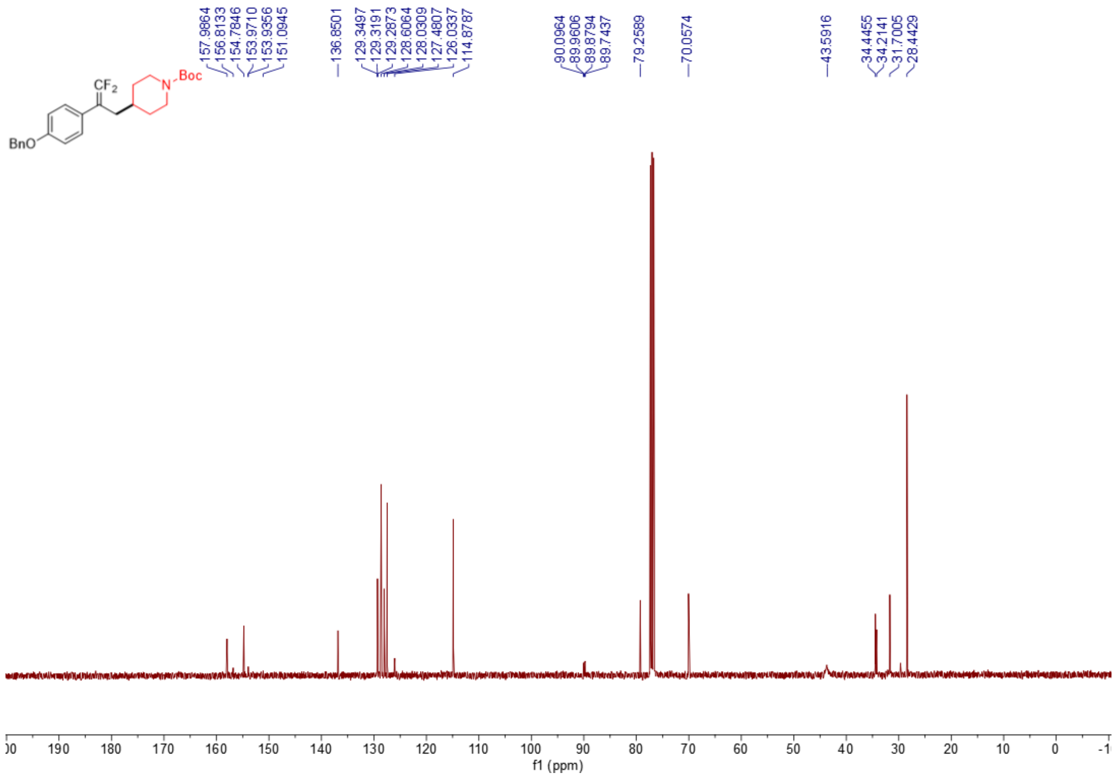
CF2
N
Boc
BnO
157.9864
156.8133
154.7846
153.9710
153.9356
151.0945
136.8501
129.3497
129.3191
129.2873
128.6064
128.0309
127.4807
126.0337
114.8787
90.0964
89.9606
89.8794
89.7437
79.2589
70.0574
43.5916
34.4455
34.2141
31.7005
28.4429
f1 (ppm)

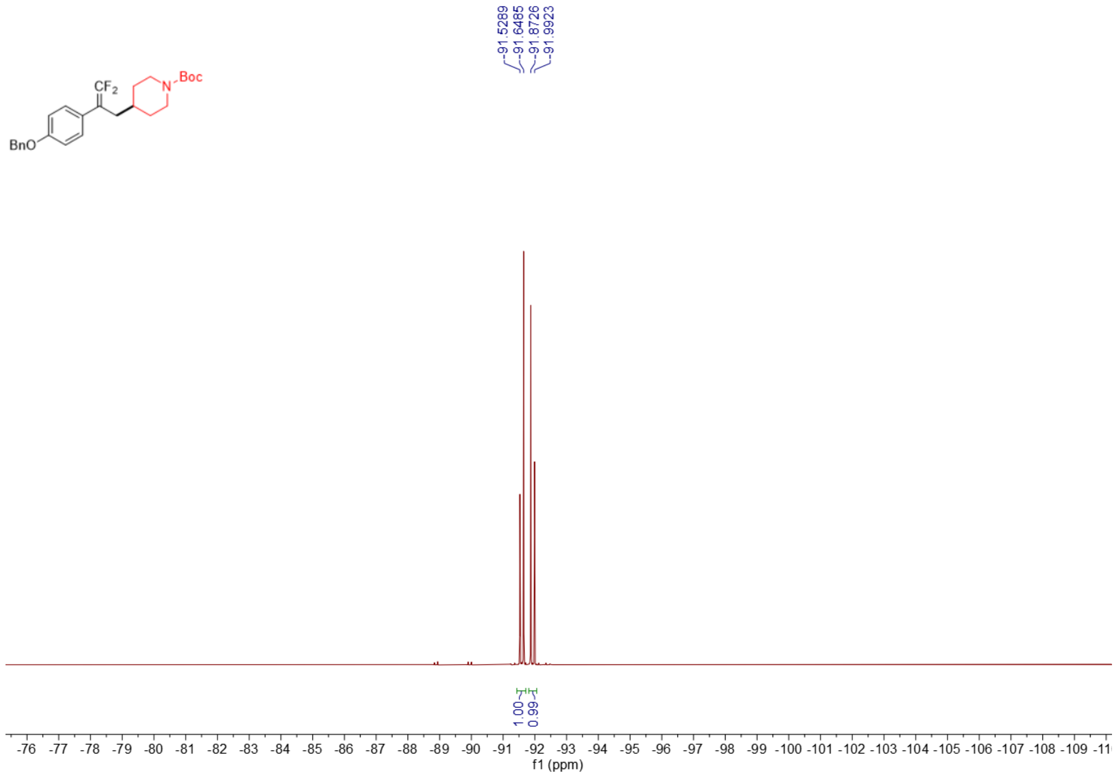
CF2
N
Boc
BnO
-91.5289
-91.6485
-91.8726
-91.9923
1.00
0.99
f1 (ppm)

NMR spectrum of 67

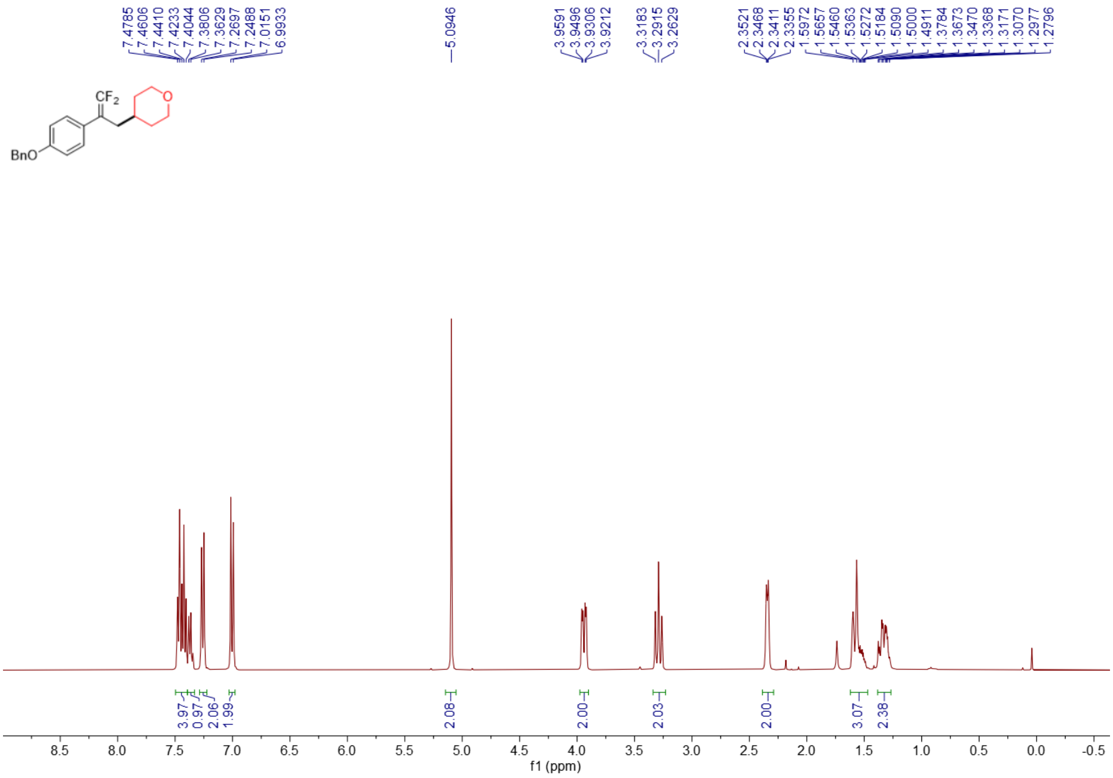

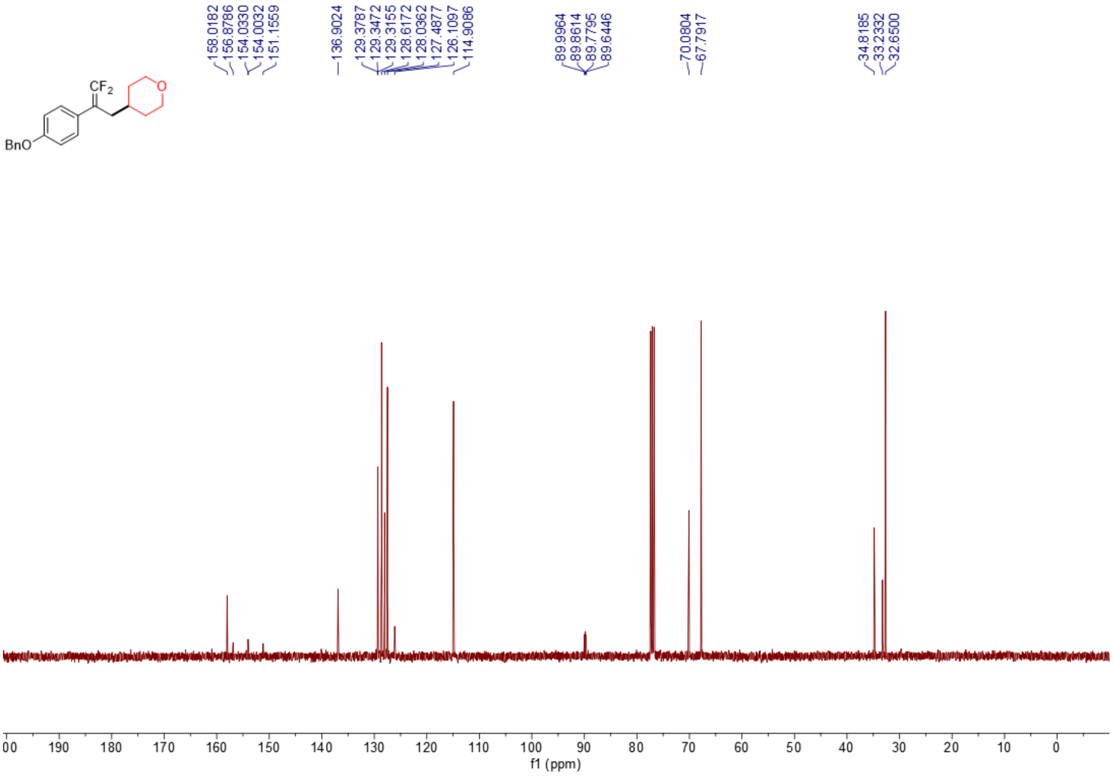

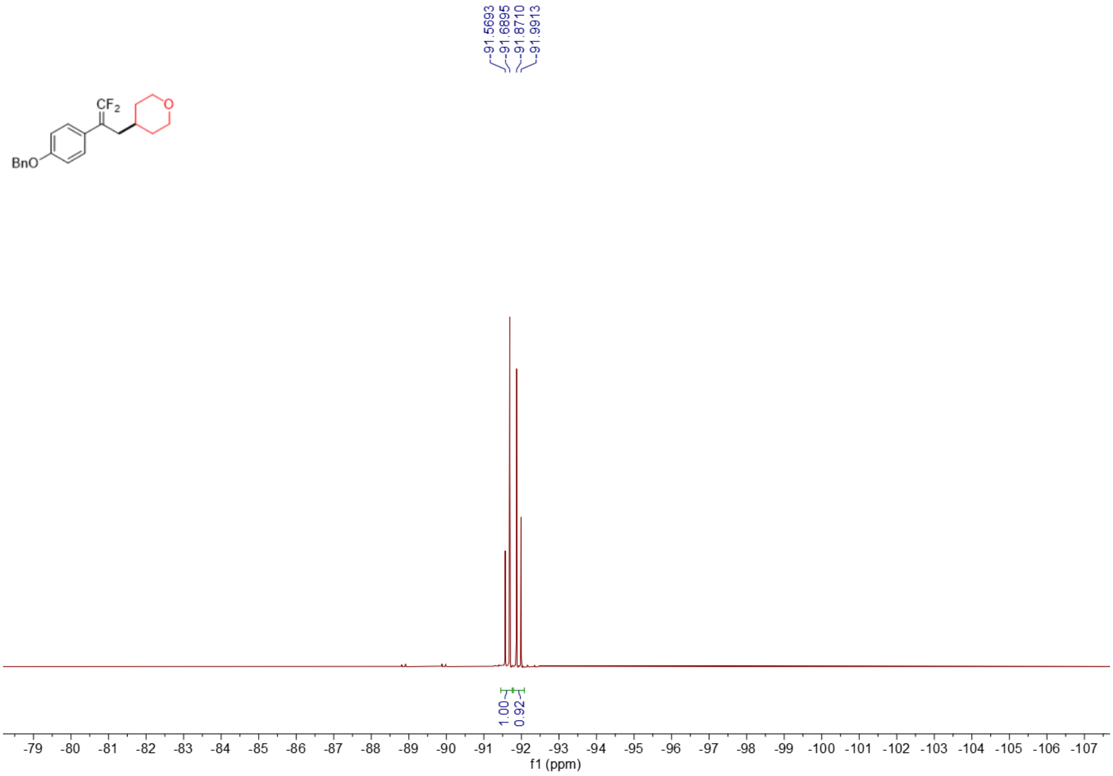

NMR spectrum of 68

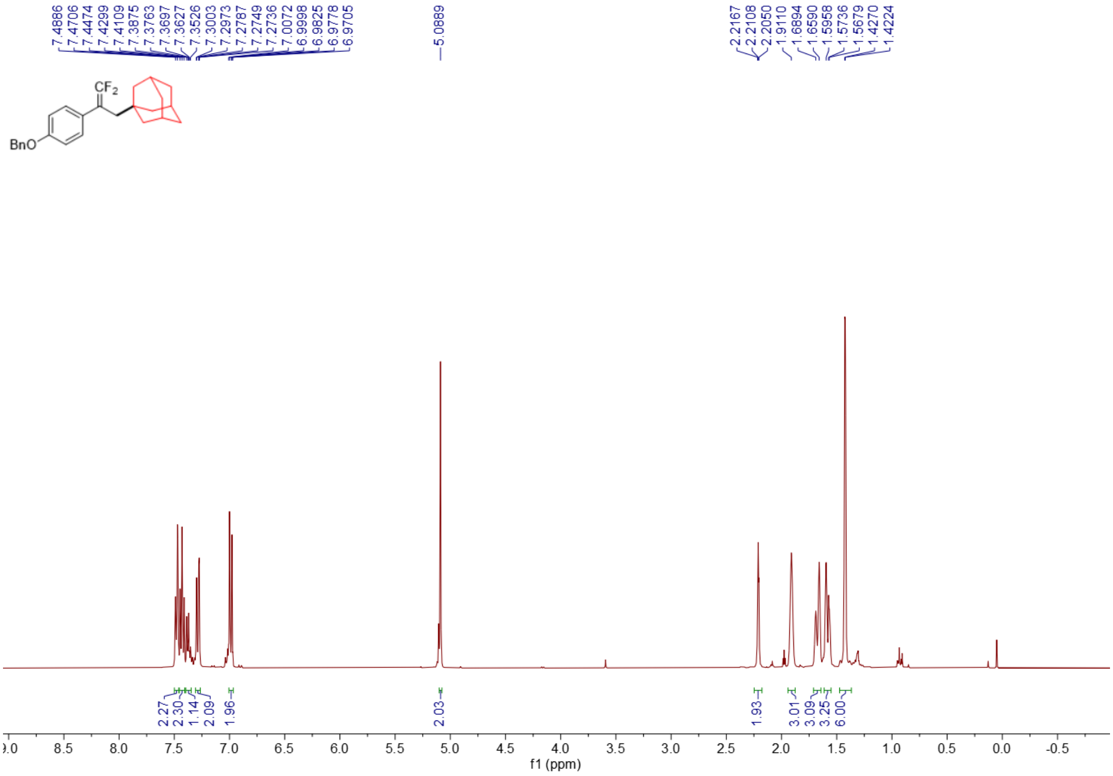

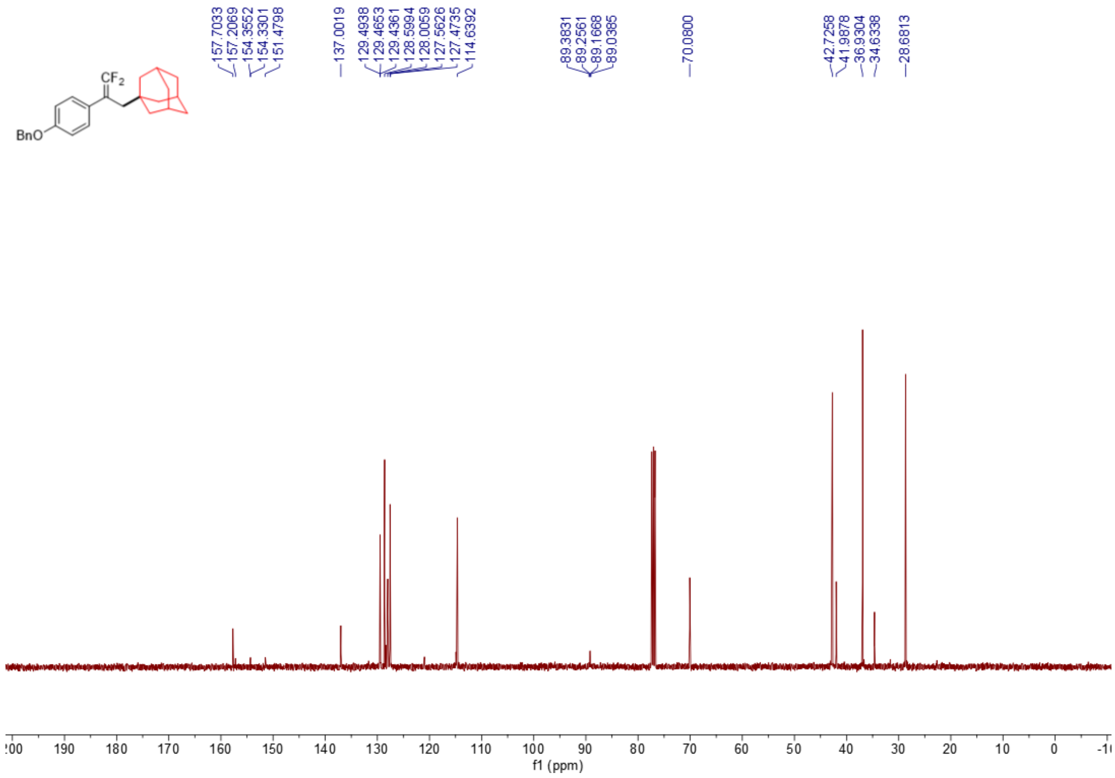
CF2
BnO
157.7033
157.2069
154.3552
154.3301
151.4798
137.0019
129.4938
129.4653
129.4361
128.5994
128.0059
127.5626
127.4735
114.6392
89.3831
89.2561
89.1668
89.0385
70.0800
42.7258
41.9878
36.9304
34.6338
28.6813
f1 (ppm)

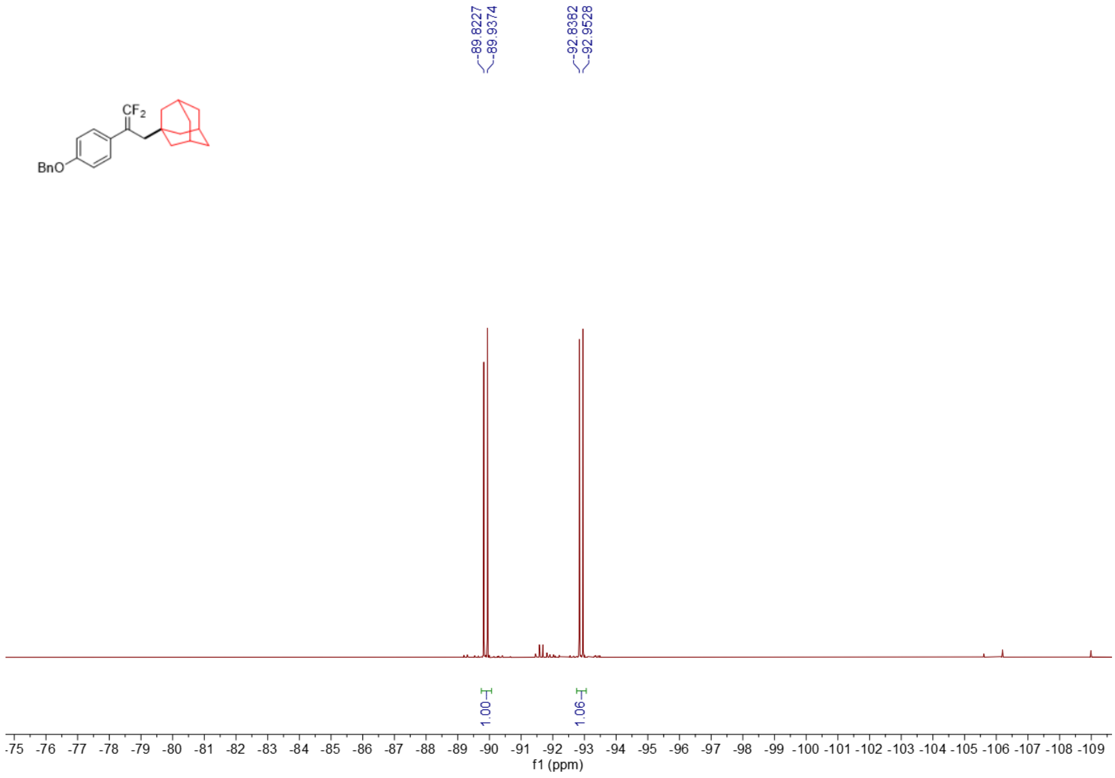
CF2
BnO
-89.8227
-89.9374
-92.8382
-92.9528
1.00
1.06
f1 (ppm)

NMR spectrum of 69

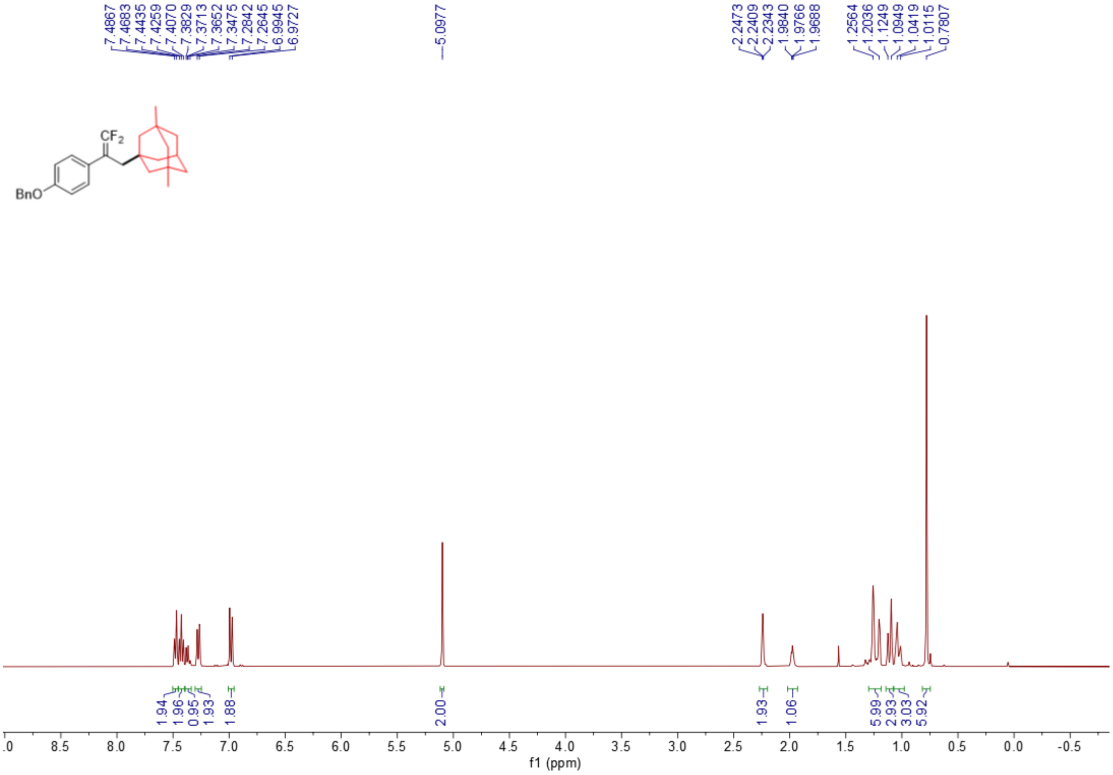

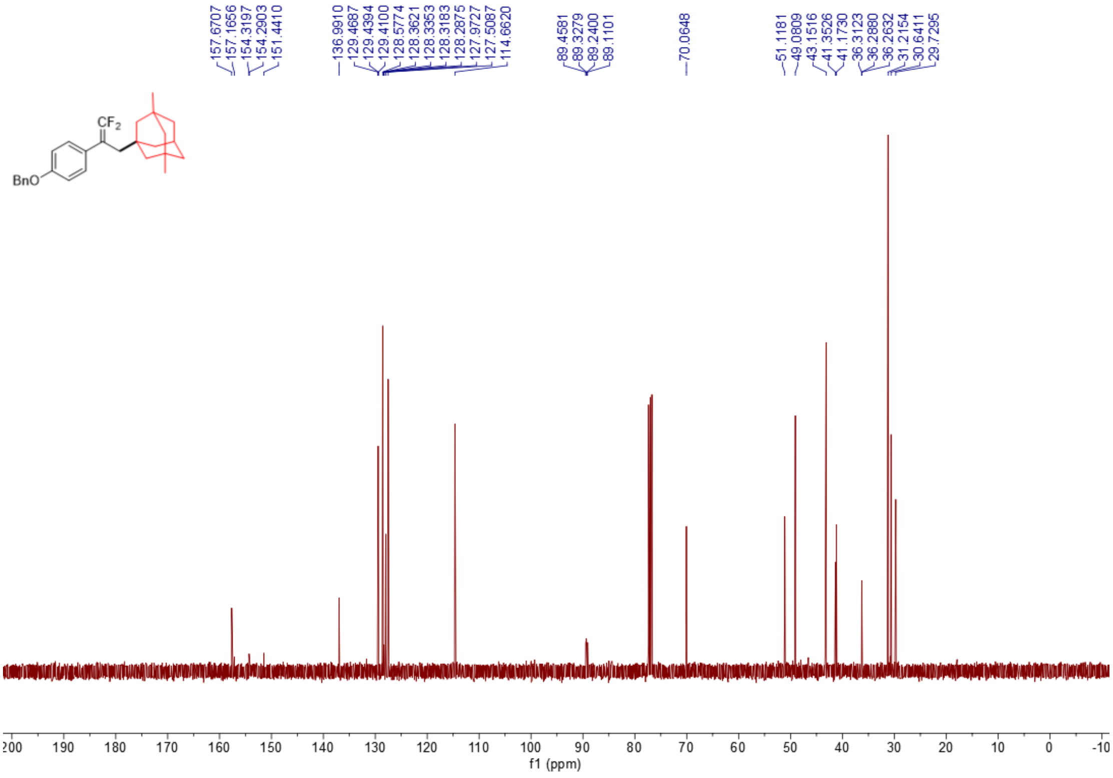

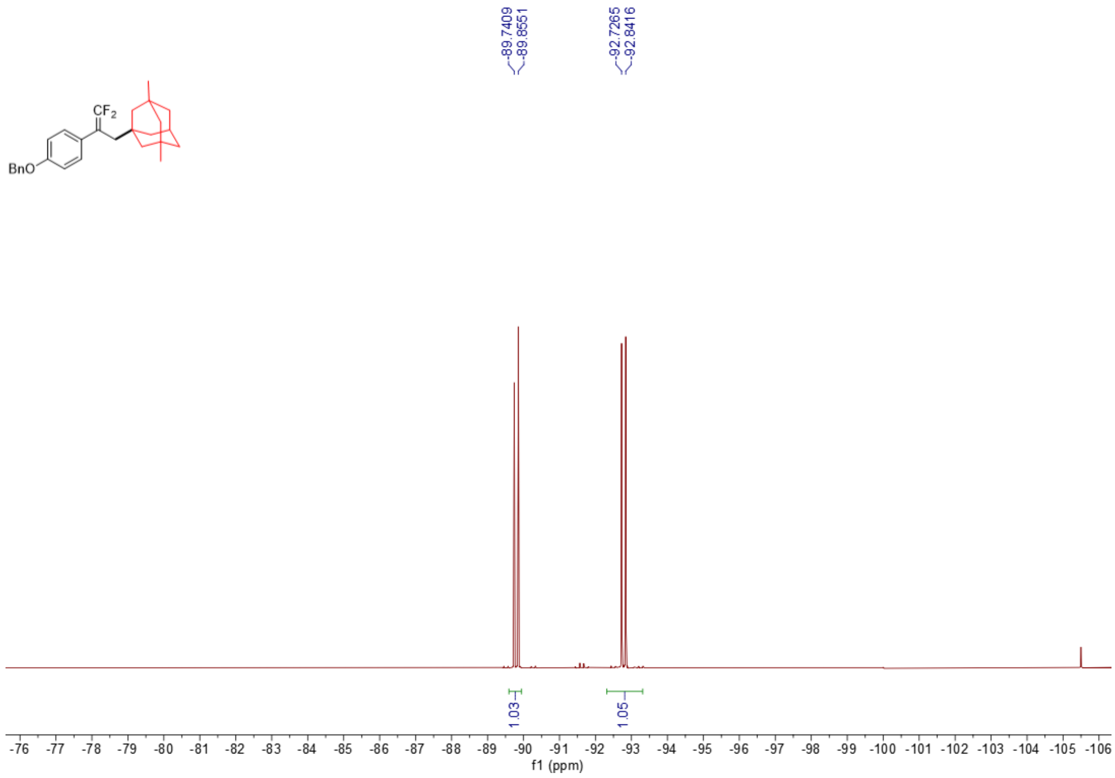

NMR spectrum of 70

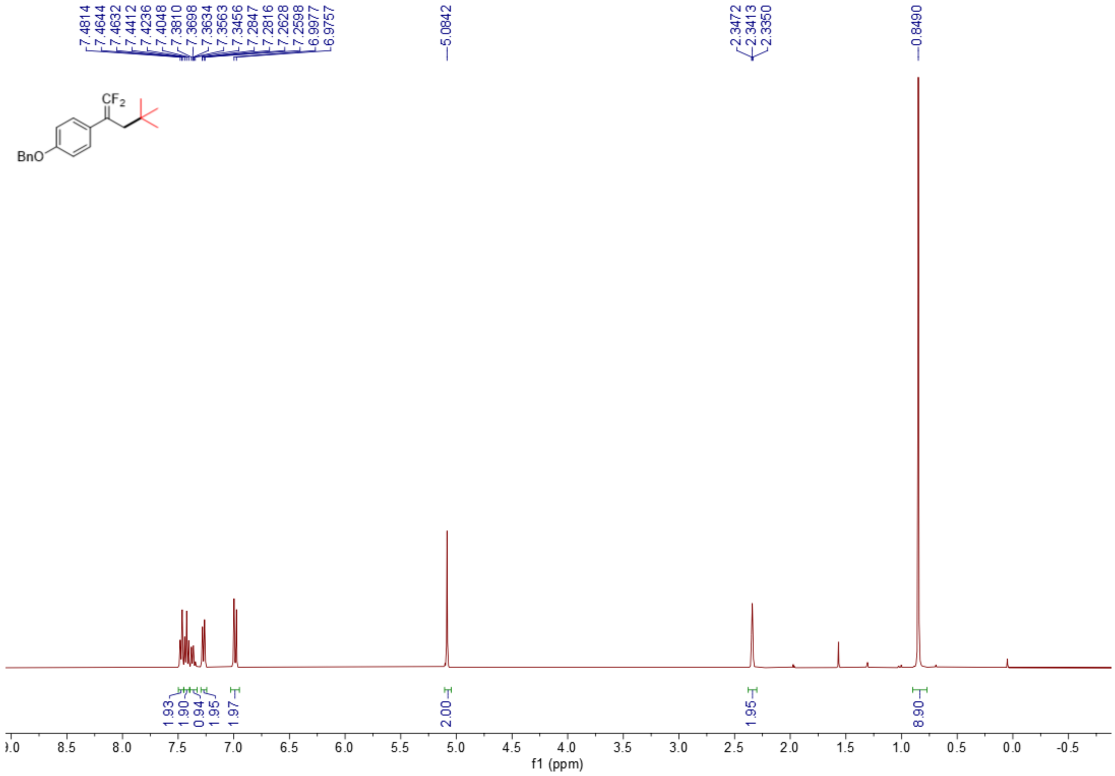

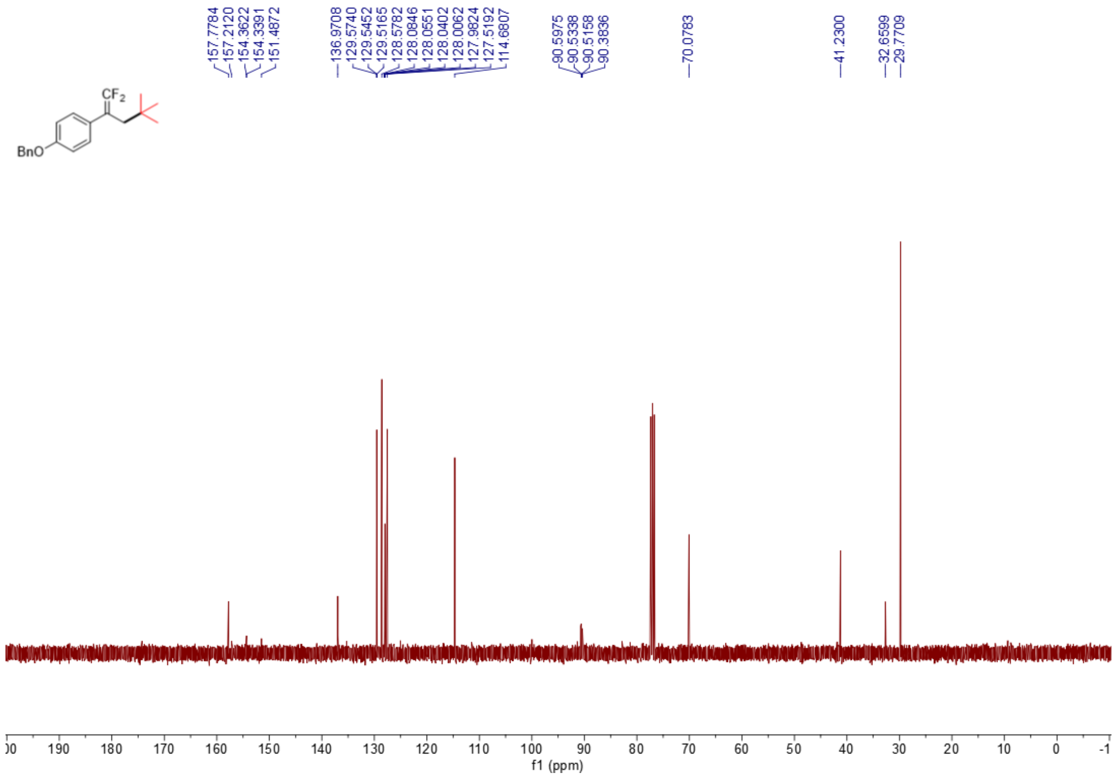

CF2
BnO
157.7784
157.2120
154.3622
154.3391
151.4872
136.9708
129.5740
129.5452
129.5165
128.5782
128.0846
128.0551
128.0402
128.0062
127.9824
127.5192
114.6807
90.5975
90.5338
90.5158
90.3836
70.0783
41.2300
32.6599
29.7709
f1 (ppm)

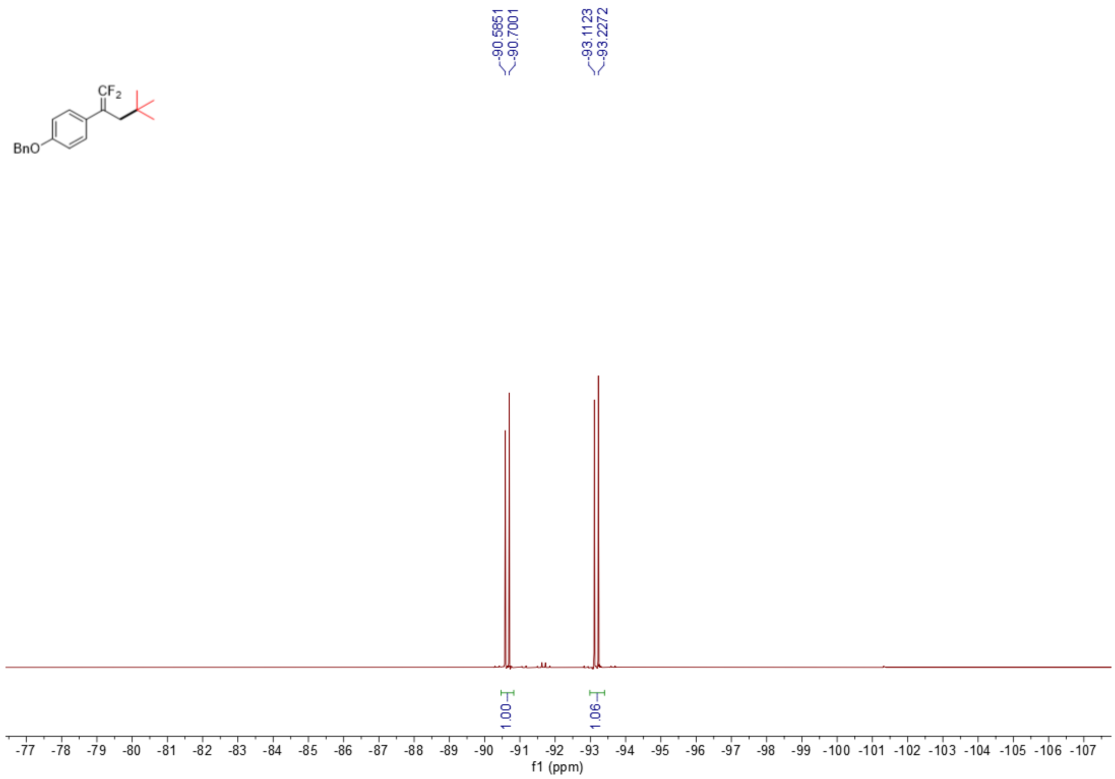

CF2
BnO
-90.5851
-90.7001
-93.1123
-93.2272
1.00
1.06
f1 (ppm)

NMR spectrum of 71

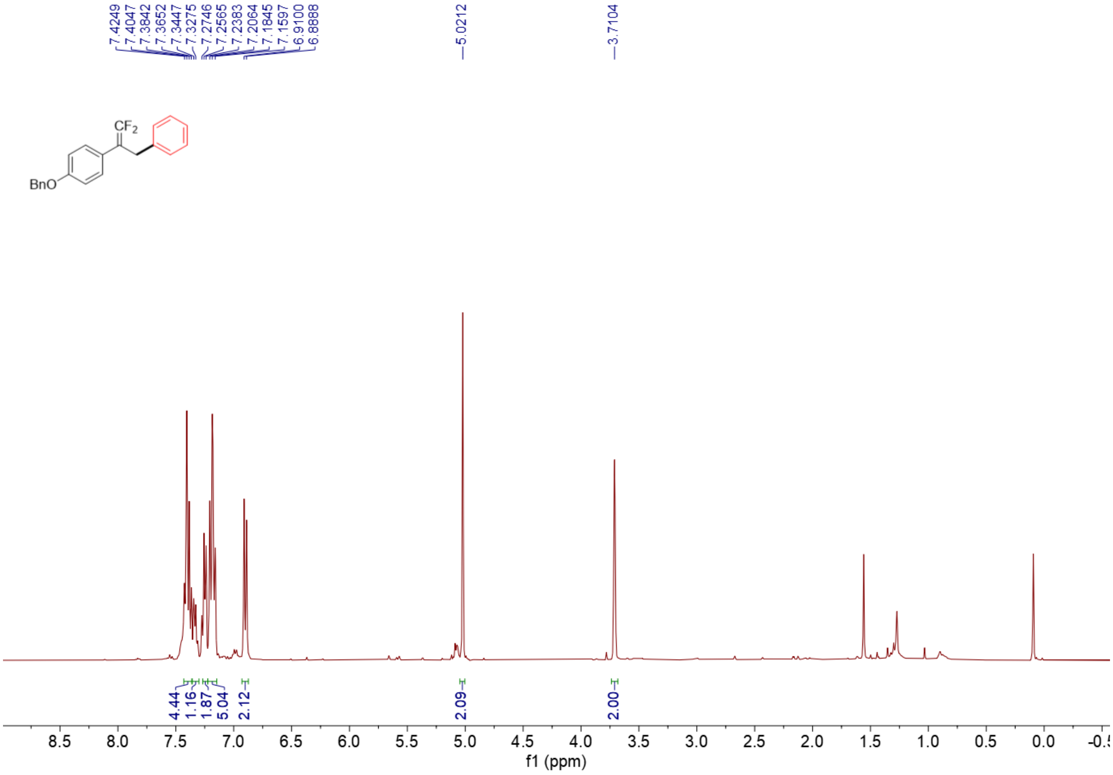

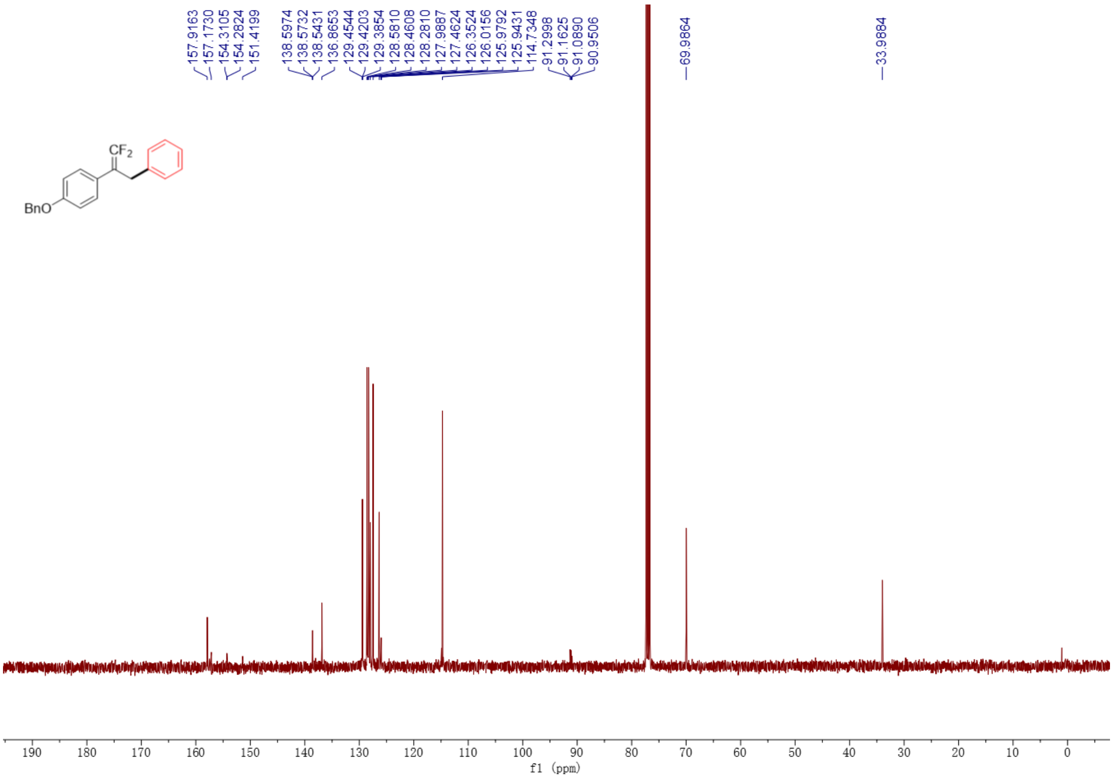

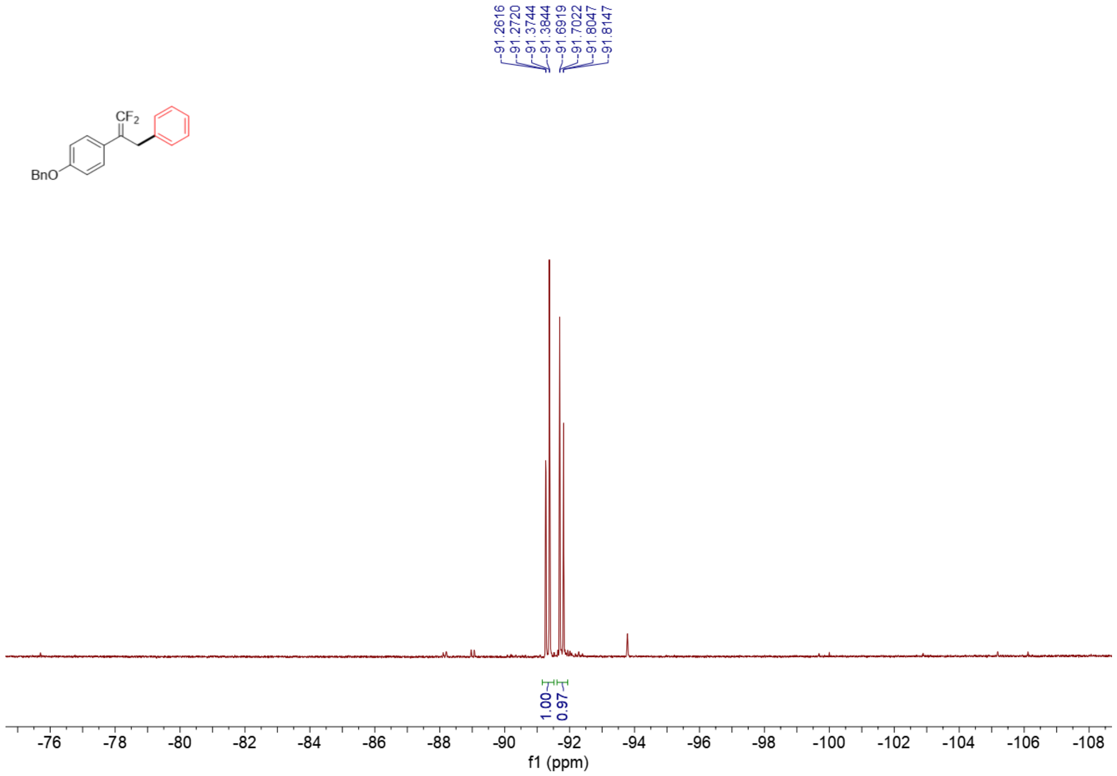

NMR spectrum of 72

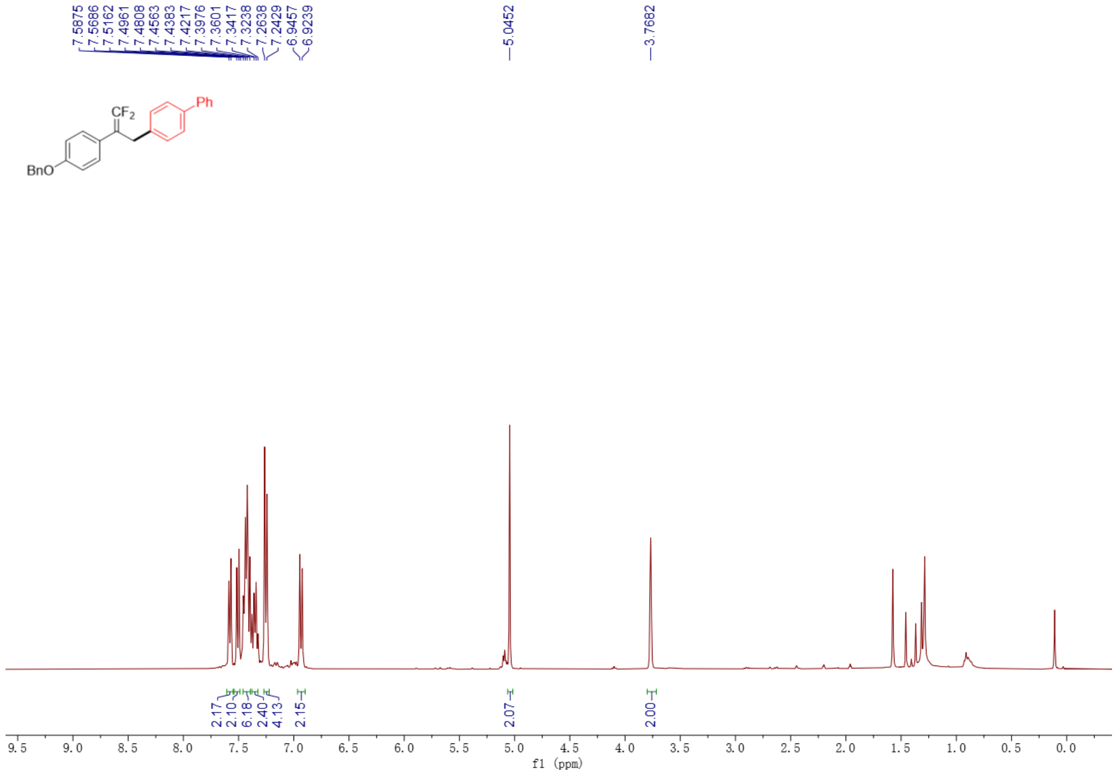

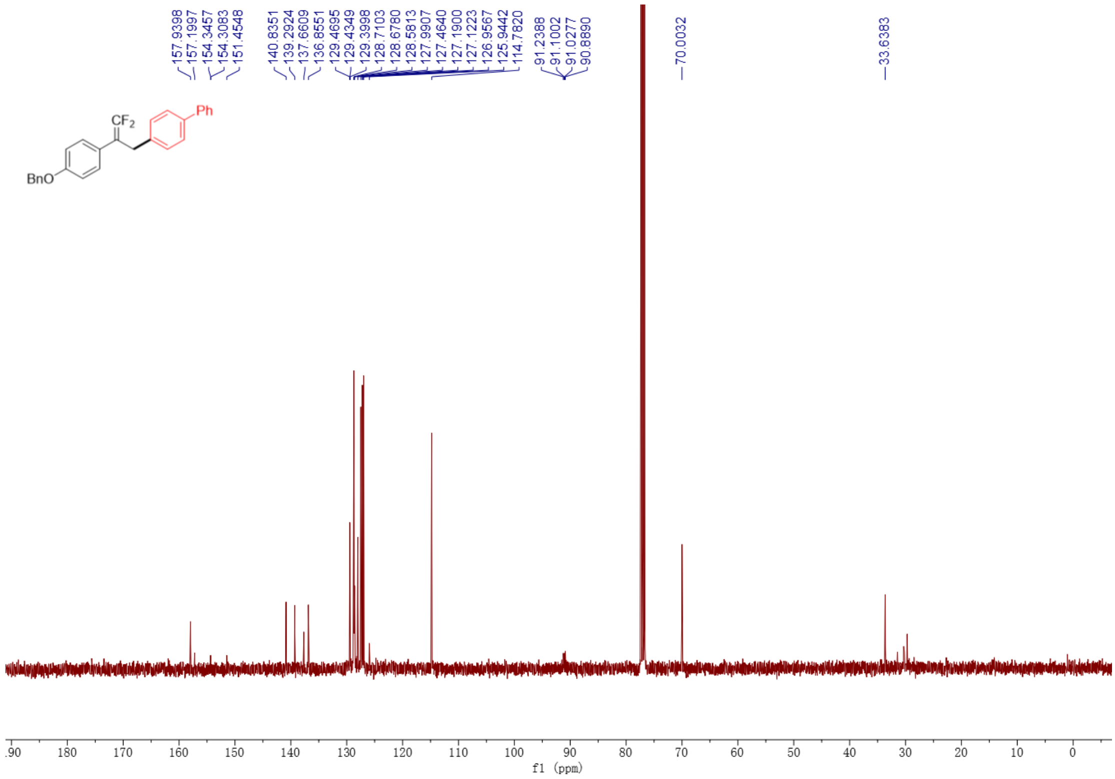
157.9398
157.1997
154.3457
154.3083
151.4548
140.8351
139.2924
137.6609
136.8551
129.4695
129.4349
129.3998
128.7103
128.6780
128.5813
127.9907
127.4640
127.1900
127.1223
126.9567
125.9442
114.7820
91.2388
91.1002
91.0277
90.8890
70.0032
33.6383
CF2
Ph
BnO
190 180 170 160 150 140 130 120 110 100 90 80 70 60 50 40 30 20 10 0
f1 (ppm)

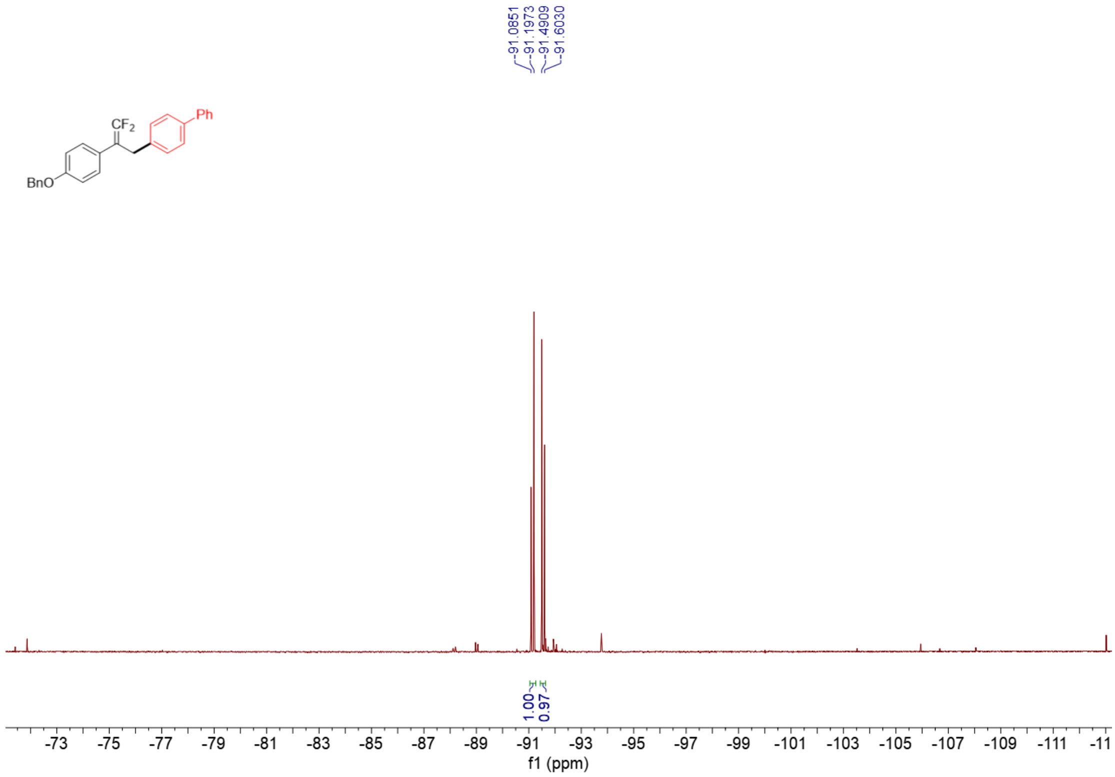
-91.0851
-91.1973
-91.4909
-91.6030
CF2
Ph
BnO
1.00
0.97
-73 -75 -77 -79 -81 -83 -85 -87 -89 -91 -93 -95 -97 -99 -101 -103 -105 -107 -109 -111 -11
f1 (ppm)

NMR spectrum of 73

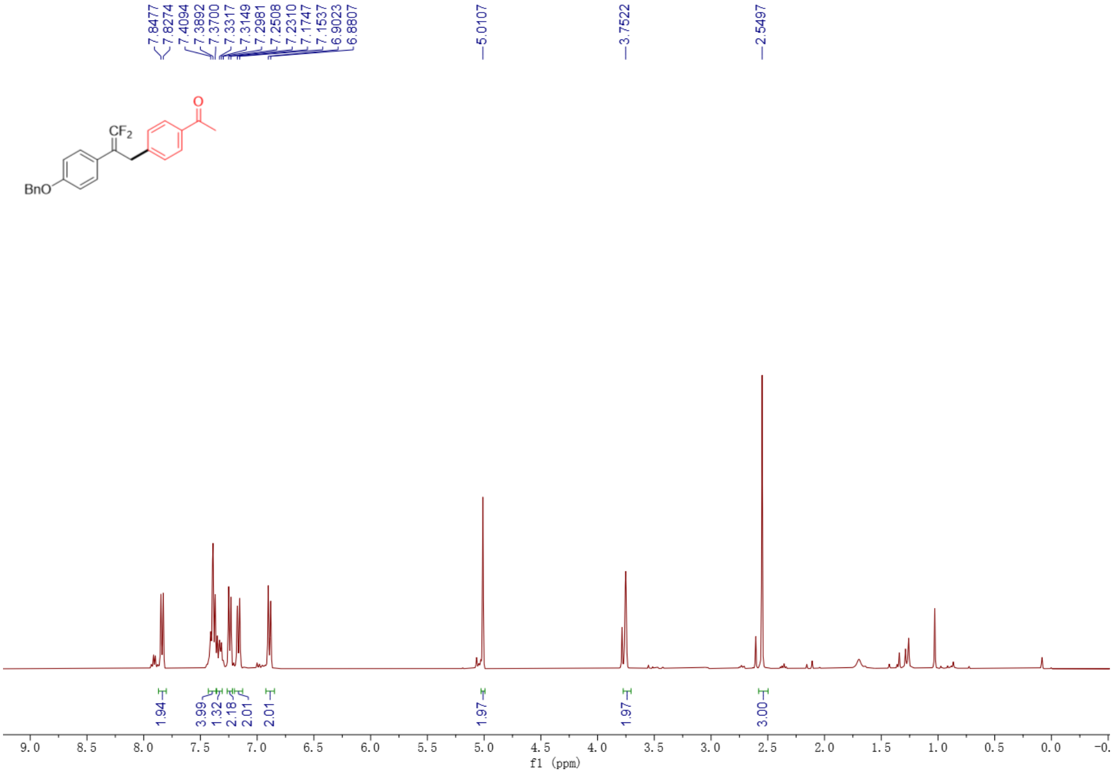

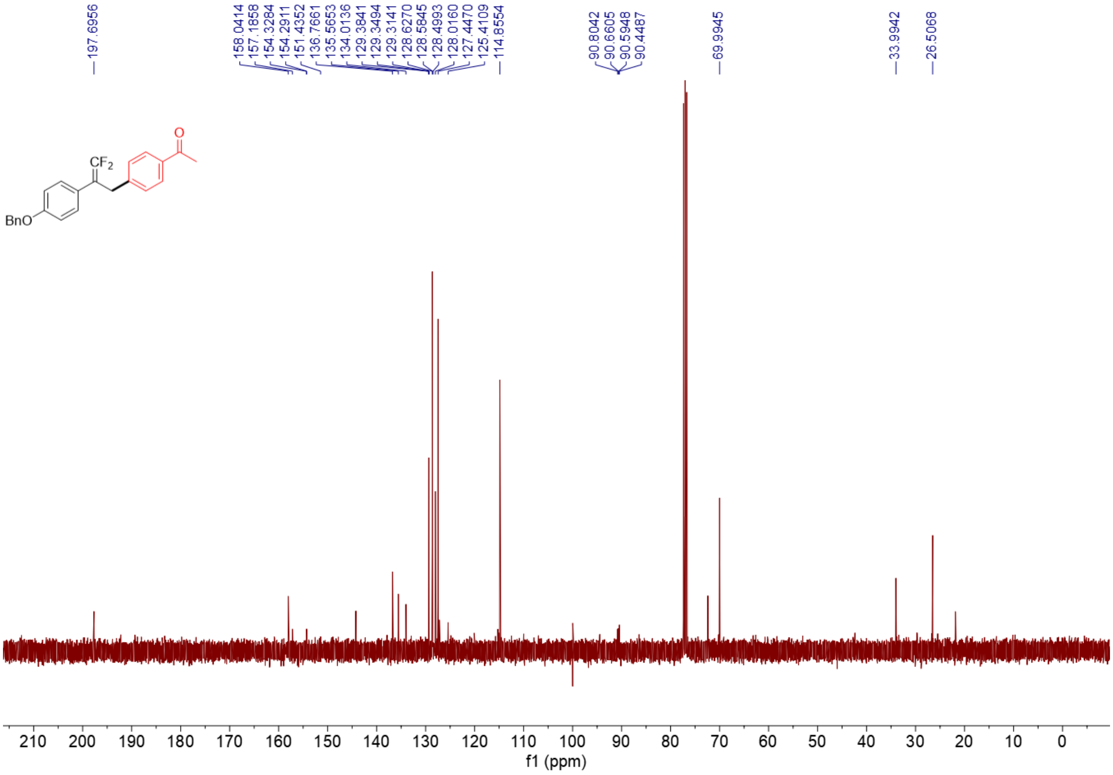

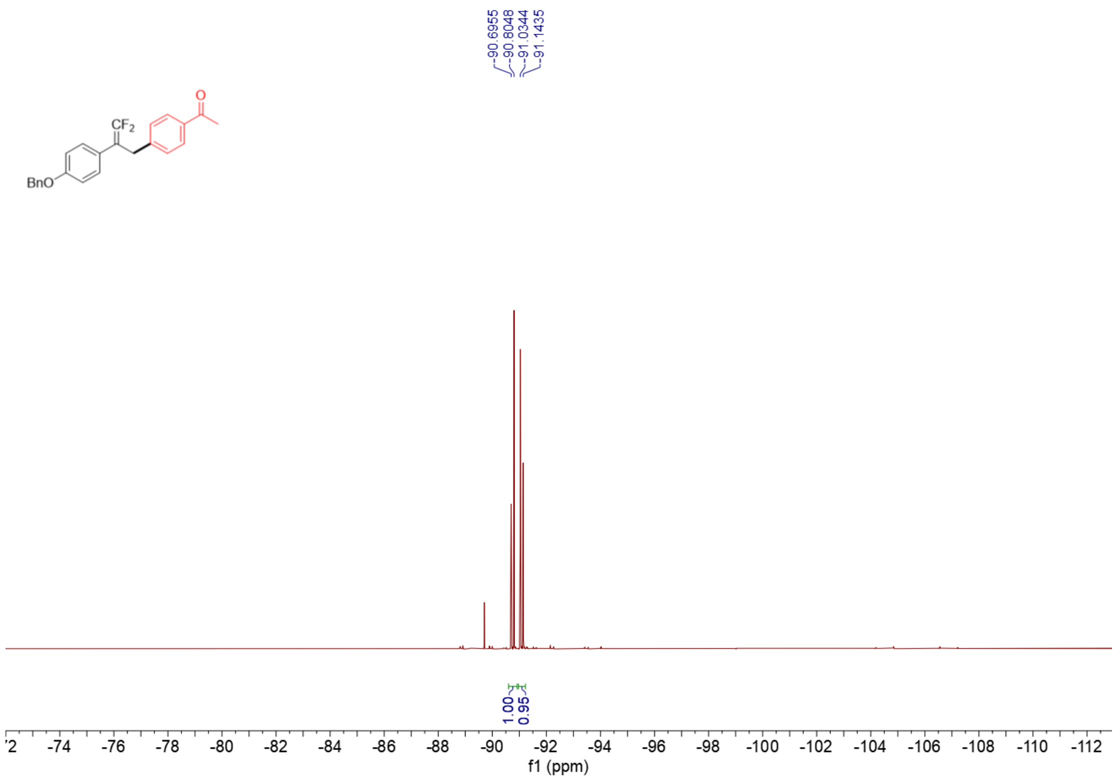

-90.6955
-90.8048
-91.0344
-91.1435
CF2
BnO
O
1.00
0.95
-74
-76
-78
-80
-82
-84
-86
-88
-90
-92
-94
-96
-98
-100
-102
-104
-106
-108
-110
-112
f1 (ppm)
